# RNA-seq and metabolomic analyses of beneficial plant phenol biochemical pathways in red alder

**DOI:** 10.3389/fpls.2024.1349635

**Published:** 2024-11-07

**Authors:** Kim K. Hixson, Qingyan Meng, Syed G. A. Moinuddin, Mi Kwon, Michael A. Costa, John R. Cort, Laurence B. Davin, Callum J. Bell, Norman G. Lewis

**Affiliations:** ^1^ Institute of Biological Chemistry, Washington State University, Pullman, WA, United States; ^2^ Environmental Molecular Sciences Laboratory, Pacific Northwest National Laboratory, Richland, WA, United States; ^3^ Biological Sciences Division, Pacific Northwest National Laboratory, Richland, WA, United States; ^4^ National Center for Genome Resources, Santa Fe, NM, United States

**Keywords:** red alder (*Alnus rubra*), Betulaceae, metabolomics, RNA-seq, transcriptomics, proanthocyanidins, ellagitannins, diarylheptanoids

## Abstract

Red alder (*Alnus rubra*) has highly desirable wood, dye pigment, and (traditional) medicinal properties which have been capitalized on for thousands of years, including by Pacific West Coast Native Americans. A rapidly growing tree species native to North American western coastal and riparian regions, it undergoes symbiosis with actinobacterium *Frankia* via their nitrogen-fixing root nodules. Red alder’s desirable properties are, however, largely attributed to its bioactive plant phenol metabolites, including for plant defense, for its attractive wood and bark coloration, and various beneficial medicinal properties. Integrated transcriptome and metabolome data analyses were carried out using buds, leaves, stems, roots, and root nodules from greenhouse grown red alder saplings with samples collected during different time-points (Spring, Summer, and Fall) of the growing season. Pollen and catkins were collected from field grown mature trees. Overall plant phenol biochemical pathways operative in red alder were determined, with a particular emphasis on potentially identifying candidates for the long unknown gateway entry points to the proanthocyanidin (PA) and ellagitannin metabolic classes, as well as in gaining better understanding of the biochemical basis of diarylheptanoid formation, i.e. that help define red alder’s varied medicinal uses, and its extensive wood and dye usage.

## 1 Introduction

**Figure 1:**
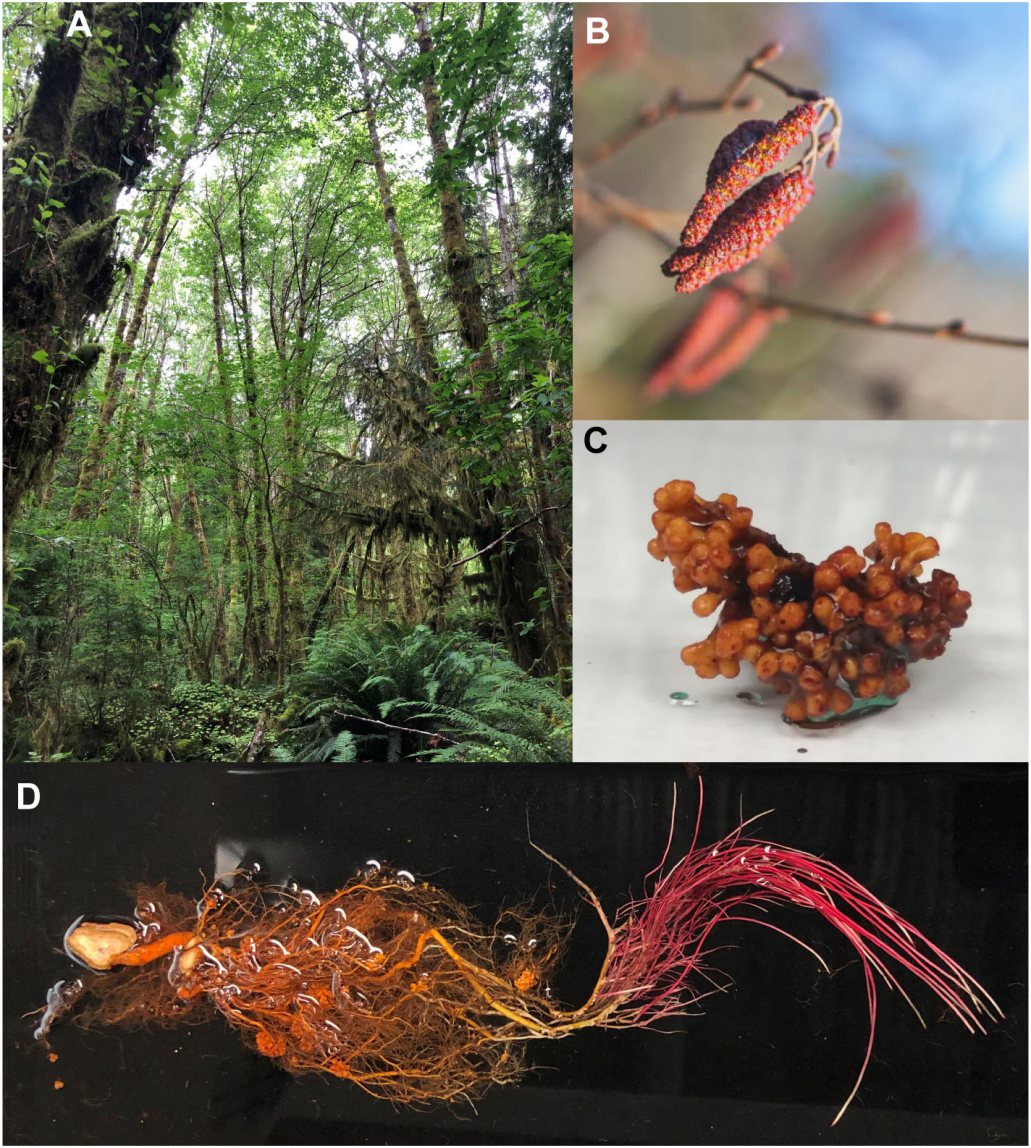
Images of red alder trees, catkins, root nodules and roots. **(A)** Red alder grove. **(B)** Male catkins. **(C)** Nodule clusters showing orange/brown pigments. **(D)** Roots from a 1-year-old Clone 639 red alder sapling. Photos credits: **(A)**, Laurence B. Davin; **(B)**, licensed through iStock.com (iStock.com/RomanKybus); **(C)**, Qingyan Meng; **(D)**, Tanya E. Winkler.

Alder (*Alnus*) tree species (family Betulaceae, order Fagales) are very important worldwide for their ecological roles and varied human uses. The genus *Alnus* has 44 species (WFO, 2022), the best known being Northern Hemisphere red alder (*A. rubra* Bong.) (**Figure 1A**) and black alder (*A. glutinosa* L.), respectively. Its timbers are highly prized, including for their remarkable stability when submerged and deeply sunk, slowly hardening to being stone-like (Gifford, 2001). Alders were long used in land reclamation for building man-made islands (e.g. crannogs, up to *ca* 5,000 years ago (Garrow and Sturt, 2019)) and later for submerged foundations for cities, such as Venice and Amsterdam (Klaassen and Creemers, 2012). Many such foundations remain viable to the present day. These remarkable properties also led to its present widespread use for lock gates, bridges, jetties, sluices, and pumps (Gifford, 2001).

In ancient times, Celtic mythology had alder signifying earth, fire, and water (Forest, 2014). Its tawny brown dye from twigs represented the classical “element” earth, its red colored slow-burning alder charcoal depicted fire, and its floral bright green dye signified water. In the Pacific Northwest (PNW), red alder bark and root dyes (orange, red, and reddish-brown) have long been widely used by coastal Native Americans to dye baskets, moccasins, hides, *etc.* Examples of red alder’s organ-specific orange-red pigmentation are shown in **Figures 1B–D**.

Red alder’s extensive ecological benefits has also resulted in its laudatory depiction as “Goddess Tree” (Alder Tree Spiritual Meaning) and “Healer of the Land” (Sati et al., 2011). Importantly, it significantly improves soil quality and fertility (Hart et al., 1997) via nitrogen (N)-fixation symbiosis with actinobacteria in the genus *Frankia*. This symbiosis can increase soil N-content annually in silvopastoral systems (Teklehaimanot and Mmolotsi, 2007) by *ca* 27.1 *kg* ha^–1^. Through this symbiosis, red alder can establish rapidly on exposed mineral soil, typically after land disturbances, hence its description as a pioneer species. Such attributes could potentially expand red alder timber and feedstock production on marginal lands (Mehmood et al., 2017).

Ethnobotanically, Native Americans have also long used red alder bark in traditional medicine, e.g. for headaches, congestion, colds, anemia, pain relief (salicin being similar to aspirin), rheumatic pains, internal injuries, and diarrhea (Turner and Hebda, 1990; Forlines et al., 1992). Bark poultices also relieve swelling, eczema, sores, and aches. Its bioactive plant phenols are considered the main contributors to its ethnobotanical use.

Commercially, red alder wood has a cherry-like, even-textured, grain which is extensively used for furniture, cabinets, millwork, veneers, paneling, plywood, pallets, pulp/paper, and other secondary manufactured products. Alder use in musical instruments is also well documented. Historically, this has ranged from Celtic Druid pipes to red alder Stratocaster electric guitar bodies being used by musicians, such as Eric Clapton and the late Jimi Hendrix (Runwal, 2020).

Red alder is thus an excellent species to probe its myriad beneficial properties, particularly as regards its bioactive plant phenols which represent much of its highly successful phytochemical defenses against opportunistic pathogens, as well as for its wood, medicinal, and reddish-orange dye properties (**Figures 1B–D**). Its bioactives include diarylheptanoids, ellagitannins, flavonoids, and proanthocyanidins (PAs), all being essentially shikimate-phenylalanine and phenylpropanoid/phenylpropanoid-acetate pathway derived.

Here, we report integrated transcriptome and metabolome data analyses from five different red alder Clone 639 sapling tissues (buds, leaves, stems, roots, and root nodules) collected over three different time-points (Spring, Summer, and Fall) in the growing season. Additionally, pollen and catkins were harvested from a mature field grown Clone 639 in Summer. The three time points chosen represent stages of Spring flush, mid-Summer growth/development, and those going into Fall senescence/dormancy, respectively. While determining the overall plant phenol biochemical pathways operative in red alder’s disparate tissue types, we also sought to potentially deduce candidates for the long unknown gateway entry points to the beneficial bioactive proanthocyanidin and ellagitannin metabolic classes, as well as in better understanding the biochemical basis of diarylheptanoid formation. Our findings herein thus address the biochemical basis underlying its ecological roles (including plant defenses), its varied medicinal uses, and its extensive dye usage.

## 2 Materials and methods

### 2.1 Total RNA extraction

Three biological replicates of buds, leaves, stems, roots (non-nodule containing segment adjacent to root nodules) and nodules from 3 year-old Clone 639 red alder saplings, that were greenhouse grown at Pacific Northwest National Laboratory (PNNL), were harvested at 3 time-points; early April (Spring), mid-June (Summer), and late October (Fall). Pollen and catkins were obtained from mature field-grown Clone 639 in Summer. Tissues were individually flash frozen immediately after harvest in liquid nitrogen and ground into fine frozen powders using a Freezer/Mill (SPEX, Metuchen, NJ) with a program that contained 2 cycles, with a 1 min pre-cool, a 2 min run time at 10 cycles/min and a 1 min cooldown period. Total RNA was extracted from each tissue type using the Spectrum™ Plant Total RNA kit (Sigma Aldrich) according to the manufacturer’s suggested protocol. Extracted RNA samples were stored at –80°C until ready for use.

### 2.2 RNA quality, library preparation and sequencing

Total RNA integrity was individually assessed using Fragment Analyzer (Advanced Analytical Technologies, Ankeny, IA) with the High Sensitivity RNA Analysis Kit. RNA Quality Numbers (RQNs) from 1 to 10 were assigned to each sample to indicate its integrity or quality. “10” stands for a RNA sample without any degradation, whereas “1” marks a completely degraded sample. RNA samples with RQNs ranging from 8 to 10 were only used for RNA library preparations with the TruSeq Stranded total RNA Library Prep Kit with Ribo-Zero rRNA Removal Kit (Illumina, San Diego, CA). Sizes of RNA libraries were assessed by Fragment Analyzer with the High Sensitivity NGS Fragment Analysis Kit, and their concentrations were measured by StepOnePlus Real-Time PCR System (ThermoFisher Scientific, San Jose, CA) with the KAPA Library Quantification Kit (Kapabiosystems, Wilmington, MA). Libraries were diluted to 2 nM with Tris-HCl buffer (10 mM, pH 8.5) and denatured with 0.1 N NaOH. Eighteen pM libraries were clustered in a high-output flow cell using HiSeq Cluster Kit v4 on a cBot (Illumina). After cluster generation, the flow cell was loaded onto HiSeq 2500 for sequencing using HiSeq SBS kit v4 (Illumina). DNA was sequenced from both ends (paired end) with a read length of 100 bp at the WSU Spokane Genomics Service Center. The raw bcl files were converted to fastq files using software program bcl2fastq2.17.1.14. Adaptors were trimmed from the fastq files during the conversion.

### 2.3 Transcriptome assembly

For each sample, RNA-seq reads were assembled into putative transcripts using the Trinity pipeline (Grabherr et al., 2011). Read coverage was normalized using bbnorm (https://sourceforge.net/projects/bbmap/), with normalized paired end reads then aligned to the red alder reference genome assembly using the default parameters of HISAT2 (Kim et al., 2019). The resulting SAM file was converted to BAM format and sorted, then the individual transcriptomes were assembled with Trinity (Grabherr et al., 2011) using the reference assembly (Hixson et al., 2023) as a guide, with parameters: –max_memory 50G –no_normalize_reads –genome_guided_bam –genome_guided_max_intron 10000. Assembly statistics were extracted with the TrinityStats.pl script provided as part of the Trinity package.

### 2.4 Bioinformatics analyses

The bioinformatics component of this study built upon the red alder Clone 639 sequencing and assembly (Hixson et al., 2023). This study herein focused upon genes involved (or potentially involved) in generation of the red alder plant defense, plant dye, and medicinal natural products, i.e. shikimate-chorismate-phenylalanine-*β*-glucogallin, and phenylpropanoid/phenylpropanoid-acetate pathway derived metabolites, including diarylheptanoids, ellagitannins (hydrolysable tannins), proanthocyanidins (condensed tannins), and other plant phenols. Sequences of previously characterized proteins/enzymes were retrieved from NCBI and used as queries to search the Clone 639 predicted proteins using BLASTP.

Protein sequences for dehydroquinate dehydratase/shikimate dehydrogenase (DHQD-SDH), UDP glucosyltransferase (UGT), arogenate dehydratase (ADT), cinnamate 4-hydroxylase (C4H), *p-*coumarate 3-hydroxylase (C3H), ferulate 5-hydroxylase (F5H), flavonoid 3′-hydroxylase (F3′H), flavonoid 3′,5′-hydroxylase (F3′,5′H) and flavone synthase II (FNSII) homologs were also retrieved from databases of seven Fagales species: Black alder (*Alnus glutinosa*), silver birch (*Betula pendula*), grey oak (*Casuarina glauca*), European hazel (*Corylus avellana*), European beech (*Fagus sylvatica*), American black walnut (*Juglans nigra*) and pedunculate oak (*Quercus robur*) (see **Supplementary Table S1** for database source).

Unrooted phylogenetic trees were generated using Clustal Omega (Madeira et al., 2024) and rendered using iTol (Letunic and Bork, 2021).

### 2.5 Gene model curation

Gene models of red alder Clone 639 genes of interest were curated in the Apollo annotation system (Dunn et al., 2019), assisted by RNA-seq data, to correct errors and determine the most likely gene models. GFF files of the Apollo gene models were exported and Transcripts Per Million (TPM) were calculated using TPMCalculator (Vera Alvarez et al., 2019). TPM values were plotted using the GD::Graph perl module to arrive at an expression atlas for each gene.

### 2.6 Metabolite extraction

Aliquots of each of the same cryogenically pulverized samples (each done in triplicate) harvested for RNA-seq analysis from roots, root nodules, stems, leaves, buds, pollen and catkins were individually used for metabolomics analyses. For metabolite extraction, each previously pulverized sample (as described above) was initially freeze-dried. Each freeze-dried sample (5 – 10 mg) was then extracted with MeOH–H_2_O (70:30, v/v) containing 0.1 mM naringenin (Aldrich) internal standard (IS) at a ratio of 1 mL to 50 mg tissue. After adding extraction solvent, each solution was vortexed for 30 s, sonicated in cold H_2_O for 15 min, vortexed again and centrifuged (20,000 *g* × 15 min, at 4°C). Each supernatant was transferred individually to vials and subjected to ultra-high performance liquid chromatography – quadrupole time-of-flight – mass spectrometry (UPLC-QToF-MS) analyses.

### 2.7 UPLC-QToF-MS analyses and metabolomics data processing

This employed an ACQUITY™ UPLC system (Waters, Milford, MA, USA) equipped with a photodiode array (PDA) eλ detector (Waters) coupled to a Xevo™ G2 QToF mass spectrometer (Waters MS Technologies, Manchester, UK) using MassLynx (V4.1) software. Separations of metabolites used an BEH C_18_ column (Waters, 2.1 × 150 mm, 1.7 *µ*m particle size), with a linear gradient for separation: 100% A (0.1% HCO_2_H in H_2_O) over 0.5 min, then sequentially to 45% B (0.1% HCO_2_H in CH_3_CN) over 25.0 min, to 100% B over 1.0 min, to 100% A in 2 min and held at 100% A for an additional 11.5 min. Flow rate was 0.2 mL min^–1^, with column and sample temperatures kept at 25 and 10°C, respectively. Injection volumes were 2 *μ*L for LC–MS analysis, with UV-visible spectra recorded between 200 and 500 nm (1.2 nm resolution). An electrospray ionization (ESI) source was used to detect masses of eluted compounds (*m/z* range: 100 – 1000 Da) and Ar was the collision gas. Detection settings were as follows: Negative ion mode (capillary voltage at 2.0 kV; cone voltage at 30 eV; collision energy at 6 eV and at 27 eV) and positive ion mode (capillary voltage at 3.0 kV; cone voltage at 30 eV; collision energy at 6 eV and at 18 eV). Sodium formate (5 mM in 2-propanol-H_2_O, 90:10, v/v) was used for calibrating the mass spectrometer, with leucine enkephalin (2 ng *µ*L^–1^ in A:B, 50:50, v/v) employed as lock-mass. Metabolomics data processing was carried out as in Lu et al. (2017).

All metabolite peak information (i.e. retention time, UV spectrum, and MS fragmentation analysis) from LC–MS data was used for targeted and non-targeted metabolite annotation. For targeted metabolite identification and annotation of compounds in red alder tissue extracts, authentic standards were used to establish coincident retention times, comparable molecular ions, and corresponding MS fragmentation patterns. For some metabolites (i.e. *p*-hydroxycinnamoyl quinic acid isomers), the previously reported order of their elution and fragmentation patterns allowed for the basis of their identifications (Gutiérrez Ortiz et al., 2018). Identification of metabolic features were performed using RStudio (Smith et al., 2006) as in Lu et al. (2017). Metabolomics data (i.e. meta-table of red alder tissue extract UPLC/EIMS analyses) included 673 features with 69 identified and/or putatively annotated (see **Supplementary Data Set** and **Supplementary Table S2**), with the total ion chromatograms (TIC) of the various red alder Clone 639 tissues individually used to help identify metabolites. The 69 metabolites above belonged to 8 distinct plant metabolite classes including diarylheptanoids, ellagitannins and precursors, proanthocyanidins, flavonoids, organic acids, gallic acid derivatives, phenolic acids, and polyamines, these being either fully identified or provisionally annotated.

Authentic alder diarylheptanoid standards included metabolites, oregonin, rubranol, hirsutanonol, rubranoside A, rubranoside B, alnuside A, alnuside B, platyphylloside, and 2′′′-*O*-*p*-coumaroyloregonin, 5-hydroxy-1,7-bis(4-hydroxyphenyl)heptan-3yl-*β*-D-apiofuranosyl-(1→6)-*β*-D-glucopyranoside, 1,7-bis-(4-hydroxyphenyl)heptan-3yl-*β*-D-apiofuranosyl-(1→6)-*β*-D-glucopyranoside, 7-(3,4-dihydroxyphenyl)-1-(4-hydroxyphenyl)-3(*R*)-*β*-D-glucosyloxy-heptane, and 5-*O*-methylhirsutanonol (Lai et al., 2012; Novaković et al., 2013), with each of these being used to analyze MS fragmentation patterns of all diarylheptanoids present in red alder Clone 639. Characteristic ion fragments of diarylheptanoids (*m*/*z* values of 327.12, 331.16, 313.14 311.13, 295.13, 205.09, and 189.09) were also used to obtain extracted ion content (EIC) from red alder tissues for additional diarylheptanoid identification/tentative annotation.

Other authentic standards used for comparison purposes included various flavonoids, organic acids, phenolic acids, spermidine derivatives, ellagitannins and precursors thereof, and proanthocyanidins:

Flavonoids: Apigenin, (+)-catechin, (–)-epicatechin, 7-*O*-methylapigenin (genkwanin), isoquercitrin, quercitrin, kaempferol-3-*O*-glucoside, and luteolin.
Organic acids: Citric, quinic, and shikimic acids.
Phenolic acids: *trans*-chlorogenic acid, cryptochlorogenic acid, *cis*- and *trans*- *p*-coumaric acid *O*-glucoside, and 3-*O*-feruloyl quinic acid.
Spermidine derivative: *N^1^,N^10^*-bis(*p*-coumaroyl)spermidine.
Ellagitannins and precursors: Casuarinin, pedunculagin (*α* and *β* anomers), strictinin, tellimagrandin I (*α* and *β* anomers), tellimagrandin II, *β*-glucogallin, and pentagalloyl glucose.
Proanthocyanidins: Procyanidins B1, B2, and B3.

Calculated and experimentally determined masses, together with ppm differences of each feature were used to assess annotation accuracy (see ppm error values listed in **Supplementary Table S2**). Relative abundances (normalized to internal standard naringenin equivalents) of annotated and tentatively annotated features from metabolic classes are shown using Excel. The features were classified as to occurrence(s) in nodules, roots, stems, buds, leaves, pollen, and catkins, respectively, with normalized data as obtained above.

## 3 Results and discussion

The research strategy builds upon our earlier red alder genome sequencing (Hixson et al., 2023), with the investigation herein integrating RNA-seq gene expression and metabolomics data analyses of different tissues at various seasonal time-points. The time-points of early Spring, mid-Summer, and Fall allowed for comparison of metabolite levels and pathway transcript abundances during distinct stages of the growing season from breaking out of dormancy to going back into dormancy.

The analyses were done and interpreted as described below on the major operative plant phenol biochemical pathways. In so doing, particular emphasis was also placed towards deducing potential gateway entry points to key plant biochemical pathways of interest (i.e. those involved in plant defense, medicinal uses, and dye formation and its Native American uses).

Analyses were carried out on stems, buds, leaves, roots (non-nodule containing segment adjacent to root nodules) and root nodules using 3-year-old greenhouse-grown red alder Clone 639. Pollen and catkins were also collected from its mature field-grown trees. Biological replicates (3) were harvested from greenhouse-grown plants including in Spring (April), Summer (mid-June), and Fall (late October), in Southeastern Washington State, and Summer (mid-June) for catkin and pollen samples from Northwestern Washington field trial sites.

### 3.1 Shikimate-chorismate-phenylalanine, quinate, and gallic acid related pathways: correlating transcript and metabolite abundances

**Supplementary Figure S1** depicts a simplified biochemical shikimate-chorismate pathway to phenylalanine (Phe) via shikimate, as well as to quinic acid and *β*-glucogallin via pathway offshoots from 3-dehydroquinate. The main carbon flux overall to these different metabolites is well known to be directed towards the phenylpropanoid pathway as this primarily results in structural lignin deposition (Nature’s second most abundant terrestrial vascular plant biopolymers), as well as to various lower molecular weight phenylpropanoids (particularly diarylheptanoids in red alder) and related mixed biochemical pathway intermediates. Also metabolically important are the quinic acid and *β*-glucogallin metabolic pathway branches, albeit representing relatively minor carbon flux offshoots to various quinate derivatives and to gallo- and ellagitannins, respectively.

These different metabolic branches, however, all utilize the 3-dehydroquinate intermediate, with the latter being differentially converted into shikimic, quinic, and gallic acids, respectively (**Supplementary Figure S1**). As described below, while only some of the overall possible branch point pathway intermediates are detected in the metabolomics analyses of different plant tissues at different time-points, it was considered instructive nevertheless to compare/contrast these trends with transcript abundances at the different time points. [Note: while these analyses were taken at three distinct time-points in the growing season or were harvested (catkins and pollen) in the summer for mature field grown Clone 639, such analyses give no indication of either any potential metabolic turnover or transcriptional abundance changes and/or protein turnover].

**Figure 2:**
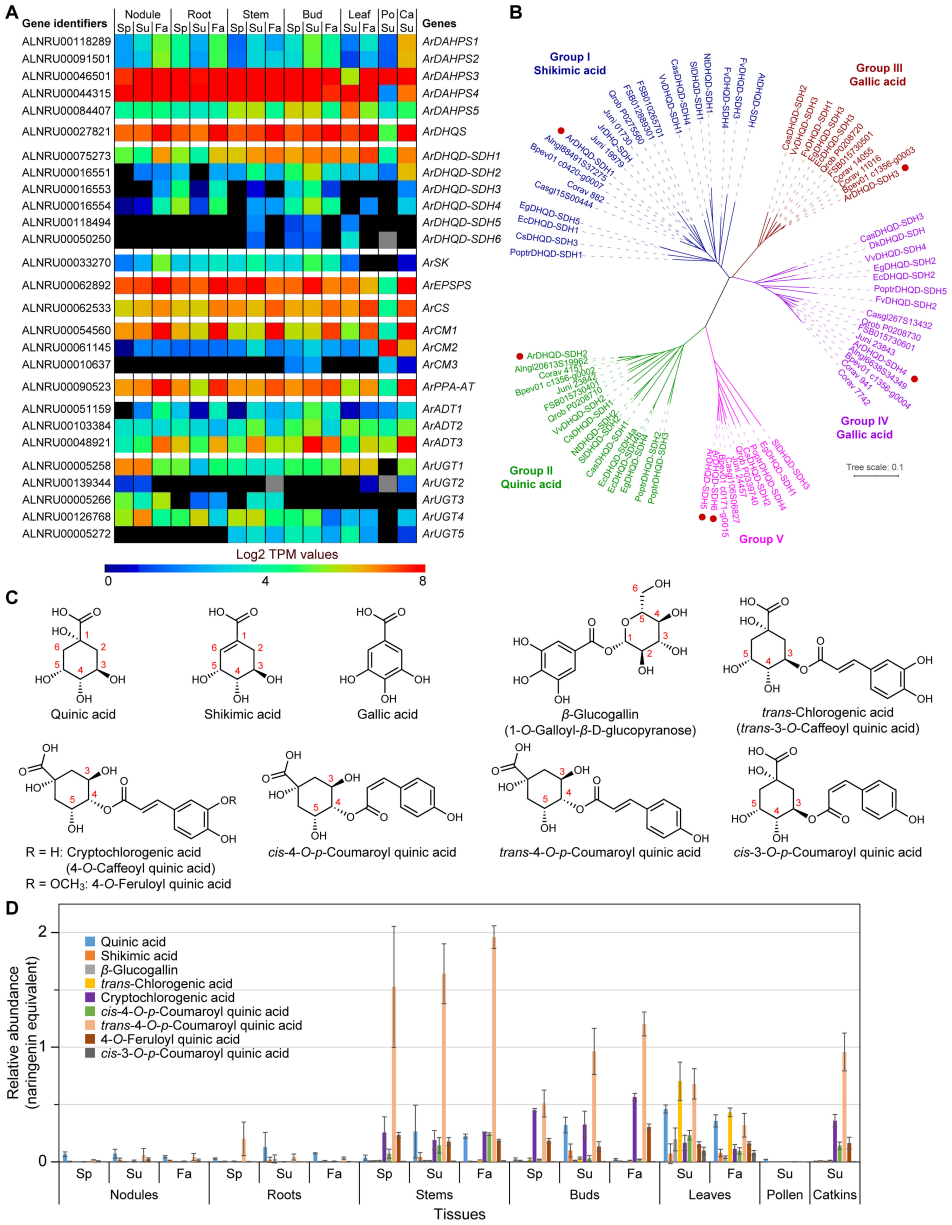
Shikimate-chorismate pathway and related metabolism. **(A)** Heatmap showing average Log2 TPM (transcripts per million) values obtained from transcriptomics analysis for the putative shikimate-chorismate-phenylalanine-*β*-glucogallin pathway genes. *Grey* shading in the heatmap indicates that there were no reads detected. **(B)** Unrooted phylogenetic tree of 3-dehydroquinate dehydratase/shikimate dehydrogenase (DHQD-SDH) families. The sub-family nomenclature of Bontpart et al. (2016) is used. Group I-V individual protein database accession numbers and species abbreviations are in **Supplementary Figure S4**. Red dots indicate red alder genes. **(C)** Shikimic, quinic, gallic, chlorogenic, cryptochlorogenic, 4-*O*-feruloyl quinic, *trans/cis* 4-*O*-*p*-coumaroyl quinic and *cis*-3-*O-p*-coumaroyl quinic acids, and *β*-glucogallin. Numbering system for quinic acid derivatives follows Alcázar Magaña et al. (2021). **(D)** Relative abundances of various shikimate-quinate-gallate-phenylpropanoid pathway derived metabolites found in red alder in naringenin equivalents. Ca, catkins; Fa, fall; Po, pollen; Sp, spring; Su, summer.

A BLAST search of our red alder predicted proteins using previously known *bona fide* shikimate-chorismate-phenylalanine and related pathway genes from *Arabidopsis* and other species gave 27 putative genes (**Supplementary Table S3**; **Figure 2A**); these are shown placed in the order of their biochemical pathway transformations.

Their corresponding 27 proteins had plastid targeted signal peptides as determined using DeepLoc 2.0 (Thumuluri et al., 2022) and LOCALIZER 1.0.4 (Sperschneider et al., 2017), with the exceptions of the presumed cytosolic bifunctional 3-dehydroquinate dehydratase/shikimate dehydrogenase (DHQD-SDH) sub-family, a putative chorismate mutase (ArCM2), and the putative *β-*glucogallin-forming UDP glucosyltransferases (UGTs) (**Supplementary Table S3**). The existence of a cytosolic chorismate mutase (CM2) has long been known (d’Amato et al., 1984), with its gene widely conserved across plant species (Westfall et al., 2014). However, its physiological function was until recently unknown. In petunia (*Petunia hybrida*), Qian et al. (2019) reported that its cytosolic PhCM2 was able to form prephenate (from chorismate), which can next be converted first to phenylpyruvate by phenylpyruvate dehydratase (PDT), and then to Phe by action of a cytosolic phenylpyruvate aminotransferase (PPY-AT) (discussed later).

Amino acid sequence alignments for each enzymatic step leading to Phe, relative to established functional proteins in other plant species, are in **Supplementary Figures S2**–**S11**. Provisional red alder amino acid identity prioritization was based on comparison to known *bona fide* proteins (**Supplementary Table S3**). Some of the putative red alder proteins though had relatively low identities (*ca* 50% or lower) with respect to corresponding known homologs from *Solanum lycopersicum*, *Corydalis sempervirens* and *Arabidopsis*, i.e. including members of the ArDHQD-SDH, shikimate kinase (ArSK), ArCM2, and arogenate dehydratase (ArADT2 and ArADT3) families.

In red alder, dehydroquinate synthase (*ArDHQS*), *ArSK*, 5-enolpyruvylshikimate-3-phosphate synthase (*ArEPSPS*), chorismate synthase (*ArCS*) and prephenate aminotransferase (*ArPPA-AT*) are apparently single gene families. The others are encoded by small (3 – 6) multi-gene families, i.e. 3-deoxy-D-arabinoheptulosonate 7-phosphate synthase (*ArDAHPS*, 5 sub-family genes), *ArDHQD-SDH* (6), *ArCM* (3) and *ArADT* (3), respectively.

Relative transcript abundances for genes putatively encoding each enzymatic step in each tissue at different time-points are in **Figure 2A** and **Supplementary Figure S12**. Highest expression levels were noted across essentially all tissues and time-points for *ArDAHPS3* and *ArDAHPS4* (highest expression levels overall), *ArDHQS*, *ArDHQD-SDH1, ArEPSPS*, *ArCS*, *ArCM1, ArPPA-AT* and, to a lesser extent, *ArADT3*, respectively. These specific transcript abundances however presumably represent genes encoding each of the actual biochemical steps involved in the predominant phenylpropanoid carbon flux to lignin, this being evident for nearly all tissues and time-points, with the exception of the lower overall levels in pollen. Conversely, *ArSK* transcript levels were notably different (low throughout) for all tissue types and time-points.

Interestingly, about half of the 27 potential shikimate-chorismate pathway genes are reasonably strongly expressed in the 7 distinct tissue types and time-points and are thus likely to encode the main sub-family isoforms involved overall.

As regards red alder’s other ArDAHPS isoforms, it was noted that both ArDAHPS1 and ArDAHPS2 marginally had the highest amino acid identity to *bona fide* DAHPS from *Arabidopsis*. Yet, their encoding genes only had moderate to low expression levels in catkins, these being even lower in all other tissues (**Supplementary Figure S12A**). Furthermore, *ArDAHPS5* had low gene expression across all tissue types (highest in bud, leaf, and stem tissues), the significance of which is currently unknown.

Of the 5 single gene family members, four (*ArDHQS*, *ArEPSPS*, *ArCS*, and *ArPPA-AT*) were expressed in all tissues at different time-points, with several being highest in Fall collected tissue (**Supplementary Figure S12B**). Somewhat comparable expression patterns for these 4 single gene family members were also noted for catkins, but not for pollen. Conversely, *ArSK* was the only single gene family member that consistently displayed low expression levels, i.e. weakly in nodules, roots, stems, buds, while barely detectable in leaf, catkin and pollen tissues.

Shikimate is formed from 3-dehydroquinate via 3-dehydroshikimate. In bacteria and fungi, these conversions are catalyzed by two monofunctional enzymes, dehydroquinate dehydratase (DHQD) and shikimate dehydrogenase (SDH), whereas in plants a bifunctional enzyme, DHQD-SDH, is used (**Supplementary Figure S1**).

Interestingly, *A. thaliana* has only one *DHQD-SDH* (AT3G06350, *AtDHQD-SDH*), whereas other plants have multiple gene copies such as red alder here. The AtDHQD-SDH reportedly shows a strict requirement for shikimate as substrate [*in vitro* assays were carried in the reverse direction, as no activity could be measured with quinate as substrate even at very high concentrations (Gritsunov et al., 2018)]. *DHQD-SDH* genes, whose corresponding proteins had quinate activity, have also been characterized in *Populus trichocarpa* (PoptrDHQD-SDH2 and 3) (Guo et al., 2014), whereas DHQD-SDHs with the capacity of producing gallic acid were identified in walnut (*Juglans regia*) and *Vitis vinifera* (Muir et al., 2011; Bontpart et al., 2016).

Phylogenetic analysis of DHQD-SDHs from different species within the dicotyledons showed that they cluster into five groups (I–V) (Bontpart et al., 2016). We thus constructed an unrooted phylogenetic tree using the sequences from Bontpart et al. (2016), the six red alder proteins (ArDHQD-SDH1 – ArDHQD-SDH6), as well as DHQD-SDHs from other Fagales genomes available [*A. glutinosa, B. pendula, C. glauca, C. avellana, F. sylvatica, J. nigra*, and *Q. robur* (**Supplementary Table S1**; **Figure 2B**)]. The 6-red alder *DHQD-SDHs* encompass all five sub-families, as do all Fagales DHQD-SDHs.

The bifunctional ArDHQD-SDH1 in Group I presumably converts 3-dehydroquinate into shikimate, with this envisaged to occur via an initial dehydration catalyzed by the DHQD domain to afford 3-dehydroshikimate, followed by a NADPH dependent reaction employing the SDH domain to afford shikimate. By contrast, ArDHQD-SDH2 in Group II can be envisaged to catalyze the NADH dependent conversion of 3-dehydroquinate into quinate. The Group III ArDHQD-SDH3 and Group IV ArDHQD-SDH4 likely produce gallic acid via initial formation of 3-dehydroshikimate (via the DHQD domain as above) followed by NADP^+^-dependent dehydrogenation using the SDH domain to produce gallic acid.

Group V homologs (ArDHQD-SDH5 and ArDHQD-SDH6) are, however, presently of unknown biochemical function.

Subsequent structural, bioinformatics, and biochemical approaches identified a primary sequence motif (^379^SX[TG]^381^; AtDHQD-SDH numbering) that could be used to predict substrate specificity between shikimate- and quinate-forming DHQD-SDHs (Gritsunov et al., 2018). That is, the *Arabidopsis* AtDHQD-SDH wild-type (WT) protein, harboring a Thr amino acid residue at position 381, only uses shikimate. However, when this residue was mutated to Gly, the mutant protein was able to oxidize quinate to 3-dehydroquinate (in the reverse reaction) (Gritsunov et al., 2018) and all known DHQD-SDHs in Group II have a Gly residue at this relative position (**Supplementary Figure S4**), i.e. perhaps suggesting that ArDHQD-SDH2 (and its Fagales homologs) produce quinate from 3-dehydroquinate. Interestingly, all group II DHQD-SDHs have a conserved Ser-Val-Gly (SVG) motif, as do Group V enzymes (although the latter’s biochemical function is as yet unknown). By contrast, Group III enzymes have a Ser-Cys-Thr (SCT) motif at these positions, whereas Group IV has a corresponding Ser-Tyr-Thr (SYT) motif, both of these being considered to be involved in gallate formation.

A cofactor key binding residue variation was also observed in all Group II sequences, e.g. the aspartate-isoleucine-aspartate (^483^DID^485^) motif is usually associated with NAD^+^ binding, in place of asparagine-arginine-threonine (^483^NRT^485^) which uses NADP^+^ in most other DHQD-SDHs (including in *A. thaliana*).

Relative *ArDHQD-SDH* sub-family gene expression (heatmap) and transcript abundances are in **Figure 2A** and **Supplementary Figure S12C**. As indicated above, the most highly expressed overall was the Group I *ArDHQD-SDH1*, particularly in Fall collected aerial tissues, versus a lower extent in root and nodules tissues (**Figure 2A**; **Supplementary Figure S12C**), i.e. in accordance with its presumed role in producing shikimic acid for phenylpropanoid/phenylpropanoid-acetate pathways. The putative quinate Group II forming *ArDHQD-SDH2* was second most highly expressed during Summer in stems, buds, and catkins, as well as to a much lower extent in nodules, roots, leaves, and pollen. By contrast, the presumed gallic acid-forming Group III *ArDHQD-SDH3* and Group IV *ArDHQD-SDH4* genes were only mildly expressed in root and bud tissues and, to an even lesser extent, in all other tissue types, except for pollen where they were not detected (discussed later below). The Group V *ArDHQD-SDH5* and *ArDHQD-SDH6*, of unknown biochemical functions, had faint expression levels in stem, bud, and leaf tissues collected in Summer. Their expression patterns though may give some clues as to their functions, i.e. in terms of seeking correlations in the future with unique stem, bud, and leaf metabolites perhaps only found in these tissues.

*ArCM* (3 genes) and *ArADT* (3 genes) expression was also examined (**Figure 2A**; **Supplementary Figures S12D, E**). Of the 3 *ArCM* genes, *ArCM1* was most highly expressed, particularly in Fall collected tissues and catkins, except for pollen (**Figure 2A**; **Supplementary Figure S12D**). *ArCM2* was also relatively highly expressed, but only in pollen and catkin tissues. By contrast, *ArCM3* was barely – if at all – detectable in bud and pollen tissues.

As regards the 3 red alder *ADT*s, *ArADT3* was generally the most highly expressed followed by *ArADT2* and *ArADT1* (**Figure 2A**; **Supplementary Figure S12E**). To better understand their possible physiological roles, we constructed an unrooted phylogenetic tree using ADT sequences from gymnosperms and angiosperms including the 7 Fagales species mentioned above (**Supplementary Figure S13**). ArADT2 clusters in the group with AtADT2 in ADT subgroup II (Cho et al., 2007), this enzyme being considered involved in protein synthesis in vascular plants, as knockouts of the gene were lethal (Corea et al., 2012). By contrast, ArADT3 is in the same cluster as AtADT3, AtADT4, AtADT5 and AtADT6 in subgroup III. Of the latter, double knockouts of *AtADT4* and *AtADT5* gave substantial reductions in lignin levels and related metabolites in *Arabidopsis*, with the corresponding quadruple knockout (*adt3/4/5/6*) having even larger effects (Corea et al., 2012; Höhner et al., 2018). ArADT1 (in subgroup I) which was expressed to the lowest level has not yet been directly correlated with a downstream metabolic process.

Interestingly, in petunia, an alternative transcription start site in *PhADT3* results in a cytosolic PhADT3 which can use prephenate generated by the cytosolic PhCM2 (as indicated earlier) to form phenylpyruvate. The latter can then be converted to small amounts of Phe through action of a cytosolic phenylpyruvate aminotransferase (PPY-AT) (Qian et al., 2019).

Best potential candidate genes for gallic acid metabolism to *β-*glucogallin were *ArUGT1* and *ArUGT2*, based on sequence comparison with *bona fide β-*glucogallin-forming UDP glucosyltransferases (UGTs) in other plant species (Mittasch et al., 2014; Cui et al., 2016; Ono et al., 2016). *ArUGT1* was relatively mildly expressed in all tissue types (except for pollen), whereas *ArUGT2* was essentially not detected, except for a very faint level in catkins and nodules (Spring/Summer) (**Figure 2A**; **Supplementary Figure S12F**). Neighbor-Joining analysis also showed that both ArUGT1 and ArUGT2 cluster within the phylogenetic group L of UGTs (Ross et al., 2001), together with ester-forming glucosyltransferases and more particularly with all characterized *β-*glucogallin-forming UGTs (**Supplementary Figure S14**). The Fagales homologs also cluster within this group. (Other red alder genes in group L, ArUGT3, ArUGT4 and ArUGT5, are discussed later below).

It was next instructive to ascertain which metabolites in the biochemical pathways leading to shikimic, quinic, and gallic acids were detectable in the seven tissue types, i.e. in an attempt to correlate gene expression with their metabolite occurrence in the different tissues. As previously indicated, some 673 features were detected of which 69 distinct metabolites were identified and/or putatively annotated. Numerous metabolites were identified based on either comparison with authentic standards, or via analyses of elution profile behavior and mass spectrometric fragmentation profiles relative to literature reports (discussed later). Provisional annotations do not though represent full unambiguous identification. Peak intensities in all chromatograms were normalized to the internal standard naringenin.

In this study, only quinic, shikimic, and gallic (as its *β*-glucogallin derivative) acids (**Figure 2C**) were unambiguously detected in all 7 tissue types to varying amounts (**Figure 2D**). Quinate was present to the largest amount in leaves, and also detected in all seven tissue types.

Mixed metabolic pathway derivatives were also detected with quinate esters being the most abundant.

In terms of correlations with transcript levels, shikimate (derived from ArDHQD-SDH1) and quinate (derived from ArDHQD-SDH2) metabolite levels were generally low in roots, nodules, and pollen (**Figures 2A, D**). Transcript level profiles for *ArDHQD-SDH1* and *ArDHQD-SDH2* showed comparable profiles. By contrast, *β*-glucogallin was at low levels in essentially all tissues examined (discussed later).

Both metabolomics and transcriptome data were thus in excellent agreement as regards each tissue type gene expression and the corresponding shikimic/quinic/gallic acid derived pathway constituents present.

### 3.2 Phenylpropanoid related and lignin forming pathways: correlating transcript and metabolite abundances

**Figure 3:**
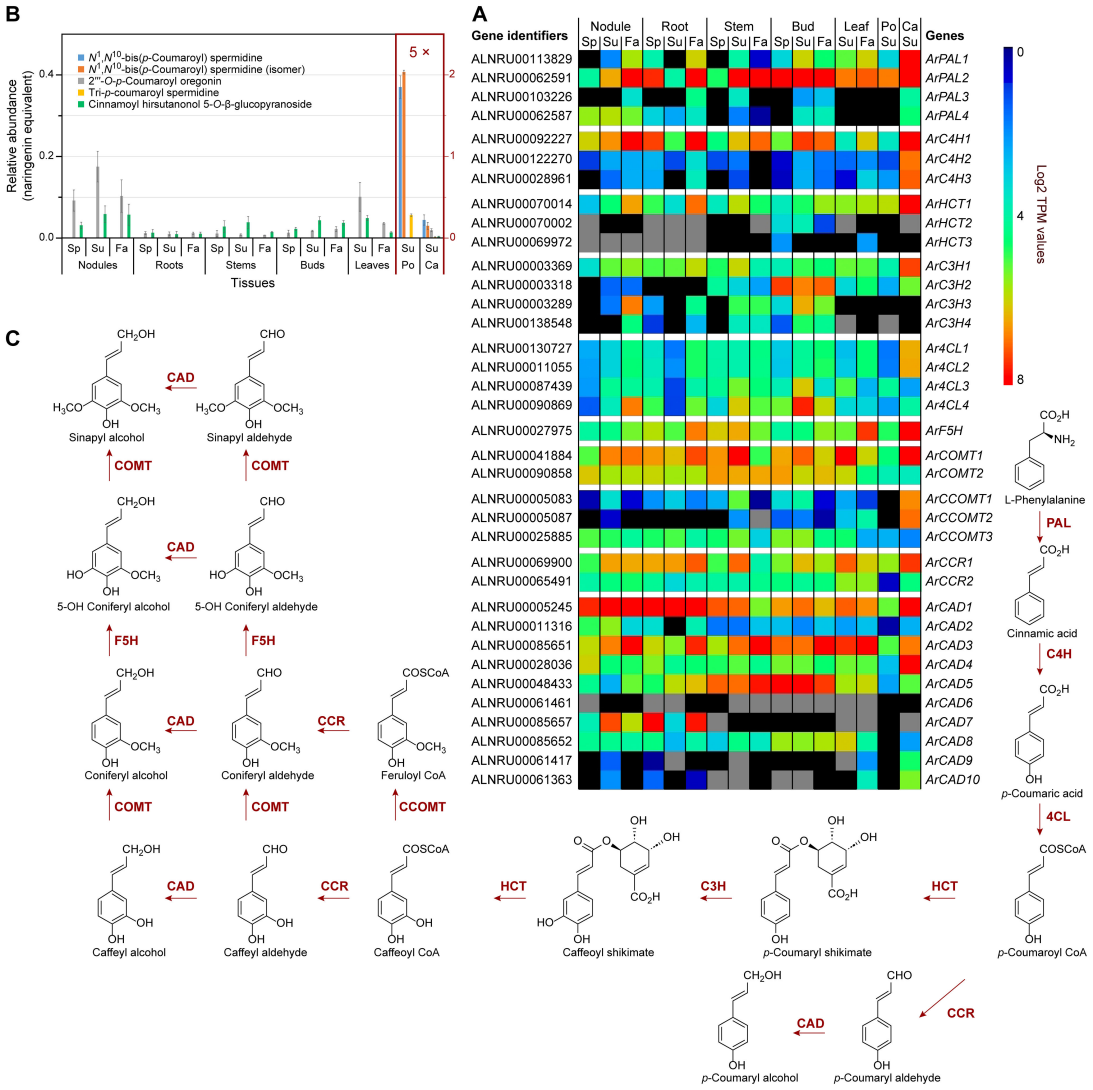
Phenylpropanoid pathway and related metabolites. **(A)** Heatmap showing average Log2 TPM (transcripts per million) values obtained from transcriptomics analysis for putative phenylpropanoid pathway genes. *Grey* shading indicates that there were no reads detected. **(B)** Relative phenylpropanoid metabolite abundances in naringenin equivalents. **(C)** Simplified phenylpropanoid pathway to monolignols. Ca, catkins; Fa, fall; Po, pollen; Sp, spring; Su, summer. See **Supplementary Table S4** for gene abbreviations.

**Figure 4:**
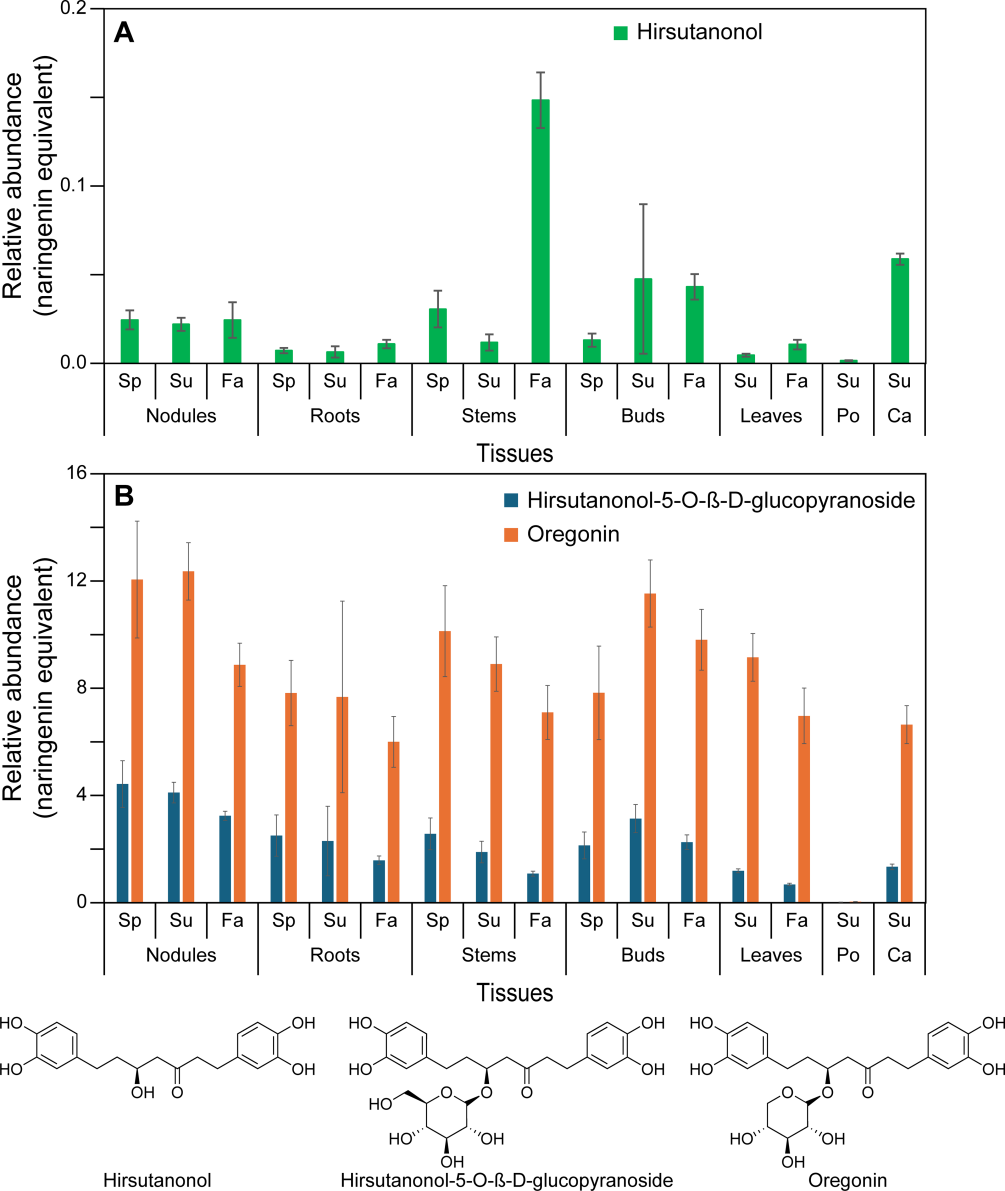
Relative abundance of diarylheptanoids in naringenin equivalents. **(A)** Hirsutanonol. **(B)** Hirsutanonol 5-*O-β*-glucopyranoside and oregonin. Ca, catkins; Fa, fall; Po, pollen; Sp, spring; Su, summer.

As indicated above, phenylpropanoids, largely derived from Phe, are mainly ultimately converted into abundant cell wall lignin biopolymers, as well as to various low molecular weight compounds (**Supplementary Figure S15**). In red alder, the latter include phenylpropanoid-derived esters/glucosides (**Figure 2D**), amides (**Figure 3B**), and diarylheptanoids (**Figure 4**; **Supplementary Figure S16**), with the diarylheptanoids vastly dominating in relative amounts.

A BLAST search of the red alder predicted proteins yielded 36 putative genes encoding all phenylpropanoid pathway transformations from Phe to lignin/lignan precursor monolignols, *p*-coumaroyl, coniferyl and sinapyl alcohols (**Figure 3C**; **Supplementary Figures S17**–**S26**; **Supplementary Table S4**), with gene families ranging from 1 – 10 members. (The first three enzymatic steps in the phenylpropanoid pathway, namely phenylalanine ammonia lyase (PAL), cinnamate 4-hydroxylase (C4H), and 4-coumarate CoA ligase (4CL) are also shared with the flavonoid (phenylpropanoid-acetate) pathway as described later).

Red alder homologs were generally ranked in decreasing sequence identity to *bona fide* homologs in other plant species, with these mainly prioritized again in terms of highest sequence identity, i.e. *ca* 70% or higher to those of known function. However, some red alder phenylpropanoid pathway multi-gene family members had low homologies to *bona fide* enzymatic steps, i.e. those annotated as ArC4H2 and ArC4H3 (*ca* 62% identity), Ar4CL4 (60.3% identity), caffeic acid *O*-methyltransferase (ArCOMT2, 62.3% identity), caffeoyl CoA *O*-methyltransferase (ArCOMT3, 58% identity), cinnamoyl CoA reductase (ArCCR2, 50.3% identity), and 8 cinnamyl alcohol dehydrogenases (ArCAD3 to ArCAD10, with 50.1 – 47.5% identities), respectively. In some of these annotated proteins, such low amino acid level identities may reflect different catalytic functions as regards substrate(s).

An unrooted phylogenetic tree was constructed that included sequences of characterized hydroxylases in the phenylpropanoid pathway (i.e. C4H, *p*-coumarate 3-hydroxylase, C3H, and ferulate-5 hydroxylase, F5H families), these belonging to the CYP73A, CYP98A, and CYP84A families, respectively, including those from the seven Fagales species mentioned above (**Supplementary Figure S27**). ArC4H1 clustered with CYP73A family members *Helianthus tuberosus* HtC4H (Teutsch et al., 1993), *Arabidopsis* AtC4H (Mizutani et al., 1997), and *Medicago sativa* MsC4H (Fahrendorf and Dixon, 1993), whereas ArC4H2 and ArC4H3 clustered with a French bean (*Phaseolus vulgaris*) PvC4H (Nedelkina et al., 1999) as a separate CYP73A subgroup. Additionally, ArF5H clustered with the CYP84A AtF5H (Meyer et al., 1996). Finally, the four alder C3Hs (ArC3H1 – 4) clustered with the CYP98A, AtC3H (Schoch et al., 2001), ObC3H from basil (*Ocimum basilicum*) (Gang et al., 2002) and CcC3H1/2 from coffee (*Coffea canephora*) (Mahesh et al., 2007).

Transcript abundances of the aforementioned 36 genes in different tissues and organs are provided in a cumulative (relative) heatmap (**Figure 3A**), and for each enzyme class individually in **Supplementary Figure S28**. Within specific gene families, the isoforms generally with highest expression levels (**Figure 3A**; **Supplementary Figures S28A–I**) in the seven tissue types included *ArPAL2*, *ArC4H1*, hydroxycinnamoyl CoA:shikimate hydroxycinnamoyl transferase *ArHCT1*, *ArC3H1/2*, *Ar4CL4*, *ArF5H, ArCOMT1/2*, cinnamoyl CoA reductase *ArCCR1*, as well as *ArCAD1, ArCAD3*, and *ArCAD5.* These mainly had high to highest sequence identities at the amino acid level relative to *bona fide* enzymatic steps in other plant species; however, *Ar4CL4, ArCAD3*, and *ArCAD5* had lower identity levels. Taken together, these expression levels are indicative of an intact pathway to the monolignols with the corresponding expected isoforms.

Catkin tissue also had moderate to relatively high expression of *ArPAL2/ArPAL1* (**Figure 3A**; **Supplementary Figure S28A**), *ArC4H1*/*ArC4H2*/*ArC4H3 and ArF5H* (**Figure 3A**; **Supplementary Figure S28B**), *ArHCT1* (**Figure 3A**; **Supplementary Figure S28C**), *ArC3H1*/*ArC3H2* (**Figure 3A**; **Supplementary Figure S28D**), *Ar4CL1*/*Ar4CL2* (**Figure 3A**; **Supplementary Figure S28E**), *ArCOMT1* (**Figure 3A**; **Supplementary Figure S28F**), *ArCCOMT1*/*ArCCOMT*2 (**Figure 3A**; **Supplementary Figure S28G**), *ArCCR1* (**Figure 3A**; **Supplementary Figure S28H**) and *ArCAD1*/*ArCAD3/ArCAD4* (**Figure 3A**; **Supplementary Figure S28I**).

*ArC3H2* also displayed notable expression levels in bud tissues, whereas *ArC3H3* and *ArC3H4* (**Supplementary Figure S28D**) had essentially low to no expression in leaf, pollen, and catkin tissues.

Of the CADs, *ArCAD1* was most highly expressed in nodules, roots, and catkins, and to a lesser extent in all other tissues (**Supplementary Figure S28I**), this being a homolog most closely related to phenylpropanoid/monolignol/lignin formation proper (Kim et al., 2004; Jourdes et al., 2007).

*ArCAD3*, however, was highly expressed in nodules, roots, stems, buds, and leaves, particularly in the Fall collected tissues, as well as in catkins, whereas *ArCAD5* had highest expression levels in lignifying stems and in buds. By contrast, *ArCAD7* expression was highest in both nodule and root tissues, the physiological significance of the latter being currently unknown given its low sequence identity.

Three additional *ArUGTs* (*ArUGT3*, *ArUGT4*, and *ArUGT5*) were present in the red alder genome, albeit of lower homology (~70%) to the afore-mentioned *β-*glucogallin-forming UGTs (**Figure 2A**; **Supplementary Table S3**). These, and other Fagales UGTs, clustered between the *β-*glucogallin-forming UGTs and hydroxycinnamic acid 1-*O*-glucosyltransferases from *Arabidopsis* (UGT84A1–UGT84A4) and *Brassica napus* (BnUGT84A9) (Milkowski et al., 2000a, b), perhaps suggesting a role in downstream plant phenol monomer glucosylation (**Supplementary Figure S14**). The putative *ArUGT3* and *ArUGT4* genes were generally moderately to weakly expressed in nodules, roots, and stems. *ArUGT4* was also expressed in buds, and, to a lesser extent, in leaves and catkins (**Supplementary Figure S12F**), whereas *ArUGT5* had lower levels of expression in stem, bud, leaf, pollen, and catkin tissues, but was not detected in nodules or roots. *ArUGT3 – ArUGT5* expression was not detected in pollen tissue.

Under the conditions employed, our metabolomics analysis did not detect all possible monomeric phenylpropanoid pathway products *per se*, but instead various derivatives thereof. This is because most of the carbon flux went directly into lignin biopolymer deposition (not shown) and diarylheptanoids (**Figures 4A, B**; **Supplementary Figures S16A–D**), with the latter discussed later. Much less abundant low molecular weight phenylpropanoid derivatives detected included the aforementioned mixed metabolic pathway hydroxycinnamoyl quinate ester derivatives, of which *trans*-4-*O*-*p*-coumaroyl quinic acid, cryptochlorogenic (4-*O*-caffeoyl quinic acid), and chlorogenic acid had highest (albeit variable) accumulation levels across stems, buds, leaves, and catkins and, to a lesser extent, in roots (**Figures 2C, D**; **Supplementary Table S2**). Pollen tissue, by contrast, lacked any of these esters under the conditions employed.

In addition, five other phenylpropanoid derivatives were found in the various tissues, with spermidine derivatives highest in pollen, i.e. tri-*p-*coumaroyl spermidine, *N^1^,N^10^*-bis(*p*-coumaroyl)spermidine (confirmed with authentic standard), and a *N^1^,N^10^*-bis(*p-*coumaroyl)spermidine like metabolite, as well as two phenylpropanoid diarylheptanoid esters (i.e. 2′′′-*O*-*p*-coumaroyl oregonin and cinnamoyl hirsutanol-5-*O*-*β*-glucopyranoside) in other tissues (**Figure 3B**; **Supplementary Table S2**).

Of these, the *N^1^,N^10^*-bis(*p-*coumaroyl)spermidine like metabolite differs from the authentic *N^1^,N^10^*-bis(*p-*coumaroyl)spermidine standard chromatographically. This could perhaps be due to either different *cis/trans p*-coumaroyl isomers or different *p*-coumaroyl positional isomers.

Hydroxycinnamic acid amide-linked spermidine metabolites had previously been reported in various *Alnus* species, and are reportedly specific to the Fagales in pollen exine (Meurer et al., 1988).

While the main metabolic flux is of course lignin and diarylheptanoid targeted, the genes that are significantly more highly expressed (*ArPAL1/2, ArC4H1, ArHCT1, ArC3H1/2, Ar4CL4, ArCOMT1/2, ArCCR1, and ArCAD1*; **Figure 3A**) can be considered as the main *bona fide* phenylpropanoid pathway genes controlling flux into lignins, diarylheptanoids (**Figure 4**; **Supplementary Figure S16**), phenylpropanoid esters (**Figure 2D**), and phenylpropanoid amides (**Figure 3B**).

Previously discussed expression levels (**Supplementary Figure S12C**) of *ArDHQD-SDH2* (encoding the envisaged quinic acid forming isoform), and *ArDHQD-SDH1* (for shikimate formation and leading mainly to lignin, hydroxycinnamic acid intermediates, and diarylheptanoids), were thus also in good agreement with metabolite occurrences (**Figures 2D**, **3B**, **4A, B**; **Supplementary Figure S16A–D**; **Supplementary Table S2**). *ArDHQD-SDH2* expression was highest in stem, bud, and catkin tissues reflecting the higher levels of hydroxycinnamic acid quinate esters present. *ArDHQD-SDH1* was more highly expressed in all tissue types, as to be anticipated for shikimate formation for hydroxycinnamic acid, diarylheptanoid, and lignin biosynthesis. Pollen, by contrast, only had traces of quinic acid and diarylheptanoids.

### 3.3 Phenylpropanoid-acetate flavonoid pathways: correlating transcript and metabolite abundances

A simplified flavonoid biochemical pathway, such as to catechin and epicatechin, is summarized in **Supplementary Figure S29**, and involves 11 additional specific enzymatic steps/enzyme classes beyond those already described above for PAL, C4H, and 4CL (**Supplementary Figure S15**).

**Figure 5:**
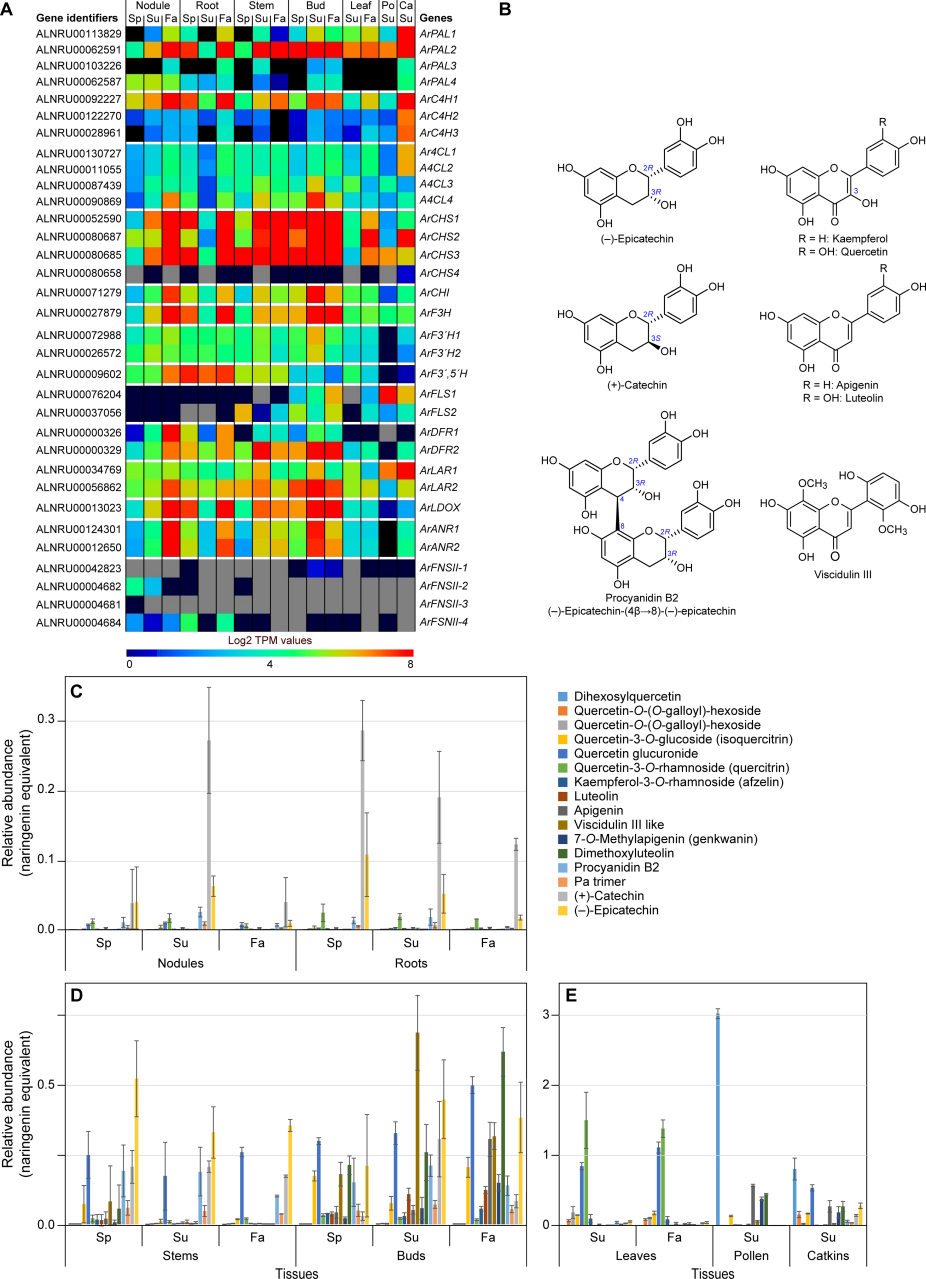
Flavonoid pathway metabolism. **(A)** Heatmap showing average Log2 TPM values obtained from transcriptomics analysis for the putative flavonoid pathway genes. *Grey* shading in the heatmap indicates that there were no reads detected. **(B)** Representative flavonoid and proanthocyanidin derivatives. **(C, D)** Differential levels of various flavonoids and proanthocyanidins (procyanidin B2 and a procyanidin trimer) in red alder roots and nodules **(C)**, stems and buds **(D)**, and leaves, pollen and catkins **(E)** in naringenin equivalents. Ca, catkins; Fa, fall; Po, pollen; Sp, spring; Su, summer.

Entry point substrates for flavonoid and PA formation are phenylpropanoid-derived *p*-coumaroyl CoA and acetate pathway derived malonyl CoA as shown (**Supplementary Figure S29**). Metabolomics analyses of the various red alder tissues resulted in detection of 17 different flavonoids and proanthocyanidins (**Supplementary Table S2**), of which apigenin, 7-*O*-methyl apigenin (genkwanin) [not shown], luteolin, isoquercitrin [not shown], quercitrin [not shown], (+)-catechin, (–)-epicatechin, and procyanidin B2 (**Figure 5B**) were unambiguously identified based on comparison with their authentic standards. Relative abundances of known and those of other provisionally annotated flavonoids (examples in **Figure 5B**) are shown in **Figures 5C–E** for the different tissues and timepoints in the growing season. While several of these flavonoids were quercetin derivatives, together with catechin/epicatechin, the relative amounts and compositions differed substantially depending upon tissue type.

Leaf, pollen, and catkin tissues had the highest concentrations of flavonoids (in naringenin equivalents), relative to the other tissues (**Figure 5E**). Most abundant metabolites in leaves were quercetin derivatives, these mainly being quercitrin (quercetin-3-*O*-rhamnoside), quercetin glucuronide, and other quercetin derivatives. By contrast, dihexosylquercetin was present as the most abundant flavonoid in pollen, while this and other quercetin derivatives were in lower abundance in catkins.

Stem and bud tissues displayed somewhat different metabolic profiles (**Figure 5D**). Of their quercetin derivatives, both isoquercitrin (quercetin 3-*O*-glucoside) and quercetin glucuronide were the most abundant of this sub-class of metabolites. In those same tissues, catechin and epicatechin were either of highest overall abundance or near highest level of abundance with smaller levels of the related PAs, procyanidin B2 and a PA trimer, at all three time points in the growing season. Buds also accumulated both a putative viscidulin III like metabolite and dimethoxyluteolin at all time points. Interestingly, levels of apigenin were highest in the Fall collected bud tissue.

Root and nodule tissues (**Figure 5C**) displayed very distinct metabolomic profiles, both in relative amounts (these being overall lowest in the two tissue types) and in compositions. Their tissues mainly accumulated differing amounts of catechin/epicatechin and PAs with overall amounts being decreased in Fall tissue. Small amounts of quercetin glucuronide and quercitrin were also present with the latter at near constant levels for all time points.

A red alder genome BLAST search gave 33 putative genes in the flavonoid pathway (**Figure 5A**; **Supplementary Tables S4, S5**; **Supplementary Figures S30**–**S40**). *ArPAL2* (as well as *ArPAL1* in catkins), *ArC4H1*, and *Ar4CL4* had been discussed in the preceding phenylpropanoid pathway section.

Following these three enzymatic steps, the further downstream flavonoid pathway gene families ranged from one (chalcone isomerase *ArCHI*; flavanone-3 hydroxylase *ArF3H*; flavonoid 3′, 5′-hydroxylase *ArF3′,5′H*; and leucoanthocyanidin dioxygenase *ArLDOX*); two (flavonoid 3′-hydroxylase *ArF3′H1* and *ArF3′H2*; flavanol synthases *ArFLS1* and *ArFLS2*; dihydroflavanol 4-reductases *ArDFR1* and *ArDFR2*; leucoanthocyanidin reductases *ArLAR1* and *ArLAR2*; and anthocyanidin reductases *ArANR1* and *ArANR2*); four (chalcone synthases *ArCHS1*, *ArCHS2*, *ArCHS3*, and *ArCHS4*, as well as flavone synthases II *ArFNSII-I, ArFNSII-2*, *ArFNSII-3*, and *ArFNSII-4*). The most likely candidate genes generally had sequence identities *ca* ≥70%, relative to *bona fide* genes in other plant species, with exception of putative *FNSII* genes which had much lower sequence identities (*ca* 48 – 57%).

The corresponding heatmap of differing expression levels of *ArPAL*, *ArC4H, Ar4CL, ArCHS*, *ArCHI, ArF3H*, *ArF3′H, ArF3′,5′H, ArFLS*, *ArDFR*, *ArLAR*, *ArLDOX*, *ArANR* and *ArFNSII* gene family members in the different tissues is shown in **Figure 5A**. [Gene expression patterns for different isoforms of the individual enzyme classes are in **Supplementary Figure S41**, with *ca* 27 of the 33 possible gene candidates having readily detectable gene expression levels albeit sometimes in a tissue specific manner].

For the enzymatic steps shared with the phenylpropanoid pathway, it had already been discussed that *ArPAL2, ArC4H1*, and *Ar4CL4* were the most highly expressed genes. For the flavonoid specific pathway, *CHS* genes *ArCHS1, ArCHS2*, and *ArCHS3* (with amino acid sequence identities of ~ 86, 74, and 86%, respectively, to *bona fide* CHSs) were highly expressed in nodules, roots, and catkins collected in Fall, and in Summer/Fall for the catkins, buds and stems (**Figure 5A**; **Supplementary Figure S41A**). *ArCHS2* expression was also highly expressed in leaves in Fall, whereas *ArCHS1* and *ArCHS3* were both expressed to lower levels. In contrast, *ArCHS4* was essentially not detected. Both ArCHS2 and ArCHS4 were, however, of somewhat lower sequence identity (*ca* 72–74%) to *bona fide* CHSs from other plant species (**Supplementary Table S5**). Furthermore, although CHS homologs suggest gateway entry points to flavonoids, related CHS-like polyketide synthase (PKS) proteins have been implicated as a gateway entry point to diarylheptanoids (Katsuyama et al., 2009) as discussed later below. *ArCHI* gene expression, by contrast, was variable, but highest in nodules and roots (Fall) and in buds (Summer and Fall) (**Figure 5A**; **Supplementary Figure S41B**).

The combined enzymatic activities of ArCHS and ArCHI result in naringenin formation (**Supplementary Figure S29**), which can then be metabolized to differentially afford quercetin, luteolin, kaempferol, apigenin, catechin, and epicatechin skeleta.

Other genes with overall high to low expression levels in different tissue types were *ArF3H*, *ArF3′,5′H* (**Supplementary Figure S41B**), *ArF3′H1, ArF3´H2* (**Supplementary Figure S41C**), *ArDFR2, ArDFR1* (**Supplementary Figure S41E**), *ArLAR2, ArLAR1* (**Supplementary Figure S41F**), and *ArLDOX, ArANR1*, *ArANR2* (**Supplementary Figure S41G**). Leaf, pollen and catkin tissues generally had much lower expression levels of these genes, with the exception of *ArFLS1* and *ArLAR1* in pollen and catkin tissues (**Supplementary Figure S41D, F**).

#### 3.3.1 Quercetin and kaempferol derivatives

From naringenin to quercetin, F3H, ArF3′H, and FLS proteins have been implicated (**Supplementary Figure S29**). Expression of the *F3H* gene (**Supplementary Figure S41B**) was highest in roots and nodules in the Fall collected tissues, and in buds in Summer/Fall, and at more moderate levels in stems, leaves, catkins, and pollen. By contrast, *ArF3′H1* and *ArF3′H2* gene expression levels (**Supplementary Figures S41C**) were more modest across all tissues, except for their very low levels in pollen tissues.

Flavonol synthase (*FLS*) genes encode the entry steps to kaempferol and quercetin (**Supplementary Figure S29**). In roots and nodules, neither *ArFLS1* nor *ArFLS2* gene expressions were detected (**Supplementary Figure S41D**); this may help explain the small to undetectable amounts of quercetin and kaempferol derivatives in those tissues. The more variable *ArFLS1/2* gene expression patterns in stem and bud tissues, with *ArFLS1* highest in Fall tissues is presumably consistent with increased levels of quercetin derivatives at the latter timepoint. Both isoforms were, however, also differentially expressed in leaf, pollen, and catkin tissues, with highest expression of *ArFLS1* overall noted in pollen, this being largely consistent with accumulation of quercetin, isoquercitrin, and dihexosylquercetin, respectively, depending upon the tissue type.

#### 3.3.2 Apigenin and luteolin derivatives

The final biosynthetic steps to apigenin and luteolin are catalyzed by flavone synthases, encoded by *FNSII* genes (**Supplementary Figure S29**). Low expression levels were generally noted for the *FNSII* genes (**Supplementary Figure S41H**): *ArFNSII-1* was mainly detected in bud (Summer/Fall) tissues, this being the tissue with highest accumulation of dimethoxyluteolin and apigenin. *ArFNSII-2* expression was observed in nodules (Spring/Summer), whereas *ArFNSII-4* had low levels of gene expression in nodules (all time-points) versus that of higher levels in roots (Spring/Fall). However, there was no strong correlation of this expression level with accumulation of either dimethoxyluteolin or apigenin. Gene expression of *ArFNSII-3* was not detected.

#### 3.3.3 Catechin, epicatechin, and procyanidin derivatives

*ArLAR2/ArLAR1* and *ArLDOX/ArANR1*/*ArANR2* reportedly encode proteins catalyzing the last steps to (+)-catechin (Tanner et al., 2003) and (–)-epicatechin (Pelletier et al., 1999; Xie et al., 2003), respectively (**Supplementary Figures S41F, G**). Of these, the low expression levels of *ArLDOX*, *ArANR1*, and *ArANR2* in leaf, pollen, and catkin tissues may account for the lower levels of (–)-epicatechin and procyanidin B2, relative to other tissue types (**Figure 5E**). Additionally, the low level of expression noted for *ArDFR1/2* in leaves and pollen may provide insight into the absence of (+)-catechin and procyanidin B2 in pollen, and their low levels in leaves.

### 3.4 Proanthocyanidins, ellagitannins, and diarylheptanoids

**Figure 6:**
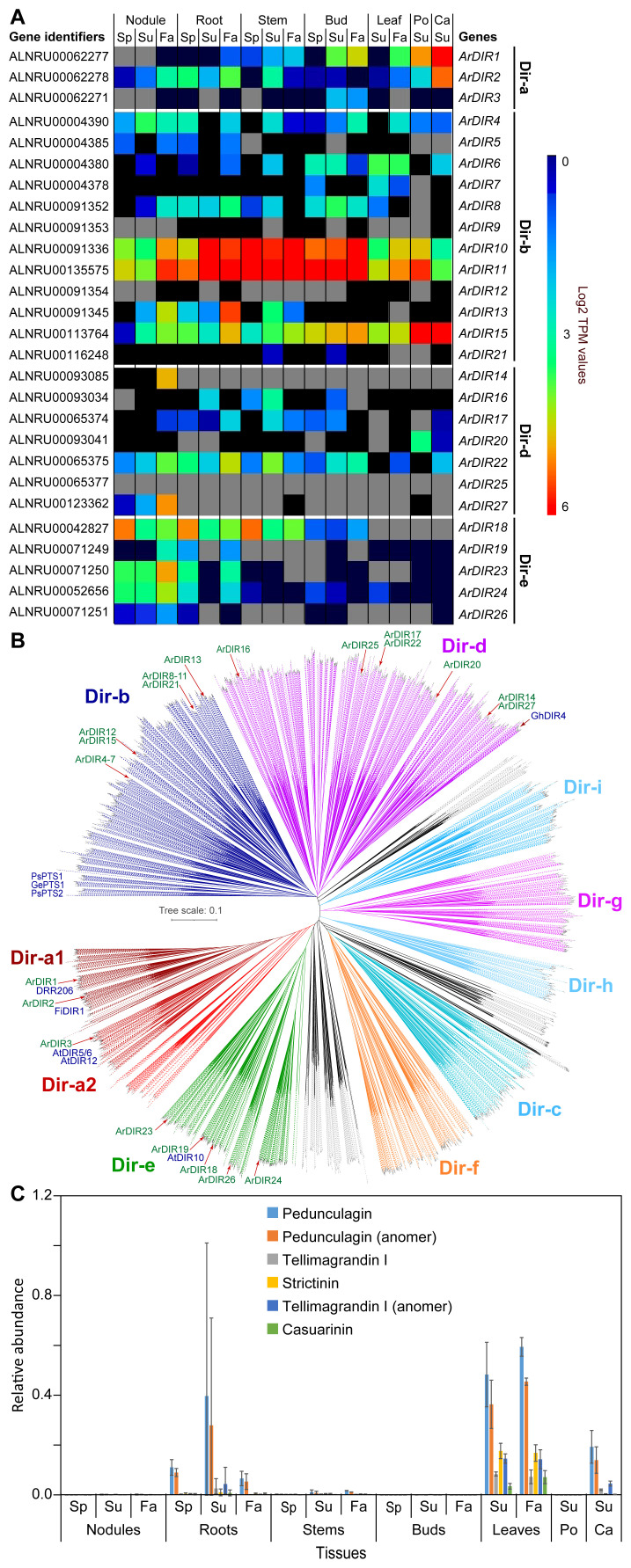
Dirigent proteins and ellagitannins. **(A)** Heatmap showing average Log2 TPM values obtained from transcriptomics analysis for the dirigent protein family. *Grey* shading in the heatmap indicates that there were no reads detected. **(B)** Unrooted phylogenetic tree of dirigent and dirigent-like protein family. The sub-family nomenclature of Ralph et al. (2007) is used. Dirigent proteins, whose functional characterization are known in the literature are indicated (e.g. DRR206 in lignan formation, and GePTS1, PsPTS1, PsPTS2 in pterocarpan/isoflavene biosynthesis). **(C)** Relative abundance of ellagitannins in naringenin equivalents. Ca, catkins; Fa, fall; Po, pollen; Sp, spring; Su, summer.

From our metabolomics analyses, red alder tissues have at least 2 PAs, procyanidin B2 and a PA trimer (**Figures 5B–E**), 6 ellagitannins or “hydrolysable tannins” (**Figure 6C**), and 25 diarylheptanoids (**Figures 4A, B**; **Supplementary Figures S16A–D**; **Supplementary Table S2**). While biochemical pathways for formation of substrates prior to entry into these three metabolic classes are well-established, the proteinaceous biochemical entry points to PAs, ellagitannins, and *potentially* diarylheptanoids, represent a major gap in our scientific knowledge, as do their downstream (post-gateway entry) metabolic processes. That is, genes encoding proteins affording entry points to both ellagitannins and PAs, as well as *potentially* to diarylheptanoids, are either unknown or poorly understood, in spite of their widespread and enormous chemical structural diversity across the plant kingdom.

#### 3.4.1 Proanthocyanidins

The two PAs identified in red alder thus far are procyanidin B2, and a putative PA trimer. The former is of significant medical interest due to its ability to stimulate hair/beard growth (Kamimura et al., 2000; Tenore et al., 2018), as well as displaying anticancer properties (Zhang et al., 2019; Li et al., 2021). A central biochemical question is how PA formation, such as to procyanidin B2, occurs *in planta*. In 1983, it was reported that leucocyanidin, under acidic conditions *in vitro* and in the presence of excess (+)-catechin, affords PAs such as procyanidin B3 and B6 (Delcour et al., 1983). However, it has also long been considered that naturally occurring PAs are formed *in vivo* under proteinaceous control (Kristiansen, 1983, 1984; Lewis and Yamamoto, 1989), but with no demonstration to date of the biochemical machinery involved.

Both procyanidin B2 and the PA trimer had highest amounts in stem and bud tissues (**Figure 5D**), as well as in roots and nodules (**Figure 5C**), and to a lesser extent in catkins (**Figure 5E**), as also previously noted for (+)-catechin and (–)-epicatechin. [The biosynthetic pathways to both (–)-epicatechin and (+)-catechin were also previously shown in **Supplementary Figure S29**, and whose gene expression profiles established that all genes from *ArCHS* to *ArANR1/2* were expressed in essentially all 7 tissue types (**Supplementary Figures S41A–C, E–G**)].

#### 3.4.2 Ellagitannins

*β*-glucogallin (**Supplementary Figure S1**), found in all tissue types and considered the gateway entry point substrate to bioactive ellagitannins, is sequentially metabolized to di-, tri-, tetra- and penta-galloyl glucose. This enzymatically occurs via galloyl residues reportedly sequentially added through *trans*-esterification, using *β*-glucogallin as both galloyl donor and acceptor in a very specific order with the galloyl moieties added sequentially at the 6, 2, 3, and 4 positions of glucose, respectively (Niemetz and Gross, 2005). However, to our knowledge, none of the encoding genes involved have yet been described leading to pentagalloyl glucose, nor those of subsequent downstream conversions to the ellagitannins.

In addition to the above glucosylated galloyl metabolites, various red alder tissues accumulate ellagitannins, these occurring mainly in root, leaf, catkin and, to a much lesser extent, stem tissues (**Figure 6C**), of which the putative pedunculagin-like metabolites are most abundant.

#### 3.4.3 Potential PA and ellagitannin gateway entry points – red alder dirigent protein sub-families

PAs and ellagitannins are often isolated in optically active forms in different plant species, and whose metabolites encompass enormous species-specific structural diversity and molecular size (Niemetz and Gross, 2005; Constabel, 2018; Zhou et al., 2022). Optical activity strongly suggests overall proteinaceous control during their various assemblies, and thus not being formed via action of non-specific oxidases (such as peroxidases) alone. Such oxidases can generate both racemic products and different regio-chemistries following coupling than those naturally accumulating *in planta*. Gateway entry proteins to optically active PAs and ellagitannins though have not been reported to date.

Dirigent proteins, DPs, (Latin: *dirigere*, to guide or align) are increasingly recognized as gateway entry points to distinct land plant phenol metabolic classes (Davin et al., 2022; Meng et al., 2023). Although there are also various outliers, DPs have at least 9 main sub-families (see **Figure 6B**) which are classified from Dir-a to Dir-i (Ralph et al., 2007; Corbin et al., 2018; Davin et al., 2022). Importantly, DP emergence appears to have coincided with transition of plants from aquatic origins to land, i.e. given that algae and cyanobacteria lack DP-encoding genes. Currently, the known DP gateway entry points include to bioactive 8–8′- and 8–*O*–4′-linked lignans (Dir-a sub-family) (Davin et al., 1997; Corbin et al., 2018; Yonekura-Sakakibara et al., 2021; Davin et al., 2022), cell wall reinforcing lignins (Dir-e) (Hosmani et al., 2013; Gao et al., 2023), bioactive aromatic diterpenoids (now placed in Dir-d) (Liu et al., 2008; Effenberger et al., 2015), as well as to anti-fungal pterocarpans and isoflavenes (now placed in Dir-b) (Meng et al., 2020; Davin et al., 2022; Meng et al., 2023, 2024). However, the vast number of DP-encoding genes (*~*95%) in the plant kingdom await discovery of physiological/biochemical functions, including in red alder.

From a biochemical mechanism perspective, PA entry point biosynthesis - such as to procyanidin B2 and its higher oligomers - can putatively involve protein-guided generation of a reactive (presumed quinone methide, QM) intermediate derived from leucocyanidin which then undergoes regio-specific “coupling” with (–)-epicatechin (see **Supplementary Figure S29** for both structures). The gateway entry point could thus be somewhat mechanistically analogous to Dir-b sub-family members that engender formation of anti-fungal pterocarpans and isoflavenes in pea (*Pisum sativum*) (Meng et al., 2020; Davin et al., 2022; Meng et al., 2023, 2024). However, whether this would occur solely with DPs, or if other proteins would be involved, is not yet known.

Additionally, stereoselective coupling to afford (optically active) ellagitannins could conceivably comparably occur as for either engendered Dir-a lignan (Davin et al., 1997, 2022) or Dir-d aromatic diterpenoids (Liu et al., 2008; Effenberger et al., 2015) biosynthesis, provided an auxiliary oxidase is present.

We therefore hypothesize herein that gateway entry points to PAs and ellagitannins may involve different DPs. There are 27 red alder DP genes, albeit all currently of unknown biochemical function. These include 3 in the Dir-a sub-family, 12 in the Dir-b sub-family, 7 in the Dir-d subfamily, and 5 in the Dir-e sub-family (**Figure 6A**). Their relative placement in the different Dir subfamilies is also shown in **Figure 6B**.

**Supplementary Table S6** lists the corresponding red alder Dir-a, Dir-b, Dir-d, and Dir-e DP sub-family members, and their amino acid comparison (**Supplementary Figures S42**–**S45**) to known homologues. Amino acid sequence identity comparisons of the 3 Dir-a homologs to the (+)-pinoresinol forming DRR206 from pea [involved in 8-8′-linked lignan biosynthesis] range from 73.2 – 51.2% making it somewhat uncertain as to whether they have the same biochemical function (**Supplementary Table S6**). The 12 and 7 red alder Dir-b and Dir-d sub-family genes also have relatively low sequence identities (55.7 – 38.1%) to pterocarpan/isoflavene/diterpenoid forming homologues.

Accordingly, as hypothetical entry points to these biosynthetic pathways, the expression of red alder DPs at different levels in each tissue type was analyzed (**Supplementary Figures S46**, **S47**), with a cumulative gene expression heatmap shown in **Figure 6A**. Perhaps significantly, gene expression data for Dir-b sub-family members *ArDIR10* and *ArDIR11* in all tissue types (**Supplementary Figure S47A**) was strikingly similar to the increased accumulation levels of procyanidin B2 and the procyanidin trimer (**Figures 5C, D**), as well as (–)-epicatechin and (+)-catechin, relative to other tissue types, i.e. perhaps suggesting one or more of these is (are) involved in PA biosynthesis. Another Dir-b gene highly expressed was *ArDIR15* in pollen and catkin tissues (**Supplementary Figure S47A**), as well as in all other tissue types albeit to a much lower extent - however, pollen appears to be devoid of catechin/epicatechin and PAs, whereas catkins have them in very low amounts.

DIR-a sub-family members in other species have only been shown thus far to be associated with 8–8′ and more recently 8–*O*–4′ linked lignan gateway entry points, perhaps indicative of a similar role in red alder. Of the 3 membered Dir-a gene sub-family in red alder, *ArDIR1* and *ArDIR2* were predominantly expressed in catkins (**Supplementary Figure S46A**). *ArDIR2* was also expressed to low levels in nodules and roots, as well as at lower levels in stems, buds, leaves and pollen. Relative to catkins, *ArDIR1* had lower expression levels in essentially all tissue types with the least expression being in nodules and roots. *ArDIR3* had the lowest level of gene expression overall, only being found in buds, and particularly in its Summer and Fall tissues. However, in the metabolomics analysis of each tissue type, no lignans were detected under the conditions employed.

Provisionally, it can be considered though that the relative gene expression profiles of Dir-a sub-family members *ArDIR1* and *ArDIR2* (**Supplementary Figure S46A**) are possibly involved in either ellagitannin or lignan biosynthesis in roots, leaves, and catkins, even though there were no ellagitannins detected in pollen. However, other DPs in the Dir-b sub-family (**Supplementary Figure S47A**), such as *ArDIR15*, cannot currently be excluded for ellagitannin biogenesis.

Addressing the open questions of how PAs and ellagitannins are formed will be the subject of future studies.

#### 3.4.4 Diarylheptanoids and gateway entry point

Highly abundant in nearly all red alder tissues (pollen excepted), the diarylheptanoid oregonin and its glucoside analog hirsutanonol-5-*O*-glucoside (**Figure 4B**) are very abundant, as are some of their derivatives (**Supplementary Figure S16B**). Other abundant diarylheptanoids in most tissues are rubranosides A-D (**Supplementary Figure S16A**), alnuside B and its related metabolites (**Supplementary Figure S16C**) and, to a lesser extent, platyphylloside and related (**Supplementary Figure S16D**). Of these, the non-oregonin diarylheptanoids that are provisionally structurally annotated and largely downstream metabolites vary chemically in different tissues, i.e. depending on aromatic ring hydroxylation pattern (ranging from 1 – 4 phenolic OH groups), and position of ketone and secondary alcohol functionalities in “heptanoid” sub-structures. Other variations included glucosylation, xylosylation, and ester formation (e.g. 2-methylbutanoyl, cinnamoyl, *p*-coumaryl and galloyl esterified moieties) (**Supplementary Table S2**). The overall physiological significance of these chemical structure variations, however, is currently unknown, as are their specific biochemical transformations leading to their formation.

Physiologically, oregonin can act as an anti-herbivory agent, e.g. against leaf eating insects (Lea et al., 2021). However, diarylheptanoids not only have plant defense functions, but also a) give rise to reddish-orange dyes used by Native Americans and b) many have important medicinal properties. For example, reddish-orange red alder wood and bark coloration occurring after either felling or weather induced injuries (i.e. via wind, rainstorm, lightning, etc.) has long been linked to oregonin. Coloration was attributed to its oxidation by peroxidase/H_2_O_2_ (Karchesy et al., 1974), peroxidase and polyphenol oxidase (Hrutfiord and Luthi, 1981), and/or catechol oxidase (Terazawa et al., 1984), respectively. Root nodules are constitutively bright orange (**Figure 1C**), presumably due to comparable enzymatic oxidation within nodules.

As indicated earlier, entry to the diarylheptanoid metabolic class has implicated action of CHS homologs, also described as type III polyketide synthases (PKSs). In turmeric (*Curcuma longa*), diarylheptanoid gateway entry points were reportedly catalyzed by two PKSs, named ClDCS and ClCURS (Katsuyama et al., 2009) (**Supplementary Figure S48**). ClDCS reportedly catalyzed a reaction using feruloyl CoA and malonyl CoA to afford feruloyl diketide CoA, while ClCURS converted this product together with another molecule of feruloyl CoA to give curcumin.

A BLAST search of the red alder genome gave no obvious homologs to either ClDCS or ClCURS. Instead, our analysis only identified 4 potential CHS-like genes (*ArCHS1–4* (**Supplementary Figure S30**). Comparison of their corresponding amino acid sequences showed they had higher identity (**Supplementary Table S7**) to a *bona fide* CHS, i.e. *Antirrhinum majus* CHS (*ca* 86 – 73%), as compared to both ClDCS and ClCURS (*ca* 49 – 66%).

RNA-seq analyses established that 3 of the 4 *CHS* homologs were differentially expressed in nodules, roots, buds, leaves, stems, and catkins, with *ArCHS3* and *ArCHS1* generally most highly expressed and having identities of *ca* 86% to CHSs proper (**Supplementary Figure S41A**; **Supplementary Table S7**). In those same tissues, *ArCHS2* had lower relative expression levels for all time-points, whereas in leaf (particularly Fall harvested) and catkins, its gene expression predominated; it also had lower identity (73.9%) to known CHSs. On the other hand, its gene expression profile more closely followed the patterns of diarylheptanoid deposition.

Pollen, by contrast, had only *ArCHS3* apparently faintly expressed and the fourth homolog *ArCHS4* also had very low expression. It thus needs to be established whether all of these are actually *bona fide* CHSs, or if only two are. This will also be the subject of future enquiry, as the genomic data does not yet provide a clear indication as to what are CHSs proper *vs* diarylheptanoid entry points, and whether any of these are even involved in red alder diarylheptanoid biosynthesis.

#### 3.4.5 Dirigent protein Dir-e sub-family and lignification

The 5-membered red alder Dir-e sub-family has not been studied either, but these are in the same sub-family implicated in constitutive Casparian band lignification in *Arabidopsis* (Hosmani et al., 2013; Gao et al., 2023), even though red alder Dir-e protein sequence identities range from *ca* 61 – 36% compared to the *Arabidopsis* homolog (**Supplementary Table S6**). However, both in *Arabidopsis* and red alder, their precise biochemical substrates and products are unknown. Nevertheless, *ArDIR18* is expressed in nodules, roots, and stems at all time-points being highest in the Spring tissues (**Supplementary Figure S46B**). That is, it is perhaps striking that they are largely expressed in stems and roots that undergo lignification. In the nodule tissues, *ArDIR23* and *ArDIR24* are next most highly expressed, this increasing in Fall tissues. Roots show a somewhat similar profile. The other Dir-e gene sub-family members, however, have very low expression in most tissues. Again, the biochemical functions for these DPs are as yet unknown and require full clarification at the protein level, in terms of substrates utilized and corresponding products obtained.

#### 3.4.6 Dir-a sub-family known downstream metabolic processes

There are 8 red alder gene homologs (**Supplementary Table S8**; **Supplementary Figures S49**) of various plant phenol reductase classes, i.e. one pinoresinol-lariciresinol reductase/pinoresinol reductase (ArPLR) (Dinkova-Kostova et al., 1996), and seven allylphenol/propenylphenol synthases (ArAPS1-5 and ArPPS1/2) (Vassão et al., 2007; Kim et al., 2014). These 8 proteins were used to generate a phylogenetic tree (**Supplementary Figure S50**), together with known phenylcoumaran benzylic ether reductase (PCBER) and isoflavone reductase (IFR) sub-family members (Vassão et al., 2008). Additionally, the phylogenetic tree includes the leucoanthocyanidin reductase (LAR, see above).

Of these, ArPLR clusters within the PLR/PR clade and has high sequence identity at the protein level to *Forsythia intermedia* PLR of 77.5% (**Supplementary Table S8**). *ArPLR*, however, was only expressed at a very low level in all tissues (**Supplementary Figure S51A**). Provisionally, it can be considered as a PLR/PR, but this needs to be determined at both the protein level and *in planta*.

Five of the others (ArAPS1, ArAPS2, ArAPS3, ArAPS4, and ArAPS5) cluster with the APS clade, with sequence identities at the protein level ranging from 81.8 to 77.0% to LtAPS1 from *Larrea tridentata*. The last two (ArPPS1 and ArPPS2) cluster with the PPS clade with identities around 63–64% to LtPPS1. APS and PPS proteins are presumed present in all alder species. For examples, eugenol is a volatile component in male and female flowers, as well as in young leaves of *A. sieboldiana* (Ghani et al., 2016), and eugenol/chavicol were both found in *A. pendula* male flowers (Suga et al., 1972). In terms of gene expression patterns, *ArAPS1* and *ArAPS2* are the most highly expressed in all tissue types, and at all time-points (**Supplementary Figure S51A**). By comparison, *ArAPS3* is expressed in nodules and roots, but only in Fall collected tissues. *ArAPS4* and *ArAPS5* were, however, expressed only in catkins, whereas *ArPPS1* and *ArPPS2* were expressed at very low levels in various tissues. Provisionally, these may be gene candidates encoding proteins able to biosynthesize eugenol/chavicol and/or related monomeric volatiles.

There are also 8 secoisolariciresinol dehydrogenase (SDH) (Xia et al., 2001) homologs, respectively, these being of relatively low identities (*ca* 50 – 56%) to a *bona fide* SDH from *Podophyllum peltatum* (**Supplementary Table S8**; **Supplementary Figure S52**). The ones most highly expressed were *ArSDH5* and *ArSDH3* in catkins, *ArSDH7* and *ArSDH8* in roots and nodules, and to a much lesser extent in stems, as well as *ArSDH6* but mainly in root, nodule, and leaf tissues in Fall and catkins (**Supplementary Figure S51B**). Their biochemical functions also need to be established, i.e. as to whether they are SDHs proper and/or have different biochemical functions.

## 4 Concluding remarks

Red alder Clone 639 was obtained as part of an earlier Weyerhaeuser research program to produce trees with superior growth characteristics (e.g. rapid growth, straight log development, superior wood attributes). This clone, among others, was provided to Washington State University for research purposes. This has enabled both its genome sequencing and molecular characterization.

Based on our genome sequencing (Hixson et al., 2023), as well as from the RNA-seq and metabolomics analyses herein, our work begins to lay down the foundation as to the molecular basis for red alder’s manifold plant defense, medicinal, and red-orange dye properties.

Additionally, these data should be able to begin to help address its long historical commercial uses – including in better understanding its remarkable properties as timber in aquatic environments, its commodity wood/fiber products, and for its musical instrument usage. Such analyses are envisaged to open-up the possibility of, for example, developing red alder further for lumber, bioenergy/biomaterial crops in marginal soils, land-reclamation, and/or large-scale carbon sequestration, and various medicinal/plant defense purposes.

## Data Availability

The datasets presented in this study can be found in online repositories. The names of the repository/repositories and accession number(s) can be found below: https://www.ncbi.nlm.nih.gov/, PRJNA691057.

## References

[B1] Alcázar Magaña A. Kamimura N. Soumyanath A. Stevens J. F. Maier C. S.. (2021). Caffeoylquinic acids: Chemistry, biosynthesis, occurrence, analytical challenges, and bioactivity. Plant. J. 107, 1299–1319. doi: 10.1111/tpj.15390 PMC9084498 34171156

[B2] Alder Tree Spiritual Meaning. Available online at: http://www.thegoddesstree.com/trees/Alder.htm (Accessed August 04, 2022).

[B3] Bontpart T. Marlin T. Vialet S. Guiraud J.-L. Pinasseau L. Meudec E.. (2016). Two shikimate dehydrogenases, *VvSDH3* and *VvSDH4*, are involved in gallic acid biosynthesis in grapevine. J. Exp. Bot. 67, 3537–3550. doi: 10.1093/jxb/erw184 27241494 PMC4892741

[B4] Cho M.-H. Corea O. R. A. Yang H. Bedgar D. L. Laskar D. D. Anterola A. M.. (2007). Phenylalanine biosynthesis in *Arabidopsis thaliana*: Identification and characterization of arogenate dehydratases. J. Biol. Chem. 282, 30827–30835. doi: 10.1074/jbc.M702662200 17726025

[B5] Constabel C. P. (2018). Molecular controls of proanthocyanidin synthesis and structure: Prospects for genetic engineering in crop plants. J. Agric. Food Chem. 66, 9882–9888. doi: 10.1021/acs.jafc.8b02950 30139248

[B6] Corbin C. Drouet S. Markulin L. Auguin D. Lainé É. Davin L. B.. (2018). A genome-wide analysis of the flax (*Linum usitatissimum* L.) dirigent protein family: From gene identification and evolution to differential regulation. Plant Mol. Biol. 97, 73–101. doi: 10.1007/s11103-018-0725-x 29713868

[B7] Corea O. R. A. Ki C. Cardenas C. L. Kim S.-J. Brewer S. E. Patten A. M.. (2012). Arogenate dehydratase isoenzymes profoundly and differentially modulate carbon flux into lignins. J. Biol. Chem. 287, 11446–11459. doi: 10.1074/jbc.M111.322164 22311980 PMC3322856

[B8] Cui L. Yao S. Dai X. Yin Q. Liu Y. Jiang X.. (2016). Identification of UDP-glycosyltransferases involved in the biosynthesis of astringent taste compounds in tea (*Camellia sinensis*). J. Exp. Bot. 67, 2285–2297. doi: 10.1093/jxb/erw053 26941235 PMC4809296

[B9] d’Amato T. A. Ganson R. J. Gaines C. G. Jensen R. A. (1984). Subcellular localization of chorismate mutase isoenzymes in protoplasts from mesophyll and suspension-cultured cells of *Nicotiana silvestris* . Planta 162, 104–108. doi: 10.1007/Bf00410205 24254043

[B10] Davin L. B. Cort J. R. Smith C. A. Meng Q. M. Moinuddin S. G. A. Costa M. A.. (2022). “Dirigent protein roadmap to lignans and other vascular plant phenol classes,” in The Lignan Handbook. Eds. Lewis N. G. Davin L. B. Munasinghe V. R. N. Roberts A. D. (Taylor & Francis, Boca Raton, London), 57–77.

[B11] Davin L. B. Wang H. W. Crowell A. L. Bedgar D. L. Martin D. M. Sarkanen S.. (1997). Stereoselective bimolecular phenoxy radical coupling by an auxiliary (dirigent) protein without an active center. Science 275, 362–366. doi: 10.1126/science.275.5298.362 8994027

[B12] Delcour J. A. Ferreira D. Roux D. G. (1983). Synthesis of condensed tannins. Part 9. The condensation sequence of leucocyanidin with (+)-catechin and with the resultant procyanidins. J. Chem. Soc. Perkin Trans. 1, 1711–1717. doi: 10.1039/P19830001711

[B13] Dinkova-Kostova A. T. Gang D. R. Davin L. B. Bedgar D. L. Chu A. Lewis N. G. (1996). (+)-Pinoresinol/(+)-lariciresinol reductase from *Forsythia intermedia*: Protein purification, cDNA cloning, heterologous expression and comparison to isoflavone reductase. J. Biol. Chem. 271, 29473–29482. doi: 10.1074/jbc.271.46.29473 8910615

[B14] Dunn N. A. Unni D. R. Diesh C. Munoz-Torres M. Harris N. L. Yao E.. (2019). Apollo: Democratizing genome annotation. PloS Comp. Biol. 15, e1006790. doi: 10.1371/journal.pcbi.1006790 PMC6380598 30726205

[B15] Effenberger I. Zhang B. Li L. Wang Q. Liu Y. Klaiber I.. (2015). Dirigent proteins from cotton (*Gossypium* sp.) for the atropselective synthesis of gossypol. Angew. Chem. Int. Ed. Engl. 54, 14660–14663. doi: 10.1002/anie.201507543 26460165

[B16] Fahrendorf T. Dixon R. A. (1993). Stress responses in alfalfa (*Medicago sativa* L.). XVIII: Molecular cloning and expression of the elicitor-inducible cinnamic acid 4-hydroxylase cytochrome P450. Arch. Biochem. Biophys. 305, 509–515. doi: 10.1006/abbi.1993.1454 8373188

[B17] Forest D. (2014). Celtic Tree Magic: Ogham Lore and Druid Mysteries (Woodbury, MN: Llewellyn Worldwide, Limited).

[B18] Forlines D. R. Tavenner T. Malan J. C. S. Karchesy J. J. (1992). “Plants of the Olympic coastal forests: Ancient knowledge of materials and medicines and future heritage,” in Plant Polyphenols. Eds. Hemingway R. W. Laks P. E. (Springer, Boston, MA), 767–782. doi: 10.1007/978-1-4615-3476-1_46 1417699

[B19] Gang D. R. Beuerle T. Ullmann P. Werck-Reichhart D. Pichersky E. (2002). Differential production of *meta* hydroxylated phenylpropanoids in sweet basil peltate glandular trichomes and leaves is controlled by the activities of specific acyltransferases and hydroxylases. Plant Physiol. 130, 1536–1544. doi: 10.1104/pp.007146 12428018 PMC166672

[B20] Gao Y. Q. Huang J. Q. Reyt G. Song T. Love A. Tiemessen D.. (2023). A dirigent protein complex directs lignin polymerization and assembly of the root diffusion barrier. Science 382, 464–471. doi: 10.1126/science.adi5032 37883539

[B21] Garrow D. Sturt F. (2019). Neolithic crannogs: Rethinking settlement, monumentality and deposition in the Outer Hebrides and beyond. Antiquity 93, 664–684. doi: 10.15184/aqy.2019.41

[B22] Ghani N. A. Ismail N. H. Asakawa Y. (2016). Comparative study of the volatile components of fresh and fermented flowers of *Alnus sieboldiana* (Betulaceae). Nat. Prod. Commun. 11, 265–266. doi: 10.1177/1934578X1601100233 27032217

[B23] Gifford J. (2001). “Alder, tree of the god Bran,” in The Wisdom of Trees. Ed. Gifford J. (Sterling Publishing Co, New York, NY), 40–47.

[B24] Grabherr M. G. Haas B. J. Yassour M. Levin J. Z. Thompson D. A. Amit I.. (2011). Full-length transcriptome assembly from RNA-Seq data without a reference genome. Nat. Biotechnol. 29, 644–652. doi: 10.1038/nbt.1883 21572440 PMC3571712

[B25] Gritsunov A. Peek J. Diaz Caballero J. Guttman D. Christendat D. (2018). Structural and biochemical approaches uncover multiple evolutionary trajectories of plant quinate dehydrogenases. Plant J. 95, 812–822. doi: 10.1111/tpj.13989 29890023

[B26] Guo J. Carrington Y. Alber A. Ehlting J. (2014). Molecular characterization of quinate and shikimate metabolism in *Populus trichocarpa* . J. Biol. Chem. 289, 23846–23858. doi: 10.1074/jbc.M114.558536 24942735 PMC4156088

[B27] Gutiérrez Ortiz A. L. Berti F. Navarini L. Crisafulli P. Colomban S. Forzato C. (2018). Aqueous extracts of walnut (*Juglans regia* L.) leaves: Quantitative analyses of hydroxycinnamic and chlorogenic acids. J. Chromatogr. Sci. 56, 753–760. doi: 10.1093/chromsci/bmy041 29762631 PMC6296405

[B28] Hart S. C. Binkley D. Perry D. A. (1997). Influence of red alder on soil nitrogen transformations in two conifer forests of contrasting productivity. Soil Biol. Biochem. 29, 1111–1123. doi: 10.1016/S0038-0717(97)00004-7

[B29] Hixson K. K. Fajardo D. A. Devitt N. P. Sena J. A. Costa M. A. Meng Q.. (2023). Annotated genome sequence of a fast-growing diploid clone of red alder (*Alnus rubra* Bong.). G3 13, jkad060. doi: 10.1093/g3journal/jkad060 36966434 PMC10234377

[B30] Höhner R. Marques J. V. Ito T. Amakura Y. Budgeon A. D. Jr. Weitz K.. (2018). Reduced arogenate dehydratase expression: Ramifications for photosynthesis and metabolism. Plant Physiol. 177, 115–131. doi: 10.1104/pp.17.01766 PMC5933128 29523714

[B31] Hosmani P. S. Kamiya T. Danku J. Naseer S. Geldner N. Guerinot M. L.. (2013). Dirigent domain-containing protein is part of the machinery required for formation of the lignin-based Casparian strip in the root. Proc. Natl. Acad. Sci. U.S.A. 110, 14498–14503. doi: 10.1073/pnas.1308412110 23940370 PMC3761638

[B32] Hrutfiord B. F. Luthi R. (1981). Chemistry of oregonin. Ekman-Days 1981 Int. Symp. Wood Pulping Chem. 1, 95–98.

[B33] Jourdes M. Cardenas C. L. Laskar D. D. Moinuddin S. G. A. Davin L. B. Lewis N. G. (2007). Plant cell walls are enfeebled when attempting to preserve native lignin configuration with *poly*-*p*-hydroxycinnamaldehydes: Evolutionary implications. Phytochemistry 68, 1932–1956. doi: 10.1016/j.phytochem.2007.03.044 17559892

[B34] Kamimura A. Takahashi T. Watanabe Y. (2000). Investigation of topical application of procyanidin B-2 from apple to identify its potential use as a hair growing agent. Phytomedicine 7, 529–536. doi: 10.1016/S0944-7113(00)80040-9 11194183

[B35] Karchesy J. J. Laver M. L. Barofsky D. F. Barofsky E. (1974). Structure of oregonin, a natural diarylheptanoid xyloside. J. Chem. Soc. Chem. Commun., 649–650. doi: 10.1039/C39740000649

[B36] Katsuyama Y. Kita T. Funa N. Horinouchi S. (2009). Curcuminoid biosynthesis by two type III polyketide synthases in the herb *Curcuma longa* . J. Biol. Chem. 284, 11160–11170. doi: 10.1074/jbc.M900070200 19258320 PMC2670121

[B37] Kim S.-J. Kim M.-R. Bedgar D. L. Moinuddin S. G. A. Cardenas C. L. Davin L. B.. (2004). Functional reclassification of the putative cinnamyl alcohol dehydrogenase multigene family in *Arabidopsis* . Proc. Natl. Acad. Sci. U.S.A. 101, 1455–1460. doi: 10.1073/pnas.0307987100 14745009 PMC341741

[B38] Kim D. Paggi J. M. Park C. Bennett C. Salzberg S. L. (2019). Graph-based genome alignment and genotyping with HISAT2 and HISAT-genotype. Nat. Biotechnol. 37, 907–915. doi: 10.1038/s41587-019-0201-4 31375807 PMC7605509

[B39] Kim S.-J. Vassão D. G. Moinuddin S. G. A. Bedgar D. L. Davin L. B. Lewis N. G. (2014). Allyl/propenyl phenol synthases from the creosote bush and engineering production of specialty/commodity chemicals, eugenol/isoeugenol, in *Escherichia coli* . Arch. Biochem. Biophys. 541, 37–46. doi: 10.1016/j.abb.2013.10.019 24189289

[B40] Klaassen R. K. W. M. Creemers J. G. M. (2012). Wooden foundation piles and its underestimated relevance for cultural heritage. J. Cult. Herit. 13, S123–S128. doi: 10.1016/j.culher.2012.02.014

[B41] Kristiansen K. N. (1983). Biosynthesis of proanthocyanidins in barley. Proc. Congr. Eur. Brew. Conv., 511–516.

[B42] Kristiansen K. N. (1984). Biosynthesis of proanthocyanidins in barley: Genetic-control of the conversion of dihydroquercetin to catechin and procyanidins. Carlsberg Res. Commun. 49, 503–524. doi: 10.1007/Bf02907552

[B43] Lai Y.-C. Chen C.-K. Lin W.-W. Lee S.-S. (2012). A comprehensive investigation of anti-inflammatory diarylheptanoids from the leaves of *Alnus formosana* . Phytochemistry 73, 84–94. doi: 10.1016/j.phytochem.2011.02.008 21388646

[B44] Lea C. S. Bradbury S. G. Constabel C. P. (2021). Anti-herbivore activity of oregonin, a diarylheptanoid found in leaves and bark of red alder (*Alnus rubra*). J. Chem. Ecol. 47, 215–226. doi: 10.1007/s10886-021-01244-3 33475940

[B45] Letunic I. Bork P. (2021). Interactive Tree Of Life (iTOL) v5: An online tool for phylogenetic tree display and annotation. Nucleic Acids Res. 49, W293–W296. doi: 10.1093/nar/gkab301 PMC8265157 33885785

[B46] Lewis N. G. Yamamoto E. (1989). “Tannins: Their place in plant metabolism,” in Chemistry and Significance of Condensed Tannins. Eds. Hemingway R. W. Karchesy J. J. (New York, NY: Plenum Press), 23–46. doi: 10.1007/978-1-4684-7511-1_2

[B47] Li Y. Lu X. Tian P. Wang K. Shi J. (2021). Procyanidin B2 induces apoptosis and autophagy in gastric cancer cells by inhibiting Akt/mTOR signaling pathway. BMC Complementary Med. Therapies 21, 76. doi: 10.1186/s12906-021-03225-1 PMC7905658 33627124

[B48] Liu J. Stipanovic R. D. Bell A. A. Puckhaber L. S. Magill C. W. (2008). Stereoselective coupling of hemigossypol to form (+)-gossypol in moco cotton is mediated by a dirigent protein. Phytochemistry 69, 3038–3042. doi: 10.1016/j.phytochem.2008.06.007 18639908

[B49] Lu D. Yuan X. Kim S.-J. Marques J. V. Chakravarthy P. P. Moinuddin S. G. A.. (2017). Eugenol specialty chemical production in transgenic poplar (*Populus tremula* × *P. alba*) field trials. Plant Biotechnol. J. 15, 970–981. doi: 10.1111/pbi.12692 28064439 PMC5506655

[B50] Madeira F. Madhusoodanan N. Lee J. Eusebi A. Niewielska A. Tivey A. R. N. (2024). The EMBL-EBI Job Dispatcher sequence analysis tools framework in 2024. Nucleic Acids Res. 52, W521–W525. doi: 10.1093/nar/gkae241 PMC11223882 38597606

[B51] Mahesh V. Million-Rousseau R. Ullmann P. Chabrillange N. Bustamante J. Mondolot L.. (2007). Functional characterization of two *p*-coumaroyl ester 3´-hydroxylase genes from coffee tree: Evidence of a candidate for chlorogenic acid biosynthesis. Plant Mol. Biol. 64, 145–159. doi: 10.1007/s11103-007-9141-3 17333503

[B52] Mehmood M. A. Ibrahim M. Rashid U. Nawaz M. Ali S. Hussain A.. (2017). Biomass production for bioenergy using marginal lands. Sustain. Prod. Consum. 9, 3–21. doi: 10.1016/j.spc.2016.08.003

[B53] Meng Q. Kim S. J. Costa M. A. Moinuddin S. G. A. Celoy R. M. Smith C. A.. (2023). “Dirigent protein subfamily function and structure in terrestrial plant phenol metabolism,” in Methods in Enzymology: Biochemical Pathways and Environmental Responses in Plants. Ed. Jez J. (Cambridge, MA: Academic Press), 101–150. doi: 10.1016/bs.mie.2023.02.025 37087184

[B54] Meng Q. Moinuddin S. G. A. Celoy R. M. Smith C. A. Young R. P. Costa M. A.. (2024). Dirigent isoflavene-forming PsPTS2: 3D Structure, stereochemical and kinetic characterization comparison with pterocarpan-forming PsPTS1 homolog in pea. J. Biol. Chem. 300, 105647. doi: 10.1016/j.jbc.2024.105647 38219818 PMC10882141

[B55] Meng Q. Moinuddin S. G. A. Kim S.-J. Bedgar D. L. Costa M. A. Thomas D. G.. (2020). Pterocarpan synthase (PTS) structures suggest a common quinone methide-stabilizing function in dirigent proteins and proteins with dirigent-like domains. J. Biol. Chem. 295, 11584–11601. doi: 10.1074/jbc.RA120.012444 32565424 PMC7450108

[B56] Meurer B. Wiermann R. Strack D. (1988). Phenylpropanoid patterns in fagales pollen and their phylogenetic relevance. Phytochemistry 27, 823–828. doi: 10.1016/0031-9422(88)84100-1

[B57] Meyer K. Cusumano J. C. Somerville C. Chapple C. C. S. (1996). Ferulate-5-hydroxylase from *Arabidopsis thaliana* defines a new family of cytochrome P450-dependent monooxygenases. Proc. Natl. Acad. Sci. U.S.A. 93, 6869–6874. doi: 10.1073/pnas.93.14.6869 8692910 PMC38900

[B58] Milkowski C. Baumert A. Strack D. (2000a). Cloning and heterologous expression of a rape cDNA encoding UDP-glucose:sinapate glucosyltransferase. Planta 211, 883–886. doi: 10.1007/s004250000411 11144274

[B59] Milkowski C. Baumert A. Strack D. (2000b). Identification of four *Arabidopsis* genes encoding hydroxycinnamate glucosyltransferases. FEBS Lett. 486, 183–184. doi: 10.1016/S0014-5793(00)02270-5 11187886

[B60] Mittasch J. Böttcher C. Frolova N. Bönn M. Milkowski C. (2014). Identification of UGT84A13 as a candidate enzyme for the first committed step of gallotannin biosynthesis in pedunculate oak (*Quercus robur*). Phytochemistry 99, 44–51. doi: 10.1016/j.phytochem.2013.11.023 24412325

[B61] Mizutani M. Ohta D. Sato R. (1997). Isolation of a cDNA and a genomic clone encoding cinnamate 4-hydroxylase from *Arabidopsis* and its expression manner *in planta* . Plant Physiol. 113, 755–763. doi: 10.1104/pp.113.3.755 9085571 PMC158193

[B62] Muir R. M. Ibañez A. M. Uratsu S. L. Ingham E. S. Leslie C. A. McGranahan G. H.. (2011). Mechanism of gallic acid biosynthesis in bacteria (*Escherichia coli*) and walnut (*Juglans regia*). Plant Mol. Biol. 75, 555–565. doi: 10.1007/s11103-011-9739-3 21279669 PMC3057006

[B63] Nedelkina S. Jupe S. C. Blee K. A. Schalk M. Werck-Reichhart D. Bolwell G. P. (1999). Novel characteristics and regulation of a divergent cinnamate 4-hydroxylase (CYP73A15) from French bean: Engineering expression in yeast. Plant Mol. Biol. 39, 1079–1090. doi: 10.1023/a:1006156216654 10380796

[B64] Niemetz R. Gross G. G. (2005). Enzymology of gallotannin and ellagitannin biosynthesis. Phytochemistry 66, 2001–2011. doi: 10.1016/j.phytochem.2005.01.009 16153405

[B65] Novaković M. Stanković M. Vučković I. Todorović N. Trifunović S. Tešević V.. (2013). Diarylheptanoids from *Alnus glutinosa* bark and their chemoprotective effect on human lymphocytes DNA. Planta Medica 79, 499–505. doi: 10.1055/s-0032-1328301 23512500

[B66] Ono N. N. Qin X. Wilson A. E. Li G. Tian L. (2016). Two UGT84 family glycosyltransferases catalyze a critical reaction of hydrolyzable tannin biosynthesis in pomegranate (*Punica granatum*). PloS One 11, e0156319. doi: 10.1371/journal.pone.0156319 27227328 PMC4882073

[B67] Pelletier M. K. Burbulis I. E. Winkel-Shirley B. (1999). Disruption of specific flavonoid genes enhances the accumulation of flavonoid enzymes and end-products in *Arabidopsis* seedlings. Plant Mol. Biol. 40, 45–54. doi: 10.1023/a:1026414301100 10394944

[B68] Qian Y. Lynch J. H. Guo L. Rhodes D. Morgan J. A. Dudareva N. (2019). Completion of the cytosolic post-chorismate phenylalanine biosynthetic pathway in plants. Nat. Commun. 10, 15. doi: 10.1038/s41467-018-07969-2 30604768 PMC6318282

[B69] Ralph S. G. Jancsik S. Bohlmann J. (2007). Dirigent proteins in conifer defense II: Extended gene discovery, phylogeny, and constitutive and stress-induced gene expression in spruce (*Picea* spp.). Phytochemistry 68, 1975–1991. doi: 10.1016/j.phytochem.2007.04.042 17590394

[B70] Ross J. Li Y. Lim E.-K. Bowles D. J. (2001). Higher plant glycosyltransferases. Genome Biol. 2, REVIEWS3004. doi: 10.1186/gb-2001-2-2-reviews3004 11182895 PMC138907

[B71] Runwal P. (2020). Climate change hits rock and roll as prized guitar wood shortage looms (Scientific American). (October 28, 2020 Letter).

[B72] Sati S. C. Sati N. Sati O. P. (2011). Bioactive constituents and medicinal importance of genus *Alnus* . Pharmacogn. Rev. 5, 174–183. doi: 10.4103/0973-7847.91115 22279375 PMC3263052

[B73] Schoch G. Goepfert S. Morant M. Hehn A. Meyer D. Ullmann P.. (2001). CYP98A3 from *Arabidopsis thaliana* is a 3´-hydroxylase of phenolic esters, a missing link in the phenylpropanoid pathway. J. Biol. Chem. 276, 36566–36574. doi: 10.1074/jbc.M104047200 11429408

[B74] Smith C. A. Want E. J. O’Maille G. Abagyan R. Siuzdak G. (2006). XCMS: Processing mass spectrometry data for metabolite profiling using nonlinear peak alignment, matching, and identification. Anal. Chem. 78, 779–787. doi: 10.1021/ac051437y 16448051

[B75] Sperschneider J. Catanzariti A.-M. DeBoer K. Petre B. Gardiner D. M. Singh K. B.. (2017). LOCALIZER: Subcellular localization prediction of both plant and effector proteins in the plant cell. Sci. Rep. 7, 44598. doi: 10.1038/srep44598 28300209 PMC5353544

[B76] Suga T. Iwata N. Asakawa Y. (1972). Chemical constituents of the male flower of *Alnus pendula* (Betulaceae). Bull. Chem. Soc Jpn. 45, 2058–2060. doi: 10.1246/bcsj.45.2058

[B77] Tanner G. J. Francki K. T. Abrahams S. Watson J. M. Larkin P. J. Ashton A. R. (2003). Proanthocyanidin biosynthesis in plants: Purification of legume leucoanthocyanidin reductase and molecular cloning of its cDNA. J. Biol. Chem. 278, 31647–31656. doi: 10.1074/jbc.M302783200 12788945

[B78] Teklehaimanot Z. Mmolotsi R. M. (2007). Contribution of red alder to soil nitrogen input in a silvopastoral system. Biol. Fertil. Soils 43, 843–848. doi: 10.1007/s00374-006-0163-9

[B79] Tenore G. C. Caruso D. Buonomo G. D’Avino M. Santamaria R. Irace C.. (2018). Annurca apple nutraceutical formulation enhances keratin expression in a human model of skin and promotes hair growth and tropism in a randomized clinical trial. J. Med. Food 21, 90–103. doi: 10.1089/jmf.2017.0016 28956697 PMC5775114

[B80] Terazawa M. Miyake M. Okuyama H. (1984). Phenolic compounds in living tissue of woods. V. Reddish orange staining in keyamahannoki (*Alnus hirsuta*) and hannoki (*A. japonica*) [Betulaceae] caused by the interaction of hirsutoside and catechol oxidase after cutting the woods. Mokuzai Gakkaishi 30, 601–607.

[B81] Teutsch H. G. Hasenfratz M. P. Lesot A. Stoltz C. Garnier J. M. Jeltsch J. M.. (1993). Isolation and sequence of a cDNA encoding the Jerusalem artichoke cinnamate 4-hydroxylase, a major plant cytochrome P450 involved in the general phenylpropanoid pathway. Proc. Natl. Acad. Sci. U.S.A. 90, 4102–4106. doi: 10.1073/pnas.90.9.4102 8097885 PMC46454

[B82] Thumuluri V. Almagro Armenteros J. J. Johansen A. R. Nielsen H. Winther O. (2022). DeepLoc 2.0: Multi-label subcellular localization prediction using protein language models. Nucleic Acids Res. 50, W228–W234. doi: 10.1093/nar/gkac278 35489069 PMC9252801

[B83] Turner N. J. Hebda R. J. (1990). Contemporary use of bark for medicine by two Salishan native elders of southeast Vancouver Island, Canada. J. Ethnopharmacol. 29, 59–72. doi: 10.1016/0378-8741(90)90098-E 2345461

[B84] Vassão D. G. Davin L. B. Lewis N. G. (2008). “Metabolic engineering of plant allyl/propenyl phenol and lignin pathways: Future potential for biofuels/bioenergy, polymer intermediates and specialty chemicals?,” in Advances in Plant Biochemistry Molecular Biology. Eds. Bohnert H. J. Nguyen H. T. (Elsevier, Oxford), 385–428. doi: 10.1016/S1755-0408(07)01013-2

[B85] Vassão D. G. Kim S.-J. Milhollan J. K. Eichinger D. Davin L. B. Lewis N. G. (2007). A pinoresinol-lariciresinol reductase homologue from the creosote bush (*Larrea tridentata*) catalyzes the efficient in *vitro* conversion of *p*-coumaryl/coniferyl alcohol esters into the allylphenols chavicol/eugenol, but not the propenylphenols *p*-anol/isoeugenol. Arch. Biochem. Biophys. 465, 209–218. doi: 10.1016/j.abb.2007.06.002 17624297

[B86] Vera Alvarez R. Pongor L. S. Mariño-Ramirez L. Landsman D. (2019). TPMCalculator: One-step software to quantify mRNA abundance of genomic features. Bioinformatics 35, 1960–1962. doi: 10.1093/bioinformatics/bty896 30379987 PMC6546121

[B87] Westfall C. S. Xu A. Jez J. M. (2014). Structural evolution of differential amino acid effector regulation in plant chorismate mutases. J. Biol. Chem. 289, 28619–28628. doi: 10.1074/jbc.M114.591123 25160622 PMC4192511

[B88] WFO (2022). *Alnus* Mill. Available online at: http://www.worldfloraonline.org/taxon/wfo-4000001331 (Accessed 04 August 2022).

[B89] Xia Z.-Q. Costa M. A. Pélissier H. C. Davin L. B. Lewis N. G. (2001). Secoisolariciresinol dehydrogenase purification, cloning and functional expression: Implications for human health protection. J. Biol. Chem. 276, 12614–12623. doi: 10.1074/jbc.M008622200 11278426

[B90] Xie D. Y. Sharma S. B. Paiva N. L. Ferreira D. Dixon R. A. (2003). Role of anthocyanidin reductase, encoded by *BANYULS* in plant flavonoid biosynthesis. Science 299, 396–399. doi: 10.1126/science.1078540 12532018

[B91] Yonekura-Sakakibara K. Yamamura M. Matsuda F. Ono E. Nakabayashi R. Sugawara S.. (2021). Seed-coat protective neolignans are produced by the dirigent protein AtDP1 and the laccase AtLAC5 in *Arabidopsis* . Plant Cell 33, 129–152. doi: 10.1093/plcell/koaa014 33751095 PMC8136895

[B92] Zhang R. Yu Q. Lu W. Shen J. Zhou D. Wang Y.. (2019). Grape seed procyanidin B2 promotes the autophagy and apoptosis in colorectal cancer cells via regulating PI3K/Akt signaling pathway. OncoTargets Ther. 12, 4109–4118. doi: 10.2147/OTT.S195615 PMC6538883 31213831

[B93] Zhou B. Alania Y. Reis M. Jing S.-X. McAlpine J. B. Bedran-Russo A. K.. (2022). *Seco* B-type oligomers from *Pinus massoniana* expand the procyanidin chemical space and exhibit dental bioactivity. J. Nat. Prod. 85, 2753–2768. doi: 10.1021/acs.jnatprod.2c00664 36382951 PMC9789173

