# Supplementary material for: RNA-seq and metabolomic analyses of beneficial plant phenol biochemical pathways in red alder
Source: Front Plant Sci. 2024 Nov 7;15:1349635. doi: 10.3389/fpls.2024.1349635 (PMC11578710; doi:10.3389/fpls.2024.1349635)
Supplement: Supplementary file 2 [file Presentation1.pdf]

frontiers

Supplementary Material

1 Supplementary Tables

Supplementary Table S1. NCBI genome assembly accession numbers and sources of the peptide annotations used for other Fagales species.

Species	Abbreviation	NCBI assembly	Source of peptide annotations
Alnus glutinosa	Alngl	GCA_003254965.1	http://gigadb.org/dataset/101042
Betula pendula	Bpev	GCA_900184695.1	https://www.hardwoodgenomics.org/Genome-assembly/2986212
Casuarina glauca	Casgl	GCA_003255045.1	http://gigadb.org/dataset/101051
Corylus avellana	Corav	GCA_901000735.1	https://www.hardwoodgenomics.org/Genome-assembly/3472010
Fagus sylvatica	FSB	GCA_003347535.1	https://www.hardwoodgenomics.org/Genome-assembly/2365974
Juglans nigra	Juni	GCA_002916485.1	https://www.hardwoodgenomics.org/Genome-assembly/3641871
Quercus robur	Qrob	GCA_900291515.1	http://www.oakgenome.fr/?page_id=587

Supplementary Table S2. Metabolites identified and/or provisionally annotated in red alder tissues. P, Phenolic acid; O, Organic acid; G, Gallic acid derivative; E, Ellagitannin; F, Flavonoid; Pa, Proanthocyanidin; D, Diarylheptanoid; Po, Polyamine. Pl, Pollen, Ca, Catkins.

Feature	RT (min)	Cate-gory	Identification/Putative annotation	Molecular formulae	Calculated mass [M-H]$^-$	ppm error	Nodules			Roots			Stems			Buds			Leaves		Pl	Ca
							Sp	Su	Fa	Sp	Su	Fa	Sp	Su	Fa	Sp	Su	Fa	Su	Fa	Su	Su
M191T128	2.13	O	Quinic acid[a]	$C_7H_{12}O_6$	191.0556	0.13	√	√	√	√	√	√	√	√	√	√	√	√	√	√	√	√
M173T157	2.62	O	Shikimic acid[a]	$C_7H_{10}O_5$	173.0450	1.10	√	√	√	√	√	√	√	√	√	√	√	√	√	√	√	√
M191T299	4.98	O	Citric acid[a]	$C_6H_8O_7$	191.0192	0.16	√	√	√	√	√	√	√	√	√	√	√	√	√	√	√	√
M331T342	5.7	G	*β*-Glucogallin[a]	$C_{13}H_{16}O_{10}$	331.0665	0.51	√	√	√	√	√	√	√	√	√	√	√	√	√	√	√	√
M783T489	8.15	E	Pedunculagin (α or β anomer)[a]	$C_{34}H_{24}O_{22}$	783.0681	1.00	ND	ND	ND	√	√	√	ND	√	√	ND	ND	ND	√	√	ND	√
M783T581	9.68	E	Pedunculagin (α or β anomer)[a]	$C_{34}H_{24}O_{22}$	783.0681	1.04	ND	ND	ND	√	√	√	ND	√	√	ND	ND	ND	√	√	ND	√
M483T594	9.90	G	Digalloyl glucose[b,c]	$C_{20}H_{20}O_{14}$	483.0775	4.14	ND	ND	ND	ND	ND	ND	ND	ND	ND	ND	ND	ND	ND	ND	ND	ND
M785T626	10.43	E	Tellimagrandin I (α or β anomer)[a,d]	$C_{34}H_{26}O_{22}$	785.0838	1.27	ND	ND	ND	√	√	√	√	√	√	ND	ND	ND	√	√	ND	√
M353T634	10.57	P	*trans*-Chlorogenic acid[a]	$C_{16}H_{18}O_9$	353.0873	0.29	√	√	√	√	√	√	√	√	√	√	√	√	√	√	√	√
M633T645	10.75	E	Strictinin[a]	$C_{27}H_{22}O_{18}$	633.0728	0.76	ND	√	√	√	√	√	√	√	√	ND	√	ND	√	√	ND	√
M289T647	10.78	F	(+)-Catechin[a]	$C_{15}H_{14}O_6$	289.0712	0.83	√	√	√	√	√	√	√	√	√	√	√	√	√	√	ND	√
M325T650	10.83	P	*cis*-*p*-Coumaric acid glucoside[a]	$C_{15}H_{18}O_8$	325.0923	1.54	ND	ND	ND	ND	ND	ND	ND	ND	ND	ND	ND	ND	ND	ND	ND	ND
M353T659	10.98	P	Cryptochlorogenic acid (4-*O*-Caffeoyl quinic acid)[a]	$C_{16}H_{18}O_9$	353.0873	0.43	√	√	√	√	ND	√	√	√	√	√	√	√	√	√	√	√
M577T688	11.47	Pa	Procyanidin B2[a]	$C_{30}H_{26}O_{12}$	577.1346	0.07	√	√	√	√	√	√	√	√	√	√	√	√	√	√	ND	√
M785T701	11.68	E	Tellimagrandin I (α or β anomer)[a]	$C_{34}H_{26}O_{22}$	785.0838	0.55	ND	√	ND	√	√	√	√	√	√	√	ND	ND	ND	√	ND	√
M337T720	12.00	P	*cis*-4-*O*-*p*-Coumaroyl quinic acid[c]	$C_{16}H_{18}O_8$	337.0923	1.57	√	√	√	√	√	√	√	√	√	√	√	√	√	√	√	√
M635T729	12.15	G	Trigalloyl glucose[b,d]	$C_{27}H_{24}O_{18}$	635.0884	4.41	ND	ND	ND	ND	ND	ND	ND	ND	ND	ND	ND	ND	ND	ND	ND	ND
M289T734	12.23	F	(–)-Epicatechin[a]	$C_{15}H_{14}O_6$	289.0712	0.35	√	√	√	√	√	√	√	√	√	√	√	√	√	√	ND	√
M337T739	12.31	P	*trans*-3-*O*-*p*-Coumaroyl quinic acid[a,b,c,d]	$C_{16}H_{18}O_8$	337.0923	0.59	ND	ND	ND	ND	ND	ND	ND	ND	ND	ND	ND	ND	ND	ND	ND	ND
M337T748	12.47	P	*trans*-4-*O*-*p*-Coumaroyl quinic acid[c]	$C_{16}H_{18}O_8$	337.0923	0.09	√	√	√	√	√	√	√	√	√	√	√	√	√	√	√	√
M865T759	12.65	Pa	Pa trimer[c]	$C_{45}H_{38}O_{18}$	865.1980	0.31	√	√	√	√	√	√	√	√	√	√	√	√	√	√	ND	√
M625T776	12.93	F	Dihexosylquercetin[c]	$C_{27}H_{30}O_{17}$	625.1405	0.87	ND	ND	ND	ND	ND	ND	ND	ND	ND	ND	ND	ND	ND	ND	√	√
M367T782	13.03	P	4-*O*-Feruloyl quinic acid[c]	$C_{17}H_{20}O_9$	367.1029	0.01	√	√	√	√	√	√	√	√	√	√	√	√	√	√	√	√

Feature	RT (min)	Cate-gory	Identification/Putative annotation	Molecular formulae	Calculated mass [M-H]$^-$	ppm error	Nodules			Roots			Stems			Buds			Leaves		Pl	Ca
							Sp	Su	Fa	Sp	Su	Fa	Sp	Su	Fa	Sp	Su	Fa	Su	Fa	Su	Su
M935T791	13.18	E	Casuarinin[a]	$C_{41}H_{28}O_{26}$	935.0791	0.88	ND	ND	√	√	√	√	ND	√	√	ND	ND	ND	√	√	ND	√
M337T818	13.63	P	*cis*-3-*O*-*p*-Coumaroyl quinic acid[c]	$C_{16}H_{18}O_8$	337.0923	0.41	√	√	ND	√	√	ND	√	√	√	√	√	√	√	√	√	√
M615T827	13.78	F	Quercetin-*O*-(*O*-galloyl)-hexoside[c]	$C_{28}H_{24}O_{16}$	615.0986	0.90	ND	ND	ND	ND	√	ND	√	√	√	√	√	√	√	√	√	√
M937T835	13.92	E	Tellimagrandin II[a,b,d]	$C_{41}H_{30}O_{26}$	937.0947	1.71	ND	ND	ND	ND	ND	ND	ND	ND	ND	ND	ND	ND	ND	ND	ND	ND
M436T839	13.98	Po	N^1,N^{10}-bis(*p*-Coumaroyl) spermidine[a]	$C_{25}H_{31}N_3O_4$	436.2236	0.03	ND	ND	ND	ND	ND	ND	ND	ND	ND	ND	ND	ND	ND	ND	√	√
M615T845	14.08	F	Quercetin-*O*-(*O*-galloyl)-hexoside[c]	$C_{28}H_{24}O_{16}$	615.0986	1.51	ND	ND	ND	√	√	ND	√	√	√	√	√	√	√	√	√	√
M436T872	14.53	Po	N^1,N^{10}-bis(*p*-Coumaroyl) spermidine[c] (isomer)	$C_{25}H_{31}N_3O_4$	436.2236	1.01	ND	ND	ND	ND	ND	ND	ND	ND	ND	ND	ND	ND	ND	ND	√	√
M507T884	14.73	D	Hirsutanonol 5-*O*-glucoside[c]	$C_{25}H_{32}O_{11}$	507.1866	0.88	√	√	√	√	√	√	√	√	√	√	√	√	√	√	ND	√
M463T892	14.87	F	Quercetin-3-*O*-glucoside (isoquercitrin)[a]	$C_{21}H_{20}O_{12}$	463.0877	0.35	√	√	√	√	√	√	√	√	√	√	√	√	√	ND	√	√
M477T895	14.92	F	Quercetin glucuronide[c]	$C_{21}H_{18}O_{13}$	477.0669	0.23	√	√	√	ND	√	√	√	√	√	√	√	√	√	√	√	√
M939T920	15.33	G	Pentagalloyl glucose[a,b]	$C_{41}H_{32}O_{26}$	939.1104	1.49	ND	ND	ND	ND	ND	ND	ND	ND	ND	ND	ND	ND	ND	ND	ND	ND
M477T932	15.53	D	Oregonin[a]	$C_{24}H_{30}O_{10}$	477.1761	0.53	√	√	√	√	√	√	√	√	√	√	√	√	√	√	ND	√
M491T959	15.98	D	1-(3,4-Dihydroxyphenyl)-7-hydroxylphenyl-heptane-3-one 5-*O*-*β*-D-glucopyranoside[c]	$C_{25}H_{32}O_{10}$	491.1917	0.37	√	√	√	√	√	√	√	√	√	√	√	√	√	√	ND	√
M447T965	16.08	F	Kaempferol-3-*O*-glucoside[a,b]	$C_{21}H_{20}O_{11}$	447.0927	1.25	ND	ND	ND	ND	ND	ND	ND	ND	ND	ND	ND	ND	ND	ND	√	√
M491T968	16.13	D	1-Hydroxylphenyl-7-(3,4-dihydroxyphenyl)-heptane-3-one 5-*O*-*β*-D-glucopyranoside[c]	$C_{25}H_{32}O_{10}$	491.1917	0.25	√	√	√	√	√	√	√	√	√	√	√	√	√	√	ND	√
M447T974	16.23	F	Quercetin-rhamnoside (quercitrin)[a,c]	$C_{21}H_{20}O_{11}$	447.0927	0.24	√	√	√	√	√	√	√	√	√	√	√	√	√	√	√	√
M629T977	16.28	D	Galloyl oregonin like[c]	$C_{31}H_{32}O_{14}$	629.1870	0.82	√	√	√	√	√	√	√	√	√	ND	√	ND	√	√	ND	√
M345T1006	16.77	D	Hirsutanonol[a]	$C_{19}H_{22}O_6$	345.1338	0.46	√	√	√	√	√	√	√	√	√	√	√	√	√	√	√	√
M461T1018	16.96	D	Alnuside A[a,d]	$C_{24}H_{30}O_9$	461.1812	0.09	ND	ND	ND	ND	ND	ND	ND	ND	ND	ND	ND	ND	ND	ND	ND	ND
M625T1019	16.98	D	Rubranoside D[c]	$C_{30}H_{42}O_{14}$	625.2496	1.16	√	√	√	√	√	√	√	√	√	√	√	√	√	√	ND	√
M629T1022	17.03	D	Galloyl oregonin like[c]	$C_{31}H_{34}O_{14}$	629.1870	0.38	√	√	√	√	√	√	√	√	√	√	√	√	√	√	ND	√
M461T1028	17.13	D	Alnuside B[a]	$C_{24}H_{30}O_9$	461.1812	0.08	√	√	√	√	√	√	√	√	√	√	√	√	√	√	ND	√

Feature	RT (min)	Cate-gory	Identification/Putative annotation	Molecular formulae	Calculated mass $[M-H]^-$	ppm error	Nodules			Roots			Stems			Buds			Leaves		Pl	Ca
							Sp	Su	Fa	Sp	Su	Fa	Sp	Su	Fa	Sp	Su	Fa	Su	Fa	Su	Su
M475T1045	17.42	D	Platyphylloside[a]	$C_{25}H_{32}O_9$	475.1968	0.63	√	√	√	√	√	√	√	√	√	√	√	√	√	√	ND	√
M431T1060	17.67	F	Kaempferol-3-rhamnoside (afzelin)[c]	$C_{21}H_{20}O_{10}$	431.0978	0.28	√	√	√	√	√	√	√	√	√	√	√	√	√	√	√	√
M493T1066	17.77	D	Rubranoside A[a]	$C_{25}H_{34}O_{10}$	493.2074	0.50	√	√	√	√	√	√	√	√	√	√	√	√	√	√	ND	√
M629T1073	17.88	D	Galloyl oregonin like[c]	$C_{31}H_{34}O_{14}$	629.1870	0.84	√	√	√	√	√	√	√	√	√	√	√	√	√	√	ND	√
M625T1099	18.32	D	Rubranoside C[c]	$C_{30}H_{42}O_{14}$	625.2496	0.64	√	√	√	√	√	√	√	√	√	√	√	√	√	√	ND	√
M463T1117	18.62	D	Rubranoside B[a]	$C_{24}H_{32}O_9$	463.1968	0.14	√	√	√	√	√	√	√	√	√	√	√	√	√	√	ND	√
M445T1120	18.67	D	Platyphyllonol 5-*O*-D-xylopyranoside[c]	$C_{24}H_{30}O_8$	445.1862	0.45	√	√	√	√	√	√	√	√	√	√	√	√	√	√	ND	√
M577T1180	19.67	D	Butanoyl hirsutanonol 5-*O*-β-D-glucopyranoside[c]	$C_{29}H_{38}O_{12}$	577.2285	0.57	√	√	√	√	√	√	√	√	√	√	√	√	√	√	ND	√
M623T1192	19.87	D	$2'''$-*O*-*p*-Coumaroyl oregonin[a]	$C_{33}H_{36}O_{12}$	623.2129	0.26	√	√	√	√	√	√	√	√	√	√	√	√	√	√	ND	√
M285T1214	20.23	F	Luteolin[a]	$C_{15}H_{10}O_6$	285.0399	0.10	√	√	√	√	√	√	√	√	√	√	√	√	√	√	√	√
M547T1233	20.55	D	Butanoyl oregonin[c]	$C_{28}H_{36}O_{11}$	547.2179	0.39	√	√	√	ND	√	ND	√	√	√	√	√	√	√	√	ND	√
M461T1240	20.67	D	Aceroside VII[c]	$C_{25}H_{34}O_8$	461.2175	0.47	√	√	√	ND	√	ND	√	√	√	√	√	√	√	√	ND	√
M591T1264	21.07	D	Methylbutanol hirsutanonol 5-*O*-β-D-glucopyranoside[c]	$C_{30}H_{40}O_{12}$	591.2442	1.19	√	√	√	√	√	√	√	√	√	√	√	√	√	√	ND	√
M582T1274	21.23	Po	Tri-*p*-coumaroyl spermidine[c]	$C_{34}H_{37}N_3O_6$	582.2604	1.03	ND	ND	ND	ND	ND	ND	ND	ND	ND	ND	ND	ND	ND	ND	√	√
M593T1284	21.40	D	1,7-bis(4-Hydroxyphenyl)-3-heptanol 3-*O*-β-D-glucopyranosyl-(1→3)-β-D-xylopyranoside[c]	$C_{30}H_{42}O_{12}$	593.2598	0.32	√	√	√	√	√	√	√	√	√	√	√	√	√	√	√	√
M561T1321	22.02	D	Alnuside C[c]	$C_{29}H_{38}O_{11}$	561.2336	0.43	√	√	√	ND	ND	ND	√	√	√	√	√	√	√	√	ND	√
M431T1323	22.05	D	1,7-bis(4-Hydroxyphenyl)-3-heptanol 3-*O*-β-D-xylopyranoside[c]	$C_{24}H_{32}O_7$	431.2070	0.73	√	√	√	√	√	√	√	√	√	√	√	√	√	√	√	√
M269T1353	22.55	F	Apigenin[a]	$C_{15}H_{10}O_5$	269.0450	0.11	√	√	√	√	√	√	√	√	√	√	√	√	ND	√	√	√
M575T1366	22.77	D	1-(3,4-Dihydroxyphenyl)-7-hydroxyphenyl-heptane-3-one methylbutanoyl 5-*O*-β-D-glucopyranoside[c]	$C_{30}H_{40}O_{11}$	575.2492	0.61	√	√	√	ND	√	√	√	√	√	√	√	√	√	√	√	√
M637T1394	23.23	D	Cinnamoyl hirsutanonol 5-*O*-β-glucopyranoside[c]	$C_{34}H_{38}O_{12}$	637.2285	0.86	√	√	√	√	√	√	√	√	√	√	√	√	√	√	ND	√
M345T1416	23.60	F	Viscidulin III[c]	$C_{17}H_{14}O_8$	345.0610	0.60	ND	ND	ND	ND	√	ND	√	√	ND	√	√	√	√	√	√	√
M283T1673	27.88	F	7-*O*-Methylapigenin (genkwanin)[a]	$C_{16}H_{12}O_5$	283.0607	0.15	ND	ND	ND	ND	ND	ND	√	√	ND	√	√	√	√	√	√	√

Feature	RT (min)	Cate-gory	Identification/Putative annotation	Molecular formulae	Calculated mass [M-H]$^-$	ppm error	Nodules			Roots			Stems			Buds			Leaves		Pl	Ca
							Sp	Su	Fa	Sp	Su	Fa	Sp	Su	Fa	Sp	Su	Fa	Su	Fa	Su	Su
M313T1679	27.98	F	Dimethoxyluteolin[c]	$C_{17}H_{14}O_6$	313.0712	0.02	ND	ND	ND	ND	ND	ND	√	√	ND	√	√	√	√	√	√	√
M329T1686	28.10	F	Unidentified flavonoid	$C_{17}H_{14}O_7$	329.0661	0.60	ND	ND	ND	ND	ND	ND	√	√	ND	√	√	√	ND	√	√	√

a = confirmed using authentic standards, including proanthocyanidins (procyanidin B2), diarylheptanoids (alnusides A and B, hirsutanonol, oregonin, 2‴-*O*-*p*-coumaroyl oregonin, platyphylloside and rubranosides A and B), gallic acid derivatives and ellagitannin related [*β*-glucogallin, pentagalloyl glucose, pedunculagin (α or β anomers), tellimagrandin I (α or β anomers), strictinin, casuarinin, tellimagrandin II], polyamine [N^1,N^{10}-bis(*p*-coumaroyl) spermidine], flavonoids [(+)-catechin, (–)-epicatechin, quercetin-3-*O*-glucoside, quercitrin, genkwanin, kaempferol-3-*O*-glucoside, luteolin and apigenin], phenolic acids/derivatives (*trans*-chlorogenic, 4-*O*-caffeoyl quinic and *trans*-3-*O*-*p*-coumaroyl quinic acids, as well as *cis*-4-*O*-*p*-coumaric acid glucoside), organic acids (citric, shikimic, and quinic acids).
b = metabolite with lower detection limit (trace)
c = provisionally annotated by analysis of mass spectrometric fragmentation patterns and comparison to literature reports such as by Sati et al. (1), Jackrel (2), and Jackrel et al. (3).
d = several known alder metabolites were not detected in these Clone 639 tissues.

Supplementary Table S3. Red alder homologs to *bona fide* shikimate, chorismate, phenylalanine, and *β*-glucogallin pathway genes.

Enzyme	Plant species	NCBI accession number	*A. rubra* homologs	Name	Amino acid identity (%)*	Predicted localization**
3-Deoxy-D-arabinoheptulosonate 7-phosphate synthase (DAHPS)	*Arabidopsis thaliana*	AAA32784 (4)	ALNRU00118289	ArDAHPS1	80.5	Plastid
			ALNRU00091501	ArDAHPS2	80.4	Plastid
			ALNRU00046501	ArDAHPS3	77.6	Plastid
			ALNRU00044315	ArDAHPS4	74.2	Plastid
			ALNRU00084407	ArDAHPS5	72.8	Plastid
3-Dehydroquinate synthase (DHQS)	*Solanum lycopersicum*	AAL77575 (5)	ALNRU00027821	ArDHQS	76.8	Plastid
Bifunctional 3-dehydroquinate dehydratase/shikimate dehydrogenase (DHQD-SDH)	*A. thaliana*	Q9SQT8 (6)	ALNRU00075273	ArDHQD-SDH1	73.2	Cytoplasm
			ALNRU00016551	ArDHQD-SDH2	50.6	Cytoplasm
			ALNRU00016553	ArDHQD-SDH3	64.1	Cytoplasm
			ALNRU00016554	ArDHQD-SDH4	63.6	Cytoplasm
			ALNRU00118494	ArDHQD-SDH5	47.8	Cytoplasm
			ALNRU00050250	ArDHQD-SDH6	53.3	Cytoplasm
Shikimate kinase (SK)	*S. lycopersicum*	NP_001234112 (7)	ALNRU00033270	ArSK	50.4	Plastid
5-Enolpyruvylshikimate-3-phosphate synthase (EPSPS)	*A. thaliana*	P05466 (8)	ALNRU00062892	ArEPSPS	73.3	Plastid
Chorismate synthase (CS)	*Capnoides sempervirens*	CAA43034 (9)	ALNRU00062533	ArCS	76.1	Plastid
Chorismate mutase (CM)	*A. thaliana*	CAB54518 (10)	ALNRU00054560	ArCM1	70.8	Plastid
			ALNRU00061145	ArCM2	49.1	Cytoplasm
			ALNRU00010637	ArCM3	61.4	Plastid
Prephenate amino-transferase (PPA-AT)	*A. thaliana*	ADM67558 (11)	ALNRU00090523	ArPPA-AT	71.9	Plastid
Arogenate dehydratase (ADT)	*A. thaliana*	At5g22630 (AtADT5) (12)	ALNRU00051159	ArADT1	73.3	Plastid
			ALNRU00103384	ArADT2	53.4	Plastid
			ALNRU00048921	ArADT3	52.5	Plastid
β-glucogallin forming UDP glucosyltransferase (UGT)	*Quercus robur*	AHA54051 (UGT84A13) (13)	ALNRU00005258	ArUGT1	87.1	Cytoplasm
			ALNRU00139344	ArUGT2	87.2	Cytoplasm
			ALNRU00005266	ArUGT3	71.7	Cytoplasm
			ALNRU00126768	ArUGT4	71.1	Cytoplasm
			ALNRU00005272	ArUGT5	70.6	Cytoplasm

*used Clustal Omega Multi Sequence Alignment Tool (14).

**used LOCALIZER 1.0.4 (15) and DeepLoc 2.0 (16).

Supplementary Table S4. Red alder homologs to *bona fide* phenylpropanoid pathway genes.

Enzyme	Plant species	NCBI accession number	*A. rubra* homologs	Name	Amino acid identity (%)*
Phenylalanine ammonia lyase (PAL)	*Arabidopsis thaliana*	At2g37040 (AtPAL1) (17)	ALNRU00113829	ArPAL1	83.9
			ALNRU00062591	ArPAL2	82.2
			ALNRU00103226	ArPAL3	81.8
			ALNRU00062587	ArPAL4	80.8
Cinnamate 4-hydroxylase (C4H)	*Helianthus tuberosus*	CYP73A1, CAA78982 (18)	ALNRU00092227	ArC4H1	89.1
			ALNRU00122270	ArC4H2	62.0
			ALNRU00028961	ArC4H3	62.2
Hydroxycinnamoyl CoA: shikimate hydroxycinnamoyl transferase (HCT)	*Nicotiana tabacum*	CAD47830 (NtHCT) (19)	ALNRU00070014	ArHCT1	79.5
			ALNRU00070002	ArHCT2	76.0
			ALNRU00069972	ArHCT3	75.3
p-Coumarate 3-hydroxylase (C3H)	*A. thaliana*	CYP98A3, At2g40890 (20)	ALNRU00003369	ArC3H1	82.3
			ALNRU00003318	ArC3H2	77.4
			ALNRU00003289	ArC3H3	74.6
			ALNRU00138548	ArC3H4	74.6
4-Coumarate CoA ligase (4CL)	*A. thaliana*	At1g51680 (At4CL1) (21)	ALNRU00130727	Ar4CL1	69.8
			ALNRU00011055	Ar4CL2	69.6
			ALNRU00087439	Ar4CL3	70.3
			ALNRU00090869	Ar4CL4	60.3
Ferulate 5-hydroxylase (F5H)	*A. thaliana*	CYP84A1, At4g36220 (22)	ALNRU00027975	ArF5H	62.3
Caffeic acid *O*-methyltransferase (COMT)	*A. thaliana*	At5g54160 (AtCOMT1) (23, 24)	ALNRU00041884	ArCOMT1	78.5
			ALNRU00090858	ArCOMT2	62.3
Caffeoyl CoA *O*-methyltransferase (CCOMT)	*Petroselinum crispum*	AAA33851 (25)	ALNRU00005083	ArCCOMT1	78.6
			ALNRU00005087	ArCCOMT2	71.8
			ALNRU00025885	ArCCOMT3	58.0
Cinnamoyl CoA reductase (CCR)	*A. thaliana*	At1g15950 (AtCCR1) (26)	ALNRU00069900	ArCCR1	78.9
			ALNRU00065491	ArCCR2	50.3
Cinnamyl alcohol dehydrogenase (CAD)	*A. thaliana*	At4g34230 (AtCAD5) (27)	ALNRU00005245	ArCAD1	79.3
			ALNRU00011316	ArCAD2	62.9
			ALNRU00085651	ArCAD3	50.1
			ALNRU00028036	ArCAD4	47.6
			ALNRU00048433	ArCAD5	49.2
			ALNRU00061461	ArCAD6	48.3
			ALNRU00085657	ArCAD7	48.5
			ALNRU00085652	ArCAD8	48.7
			ALNRU00061417	ArCAD9	47.5
			ALNRU00061363	ArCAD10	47.9

*used Clustal Omega Multi Sequence Alignment Tool (14).

Supplementary Table S5. Red alder homologs to *bona fide* flavonoid pathway genes.

Enzyme	Plant species	NCBI accession number	*A. rubra* homologs	Name	Amino acid identity (%)*
Chalcone synthase (CHS)	*Antirrhinum majus*	CAA27338 (28)	ALNRU00052590	ArCHS1	86.1
			ALNRU00080687	ArCHS2	73.9
			ALNRU00080685	ArCHS3	86.2
			ALNRU00080658	ArCHS4	72.7
Chalcone isomerase (CHI)	*Petunia × hybrida*	CAA32730 (29)	ALNRU00071279	ArCHI	70.5
Flavanone 3-hydroxylase (F3H)	*Arabidopsis thaliana*	AAC49176 (30)	ALNRU00027879	ArF3H	84.6
Flavonoid 3′-hydroxylase (F3′H)	*A. thaliana*	At5g07790 (31)	ALNRU00072988	ArF3′H1	71.3
			ALNRU00026572	ArF3′H2	71.2
Flavonoid 3′,5′-hydroxylase (F3′,5′H)	*P. hybrida*	CAA80266 (32)	ALNRU00009602	ArF3′,5′H	76.8
Flavonol synthase (FLS)	*P. hybrida*	CAA80264 (33)	ALNRU00076204	ArFLS1	73.9
			ALNRU00037056	ArFLS2	67.5
Dihydroflavonol 4-reductase (DFR)	*A. majus*	P14721 (34)	ALNRU00000326	ArDFR1	69.4
			ALNRU00000329	ArDFR2	62.2
Leucoanthocyanidin reductase (LAR)	*Desmodium uncinatum*	CAD79341 (35)	ALNRU00034769	ArLAR1	66.2
			ALNRU00056862	ArLAR2	58.1
Leucoanthocyanidin dioxygenase (LDOX) also called anthocyanidin synthase (ANS)	*A. thaliana*	Q96323 (36)	ALNRU00013023	ArLDOX	79.9
Anthocyanidin reductase (ANR)	*Medicago truncatula*	AAN77735 (37)	ALNRU00124301	ArANR1	76.6
			ALNRU00012650	ArANR2	74.9
Flavone synthase II (FNS II)	*Gerbera* hybrid cultivar	AAD39549 (CYP93B2) (38)	ALNRU00042823	ArFNSII-1	57.1
			ALNRU00004682	ArFNSII-2	51.3
			ALNRU00004681	ArFNSII-3	51.4
			ALNRU00004684	ArFNSII-4	48.5

*used Clustal Omega Multi Sequence Alignment Tool (14).

Supplementary Table S6. Red alder homologs to *bona fide* dirigent protein genes

Enzyme	Plant species	NCBI accession number	*A. rubra* homologs	Name	Amino acid identity (%)*
Dir-a sub-family	*Pisum sativum*	P13240 (DRR206) (39)	ALNRU00062277	ArDIR1	73.2
			ALNRU00062278	ArDIR2	62.0
			ALNRU00062271	ArDIR3	51.2
Dir-b sub-family	*P. sativum*	PsCam039127<br>6OOD (PsPTS1) (40)	ALNRU00004390	ArDIR4	55.7
			ALNRU00004385	ArDIR5	53.3
			ALNRU00004380	ArDIR6	53.8
			ALNRU00004378	ArDIR7	54.4
			ALNRU00091352	ArDIR8	49.7
			ALNRU00091353	ArDIR9	48.4
			ALNRU00091336	ArDIR10	46.7
			ALNRU00135575	ArDIR11	46.7
			ALNRU00091354	ArDIR12	45.4
			ALNRU00091345	ArDIR13	43.2
			ALNRU00113764	ArDIR15	46.0
			ALNRU00116248	ArDIR21	49.2
Dir-d sub-family	*Gossypium hirsutum*	GhDIR4 (41)	ALNRU00093085	ArDIR14	38.1
			ALNRU00093034	ArDIR16	44.6
			ALNRU00065374	ArDIR17	46.3
			ALNRU00093041	ArDIR20	46.0
			ALNRU00065375	ArDIR22	41.1
			ALNRU00065377	ArDIR25	44.6
			ALNRU00123362	ArDIR27	38.1
Dir-e sub-family	*Arabidopsis thaliana*	At2g28670 (AtDIR10) (42)	ALNRU00042827	ArDIR18	60.2
			ALNRU00071249	ArDIR19	61.1
			ALNRU00071250	ArDIR23	41.8
			ALNRU00052656	ArDIR24	40.9
			ALNRU00071251	ArDIR26	36.4

*used Clustal Omega Multi Sequence Alignment Tool (14).

Supplementary Table S7. Sequence comparisons (percent identity) of chalcone synthases (CHS), curcumin synthase (CURS) and diketide CoA synthase (DCS).

	AmCHS*	ClDCS	ClCURS	ArCHS1	ArCHS2	ArCHS3
ClDCS	65.8					
ClCURS1	60.2	62.6				
ArCHS1	86.1	65.5	60.7			
ArCHS2	73.9	62.5	55.3	74.8		
ArCHS3	86.2	65.8	59.4	89.5	76.5	
ArCHS4	72.7	61.2	48.8	72.7	91.9	74.6

*AmCHS, *Antirrhinum majus* CHS (CAA27338); ClCURS1, *Curcuma longa* CURS (BAH56226); ClDCS, *C. longa* DCS (BAH56225); ArCHS1 – ArCH3, putative *Alnus rubra* CHS (ALNRU00052590, ALNRU00080687, ALNRU00080685)

Supplementary Table S8. Red alder homologs *bona fide* pinoresinol-lariciresinol reductase, allylphenol synthase, propenylphenol synthase, and secoisolariciresinol dehydrogenase genes.

Enzyme	Plant species	NCBI accession number	*A. rubra* homologs	Name	Amino acid identity (%)*
Pinoresinol-lariciresinol reductase (PLR)	*Forsythia intermedia*	AAC49608 (FiPLR1) (43)	ALNRU00108755	ArPLR1	77.5
Allylphenol synthase (APS)	*Larrea tridendata*	AHA90804 (LtAPS1) (44)	ALNRU00041001	ArAPS1	81.8
			ALNRU00117167	ArAPS2	81.8
			ALNRU00040994	ArAPS3	77.0
			ALNRU00041004	ArAPS4	77.0
			ALNRU00117169	ArAPS5	77.0
Propenylphenol synthase (PPS)	*L. tridendata*	AHA90806 (LtPPS1) (44)	ALNRU00072637	ArPPS1	64.4
			ALNRU00072634	ArPPS2	63.1
Secoisolariciresinol dehydrogenase (SDH)	*Podophyllum peltatum*	AF352734 (PpSDH) (45)	ALNRU00043749	ArSDH1	56.2
			ALNRU00043779	ArSDH2	56.2
			ALNRU00043753	ArSDH3	55.8
			ALNRU00043507	ArSDH4	56.0
			ALNRU00043783	ArSDH5	53.5
			ALNRU00049317	ArSDH6	53.4
			ALNRU00043813	ArSDH7	50.2
			ALNRU00049319	ArSDH8	54.1

*used Clustal Omega Multi Sequence Alignment Tool (14).

2 Supplementary Figures

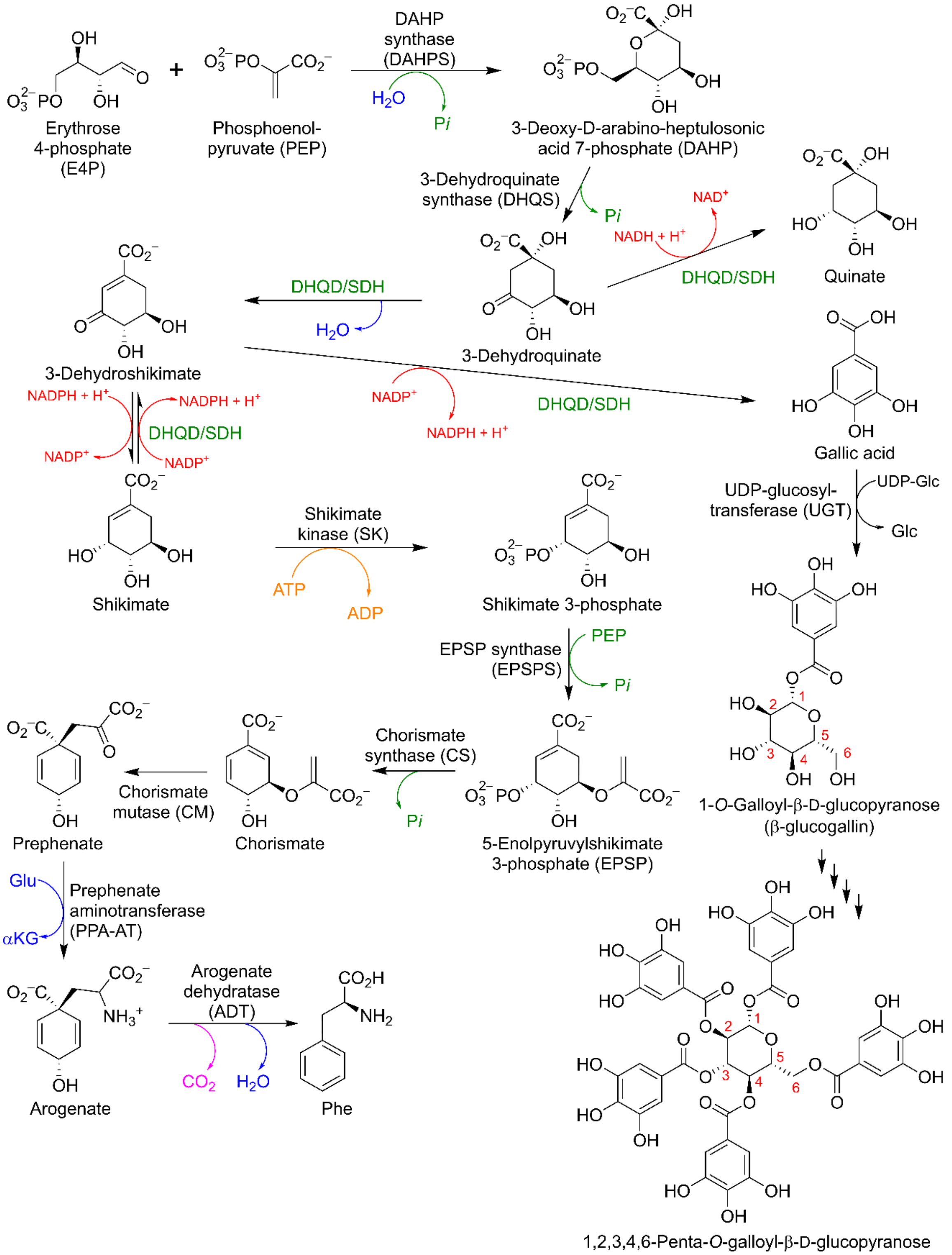

Supplementary Figure S1. Current simplified shikimate-chorismate pathway and offshoots to phenylalanine, quinate, *β*-glucogallin, and 1,2,3,4,6-penta-*O*-galloyl-*β*-D-glucopyranose. DHQD-SDH: bifunctional 3-dehydroquinate dehydratase/shikimate dehydrogenase.

```
AtDAHPS     1 MALSNASSLSTRSIYGGDLSHRPSNRQSSFTFHPAVNTKPKSVNLV---TAVHAAEPARNAVSVKE----------SVAS
ArDAHPS1    1 MALSNTATLSSKSLYSNTQT--LSHHQPTFSVVPTTTTKTNSGKLVTRISAVHAAEPSTK------------------PS
ArDAHPS2    1 MALSNTATLSSKSLYSNTQT--LSHHQPTFSVVPTTTTKTNSGKLVTRISAVHAAEPSTK------------------PS
ArDAHPS3    1 MALTSSSVIPTKSFIQTQSSLLPST-------KPHQPSIPSRRRSLPSISAVHAAEPAKNPVVSDKPPKQQVTPPPTKTT
ArDAHPS4    1 MALTVTSTLSSRPLLQPHQPAKPLH-------HHHHLSLPDRK---KPISAVHAADHSKS--------------SKSTPT
ArDAHPS5    1 MALTLKGSVN----------LTPPTITKALPLKPHFL---------TP---LHFTTTRHIPSVSS-----SSSSVTDNAN

AtDAHPS    68 SSSGALKWTPESWKLKKALQLPDYPNANELESVLKTIEAFPPIVFAGEARNLEERLADAAVGKAFLLQGGDCAESFKEFN
ArDAHPS1   61 AAASPSKWTVESWKSKPTLQLPEYPNQQELETVLKTIEAFPPLVFAGEARHLEEKLAEAAIGNAFLLQGGDCAESFKEFN
ArDAHPS2   61 AAASPSKWTVESWKSKPTLQLPEYPNQQELETVLKTIEAFPPLVFAGEARHLEEKLAEAAIGNAFLLQGGDCAESFKEFN
ArDAHPS3   74 HNAVPVTWSVESWKTKKALQLPEYPNQEELESVLKTLDTFPPIVFAGEARNLEERLAEAAMGNAFLLQGGDCAESFKEFN
ArDAHPS4   57 TTPTPTRWSLDSWKSKKVFQLPEYPDQNALDFVLNTLESFPPVVFAGEARSLEDRLAEAAQGKAFLLQGGDCAESFKEFN
ArDAHPS5   54 TSTGPSNWAPYSWKSKKARQLPDYPDPDELRSVIQTLESFPPIVFAGEARKLEERLAQAAMGEAFLLQGGDCAESFKEFN

AtDAHPS   148 ATNIRDTFRVLLQMSIVLTFGGQVPVIKVGRMAGQFAKPRSDAFEEKDGVKLPSYKGDNINGDTFDEKSRIPDPNRMIRA
ArDAHPS1  141 ANNIRDTFRVLLQMGAVLMFGGQVP---VGRMAGQFAKPRSDSLEEKDGVKLPSYKGDNINGDEFTEKSRIPDPERLIRA
ArDAHPS2  141 ANNIRDTFRVLLQMGAVLMFGGQVPVIRVGRMAGQFAKPRSDSLEEKDGVKLPSYKGDNINGDEFTEKSRIPDPERLIRA
ArDAHPS3  154 ANNIRDTFRIILQMGAVLMFGGQLPVIKMGRMAGQFAKPRSDPFEEKNGVKLPSYRGDNVNGDAFDEKSRTPDPQRMIRA
ArDAHPS4  137 ANNIRDTFRLLLQMGVVLMFGGQMPIIKVGRMAGQFAKPRSDPFEEKDGVKLPSYRGDNINGDSFDTESRVPDPDRMIRA
ArDAHPS5  134 GNNIRDTFRVLLQMGIALTYGAQIPVIKVGRMAGQFAKPRSDPFEIKDGVKLPSYRGDNINGDAFDERSRTPDPQRLIRA

AtDAHPS   228 YTQSAATLNLLRAFATGGYAAIQRVTQWNLDFVEQSEQADRYQELANRVDEALGFMSACGLGTDHPLMTTTDFYTSHECL
ArDAHPS1  218 YCQSAATLNLLRAFATGGYAAIQRISEWNLDFAEHSEQGDRYQELANRVDEALGFMAAAGLTVDHPVMRTTEFWTSHECL
ArDAHPS2  221 YCQSAATLNLLRAFATGGYAAIQRISEWNLDFAEHSEQGDRYQELANRVDEALGFMAAAGLTVDHPVMRTTEFWTSHECL
ArDAHPS3  234 YCQSAATLNLLRAFATGGYAAMQRVTQWNLDFAEHSEQGDRYRELAHRVDEALGFMTAAGLTVDHPIMTTTDFWTSHECL
ArDAHPS4  217 YGQSVATLNLLRAFATGGYAAMQRVTHWNLDFMEHSEQGDRYRELARRVDEALGFMSAAGLTADHPIMTTTDFWTSHECL
ArDAHPS5  214 YLQSVGTLNLLRAFATGGYAAMQRVSQWNLDFVVHSEQGDRYMELAQRVDEALGFMAAAGVTVNHPIMNTIEFWTSHECL

AtDAHPS   308 LLPYEQSLTRLDSTSGLYYDCSAHMVWCGERTRQLDGAHVEFLRGIANPLGIKVSNKMDPFELVKLVEILNPNNKPGRIT
ArDAHPS1  298 HLPYEQSLTRKDSTSGLYYDCSAHMVWVGERTRQLDGGHVEFLRGIANPLGIKVSNKMDPNDLVKLIEILNPHNKPGRIT
ArDAHPS2  301 HLPYEQSLTRKDSTSGLYYDCSAHMVWVGERTRQLDGGHVEFLRGIANPLGIKVSNKMDPNDLVKLIEILNPHNKPGRIT
ArDAHPS3  314 LLPYEQSLTRLDSTSGLYYDCSAHFLWAGERTRQLDGAHVEFLKGIANPLGIKVSDKMDPNELVKLIEILNPQNKAGRIT
ArDAHPS4  297 LLPYEQALTREDSISGLYYDCSAHMLWVGERTRQLDGAHVEFLRGVANPLGIKASDKMDPNELVRLIDILNPKNKPGRIA
ArDAHPS5  294 HLPYEQALTRVDSTSGCYYDCSAHMLWVGERTRQLDGAHVEFLRGVSNPLGIKVSDKMDPKELVKLCEILNPRNKPGRLT

AtDAHPS   388 VIVRMGAENMRVKLPHLIRAVRRSGQIVTWVCDPMHGNTIKSTCGLKTRAFDSILAEVRAFLDVHEQEGSHAGGIHLEMT
ArDAHPS1  378 IIARMGAENMRVKLPHLIRGVRRAGQIVTWVCDPMHGNTIKAPCGLKTRPFDAILAEVRAFFDVHEQEGSHPGGVHLEMT
ArDAHPS2  381 IIARMGAENMRVKLPHLIRGVRRAGQIVTWVCDPMHGNTIKAPCGLKTRPFDAILAEVRAFFDVHEQEGSHPGGVHLEMT
ArDAHPS3  394 IITRMGAENMRVKLPHLIRAVRRAGHIVTWVSDPMHGNTIKAPCGLKTRPFDSIRAEVRAFFDVHEQEGSHPGGVHLEMT
ArDAHPS4  377 VIVRMGAENMRVKLPHLIRAVRGAGQIVTWVSDPMHGNTIKAPCGLKTRSFDAIRAEVRAFFDVHDQEGSYPGGVHLEMT
ArDAHPS5  374 IITRMGADNMRVKLPHLIRAVRQAGLIVTWVSDPMHGNTIKAPSGLKTRPFDAIRAELRAFFDVHEQEGSYPGGVHLEMT

AtDAHPS   468 GQNVTECIGGSRTVTYDDLSSRYHTHCDPRLNASQSLELAFIVAERLRKRRTGSQRV-S----
ArDAHPS1  458 GQNVTECIGGSRTVTFDDLSSRYHTHCDPRLNASQSLELAFIVAERLRKRRIGTQRL-ASLSL
ArDAHPS2  461 GQNVTECIGGSRTVTFDDLSSRYHTHCDPRLNASQSLELAFIVAERLRKRRIGTQRL-ASLSL
ArDAHPS3  474 GQNVTECIGGSRTVTFDDLSSRYHTHCDPRLNASQALELAFIIAERLRKRRIRSQQPLSSLGF
ArDAHPS4  457 GQNVTECVGGSVTVTYNDLSSRYHTHCDPRLNASQSLELSFIIADRLQRRRIGSRRFLSVS--
ArDAHPS5  454 GQNVTECIGGSKTVTFDDLSSRYHTHCDPRLNASQSLELAFAIAERLRKKRLKSVNDFQRGQR
```

Supplementary Figure S2. Protein sequence alignment of *bona fide* 3-deoxy-D-arabino-heptulosonic acid 7-phosphate synthase (DAHPS) from *Arabidopsis thaliana* (AtDAHPS (4)) and that of putative homologs (ArDAHPS1 – ArDAHPS5) in red alder.

```
SlDHQS    1 MASSFCPKQALSFTNSTHQLHQSRAIPRDIHVRFPAPVSSPSSRCGLKS------------------KATTRLKVLATSAT
ArDHQS    1 MATTTNPFS-LSLS----------KNSADIFSRVPNPPNSVSLRSAFVSPGSSDLSLIRGQNSVVSASRVRSVRICASSA

SlDHQS   64 KVMDHSSSKASSQAPTVVEVDLGTRSYPIYIGAGLLDQPDLLQRHIHGKRVLVVTNTTVAPLYLDKTISALTDGNPNVTV
ArDHQS   70 QVMDQSVAKTDSKVPVIVDVNLGDRSYPIYIGSGLLDQPHLLQRHVHGKRVLVVTNNTVGPIYLDKVVEALTKGNPNVSV

SlDHQS  144 ESVILPDGEQFKNMETLMKVFDKAIESRLDRRCTFVALGGGVIGDMCGYAAASYLRGVNFIQIPTTVMAQVDSSVGGKTG
ArDHQS  150 ESVILPDGEKYKDMDTLMKIFDKAIESRLDRRCTFVALGGGVIGDMCGFAAASFLRGVNFIQIPTTVMAQVDSSVGGKTG

SlDHQS  224 INHPLGKNMIGAFYQPQCVLIDTDTLNTLPDRELASGLAEVIKYGLIRDAEFFEWQEQNMPLLLARDPTAFTYAIKRSCE
ArDHQS  230 INHRLGKNLIGAFYQPQCVLIDTDTLNTLPDRELASGLAEVIKYGLIRDAEFFEWQERNIQALMSRDPSALAYAIKRSCE

SlDHQS  304 NKADVVSQDEKESGVRATLNLGHTFGHAVETGVGYGQWLHGEAVAAGTVMAVDMSRRLGWIDDSLVQRVQKILQQAKLPT
ArDHQS  310 NKAEVVSLDEKEGGLRATLNLGHTFGHAIETGVGYGLWLHGEAVAAGMVMAVDLSRRLGWIDDSIVKRVHNILTQAKLPT

SlDHQS  384 SPPETMTVEMFKSIMAVDKKVADGKLRLILLKGSLGNCVFTGDYDQKALDETLRAFSKS
ArDHQS  390 APAKTMTVEMFKSVMAVDKKVADGLLRLILLKGPLGNCVFTGDYDRKAVDETLRAFCKS
```

Supplementary Figure S3. Protein sequence alignment of *bona fide* 3-dehydroquinate synthase (DHQS) from *Solanum lycopersicum* (SlDHQS (5)) and that of a putative homolog (ArDHQS) in red alder.

```
AtDHQD-SDH            68 ----------MEPSNVYVASNSTEMEIGSHDIVKNPSLICAPVMADSIDKMVIETSKAHELGADLVEIRLDWLKDFNPLE
FvDHQD-SDH3            1 -------------------------------MMGSSTLVCAPIMAETVHKMVRDMSKARDLGADVVEIRLDYLKVFNSNQ
FvDHQD-SDH4            1 --------------MDSPTLTVASAQVGGGGMRKSSTLICAPIMAESVAKMVVEMGRAKAVGADLVEIRLDHLKVFDSSE
NtDHQD-SDH1            1 ------------MELV---VDSGVRKMEGEAMTRNETLICAPIMADTVDQMLNLMQKAKISGADLVEVRLDSLKSFNPQS
SlDHQD-SDH1            1 ------------MELV---VDSGVKKMEGEAMRKNQTLICAPIMADSVDQMLILMQKAKISGADLVEVRVDSLKSFNPRP
CasDHQD-SDH4           1 ------------MELVAPVVAGCGVKMESEGMRKNSTLICVPLMADSVDQMLIYMNKAKLNGADLVEVRLDSLKSFNPPL
VvDHQD-SDH1            1 --------------------------MESGGMSKNSTLICVPIMGETIEKMVVDMSKAKTSGADLVEVRLDTLKRFNPRQ
FSB010265701           1 ----------MDSSSVLLASSSAGLQMGGGEIRKNPTLVCAPIMAESVDKMVINMDKAKQGGADLVEIRLDSLKSFNPNE
FSB012889301           1 ----------MDSTNVLLASSSAGLQIGGGEIRKNPTLVCAPIMAESVDKMVINMDKAKQGGADLVEIRLDSLKSFNPNE
Qrob_P0275560          1 ----------MDSTNVLLASSSAGLEMGIREIRKNQTLLCAPIMAETVDKMVINMDKAKQSDADLVEIRLDSLKSFNPYE
Juni_01730             1 --------------------------MASGGGRNGPTLVCAPIMAESVDKMVANMEKAKQGGADLVEIRLDSLKSFNPHE
JrDHQD-SDH             1 ------------------------------------TLVCAPIMAESVDKMVINMNKAKQGDADLVEIRLDSLKSFNPSN
Juni_19979             1 -----------MESSNVLLASSAALQMGSGG-RNNPTLVCAPIMAESVDKMVINMNKAKQGDADLVEIRLDSLKSFNPSD
ArDHQ-SDH1             1 -----------MESGNVLLASSAALQMRSGGARNNSTLVCAPVMADSVDKMVTNMDKAKREGADLVEIRLDSLKSFDPYE
Alngl88491S37275       1 -----------MESGNVLLASSAALQMRSGGARNNSTLVCAPVMADSVDKMVTNMDKAKREGADLVEIRLDSLKSFDPYE
Bpev01_c0420-g0007     1 -----------MESRNVLLASSAALQMGSGGVRNNPTLVCAPVMAESVDKMVTNMDKAKQDGADLVEIRLDSLKSFDPYE
Corav_882                --------------------------------------------------------------------------------
Casgl15S00444          1 -----------------MLASSSAPQMATEGVRNNPSLVCAPIMAESIDKMVINMGKAKQDGADLVEIRLDSLKSFHPDE
EgDHQD-SDH5            1 ---------MASTGNILLATSSAPHEMESGAVRKNSTLICVPVMADSVEEMVIQLDKAKSSGADLVEIRVDGLKNLSPHE
EcDHQD-SDH1            1 ---------MASTGNVLLATSSAPHEMESGAVRKNSTLICVPVMADSVEEMVIQLDKAKSSGADLVEIRVDSLKNLSPHE
CsDHQD-SDH3            1 -------------MESPNLLVASGSKLVSGGMRKNPTLICVPIMGESVDKMVVDMGKANASGADLVEIRLDGLKNFNPRE
PoptrDHQD-SDH1         1 ------MDSASNVLLASSPSAAAAVGMGSGGVRRNPTLICTPIMADSVDKMAILMAEAKSVGADLVEIRLDSLKDFNPNS
PoptrDHQD-SDH3         1 -------------------------MGSVGV-LTNSVMVCAPLMAQSVEQMVIDMHSAKAQGADVVEVRLDCISKFQPRQ
PoptrDHQD-SDH2         1 -------------------------MGRAGI-LANSTMVCAPLMSRSVEQMVIDMQSAEAQGADAVEVRLDYINSFQPSQ
EgDHQD-SDH4            1 -------------------------MGSLSLSSVGLTMVCAPVMGESVDQVVEEMHKAKAQGADVVEVRLDCIKQFQAHQ
EcDHQD-SDH4a           1 -------------------------MGSLSLSSVGLTMVCAPVMGESVDQVVEEMHKAKAQGADVVEVRLDCIKQFQAHQ
EcDHQD-SDH4b           1 -------------------------MGSLSLSSVGLTMVCAPVMGESVDQVVEEMHKAKAQGADVVEVRLDCIKQFQAHQ
CasDHQD-SDH1           1 -------------------------MGSVGV-LTNSTTICAPLMSQSVEQMVSDMNQAKAQGADVVEIRLDCIKDFQPNR
SlDHQD-SDH2            1 -------------------------MGSVGL-LKNSAMVCAPLMATSVDQLIDEMVEAKSQGADCVEIRLDAIHNFQPHK
NtDHQD-SDH2            1 -------------------------MGSVGL-LKNSAMVCAPLMAPSVEQLVHGMLQAKAQGADLVEIRLDGINNFQPQK
CsDHQD-SDH1            1 -------------------------MGVVNI-TKNTTMICAPLMAQSVEQVLSNMYQAKAEGADVVEIRLDCINNFQPGK
VvDHQD-SDH2            1 -------------------------MDDVGV-LKKETMICTPLMGQSVEQMVRDMHKAKVEGADLVEVRLDYINNFHPQQ
Qrob_P0208710          1 -------------------------MGSIGMMVSKTTLVCAPLMAETVEQIMTDMYKAKVQGADLVEIRLDYIKNFQPRQ
FSB015730401           1 -------------------------MGSLGI-VSKSTLVCAPLMSQTVEQIVTDMYRAKVQGADLVEVRLDYIKNFKPHQ
Juni_23842             1 -------------------------MGSIGM-LNNSTLICAPLMAQSVEQMVSDMYEAKAQGADVVEVRLDCIKNFQPRQ
Bpev01_c1356-g0002     1 -------------------------MGSIGI-VDNSTLICAPIVAQSVEQMVIDMKQAKAEGADVVEVRLDCLKNFQPRQ
Corav_4151             1 -------------------------MGTVGI-FDRSTLICAPLVAQSVEQMVIDMKQAKAEGADVVEVRLDYIKNFQPRQ
Alngl20613S19962       1 -------------------------MGSVGI-LENSTLICAPLVAQSVEQMVIDMKQAKAEGADVVEVRLDYIKNFQPRQ
ArDHQD-SDH2            1 -------------------------MGSVGI-LENSTLICAPLVAQSVEQMVIDMKQAKAEGADVVEVRLDYMKNFQPRQ
CasDHQD-SDH2           1 ------------MASGSFSFATSDVQTSSSGVRSGPTLLCTPLIGTTVDQMLTDMRKAKEIGADMVEIRLDCLREFNPRP
VvDHQD-SDH3            1 --------------MGSLPFTVSDLQTSVSGVRSNPTLLCTPLMGTTVEQMLTEMRKAKEIGADIVEIRLDCLRNFSPAQ
FvDHQD-SDH1            1 --------------MGSLPFTTSDLHTSTGGFLSSPTLLCTPLMGTTVDQMLIEMHKAKEIGSDVVEIRLDCLRNFNPSS
EgDHQD-SDH3            1 --------------MGSVPFTTSDLQTSTSGFRSSPTLLCTPLMGTTVDQMLIEMRKAKEIGADLVEIRLDCLRNFNPHQ
EcDHQD-SDH3            1 --------------MGSVPFTTSDLQTSTSGFRSSPTLLCTPLMGTTVDQMLIEMRKAKEIGADLVEIRLDCLRNFNPHQ
Qrob_P0208720          1 --------------MGSLPFTVSDLQASTNGARSSPTLLCTPLMGTTVDQMLIEMGKAKEIGADVVEIRLDCLRTFNPRQ
FSB015730501           1 --------------MGSLPFTVSGLQTSIDGARSGPTLLCTPLMGTTVDQMLIEMGKAKEIGADVVEIRLDCLRKFNPRQ
Corav_14055            1 --------------MASLPFTVSGLQTSSTGGRSSPTLLCTPLMGTTVDQMLIEMQKAKEIGSDVVEIRLDCLRNFNPRP
Corav_11016            1 --------------MASLPFTVSGLQTSSTGGRSSPTLLCTPLMGTTVDQMLIEMQKAKEIGSDVVEIRLDCLRNFNPRP
Bpev01_c1356-g0003     1 --------------MASLPFTVSGLQTSSTGGRSSPTLLCTPLMGTTVDQMLIEMQKAKEIGSDVVEIRLDCLRNFNPRP
ArDHQD-SDH3            1 --------------MGSLPFTVSGLQTSSTGGRSSPTLLCTPLMGTTVDQMLIEMQKAKEIGSDVVEIRLDCLRNFNPRP
CasDHQD-SDH3           1 -------------------------------------MICAPVMAENVDQMLLMMRKAKELGADLVEVRIDYLKNFSPHH
DkDHQD-SDH             1 -------------------------------------MICAPVMAETAEQMLGQMRKAKELGADLVEIRIDYLKSFSPQQ
VvDHQD-SDH4            1 ------------MTLSSVPLATSDIQIPE-GARRNSTLICVPIMADSVDQMLGQIRKAKEVGGDLVEIRLDYLKNFSPRQ
EgDHQD-SDH2            1 ------------MTLSSIPLTAADLQIPA-GGRRNSTLLCAPVMGESVDQMLGQIRAAKEQGADLVEVRLDFLKSFSPKQ
EcDHQD-SDH2            1 ------------MTLSSIPLTAADLQIPA-GGRRNSTLLCAPVMGESVDQMLGQIRAAKEQGADLVEVRLDFLKSFSPKQ
PoptrDHQD-SDH5         1 ----------------------MDLQSAD-GERRNSTLICAPIMAESVDQMLVQMKRAKELGADVAEVRVDFLKNFSPRN
FvDHQD-SDH2            1 ------------MTLSSIPLVASDLQISY-GTGRNSTLICAPVMGESVDQMLRQLQQAKELGADLVEIRLDYIKNFSPRQ
Casgl267S13432         1 ------------MTLSSVPLAATDLKMGD-AVRRNSTLICAPVMAETVDQMLIQTRNAKELGADLVEIRLDSLKNFSPRQ
Qrob_P0208730          1 ------------MTLSSIPLATSDLQISG-GIRRSSTQICAPVMAETVDQMLLLMRKAKELGADLVEVRVDFLKSFNPRQ
FSB015730601           1 ------------MTLSSIPLATSDLQIAD-GTRRSSTLICAPVMAETVDQMLLQMRKAKELGADLVEIRVDFLKNFSPRQ
Juni_23843             1 ------------MTLDSIPLATMDLQIAD-GVRRNGTQICAPVMAETVDQMLTQARKAKELGADLVEIRLDFLKTFNPRQ
ArDHQD-SDH4            1 -----------MTLIGSVPLATSDLQIVD-GARRSSTLICAPVMAETVDQMLIQTRKAKELGADLVEIRLDFLKNFSPRQ
Alngl6638S34349        1 ---------------------------------------------------------------MLIRQGFEQYKNFT---
Bpev01_c1356-g0004     1 ------------MTLGSVPLATTDLQIVG-GARRSSTLICAPVMAETVDQMLIQARKAKELGADLVEIRLDFLKNFSPTQ
Corav_941              1 ------------MTLGSVPLATPDLQIVG-GARRSSTLICAPVMAETVDQMLIQARKAKELGADLVEIRLDFLKNFSPRQ
Corav_7742             1 ------------MTLGSVPLATPDLQIVG-GARRSSTLICAPVMAETVDQMLIQARKAKELGADLVEIRLDFLKNFSPRQ
SlDHQD-SDH3            1 MQVLTWFVLYMFPPLYKRERWSVKEERDIIMGLKNDLVVYTRLECESCEEMTCCIEKAKEEGADLVELCIDDF-TFSDIS
EgDHQD-SDH1           40 VAEGVTAQTLHSSSRIFFLHCPSSRRERPREMDRNGVLVCAPLECETLEGMLSSMDKAKAHGADLVELRVDAM-SFGRVS
PoptrDHQD-SDH4         1 ------------------------------MAFKNNLLVCTPLECETAGEMLSSMKRAETEGADLTELRLDSL-SFSHNS
CsDHQD-SDH2            1 ----------------------------MEVAAKNSLLVCTQLECETTEEMQASIEQAKVEGADLVELCIDSM-EFSHIS
```

```
Qrob_P0339740        1 ------------------------------MAFKKNLLVCTQLQCETMEEMLASMEKAKAEGADLVELCIDSM-SFSHTS
Juni_24457           1 ------------------------------MAFKNNILVCTPLECETEEEMVGSMELAKAEGADLVELCVDSM-SFSHIS
Casgl106S06827       1 ------------------------------MAFKNNLLVCTSLECETMEEMLASMDKAKAEGADLVELCVDSL-SFSHIS
Bpev01_c0171-g0015   1 ------------------------------MAFKNNLLLCTPLECETMEEMQASMEKAKAEGADLVELCLDSM-QFSHIS
ArDHQD-SDH5          1 ------------------------------MAFKNNLLLCTPLECETMEEMQASMEKAKAEGADLVELCLDSM-SFSHIS
ArDHQD-SDH6          1 --------------------------------------------------------------------------------

AtDHQD-SDH         138 DLKTIIKKSPLPTLFTYR-------------------PKWEGGQYEGDE---NERRDVLRLAMELGADYIDVELQVASEF
FvDHQD-SDH3         50 DLKTLIKESPMPTLFTYR-------------------PKWEGGQYDGDE---KHRQDILRLAMELGADYIDVEFQVAHEF
FvDHQD-SDH4         67 DVKTLIDQSPLPTLFTYR-------------------PKWEGGQYDGDE---KSRLDALRLAMELGADYIDVELQVAQEF
NtDHQD-SDH1         65 DIDTIIKQSPLPTLFTYR-------------------PTWEGGQYAGDE---VSRLDALRVAMELGADYIDVELKAIDEF
SlDHQD-SDH1         66 DIDTLIKQCPLPTLFTYSYVLGVGQGILLIRYYKGIGPTWEGGQYAGDE---KSRLDALRLAMELGADYIDVELKAIGEF
CasDHQD-SDH4        69 DIETLIKRCPLPTLFTYR-------------------PKWEGGMYDGDE---SSRLEALQLAMELGADYIDVELQVIHEF
VvDHQD-SDH1         55 DLEVLIRKCPLPTLFTYR-------------------PKWEGGQYEGDE---NSRRDALRLAMELGADYVDIELKVAHEF
FSB010265701        71 DLKTLIKECPLPTLFTYR-------------------PKWEGGQYDGDE---KKRLDALRLAMEFGADYIDVELQVAQEF
FSB012889301        71 DLKTLIKECPLPTLFTYR-------------------PKWEGGQYDGDE---KKRLDALRLAMEFGADYIDVELQVAQEF
Qrob_P0275560       71 DLKTLIKECPLPTLFTYR-------------------PKWEGGQYDGDE---KKRLDALRLAMEFGADYIDVELQVAREF
Juni_01730          55 DLKNLIKECPLPTLFTYR-------------------PKWEGGQYDGDE---KKRLDALRLAMEFGADYIDVEIQVAREF
JrDHQD-SDH          45 DLKTIIKASPLPTLFTYR-------------------PKWEGGQYDGDE---KKRLDALRLAMEFGADYIDVELQVACEF
Juni_19979          69 DLKTIIKASPLPTLFTYR-------------------PKWEGGQYDGDE---KERLDALRLAMEFGADYIDVELQVACEF
ArDHQ-SDH1          70 DLKTLIKECPLPTLVTYR-------------------PTWEGGQYDGDE---KTRLDALRLAMEFGADYIDVELQVAHEF
Alngl88491S37275    70 DLKTLIKECPLPTLVTYR-------------------PTWEGGQYDGDE---KTRLDALRLAMEFGADYIDVELQVAHEF
Bpev01_c0420-g0007  70 DLKTLIKECPLPTLFTYR-------------------PTWEGGQYDGDE---KKRLDALRLAMEFGADYIDVELKVAHEF
Corav_882              --------------------------------------------------------------------------------
Casgl15S00444       64 DLKTLLKECPLPTLFTYR-------------------PTWEGGMYDGDE---KKRLDALRLAMELGADYIDVELQVAHEF
EgDHQD-SDH5         72 DLKTLIKASALPTLFTYR-------------------PKWEGGQYDGDD---KPRLETLRLAMELGADYIDVELKVASEF
EcDHQD-SDH1         72 DLKTLIKASALPTLFTYR-------------------PKWEGGQYDGDD---KPRLETLRLAMELGADYIDVELKVASEF
CsDHQD-SDH3         68 NIKTLIKESPVPTLFTYR-------------------PIWEGGQYDGDE---NERVDVLRLAMELGADYIDVELQVAREF
PoptrDHQD-SDH1      75 DIKTLILHSPLPTLFTYR-------------------PMWEGGQYNGDE---KPRLDALRLAMELGADYIDVELKVAHEF
PoptrDHQD-SDH3      55 DLETIIRNKPLPVIIVYRP-------------------KWEGGQYEGDE---HRRLEALRLANDLGADYIDLELKVASEL
PoptrDHQD-SDH2      55 DLETIIRNKPLPVIIVYRP-------------------RWEGGQYEGDE---HTRLEALRLAHELGADYIDVELKVASDL
EgDHQD-SDH4         56 DLEIILKSKPLPVIIVYRP-------------------KCEGGLYEGDE---TARLEALHSALKLGADYVDFELKVAAEL
EcDHQD-SDH4a        56 DLEIILKSKPLPVIIVYRP-------------------KCEGGLYEGDE---TARLEALRSALKLGADYVDFELKVAAEL
EcDHQD-SDH4b        56 DLEIILKSKPLPVIIVYRP-------------------KCEGGLYEGDE---TARLEALRSALKLGADYVDFELKVAAEL
CasDHQD-SDH1        55 DL ILL NKPLPVLIVYRP-------------------QWEGGQYGGDE---NMRLDTLRLAKELGADYIDLELKVASHL
SlDHQD-SDH2         55 HLQLLFKNKPLPILILYRP-------------------IWERNDFEADA---HKQLEALRLAKELGADYVELDLKIASEF
NtDHQD-SDH2         55 DLQVLLNNNPLPVLIVYRP-------------------IWEGNEFEEDDDHIHKQLEVLRWAKELGADYIELDLKIASDF
CsDHQD-SDH1         55 DLEIILTKKPLPVLIVYRP-------------------KWAGGLYEGDE---HKRLEALHLAEDLGADYVDFELKVASNI
VvDHQD-SDH2         55 DLEIILRNKPLPVMIVYRP-------------------KWEGGQYEGDE---HSRLEALHLAEKLGADYIDFELKVASDF
Qrob_P0208710       56 DLETILKKKPLPVLIVYRP-------------------KWEGGQYEGDE---HTRLETLHTAKELGADYIDFELKVASNL
FSB015730401        55 DLETILKKKPLPVLIVYRP-------------------KWEGGQYEGDE---HSRLETLNLAKELGADYIDFELKVASNH
Juni_23842          55 DLQTIIRKKPLPVLIVYRP-------------------RWEGGQYEGDE---CTRVETLHLAKDLGADYIDFELKVASNL
Bpev01_c1356-g0002  55 DLEIILKNKPLPVIVVYRP-------------------KWEGGQYEGDE---HMRVETLQLAKELGADYIDFELKVASDL
Corav_4151          55 DLEIILRNKPLPVMIVYRP-------------------KWEGGQYEGDE---HMRVETLQLAKELGADYIEFELKVASDL
Alngl20613S19962    55 DLEIILRNKPLPVIIVYRP-------------------KWEGGLYEGDE---HTRVETLQLAKELGADYIDFELKVASDL
ArDHQD-SDH2         55 DLEIILRNKPLPVIIVYRP-------------------KWEGGLYEGDE---HTRVETLQLAKELGADYIDFELKVASDL
CasDHQD-SDH2        69 DLQILIKQSPLPTLVTYR-------------------PIWESGQYEGDE---NKRQDALRLAMELGADYIDVELQVAQEF
VvDHQD-SDH3         67 DLQILIKQSPLPTLVTYR-------------------PIWEGGQYEGDE---NKRQDALRLAMELGASYIDVELEVAHEF
FvDHQD-SDH1         67 DLQILIKQSPLPTLVTYR-------------------PVWEGGQYEGDE---TKRQDALRSAMELGANYIDIELEVAHEF
EgDHQD-SDH3         67 DLQILIKQSPLPTLVTYR-------------------PVWEGGQYEGDE---SKRQDALRLAMQLGAHYIDVELEVAHDF
EcDHQD-SDH3         67 DLQILIKQSPLPTLVTYR-------------------PVWEGGQYEGDE---SKRQDALRLAMQLGAHYIDVELEVAHDF
Qrob_P0208720       67 DLEILIKRSSLPTLVTYR-------------------PIWEGGQYEGDE---NKRQDALRLAMELGANYIDVELEVAHDF
FSB015730501        67 DLEILIKCSSLPTLVTYR-------------------PVWEGGQYEGDE---NKRQDALRLAMELGANYIDVELEVAHDF
Corav_14055         67 DLEFLVKQSPLPTLVTYR-------------------PVWEGGQYEGDE---TKRQDALRLAMELGADYIDVELEVAPDF
Corav_11016         67 DLEFLVKQSPLPTLVTYR-------------------PVWEGGQYEGDE---TKRQDALRLAMELGADYIDVELEVAPDF
Bpev01_c1356-g0003  67 DLEFLVKQSPLPTLVTYR-------------------PVWEGGQYEGDE---TKRQDALRLAMELGADYIDVELEVAHDF
ArDHQD-SDH3         67 DLEFLVKQSPLPTLVTYR-------------------PVWEGGQYEGDE---TKRQDALRLAMELGADYIDVELEVAHDF
CasDHQD-SDH3        44 DLQILIKQCPLPTLITYR-------------------PTWEGGQFDGDE---TRRQAALRQAVELGADYIDIELKVADEF
DkDHQD-SDH          44 HLEVLIKQSPLPTIITYR-------------------PTWEGGQYDGDE---SRRQATLHQAMELGADYIDIELKVADEF
VvDHQD-SDH4         68 DLQFLVKQSPLPTLVTYR-------------------PTWEGGQYDGDE---GKRLDALRLAIELGADYIDVELQVAPEF
EgDHQD-SDH2         68 DLEILLKQSALPTLVTYR-------------------PKWEGGQYEGDD---SRRLDALRLALELGADYVDVELQVAQEF
EcDHQD-SDH2         68 DLEILLKQSALPTLVTYR-------------------PKWEGGQYEGDD---SQRLDALRLALELGADYVDVELQVAQEF
PoptrDHQD-SDH5      58 DLEALIKQCPLPTLITYR-------------------PKWEGGQYDGDE---NKRQKALQIAMELGADFIDIELKVAQEF
FvDHQD-SDH2         68 DLETLIKRSPLPTLVTYR-------------------PKWEGGQYEGDE---KKRQEALILAMELGADYIDVELKVANDF
Casgl267S13432      68 DLEILINKSSLPTLVTYR-------------------PKWEGGLYDGDE---SKRQNALCLAMELGAHYIDVELKVAQEF
Qrob_P0208730       68 DLEILIKQSPLPTLITYR-------------------PIWEGGQYDGDE---SQRQNALRLAVELGADYVDVELKVAHDF
FSB015730601        68 DLEILIKQSPLPTLITYR-------------------PKWEGGQYDGDE---SQRQNALRLAMELGADYIDVELQVAHDF
Juni_23843          69 DLEILIKQCPLPTLITYR-------------------PIWEGGKYDGDE---SKRQNALHLAMELGADYIDVELKVAHDF
ArDHQD-SDH4         69 DLEILIKQCPLPTLITYR-------------------PKWEGGEYDGDE---NKRQTALRLAMELGADYIDVELKVAHDF
Alngl6638S34349        --------------------------------------------------------------------------------
```

```
Bpev01_c1356-g0004    68 DLEILIKQSPLPTLITYR-------------------PKWEGGQYDGDE---SKRQTALRQAMELGADYIDVELQVAHEF
Corav_941             68 DLEILIKQSALPTLITYR-------------------PKWEGGQYDGDE---GKRQTALRLAMELGADYIDVELKVAHDF
Corav_7742            68 DLEILIKQSALPTLITYR-------------------PKWEGGQYDGDE---GKRQTALRLAMELGADYIDVELKVAHDF
SlDHQD-SDH3           80 QLEELLKQRSLPSIVSFR---------------------PKSPINS---EGKKTCIQVLKLAVELDVEFVEVDTQVVCHQ
EgDHQD-SDH1          119 EVEELIRRRTLPAIVSFR---------------------LNSARASRRQNDKTTCLQVLRLALELDVEFVEMEHEVVSHF
PoptrDHQD-SDH4        50 EVEKLIKQRTLPSIVSFR---------------------LEPSRISSNKDRKNTCLQVLRLAFDLNVEFVEMDYEVASED
CsDHQD-SDH2           52 EVDKLIQHPTLPAIVSYR---------------------LKSSRKSSDEACKNTCLQVLRRALDLDVEFVEMDYEVASDP
Qrob_P0339740         50 EVEKLIKQRALPAIVSYR---------------------LKSSRTSGKGECKTTCMQVLRLALELDVEFVEMDFEVASDI
Juni_24457            50 EVKKLIQHRTLPAIVSYR---------------------LKSSRTSGKGDSKFLCLQVLRLALEVDVEFVEMDYEVASDI
Casgl106S06827        50 EIEQLIKQRTLPTIVCFR---------------------LQSSGTSGKGGCKFTCSQVLRLALELDVEFVEMDYEVVSNI
Bpev01_c0171-g0015    50 EVENLIKQRTLPSIVSFR---------------------LKSSRTSGKGDCKFTCLQVLRLALELDVEFVEMDYEVASDI
ArDHQD-SDH5           50 EVQKLIKQRTLPSIVSFR---------------------LKSSGSSGKGDCKFTCLQVLRLALELDVEFVEMDYEVASDV
ArDHQD-SDH6            1 -----------------------------------------------------------------------MDYEVASDV

AtDHQD-SDH              IK-SIDGKKPGKFKVIVSSHNYQN-TPSVEDLDGLVARIQQTGADIVKIATTAVDIADVARMFHITSKAQV---PTIGLV
FvDHQD-SDH3             ID-SINGNKPEKLKVIVSSHNYQD-TPSVKDLGDLVARIQATGADIVRITTTALDITDVARIIQISVHSHV---PVIGLA
FvDHQD-SDH4             VD-FIRDKKPEKFKVIVSSHNYQE-TPSVEALGNLVAAIQATGADIVKIATTALDITDVVRIFQITVHSQV---PIIGLV
NtDHQD-SDH1             NT-ALHGNKSAKCKVIVSSHNYDN-TPSSEELGNLVARIQASGADIVKFATTALDIMDVARVFQITVHSQV---PIIAMV
SlDHQD-SDH1             NN-ALHGNKSAKCKLIVSSHNYES-TPSAEDLGNLVARIQASGADIVKFATTAQDITDVARVFQITVHSQV---PIIAMV
CasDHQD-SDH4            NS-SMHGKKPAKCKLIVSSHNYEN-TPSVEDLGNLVVKIQATGADIVKIATTALDITDAARVFQITVHSQVRGVPIIAMV
VvDHQD-SDH1             IN-SIHGRKPEKFKVIVSSHNYQN-TPSVEDLGNLVVSIQATGADIVKIATTALEITDVARIFQITVHSQV---PVIGLV
FSB010265701            NE-SIYGKKPEKFKVIVSSHNYQN-TSSVEDLGNLVARIQATGADIVKFATTALAITDVARIFQIIVHSQV---PIIAIV
FSB012889301            NE-SIYGKKPEKFKVIVSSHNYQN-TSSVEDLGNLVARIQATGADIVKFATTALAITDVARIFQIIVHSQV---PIIAIV
Qrob_P0275560           NE-SIFGKKPEKFKVIVSSHNYHD-TPSVEDLSNLVARIQEAGADIVKIATTALEISDVAHIFQITVHSQV---PIIAIV
Juni_01730              ND-SIYGKKPEKFKVIVSSHNYLD-TPSVEDLGNLVARIQATGADIVKIATTAVEITDVARVFQITVHSQVRSVPIIGIV
JrDHQD-SDH              ND-SIYGRKPENSKVIVSSHNYQD-TPSAEDLGNLVARIQATGADIVKIATTALEIADVARIFQITVHSQV---PIIGIV
Juni_19979              ND-SIYGRKPENFKVIVSSHNYQD-TPSAEDLGNLVAIIQATGADIVKIATTALEIADVARIFQITVHSQV---PIIGVV
ArDHQ-SDH1              ND-SIYGKKPEKFKLIVSSHNYLD-TPSVEDLGNLVARIQATGADIVKIATTALEIIDVARVFQITVHSQV---PIIAMV
Alngl88491S37275        ND-SIYGKKPEKFKLIVSSHNYLD-TPSVEDLGNLVARIQATGADIVKIATTALEIIDVARVFQITVHSQV---PIIAMV
Bpev01_c0420-g0007      ND-SIYGKKPEKFKLIVSSHNYLD-TPSVEDLGNLVATIQSTGADIVKIATTASDITDVTRVFQITVHSQV---PIIAMV
Corav_882               ------------------------------------------------------------------------------MV
Casgl15S00444           ND-SICGKKPEKFKLIVSSHNYLD-TPPVEDLGNLVARIQATGADIVKIATTALEITDVTRVFQISVHSQV---PFIGMV
EgDHQD-SDH5             NA-SIQGRKPEKCKVIVSSHNYEN-TPSAEDLSNLVARIQAAGADIVKIATTALDITDVARMFHITVHSQVSSVPVIAMV
EcDHQD-SDH1             NA-SIQGRKPEKCKVIVSSHNYEN-TPSAEDLSNLVARIQAAGADIVKIATTALDITDVERMFHITVHSQVSSVPVIGMV
CsDHQD-SDH3             ND-SIRGKKPEKCKVIVSSHNYQY-TPSVEDLSNLVARIQASGADIVKFATTALDITDVARVFQITVHSQV---PIIGLV
PoptrDHQD-SDH1          NE-LLRGNKPGKCKLIVSSHNYEN-TPSVEELGNLVARIQAAGADIVKIATTALDISDVARIFQITVHSQVRSVPIIGLV
PoptrDHQD-SDH3          IW-ELKNKHQNGGKVIVSSYLNGA-TPSKENLSHLVATMQATEADIIKVVSNADDITEMERIFHLLSHCEV---PAVAYS
PoptrDHQD-SDH2          VR-EVKNKHQTGGKVIVSSYLSGA-TPSKEDLSHLVASMQATKADIIKVVSNANDITELDRIFHLLSHSEV---PAVAYS
EgDHQD-SDH4             MS-KQNKLHCGGTKVIVSCFLDGV-TPSKEELSNLATHMQATGADIIKIVTSASNITELARLFHILSYSQM---PVVAYA
EcDHQD-SDH4a            MS-KQNKLHCGGTKVIVSCFLDGV-TPSKEELSNLATHMKATGADIIKIVTSASNITELAGLFHLLSYSQM---PVVAYA
EcDHQD-SDH4b            MS-KQNKLHCGDTKVIVSCFLDGV-TPSKEELSNLATHMKATGADIIKIVTSASNITELARLFHLLSYSQM---PVVAYA
CasDHQD-SDH1            ME-EHNSNKHRSSKIIVSRYIDGT-TPSEEDLSQLVAHMQSTGADIIKLVSKSSSITELPRFFHLLSHCQI---PLITYS
SlDHQD-SDH2             AK-NEKSSWSSGCKLITSCFVDNV-T-SKEDLSQVVASMQSTGADILKIVINANDITELEKTFHLLSHCQV---PLIAYS
NtDHQD-SDH2             TK-KEKPRWSSGCKVIASCFVDNV-T-SKEDLSQVVAHMQSTGADILKIVTNANDITELEKMFHLLSHCQV---PLIAYS
CsDHQD-SDH1             LG-KQYSSHQSGTRFIVSCNLDCE-TPSEEDLGYLVSRMQATGADIIKLVFSVNDITEIARIFQLLSHCQV---PIIAYS
VvDHQD-SDH2             LG-KQKMDQHSSSRTIVSCYVDGV-TPPIEDLICRVALLQSTGADMIKLVINATNITEITKIFHLLSHCQM---PLIAYS
Qrob_P0208710           ME-ELKMNCHSGSKVIVSCYVNGV-TPLEEDLNRLVTSMQSTGADMIKLVINATNITEISRIFHLLSHCQV---PLIAYS
FSB015730401            ME-ELKMNCHSGSKVIVSCYVNGM-TPSEEYLSYLVANMQATRADIIKLVINATNITEITRIFHLLSHCQV---PLIAYS
Juni_23842              RG-ELKMDHHDGSKVIVSCYVNGM-TSLE-DLSHLVASMQATGADIIKLVTKAIDITEISRIFHLLSHCQV---PLIAYS
Bpev01_c1356-g0002      MG-ELRMDHHSGSKIIVSCYVNSM-DSLEEDLSYLVASMQATGADIIKLVTNATSITEISRIFRLLSHCQV---PLIAYS
Corav_4151              KG-ELRMNHHSGSKIIVSCYVNSM-ASLEEDLNHLVASMQTTGADIIKLVTNVTTITEIPRIFHLLSRCQV---PLIAYS
Alngl20613S19962        MG-ELRKNRHSGSKIIVSCYVNSM-ASLEEDLSHLVASIQTTGANIIKLVANATSITEISRIFHLLSHCQV---PLIAYS
ArDHQD-SDH2             MG-ELRKNRHSGSKIIVSCYVNSM-ASLEEDLSHLVSSIQTTGANIIKLVANATSITEISRIFHLLSHCQV---PLIAYS
CasDHQD-SDH2            NN-SISAKKPEKFKVIVSSQNFHS-TPSAEAIGNLVARIQATGADIVKIVTTALDITDVARIFQITVHSQI---PMIGIA
VvDHQD-SDH3             NN-SIYGKKPQNFKVIVSSHNFHN-TPSTEAIGNLVARIQASGADIVKIATTALDITDVARVLQVTVHSQV---PTIAIV
FvDHQD-SDH1             NN-SIYEKKPDNFKVIVSSHNFHN-TPSSEAIGNLVARIQATGADIVKIATTALDITDCARIFQITVHSQV---PTIGIV
EgDHQD-SDH3             NS-SILGKKPDNFKIIVSSHNFHN-TPSAEAIGNLVARIQATGADIAKIATTALDITDCARIFQITVHSQI---PIIGIV
EcDHQD-SDH3             NS-SILGKKPDNFKIIVSSHNFHN-TPSAEAIGNLVARIQATGADIAKIATTALDITDCARIFQITVHSQI---PIIGIV
Qrob_P0208720           TS-SINGKKPDNFKLIVSSHNFHN-TPSAEAIGNLVARIQATGADIVKIATTALDITDCARIFRIMVHSQV---PTIGIV
FSB015730501            NS-SIYGSKPENFKVIVSSHNFHN-TPSAEAIGNLVARIQATGADIVKIATTALDITDCARIFQIMVHSQV---PTIGIV
Corav_14055             NS-SIYGKKPENFKVIVSSHNFHN-TPSAEAIGNLVARIQATGADIVKIATTALDITDCARIFQIMVHSQV---PTIGIV
Corav_11016             NS-SIYGKKPENFKVIVSSHNFHN-TPSAEAIGNLVARIQATGADIVKIATTALDITDCARIFQIMVHSQV---PTIGIV
Bpev01_c1356-g0003      NS-SIYGKKPENFKVIVSSHNFHN-TPSAEAIGNLVARIQATGADIVKIATTALDITDCARIFQITVHSQV---PTIGIV
ArDHQD-SDH3             NS-SIYGKKPENFKVIVSSHNFHN-TPSAEAIGNLVARIQATGADIVKIATTALDITDCARIFQITVHSQV---PTIGIV
CasDHQD-SDH3            YK-LIQGKKPEKVKIIVSSHNYEN-TPSAEEIGNLAARIQATGADIVKIATTAQDITDSARIFQLLAHSQV---PTIGIV
DkDHQD-SDH              FS-SIQERSPKRPKVIVSSHNYEN-TPSAEEIGNIAARIQATGADIVKIATTALDITDSARILQLIAHSQV---PTIGLA
VvDHQD-SDH4             IN-SIQGKTSGKVKIIVSSHNYQN-TPSAEELGNLVARIQATGADIVKIATTALDITDCARIFQVLAHSQV---PTIGIA
EgDHQD-SDH2             FS-SIQGKKPEKAKIIVSSHNYQN-TPSSEELGNLVAKIQATGADIVKIATTALDISDCPRIFEVLAHSQV---PTIAIA
EcDHQD-SDH2             FS-SIQGKKPEKAKIIVSSHNYQN-TPSSEELGNLVAKIQATGADIVKIATTALDISDCPRIFEVLAHSQV---PTIAIA
PoptrDHQD-SDH5          YN-FIQGKKPEKVKIIVSSHNYEC-TPSIEEIGDLVARIQATGADIVKVATTALDITDNARMFHIIVNLQV---PMIGLV
```

```
FvDHQD-SDH2          YS-SIQGKKPERVKIIVSSHNYES-TPSAEEIGNLVATIQATGADIVKVATTALDITDNASIFQVLARSQV---PMIGLV
Casgl267S13432       YN-TIQGKKPQKVKIIVSSHNYQN-TPSLEEIGNLVARIQATGADIVKVATTGLDITDSARIFQILVHSQV---PMIGIV
Qrob_P0208730        YN-AIQGKKPEKVKIIVSSHNYQN-TPSVEEIGDLVAKILSTGADIVKVATTALDIVDSARVFQVLVHSQV---PMIGIV
FSB015730601         YN-TIQGKKPEKVKIIVSSHNYQN-TPSVEEIGNLVARILATGADIVKVATTGLDIVDSARIFQVLVHSQV---PMIGLV
Juni_23843           YN-AIQGKKPAKIKIIVSSHNYQN-TPSLEEIGNLVARIQATGADIVKVATTAVDITDSARIFQILVHSQV---PMIGIV
ArDHQD-SDH4          YN-TIQGKKPEKVKIIVSSHNYQN-TPSLEEIGNLVARILATGADIVKVATTAVDITDSARIFQILVHSQV---PMIGIV
Alngl6638S34349      ---GLPGKKPEKVKIIVSSHNYQN-TPSLEEIGNLVARILATGADIVKVATTAVDITDSARIFQILVHSQV---PMIGIV
Bpev01_c1356-g0004   YK-TIQGKKPEKVKIIVSSHNYQN-TPSLEEIGNLVARIQATGADIVKVATTAVDITDSARIFQVLVHSQV---PMIGLV
Corav_941            YN-TIQGKKPEKVKIIVSSHNYQN-TPSLEEIGNLVVRIQATGADIVKVATTAVDITDSARVFQILVHSQV---PMIGIV
Corav_7742           YN-TIQGKKPEKVKIIVSSHNYQN-TPSLEEIGNLVVRIQATGADIVKVATTAVDITDSARVFQILVHSQV---PMIGIV
SlDHQD-SDH3          VV-AELMKSRSNSKIIASTYVNGG-NPTKDTLCNSIINLQSTGADIIKLVIDVAYITDVAPVFHMLTHSQV---PLIVRA
EgDHQD-SDH1          NI-DELMEKRSSSKIIVSRHLNGD-KPCKEKLGNLIALMQSSGGDVIKLLVDVDYITDLAPIFQLLTSCQV---PLIATT
PoptrDHQD-SDH4       VM-AEYVYNRSNTKLIVSSYVNGR-KPSAEELGYLIACMQSTGADVLKLVLDVEKITDLAPVFTMLTHCQI---PLIALA
CsDHQD-SDH2          LM-SEIIYSRSNTKIIVSSYLNGGGKPTTEKLGDVIACMQATGADVMKLEIAVDSITDLAPVFEMLTHCQV---PLIALA
Qrob_P0339740        GM-AECMYKRANSKIIVSSFVNGG-KPSNERLGNLIACMQSTGADVIKLVIDVDYITDLAPVFQMLAHCQV---PLIAIA
Juni_24457           IVKAEHLYGRVNSRIIVSSYVNGG-KPSTEKLGHLIACMQSTGADVIKLVIDVDYITDLAPVFEMLTRCQV---PLIVIA
Casgl106S06827       GM-AEYVHKRAKSKIIVSTYVNGG-KPSTEKLGLLIANMQSTGADVIKLVINVNYITDLAPVFQMLTHCQV---PLIAVA
Bpev01_c0171-g0015   AM-AEHVYKRADSKIIVSSYVNGG-KPSTEKLGHLIACMQSTGADVIKLEINVDYITDLAPVFQMLTHCQV---PLIAVA
ArDHQD-SDH5          GM-AEHVYKRADSKLIVSSYVNGG-KPSTERLGHLIASMQSTGADVIKLEINVDYITDLAPVFQMLTYCQV---PLIAVA
ArDHQD-SDH6          GM-AEHVYKRADSKLIVSSYVNGG-KPSTERLGHLIASMQSTGADVIKLEINVDYITDLAPVFQMLTYCQV---PLIAVA

AtDHQD-SDH           MGERGLMSRILCSKFGGYLTFGTLDSSKVSAPGQPTIKDLLDLYNFRRIGPDTKVYGIIGKPVSHSKSPIVHNQAFKSVD
FvDHQD-SDH3          MGERGLISRILCAKFGGYFTSGTLHSGIVSVPGEPAIKDILDVYNFRLIRPCTKMFGVIGKPISHSKSPLLYNEGFKSVG
FvDHQD-SDH4          MGERGLISRILCAKFGGYLTFGTIDSGAVSAPGQPTIKDILNLYNFRQIGPDTKVFGIIGKPVSHSKSPILHNEGFKSVG
NtDHQD-SDH1          MGEKGLMSRILCPKFGGYLTFGTLEVGKVSAPGQPTIKDLLNIYNFRQLGPDTRIFGIIGKPVSHSKSPLLYNEAFRSVG
SlDHQD-SDH1          MGEKGLMSRILCPKFGGYLTFGTLEVGKVSAPGQPTVEDLLNLYNFRQLGPDTKIFGIIGKPVSHSKSPLLYNESFRSVG
CasDHQD-SDH4         MGERGLMARILCPKFGGYLTFGTLESGIVSAPGQPTMDDLVNLYNIRQIGPDTKVFGIIGKPVGHSKSPILYNEAFKKVG
VvDHQD-SDH1          MGERGLISRILCPKFSGYLTFGSLEPGIVSAPGQPTIKDLLNLYNFRQLGPDTKVFGVIGKPVSHSKSPHLYNEAFKSVG
FSB010265701         MGERGFISRILCPKFGGFLSFGTIESGIVSAPGQPTMKDLLHLYNFRQIGPDTKVFGIIGKPVGHSKSPILYNEAFKSVG
FSB012889301         MGERGFISRILCPKFGGFLSFGTIESGIVSAPGQPIMKDLLHLYNFRQIGPDTKVFGIIGKPVGHSKSPILYNEAFKSVG
Qrob_P0275560        MGERGFISRILCPKFGGFLSFGTIESGVVSAPGQPTMKDLLTLYNFREIGPDTKVFGIIGKPVGHSKSPILYNEAFKSVG
Juni_01730           MGEKGFISRILCPKFGGFLTFGTIESGIVSAPGQPTMKELLHLYNFRLIGPDTKLFGIIGKPVHHSKSPILYNEAFESVG
JrDHQD-SDH           MGERGFMSRILCPKFGGFLTFGTIESGIVSAPGQPTMKDLLHLYNLRRIGPDTKVFGIIGKPVHHSKSPILYNEAFKSVC
Juni_19979           MGERGFMSRILCPKFGGFLTFGTIESGIVSAPGQPTMKDLLHLYNLRRIGPDTKVFGIIGKPVHHSKSPILYNEAFKSVC
ArDHQ-SDH1           MGERGFLSRILSPKFGGFLTFGTIESGVVSAPGQPSMKDLLYLYNFRHIGPDTKVFGIIGKPVHHSKSPILYNEAFKSAG
Alngl88491S37275     MGERGFLSRILSPKFGGFLTFGTIESGVVSAPGQPSMKDLLYLYNFRHIGPDTKVFGIIGKPVHHSKSPILYNEAFKSAG
Bpev01_c0420-g0007   MGERGFASRILCPKFGGFLTFGTIESGIVSAPGQPTMKDLLYLYNFRQIGPDTKVFGIIGKPVHHSKSPILYNEAFKSAG
Corav_882            MGERGFISRILCPKFGGFLTFGTIESGIVSAPGQPTMKDLLYLYNFRDIGPDTKVFGIIGKPVHHSKSPILYNEAFKSVG
Casgl15S00444        MGDRGFISRILCPKFGGFLTFGTIESGIVSAPGQPSMKDLLYLYNFRQIGPDTKVFGIIGKPVYHSKSPLLYNEAFKSVG
EgDHQD-SDH5          MGERGLISRVLCAKFGGFLTFGTLESGVVSAPGQPTIKDLLDLYNFRSIGPDTKVFGIIGKPVGHSKSPLLYNQAFKSAG
EcDHQD-SDH1          MGERGLISRVLCAKFGGFLTFGTLESGVVSAPGQPTIKDLLDLYNFRSIGPDTKVFGIIGKPVGHSKSPLLYNQAFKSAG
CsDHQD-SDH3          MGERGLISRILCAKFGGFLTFGTLENGIVSAPGQPTIKDLLDLYNFRQMGPDTKVFGIIGKPVGHSKSPILYNEAFKSVG
PoptrDHQD-SDH1       MGERGLISRILCAKFGGYLTFGTLESGVVSAPGQPTIKDLLDLYNFRLIGPDTKVFGIIGKPVGHSKSPVLFNEAFKSVG
PoptrDHQD-SDH3       VGERGLISQLLCPKFGGALVYGSMEGN--SIPGLPTLDSLREAYKVDCINSDTKVFGLVSKPVGHSKGPLLHNPTLRHVN
PoptrDHQD-SDH2       LGERGLISQLLCPKFGGALVYGAMEGN--SIPGLPTLDSLREAYKVENINSDTKVFGLVSKPVSHSKGPILHNPAFRHAN
EgDHQD-SDH4          VGERGLISQLLSPKFGSNLVYGSIEGM--AVPGLPTLESLRKAYKVEHINSDTKVFGLISKPVGHSKGPILHNPTLRHMN
EcDHQD-SDH4a         VGERGLISQLLSPKFGSTLVYGSIEGM--AVPGLPTLESLRKTYKVEHINSDTTVFGLISKPVGHSKGPILHNPTLRHMN
EcDHQD-SDH4b         VGERGLISQLLSPKFGSTLVYGSIEGM--AVPGLPTLESLRKTYKVEHINSDTTVFGLISKPVGHSKGPILHNPTFRHMN
CasDHQD-SDH1         IGDRGLISQLLGPKFDGFLIYGSIEGN--PVPGLPTLHNLRQAYGVDYLNAETKVFGLISKTSGPQQRSPSPYPTFRHVG
SlDHQD-SDH2          VGERGLISQLLGPKFGSVLLYGSLDGN--AVPGLPSLASLRQAYGVDLMDNDTKVFGLISKPVGHSKGPILHNPTFRHVG
NtDHQD-SDH2          LGERGLISQLLGPKFGSVLVYGSLDCN--AVPGLPTLGSLRQAYGVDFMDTDTKVFGLISKPVGHSKGPILHNPTFRHVG
CsDHQD-SDH1          VGERGLVSQLLSPKFNGALVYGSLKGT--PVLGLPTVESLRQTYKVEHINADTKVFGLISKPVGHSKGPILHNPTFRHVN
VvDHQD-SDH2          IGDRGFMSQILCRKFGGFLVYGSMEGS--PVAGLPTLESLREAYKVQYINKDTKVFGLISKPVGHSKGPILHNPAFRHVN
Qrob_P0208710        VGERGLISQILCPKFGGFLVYGSMEGN--SVPGLPTLENLRQAYKIEDIDEDTKVFGLISKPVGHSKGPLLHNPTFRRVK
FSB015730401         VGERGLISQILCPKYGGFLVYGSMEGN--SIPGLPTLDSLRQAYKVDNIDADTTVFGLISKPVGHSKGPLLHNPTFTHVK
Juni_23842           VGERGLMSQILSPKFGGLLVYGSMEGN--SVPGLPTLDSLRQAYKVIHINADTKVFGLISKPVSHSKGPLLHNPTFRHVN
Bpev01_c1356-g0002   AGERGLISQILCRKFGGFLVYGSIKGY--SFPGLPALDSLRQAYKVDYVNADTKVFGLISKPVSHSKGPLLHNPTFRHVN
Corav_4151           EGERGLISQLLCRKFGGFLVYGSIKGN--SDPGLPTLDGLIQAYKVDNVNADTKIFGLISKPVGHSKGPLLHNPTFRHVN
Alngl20613S19962     VGERGLISQILCRKFGGFLAYGSIEGN--SVPGLPTLDSLRQAYKVEYVNEDTKVFGLISKPVGHSKGPLLHNPTFRHVN
ArDHQD-SDH2          VGERGLISQILCRKFGGFLAYGSIEGN--SVPGLPTLDSLRQAYKVEYVNEETKVFGLISKPVGHSKGPLLHNPTFRHVN
CasDHQD-SDH2         MGERGLISRLLSPKFNGYLTYAALEAGAISAPGQPTVKDLLDLYNFRLIRPDTKVYGIIGKPVGHSKSPLLFNESFKKVG
VvDHQD-SDH3          MGERGLISRLLSPKFGGYLTYGALEAGAISAPGQPTAKDLLDLYNFRLVKPDTKVYGIIGKPVGHSKSPLLFNAAFKKVG
FvDHQD-SDH1          MGERGLISRLLSPKFGGYLTYGALEAGAISAPGQPTARDLLDLYNFRLIRPDTKIYGIIGKPVGHSKSPLLFNAAFKSVG
EgDHQD-SDH3          MGERGLISRILSPKFGGYLTYGALEAGAISAPGQPTAKDLLDLYNFRLIRPDTKVYGIIGKPVGHSKSPLLFNASYKSVG
EcDHQD-SDH3          MGERGLISRILSPKFGGYLTYGALEAGAISAPGQPTAKDLLDLYNFRLIRPDTKVYGIIGKPVGHSKSPLLFNASYKSVG
Qrob_P0208720        MGERGLISRLLSPKFGGYLTYGALEAGAISAPGQPTAKDLLDLYNFRLIGPDTKVYGIIGKPVGHSKSPLLFNAAFKSVG
FSB015730501         MGERGLISRLLSPKFGGYLTYGALEAGAISAPGQPTAKDLLDLYNFRLITPDTKVYGIIGKPVGHSKSPLLFNAAFKSVG
Corav_14055          MGERGLISRLLSPKFGGYLTYGALEAGAISAPGQPTAKDLLDLYNFRFIRPDTKVYGIIGKPVGHSKSPLLFNAAFKSVG
Corav_11016          MGERGLISRLLSPKFGGYLTYGALEAGAISAPGQPTAKDLLDLYNFRFIRPDTKVYGIIGKPVGHSKSPLLFNAAFKSVG
Bpev01_c1356-g0003   MGERGLISRLLSPKFGGYLTYGALEAGAISAPGQPTAKDLLDLYNFRLIRPDTKVYGIIGKPVGHSKSPLLFNAAFKTVG
```

```
ArDHQD-SDH3          MGERGLISRLLSPKFGGYLTYGALEAGAISAPGQPTAKDLLDLYNFRLIRPDTTVYGIIGKPVGHSKSPLLFNAAFKSVG
CasDHQD-SDH3         MGERGLMSRILCAKFGGFLTFGALESGIVSAPGQSTLKDLLELYNFRQIGPDTKVHGVIGNPIGHSKSPHLYNTAFRSAG
DkDHQD-SDH           MGERGLISRILCAKFGGFLTFGALESGIHSAPGQPTLRDLLDLYNFRQIEPDTKVHGVIGNPIGHSKSPHLYNTAFKKAA
VvDHQD-SDH4          MAERGLISRILSAKFGSYLTFGSLEAGVVSAPGQPTVKDLLDLYNFRQIGPDTKVHGVIGKPIGHSKSPHLYNSAFKSVG
EgDHQD-SDH2          MGERGLISRILAAKFGGYLTFGAIEAGVVSAPGQPSIKDLLDLYNLRQLGPDTKVHGVIGKPIGHSKSPHLYNAAFRSVN
EcDHQD-SDH2          MGERGLISRILAAKFGGYLTFGAIEAGVVSAPGQPSIKDLLDLYNLRQLGPDTKVHGVIGKPIGHSKSPHLYNAAFKSVN
PoptrDHQD-SDH5       MGERGLMSRVLAAKYGAFLTFGSIEAGVVSAPGQPTVKDLLELYNLRQIEADTKVHGVIGNPIGHSKSPHLYNAAFKSVG
FvDHQD-SDH2          MGDKGLISRVLSAKYGSFATFGTLVAGEVSAPGQPTVTDLLELYNFRQIGADTKVHGVIGNPIGHSKSPHLYNAAFKANN
Casgl267S13432       MGDKGLISRILSAKFGGFLTFGSIEPGVTSAPGQPSIKDLLDLYNFRQIGPDTKVHGVIGNPIGHSKSPHLYNPAFKSVN
Qrob_P0208730        MGEKGLMSRILSAKFGGFLTFGSIEAGVISAPGQPTVTDLLDLYNFRQIGPDTKVHGVIGNPIGHSKSPHLYNGAFKSVN
FSB015730601         MGEKGFISRILSAKFGGFLTFGSIE-----------------------IGPDTKVHGVIGNPIGHSKSPHLYNGAFKSVN
Juni_23843           MGEKGFISRILSAKFGGFLTFGSIEAGLVSAPGQPTVKELLDLYNFRQLGPDTKVHGVIGNPIGHSKSPHLYNPAFKSVN
ArDHQD-SDH4          MGERGFISRILSAKFGGFLTFGSIEAGVVSAPGQPSIKDLLDLYNFRLLGPDTKVHGVIGNPIGHSKSPHLYNPAFKSVN
Alngl6638S34349      MGERGFISRILSAKFGGFLTFGSIEAGVVSAPGQPSIKDLLDLYNFRLLGPDTKVHGVIGNPIGHSKSPHLYNPAFKSVN
Bpev01_c1356-g0004   MGDRGFISRILSAKFGGFLTFGSIEAGVVSAPGQPSIKDLLDLYNFRLLGPDTKVHGVIGNPIGHSKSPHLYNPAFKSVN
Corav_941            MGDRGFISRILSAKFGGFLTFGSIEAGVVSAPGQPSIKDLLELYNFRQLGPDTKVHGVIGNPIGHSKSPHLYNPAFKSVN
Corav_7742           MGDRGFISRILSAKFGGFLTFGSIEAGVVSAPGQPSIKDLLELYNFRQLGPDTKVHGVIGNPIGHSKSPHLYNPAFKSVN
SlDHQD-SDH3          AGDRGLISQLLGPKYGAFFVCGSLGGK--YTPGLPSLTTIKQVYKLQYVNPDTRIFGVISNPVGHSKGPLLHNPAFRHTG
EgDHQD-SDH1          VGDRGLIGQLLGPKFGGFLAYGSLEG---AIPGLPTLTSLRQVYKIEHMNADTKVFGLISNPVGHSKGPLLHNPTFRYVG
PoptrDHQD-SDH4       VGSRGLISQLLGPKFGGFLVYGSLSDK--AVPGMPTLLSLRQIYKLEYINADTKVFGLISNPVGHSKGPVLHNPAFRHTG
CsDHQD-SDH2          VGSRGLISQLLGPKFGGFLVYGSLGGK--SVPGLPTLVSLKQVYQLEHINPDTKIFGLVSNPVGHSKGPILHNPAFRHTR
Qrob_P0339740        VGSRGLISQLLGPKFGGFLVYGSLEGK--SVPGLPTLVSLKHVYKLEYLNADTKVFGLISNPVGHSKGPILHNPAFRHTG
Juni_24457           AGSRGLISQLLGPKFGGFLVYGSLECK--SIPGLPTLLSIKHVYKLEHLNADTKVFGLISNPVGHSKGPILHNPAFRHTG
Casgl106S06827       VGSRGLISQLLGPKFGGFLVYGSLEGK--SVPGLPTLVSLKHVYQLQYINAETKVFGLISNPVGHSKGPILHNPAFRHTG
Bpev01_c0171-g0015   VGSRGLISQLLGPKFGGFLVYGSLEGK--SVPGLPSLVSLKHVYRLEYMNADTKVFGVISNPVGHSKGPILHNPAFRHTG
ArDHQD-SDH5          VGSRGLISQLLGPKFGGFLVYGSLEGK--SVPGLPTLVSLKHVYRLEYMNADTKVFGLISNPVGHSKGPILHNPAFRHTG
ArDHQD-SDH6          VGSRGLISQLLGPKFGGFLVYGSLEGK--SVPGLPTLVSLKHVYRLEYMNADTKVFGLISNPVGHSKGPILHNPAFRHTG
```

381

```
AtDHQD-SDH           FNGVYVHLLVDNLVSFLQAYSSSDFAGF------------SCTIPHKEAALQCCDEVDPLAKSIGAVNTILRRKSDGKLL
FvDHQD-SDH3          FDGVYLHLLVDDIANFLHTYSSTDFSGF------------SVGIPHKEAALKCCDEVDPIAKSIGAVNCIVRRPTDGKLF
FvDHQD-SDH4          FNGVYVHLLVDDIANFLKTYSSADFAGF------------SVTIPHKEAALKCCDEVDPVAKSIGAINCIIRRPNDGKLY
NtDHQD-SDH1          FNGVYMPLLVDDVANFFRTYSSLDFAGS------------AVTIPHKEAIVDCCDELNPTAKVIGAVNCVVSRL-DGKLF
SlDHQD-SDH1          FNGVFMHLLVDDIANFFRTYSSLDFAGF------------SCTIPHKEAALDCCAEIDPTAKAIGAVNCIIRRP-DGKLF
CasDHQD-SDH4         FNGVYLHLLVDDVANFLQTYSSTDFAGF------------SCTIPHKEAALKCCDEVDPVAKSIGAVNCIVRKS-DGKFF
VvDHQD-SDH1          FNGVYVHLLVDDIAKFFHTYSAADFAGF------------SCTIPHKEAALKCCDEVSPVAKSIGAVNCIIRRPSDGKLF
FSB010265701         FNGVYVPFLVDDIAKFLQTYSSSDFAGF------------SITIPHKEAALKCCDEVNPVAKSIGAVNCIVRRSTDGKIF
FSB012889301         FNGVYVPFLVDDIAKFLQTYSSSDFAGF------------SITIPHKEAALKCCDEVNPVAKSIGAVNCIVRRSTDGKIF
Qrob_P0275560        FNGVYVPFLVDDVAKFLQTYSSIDFAGF------------SCTIPHKEDALKCCDEVDPVAKSIGAVNCVIRRPTDGKMF
Juni_01730           FNGVYVPLLVDDVANFFKTYSSADFSGF------------SCTIPHKEAALKCCDEVDPVAKSIGAVNCIIRRPTDGKLI
JrDHQD-SDH           FNGVYIPLLVDDIANFLQTYSSTDFAGF------------SCTIPHKEAALKCCDEVDPVAKSIGAVNCIIRRPTDGKLV
Juni_19979           FNGVYIPLLVDDIANFLRTYSSTDFAGF------------SCTIPHKEAALKCCDEVDPVAKSIGAVNCIIRRPTDGKLV
ArDHQ-SDH1           FNGVYVPLLVDDVSTFLQTYSTSDFAGF------------SCTIPHKEAALKCCDEVDPVAKSIGAVNCIVRRPSDGKLF
Alngl88491S37275     FNGVYVPLLVDDVSTFLQTYSSSDFAGF------------SCTIPHKEAALKCCDEVDPVAKSIGAVNCIVRRPSDGKLF
Bpev01_c0420-g0007   FNGVYVPLLVDDVSTFLQTYSSSDFAGF------------SCTIPHKEAALKCCDEVDPVAKSIGAVNCIIRRPADGKLF
Corav_882            FNGVYVPLLVDDVSTFLRTYSSTDFAGF------------SCTIPHKEAALKCCDEVDPVAKSIGAVNCIIRRPTDGKLF
Casgl15S00444        FDGVYVPLLVDDVSNFLRTYSSTDFAGFRFIFFV-EHYESSCTIPHKEAALKCCDEVDPVAKSIGAVNCIIRRPTDGKLF
EgDHQD-SDH5          FDGVFLHLLVDDVASFLQTYSSTDFAGF------------SCTIPHKEAAVKCCDEVDPVAKSIGAVNCIIRRQSDAKLF
EcDHQD-SDH1          FDGVFLHLLVDDVASFLQTYSSTDFAGF------------SCTIPHKEAAVKCCDEVDPVAKSIGAVNCIIRRQSDAKLF
CsDHQD-SDH3          FNGVFVHLLVDDIAKFFQTYSSNDFAGF------------SCTIPHKEAAVKCCDEVDTVAKSIGAVNCIIRRQSDGKLF
PoptrDHQD-SDH1       INGVYVHLLVDDIARFLQTYSSTDFAGF------------SCTIPHKEDAAKCCDEVHPVAKSIGAVNCIIRRQNDGKLF
PoptrDHQD-SDH3       FNGIYVPMFVDDLKKFFDVYASPDF------------AGYSVGFPYKEAVVQFCDEVHPLAKCIGAVNTIIRRPCDGKLI
PoptrDHQD-SDH2       FNGIYVPMFVDDLKEFFEVYASPDF------------AGYSVGFPYKEAVVQFCDEVHPLAKSIGAVNTIIRKPSDGKLI
EgDHQD-SDH4          FNGLYVPMFVDNLKEFFEVYSTPDF------------AGFSVGIPYKEAVIQFCDEVHPLAQNIGAVNTIVRRPSDGKLI
EcDHQD-SDH4a         FNGLYVPMFVDNLKEFFEVYSTPDF------------AGFSVGFPYKEAVIQFCDEVHPLAQTIGAVNTIVRRPSDGKLI
EcDHQD-SDH4b         FNGLYVPMFVDNLKEFFEVYSTPDF------------AGFSVGFPYKEAVIQFCDEVHPLAQTIGAVNTIVRRPSDGKLI
CasDHQD-SDH1         YNGIYVPMLVDDLKEFFSVYSSPDY------------AGFSVGFPYKETVTAFCDEVDPLAQSIGAVNTIIRRHSDGKLV
SlDHQD-SDH2          YNGIYVPMFVDDLKEFFRVYSSPDF------------AGFSVGIPYKEAVVSFCDEVDPLAESIGAVNTIIRRPCDGKLI
NtDHQD-SDH2          YNGIYVPMFVDDLKEFFRVYSSPDF------------AGFSVGIPYKEAVVSFCDEVDPLAKSIGAVNTIIQRPCDGKLI
CsDHQD-SDH1          YNGIYVPMFVDDLKKFFSTYSSPDF------------AGFSVGFPYKEAVMKFCDEVHPLAQAIAAVNTIIRRPSDGKLI
VvDHQD-SDH2          YNGIYVPMLVDDLKEFFSIYSSPDF------------AGFSVGIPYKEAVTGFCDELHPLAQSIGAVNTIMRRPSDGKLI
Qrob_P0208710        YNAIYVPMFVDDLKDFFNVYSSPEF----------EFAGFSVGFPYKEAVVEFCDEVHPLAKAIGAVNTIIRRPSDGKWV
FSB015730401         YNAVYVPMFVDDLKDFFSAYSSPDFAGFSITSRFLDSCEYSVGIPYKEAVIGFCDEVHPLAKEIGAANTIIRRPSDGKLV
Juni_23842           YNGIYVPMFVDNLKDFFSVYSSPDF------------AGFSVGIPYKEAVIGFCDEVHPLAKAIGAVNTIIRRPTDGKLV
Bpev01_c1356-g0002   YNGIYVPMFVDDLRDFFSVYSSADF------------AGFSVGFPYKEAVVGFCDEVHPLAQAIGAVNTIVRRPSDGKLI
Corav_4151           YNGIYVPMFVDDLRDFFSVYSSAEY------------AGFSVGFPYKEAIIGFCDEVHPLAQAISAVNTIIRRPRDGKLI
Alngl20613S19962     YNGIYVPMFVDDLRDFFSVYSTADF------------AGFSVGFPYKEAIFGFCDEVHPLAQAIGAVNTIVRRPSDGKLI
ArDHQD-SDH2          YNGIYVPMFVDDLRDFFSVYSTADF------------AGFSVGFPYKEAIFGFCDEVHPLAQAIGAVNTIVRRPSDGKLI
CasDHQD-SDH2         LNSVYVHLLVDDVKKFFNTYSSVDFA-GF-----------SCTIPHKEVALECMDEIDPIAKKIGAINNIVRRP-DGTLK
```

```
VvDHQD-SDH3           LNAVYVHLLVDDVEKFFNTYSAPDFISGC-----------SCTIPHKEVAIKCMDTIDPIARKIGAINNIVRKP-DGKLT
FvDHQD-SDH1           LNAVYVHFLVDDVEKFFNTYSSVDFS-GC-----------SCTIPHKEAALKCMDEIDSIAKKIGAINNIVRKP-DGRLV
EgDHQD-SDH3           LNAVYLHFLVDDVEKFFNTYSAVDFASGC-----------SCTIPHKEAALKCMDEIDPIAKKIGAINNIVRRP-DGTLT
EcDHQD-SDH3           LNAVYLHFLVDDVEKFFNTYSAVDFASGC-----------SCTIPHKEAALKCMDEIDPIAKKIGAINNIVRRP-DGTLT
Qrob_P0208720         LDAVYVHYLVDDVEKFFNTYSSIDWS-GC-----------SCTIPHKEAALKCMDEIDPIAKKIGAINNIVRRP-DGKLI
FSB015730501          LDAVYVHYLVDDVEKFFNTYSSLDWC-GC-----------SCTIPHKEAALKCMDDIDPIAKKIGAINNIVRRP-DGKLT
Corav_14055           LNAVYVHFLVDDVKKFFDTYSTIDWA-GC-----------SCTIPHKEAALRCMNEIDPIAKKIGAINNIVRRP-DGTLT
Corav_11016           LNAVYVHFLVDDVKKFFDTYSTIDWA-GC-----------SCTIPHKEAALRCMNEIDPIAKKIGAINNIVRRP-DGTLT
Bpev01_c1356-g0003    LNAVYVHYLVDDVKKFFDTYSSPDWA-GC-----------SCTIPHKEAALKCMNEIDPIAKKIGAINNIVRRP-DGTLT
ArDHQD-SDH3           LNAVYVHYLVDDVKKFFDTYSSPDWA-GC-----------SCTIPHKEAALKCMNEIDPIAKKIGAINNIVRRP-DGTLT
CasDHQD-SDH3          FNGIYLPLLVDSVANFFNTYSSPDFV-GY-----------SYTIPHKEAGPKCCDEVDPIAKAIGAISCMVRKPSDGKLI
DkDHQD-SDH            FNGIYLPLLVDNVANFLNTYSSPDFT-GY-----------SYTIPHKMAGLECCDEVDPIAKAIGAISCMIKKPSDGKLI
VvDHQD-SDH4           FNGIYLPLLVDSVKNFLATYSSPDFV-GY-----------SYTIPHKEDGLRCCDEIDPIAQAIGAISCMIRRPADGKLM
EgDHQD-SDH2           FNGIYLPLLVDNVANFINAYSSPDFV-GY-----------SYTIPHKEDGLRCCDEVDPIAKAIGAISQMIRRPTDGKMI
EcDHQD-SDH2           FNGIYLPLLVDNVANFINAYSSPDFV-GY-----------SYTIPHKEDGLRCCDEVDPIAKAIGAISQMIRRPTDGKMI
PoptrDHQD-SDH5        FNGIYLPLLVDSVANYISTYSSPDFA-GY-----------SYTIPHKEDGLKCCDEVDPIAKEIGAISCMIRRPDDGKLK
FvDHQD-SDH2           FNGIYLPLLVDSVANFINTYNSPDWV-GY-----------SYTIPHKEAGFKCCDEIDPNALAIGAISCMIRNPTDGKLK
Casgl267S13432        FNGIYLPLLVDNVANFLKAYSSHDFV-GY-----------SYTIPHKEAGFNCCDEIDPIAKEIGAISCMIKRP-DGNLM
Qrob_P0208730         FNGIYLPLLVDNVANFLNTYSSPDFV-GY-----------SYTIPHKEAGFNCCDEIDPIAKEIGAISCMIRRP-DGKLK
FSB015730601          FNGIYLPLLVDNVANFLNTYSSPDFV-GY-----------SYTIPHKEAGFNCCDEIDPIAKEIGAISCMIRRP-DGKLK
Juni_23843            FDGIYLPLLVDNVANFLKTYSSHDFV-GY-----------SYTIPHKEAGFNCCDEIDPIAKEIGAISCMIKRP-NGNLM
ArDHQD-SDH4           FNGIYLPLLVDNVANFLKTYSSPDFV-GY-----------SYTIPHKEAGFNCCDEIDPIAKEIGAISCMIKRP-DGNLM
Alngl6638S34349       FNGIYLPLLVDNVANFLKTYSSPDFV-GY-----------SYTIPHKEAGFNCCDEIDPIAKEIGAISCMIKRP-DGNLM
Bpev01_c1356-g0004    FNGIYLPLLVDNVANFLKTYSSPDFV-GY-----------SYTIPHKEAGFNCCDEIDPIAKEIGAISCMIKRP-DGNLM
Corav_941             FNGIYLPLLVDNVANFLKTYSSHDFV-GY-----------SYTIPHKEAGFNCCDEIDPIAKEIGAISCMIKRP-DGRLM
Corav_7742            FNGIYLPLLVDNVANFLKTYSSHDFV-GY-----------SYTIPHKEAGFNCCDEIDPIAKEIRTRLRLL---------
SlDHQD-SDH3           YNGIYVPLLVDNVKEFFRVFSCNDY------------AGFSVGIPHKEAAVRCCDEVDPLAKSIGAVNTIIRRPSDGKLI
EgDHQD-SDH1           FNGIYVPMLVDNIKEFFKVYTSQDF------------AGFSVGIPHKEAAVACCDEVHPLAKSIGAVNTIVRRPTDGKLI
PoptrDHQD-SDH4        YNGIYVPMQVDDVKEFFRTYTSSDF------------AGFSVGIPHKEAAVGCCDEVHPLAKSIGAVNTIVRRPTDGKLV
CsDHQD-SDH2           FNGIYVPMLVDDVKEFFRTYSGTDF------------AGFSVGIPHKEPAVACCDEVHPLAKSIGAVNTIIRRPIDGKLV
Qrob_P0339740         YNGIYVPMLVDDIKEFFRTYTGTDF------------AGFSVGIPHKEAAVGCCDEVHPLAKSIGAVNTIVRRPIDGKLI
Juni_24457            YNGIYVPMLVDDIKEFFETYTGTDF------------AGFSVGIPHKEAAVGCCDEVHPLAKSIGAVNTIVRRPIDGKLI
Casgl106S06827        YNGIYVPMLVDDIKEFFKTYTGTDF------------AGFSVGIPHKEAAAGCCDEVHPLAKSIGAVNTIIRRPIDGKLI
Bpev01_c0171-g0015    YNGIYVPMLVDDINEFFQTYTGTDF------------AGFSVGIPHKEAAVGCCDEVHPLAKSIGAVNTIIRRPIDGKLI
ArDHQD-SDH5           YNGIYVPMLVDDIKEFFETYTGTDF------------AGFSVGIPHKEAAVGCCDEVHPLAKSIGAVNTIIRRPIDGKLI
ArDHQD-SDH6           YNGIYVPMLVDDIKEFFETYTGTDF------------AGFSVGIPHKEAAVGCCDEVHPLAKSIGAVNTIIRRPIDGKLI
```

```
AtDHQD-SDH            GYNTDCIGSISAIEDGLRSSG--------------------DPSSVPSSSSPLASKTVVVIGAGGAGKALAYGAKEKGAK
FvDHQD-SDH3           GLNTDYFGSISAIEDGLRGSH--------------------D--KSNIIGSPLAGRLFVIMGAGGAGKALAYGAKQKGAR
FvDHQD-SDH4           GLNTDYVGAISAIEDGLQGSL--------------------N--GSHVTGSPLAGRLFVVIGAGGAGKALAYGAKQKGAR
NtDHQD-SDH1           GCNTDYVGAISAIEEALQGSQ--------------------P----SMSGSPLAGKLFVVIGAGGAGKALAYGAKEKGAR
SlDHQD-SDH1           GCNTDYIGAISAIEEGLQGSQ--------------------P----SISGSPLAGKLFVVIGAGGAGKAIAYGAKEKGAR
CasDHQD-SDH4          GCNTDYVGAITAIEDALGGLQ--------------------H--VNGMVVSPLSGKLFVVIGAGGAGKALAYGAKAKGAK
VvDHQD-SDH1           GYNTDYVGAISAIEDGLRDLH--------------------K--ISSTSGSPLAGKLFVVIGAGGAGKALAYGAKEKGAR
FSB010265701          GYNTDYVGAISAIEDGLRGSK--------------------N--ISYAADSPLAGKLFVVIGAGGAGKALAYGAKEKGAR
FSB012889301          GYNTDYVGAISAIEDGLRGSK--------------------N--ISYAADSPLAGKLFVVIGAGGAGKALAYGAKEKGAR
Qrob_P0275560         GYNTDYVGAISAIEDGLRGSH--------------------N--ISATADSPLAGKLFVVIGAGGAGKALAYGAKEKGAQ
Juni_01730            GYNTDYVGAISAIEDGLRGSH--------------------N--SNNTGDSPLAGKLFVVIGAGGAGKALAYGAKEKGAR
JrDHQD-SDH            GYNTDYVGAISAIEDGLRGSH--------------------N--SSNTADSPLAGKLFVVIGAGGAGKALAYGAKEKGAR
Juni_19979            GYNTDYVGAISAIEDGLRGSH--------------------N--SSNTADSPLAGKLFVVIGAGGAGKALAYGAKEKGAR
ArDHQ-SDH1            GCNTDYVGAISAIEDGLRGSH--------------------N--ISNTADSPLAGKLFVVIGAGGAGKALAYGAKEKGAR
Alngl88491S37275      GCNTDYVGAISAIEDGLRGSH--------------------N--ISNTANSPLAGKLFVVIGAGGAGKALAYGAKEKGAR
Bpev01_c0420-g0007    GCNTDYVGAISAIEDGLRGSH--------------------N--ISNTADSPLAGKLFVVIGAGGAGKALAYGAKEKGAR
Corav_882             GCNTDYVGAISAIEDGLRGSH--------------------N--ISNTADSPLAGKLFVVIGAGGAGKALAYGAKEKGAR
Casgl15S00444         GYNTDYVGAISAIEDGLRGSH--------------------NN-ISNTADSPLAGKLFVVIGAGGAGKALAYGAKEKGAR
EgDHQD-SDH5           GYNTDYVGAISAIEDGLRGSQ--------------------N--GNSAGASPLNGKLFVVIGAGGAGKALGYGAKEKGAR
EcDHQD-SDH1           GYNTDYVGAISAIEDGLRGSQ--------------------N--GNSAGASPLNGKLFVVIGAGGAGKALGYGAKEKGAR
CsDHQD-SDH3           GYNTDYVGAISAIEDGLRGRL--------------------N--VSGGVSSALAGKLFVVIGAGGAGKALAYGAKAKGAR
PoptrDHQD-SDH1        GYNTDYVGAISAIEEGLRASQ--------------------N--VSNTVGSPLAGKLFVVIGAGGAGKALAYGAKEKGAR
PoptrDHQD-SDH3        GYNTDCEGSITAIEDALRD--------------------QKYVNG-RSLNSPLAGKQFVVVGAGGAGRAIAVGAKSRGAR
PoptrDHQD-SDH2        GYNTDCEGSIASIEDALKD--------------------QRYING-ASLNSPLAGKQFVVVGAGGAGRAIAVGAKSRGAR
EgDHQD-SDH4           GYNTDCEASVTAMEDALQE--------------------CRCINGEKSLVSPLAGKEFVLVGAGGAGRALAFGAKTRGAR
EcDHQD-SDH4a          GYNTDCEASVTAMEDALQE--------------------CRCINGEKSLVSPLAGKEFVLVGAGGAGRALAFGAKTRGAR
EcDHQD-SDH4b          GYNTDCEASVTAMEDALQE--------------------CRCINGEKSLVSPLAGKEFVLVGAGGAGRALAFGAKTRGAR
CasDHQD-SDH1          GYNTDCEASITAIEDALKV--------------------WGCTNGEVSLPSPLTGKMFVLVGAGGAGRALAFGAKSRGAR
SlDHQD-SDH2           GYNTDCEASITAIEDALK------------------------ANGEALVPCSLARKMFVLVGAGGAGRALAFGAKSRGAR
NtDHQD-SDH2           GYNTDCEASITAIEDALKV--------------------NGLTNGAAFLPSPLAGKLFVLVGAGGAGRALAFGAKSRRAE
CsDHQD-SDH1           GYNTDCEASITAIEDAIKE--------------------RGYKNGTASFGSPLAGRMFVLAGAGGAGRALAFGAKSRGAR
VvDHQD-SDH2           GYNTDCEASITAIEDALRE--------------------RGLPNGEAPLNSPLTGKQFVLVGAGGAGRALAFGARSRGAQ
Qrob_P0208710         GYNTDREASITAIEDALKH--------------------QGYTNGETSFDSPLAGKQFVLAGAGGAGRALAVGAKSRGAR
```

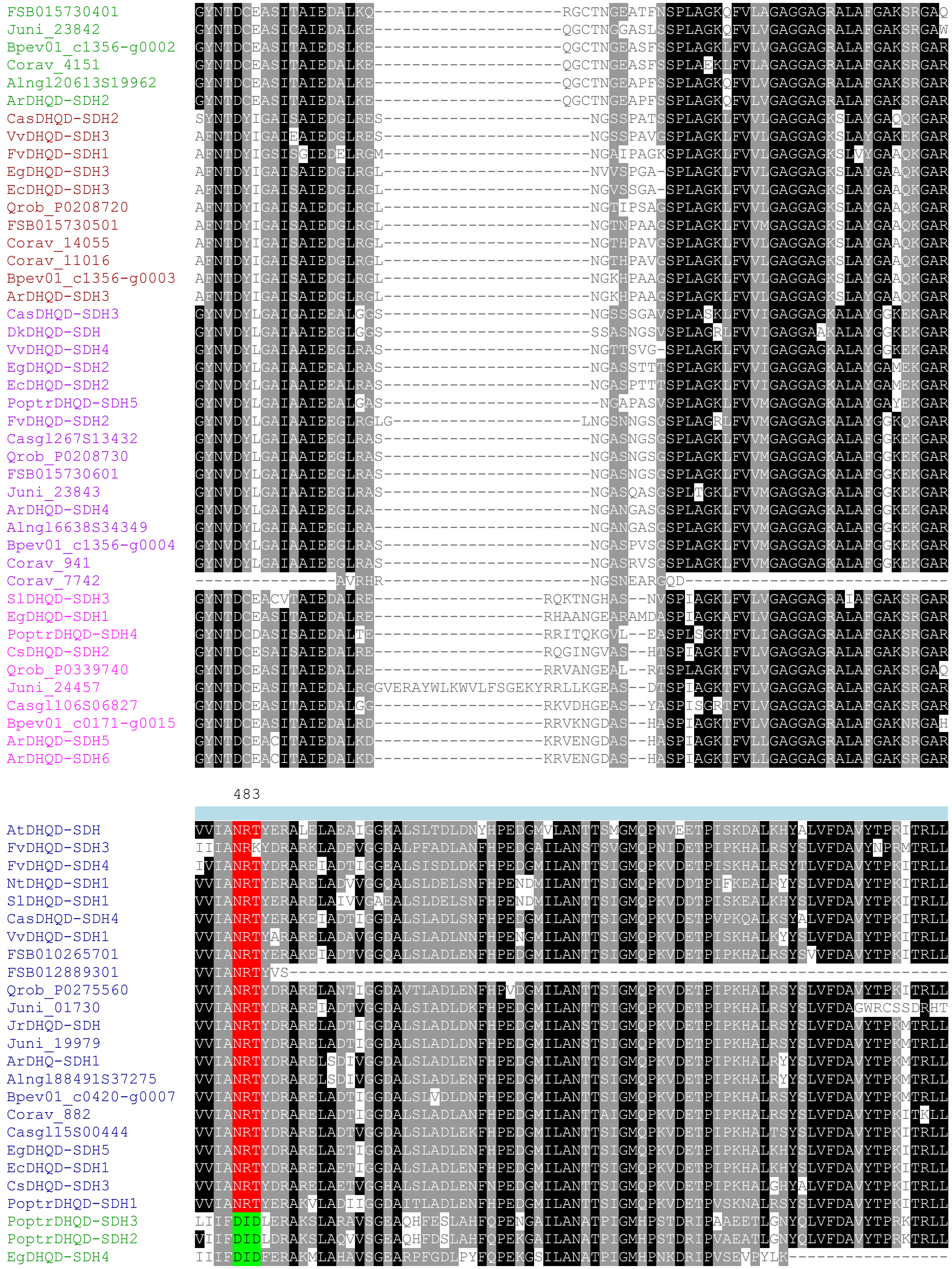

FSB015730401 GYNTDCEASITAIEDALKQ--------------------RGCTNGEATFNSPLAGKQFVLAGAGGAGRALAFGAKSRGAQ
Juni_23842 GYNTDCEASICAIEDALKE--------------------QGCTNGGASLSSPLAGKQFVLVGAGGAGRALAFGAKSRGAW
Bpev01_c1356-g0002 GYNTDCEASITAIEDSLKE--------------------QGCTNGEASFSSPLAGKLFVLVGAGGAGRALAFGAKSRGAR
Corav_4151 GYNTDCEASITAIEDALKE--------------------QGCTNGEASFSSPLAEKLFVLAGAGGAGRALAFGAKSRGAR
Alngl20613S19962 GYNTDCEASITAIEDALKE--------------------QGCTNGEAPFSSPLAGKQFVLVGAGGAGRALAFGAKSRGAR
ArDHQD-SDH2 GYNTDCEASITAIEDALKE--------------------QGCTNGEAPFSSPLAGKQFVLVGAGGAGRALAFGAKSRGAR
CasDHQD-SDH2 SYNTDYIGAISAIEDGLRES----------------------NGSSPATSSPLAGKLFVVLGAGGAGKSLAYGAQQKGAR
VvDHQD-SDH3 AFNTDYIGAIEAIEDGLRES----------------------NGSSPAVGSPLAGKLFVVLGAGGAGKSLAYGAKEKGAR
FvDHQD-SDH1 AFNTDYIGSISGIEDELRGM----------------------NGAIPAGKSPLAGKLFVVLGAGGAGKSLVYGAAQKGAR
EgDHQD-SDH3 AFNTDYIGAISAIEDGLRGL----------------------NVVSPGA-SPLAGKLFVVLGAGGAGKSLAYGAAQKGAR
EcDHQD-SDH3 AFNTDYIGAISAIEDGLRGL----------------------NGVSSGA-SPLAGKLFVVLGAGGAGKSLAYGAAQKGAR
Qrob_P0208720 AFNTDYIGAISAIEDGLRGL----------------------NGTIPSAGSPLAGKLFVVLGAGGAGKSLAYGAAQKGAR
FSB015730501 AFNTDYIGAISAIEDGLRGL----------------------NGTNPAAGSPLAGKLFVVMGAGGAGKSLAYGAAQKGAR
Corav_14055 AFNTDYIGAISAIEDGLRGL----------------------NGTHPAVGSPLAGKLFVVLGAGGAGKSLAYGAAQKGAR
Corav_11016 AFNTDYIGAISAIEDGLRGL----------------------NGTHPAVGSPLAGKLFVVLGAGGAGKSLAYGAAQKGAR
Bpev01_c1356-g0003 AFNTDYIGAISAIEDGLRGL----------------------NGKHPAAGSPLAGKLFVVLGAGGAGKSLAYGAAQKGAR
ArDHQD-SDH3 AFNTDYIGAISAIEDGLRGL----------------------NGKHPAAGSPLAGKLFVVLGAGGAGKSLAYGAAQKGAR
CasDHQD-SDH3 GYNVDYLGAIGAIEEALGGS----------------------NGSSSGAVSPLASKLFVVIGAGGAGKALAYGGKEKGAR
DkDHQD-SDH GYNVDYLGAIAAIEEGLGGS----------------------SSASNGSVSPLAGRLFVVIGAGGAAKALAYGGKEKGAR
VvDHQD-SDH4 GYNVDYLGAIAAIEEGLRAS----------------------NGTTSVG-SPLAGKLFVVIGAGGAGKALAYGGKEKGAR
EgDHQD-SDH2 GYNVDYLGAIAAIEEALRAS----------------------NGASSTTTSPLAGKLFVVIGAGGAGKALAYGAMEKGAR
EcDHQD-SDH2 GYNVDYLGAIAAIEEALRAS----------------------NGASPTTTSPLAGKLFVVIGAGGAGKALAYGAMEKGAR
PoptrDHQD-SDH5 GYNVDYLGAIAAIEEALGAS-----------------------NGAPASVSPLAGKLFVVMGAGGAGKALAYGAYEKGAR
FvDHQD-SDH2 GYNVDYLGAIAAIEEGLRGLG--------------------LNGSNNGSGSPLAGRLFVVMGAGGAGKALAYGGKQKGAR
Casgl267S13432 GYNVDYLGAIAAIEEGLRAS----------------------NGASNGSGSPLAGKLFVVMGAGGAGKALAFGGKEKGAR
Qrob_P0208730 GYNVDYLGAIAAIEEGLRAS----------------------NGASNGSGSPLAGKLFVVMGAGGAGKALAFGGKEKGAR
FSB015730601 GYNVDYLGAIAAIEEGLRAS----------------------NGASNGSGSPLAGKLFVVMGAGGAGKALAFGGKEKGAR
Juni_23843 GYNVDYLGAIAAIEEGLRAS----------------------NGASQASGSPLTGKLFVVMGAGGAGKALAFGGKEKGAR
ArDHQD-SDH4 GYNVDYLGAIAAIEEGLRA-----------------------NGANGASGSPLAGKLFVVMGAGGAGKALAFGGKEKGAR
Alngl6638S34349 GYNVDYLGAIAAIEEGLRA-----------------------NGANGASGSPLAGKLFVVMGAGGAGKALAFGGKEKGAR
Bpev01_c1356-g0004 GYNVDYLGAIAAIEEGLRAS----------------------NGASPVSGSPLAGKLFVVMGAGGAGKALAFGGKEKGAR
Corav_941 GYNVDYLGAIAAIEEGLRAS----------------------NGASRVSGSPLAGKLFVVMGAGGAGKALAFGGKEKGAR
Corav_7742 ---------------AVRHR----------------------NGSNEARGQD----------------------------
SlDHQD-SDH3 GYNTDCEACVTAIEDALRE------------------RQKTNGHAS--NVSPIAGKLFVLVGAGGAGRAIAFGAKSRGAR
EgDHQD-SDH1 GYNTDCEASITAIEDALRE------------------RHAANGEARAMDASPIAGKAFVLVGAGGAGRALAFGAKSRGAR
PoptrDHQD-SDH4 GYNTDCDASISAIEDALTE------------------RRITQKGVL--EASPLSGKTFVLIGAGGAGRALAFGAKSRGAR
CsDHQD-SDH2 GYNTDCESAISAIEDALRE------------------RQGINGVAS--HTSPIAGKIFVLVGAGGAGRALAFGAKSRGAR
Qrob_P0339740 GYNTDCEASITAIEDALRE------------------RRVANGEAL--RTSPLAGKTFVLVGAGGAGRALAFGAKSRGAQ
Juni_24457 GYNTDCEASITAIEDALRGGVERAYWLKWVLFSGEKYRRLLKGEAS--DTSPIAGKTFVLVGAGGAGRALAFGAKSRGAR
Casgl106S06827 GYNTDCEASITAIEDALGG------------------RKVDHGEAS--YASPISGRTFVLVGAGGAGRALAFGAKSRGAR
Bpev01_c0171-g0015 GYNTDCEASITAIEDALRD------------------RRVKNGDAS--HASPIAGKTFVLVGAGGAGRALAFGAKNRGAH
ArDHQD-SDH5 GYNTDCEACITAIEDALKD------------------KRVENGDAS--HASPIAGKIFVLLGAGGAGRALAFGAKSRGAR
ArDHQD-SDH6 GYNTDCEACITAIEDALKD------------------KRVENGDAS--HASPIAGKIFVLLGAGGAGRALAFGAKSRGAR
483
AtDHQD-SDH VVIANRTYERALELAEAIGGKALSLTDLDNYHPEDGMVLANTTSMGMQPNVEETPISKDALKHYALVFDAVYTPRITRLL
FvDHQD-SDH3 IIIANRKYDRARKLADEVGGDALPFADLANFHPEDGAILANSTSVGMQPNIDETPIPKHALRSYSLVFDAVYNPRMTRLL
FvDHQD-SDH4 IVIANRTYDRAREIADTIGGEALSISDLDKFHPEDGMILANTTSIGMQPKVDETPISKHALRSYTLVFDAVYTPKITRLL
NtDHQD-SDH1 VVIANRTYERARELADVVGGQALSLDELSNFHPENDMILANTTSIGMQPKVDDTPIFKEALRYYSLVFDAVYTPKITRLL
SlDHQD-SDH1 VVIANRTYERARELAIVVGAEALSLDELSNFHPENDMILANTTSIGMQPKVDDTPISKEALKHYSLVFDAVYTPKITRLL
CasDHQD-SDH4 VVIANRTYERAKEIADTIGGDALSLADLSNFHPEDGMILANTTSIGMQPKVDETPVPKQALKSYALVFDAVYTPKITRLL
VvDHQD-SDH1 VVIANRTYARARELADAVGGDALSLADLNNFHPENGMILANTTSIGMQPKVDETPISKHALKYYSLVFDAIYTPKITRLL
FSB010265701 VVIANRTYERAKEIADTVGGQALSLADLENFHPEDGMILANTTSIGMQPKVDETPIPKHALRSYSVVFDAVYTPKITRLL
FSB012889301 VVIANRTYVS--
Qrob_P0275560 VVIANRTYDRARELANTIGGDAVTLADLENFHPVDGMILANTTSIGMQPKVDETPIPKHALRSYSLVFDAVYTPKITRLL
Juni_01730 VVIANRTYDRAREIADTVGGDALSIADLDKFHPEDGMILANTTSIGMQPKVDETPIPKHALRSYSLVFDAGWRCSSDRHT
JrDHQD-SDH VVIANRTYDRARELADTIGGDALSLADLDNFHPEDGMILANSTSIGMQPKVDETPIPKHALRSYSLVFDAVYTPKMTRLL
Juni_19979 VVIANRTYDRARELADTIGGDALSLADLDNFHPEDGMILANSTSIGMQPKVDETPIPKHALRSYSLVFDAVYTPKMTRLL
ArDHQ-SDH1 VVIANRTYDRARELSDIVGGDALSLADLENFHPEDGMILANTTSIGMQPKVDETPIPKHALRYYSLVFDAVYTPKMTRLL
Alngl88491S37275 VVIANRTYDRARELSDIVGGDALSLADLENFHPEDGMILANTTSIGMQPKVDETPIPKHALRYYSLVFDAVYTPKMTRLL
Bpev01_c0420-g0007 VVIANRTYDRARELADTIGGDALSLVDLDNFHPEDGMILANTTSIGMQPKVDETPIPKHALRSYSLVFDAVYTPKMTRLL
Corav_882 VVIANRTYDRARELADTIGGDALSLADLANFHPEDGMILANTTAIGMQPKVDETPIPKHALRSYSLVFDAVYTPKITKLL
Casgl15S00444 VVIANRTYDRARELADTVGGDALSLADLEKFHPEDGMILANTTSIGMQPKVDETPIPKHALTSYSLVFDAVYTPKITRLL
EgDHQD-SDH5 VVIANRTYDRARELAETIGGDALSLADLENFHPEDGMILANTTSIGMQPKVDETPIPKHALKHYSLVFDAVYTPKITRLL
EcDHQD-SDH1 VVIANRTYDRARELAETIGGDALSLADLENFHPEDGMILANTTSIGMQPKVDETPIPKHALKHYSLVFDAVYTPKITRLL
CsDHQD-SDH3 VVIANRTYDRARELAETVGGHALSLADLENFNPEDGMILANTTSIGMQPKVDETPIPKHALGHYALVFDAVYTPKITRLL
PoptrDHQD-SDH1 VVIANRTYERAKVLADIIGGDAITLADLENFHPEDGMILANTTSIGMQPKVDETPVSKNALRSYSLVFDAVYTPKITRLL
PoptrDHQD-SDH3 LIIFDIDLERAKSLARAVSGEAQHFESLAHFQPENGAILANATPIGMHPSTDRIPAAEETLGNYQLVFDAVYTPRKTRLL
PoptrDHQD-SDH2 VIIFDIDLDRAKSLAQVVSGEAQHFDSLAHFQPEKGAILANATPIGMHPSTDRIPVAEATLGNYQLVFDAVYTPRKTRLL
EgDHQD-SDH4 IIIFDIDFERAKMLAHAVSGEARPFGDLPYFQPEKGSILANATPIGMHPNKDRIPVSEVPYLK-----------------

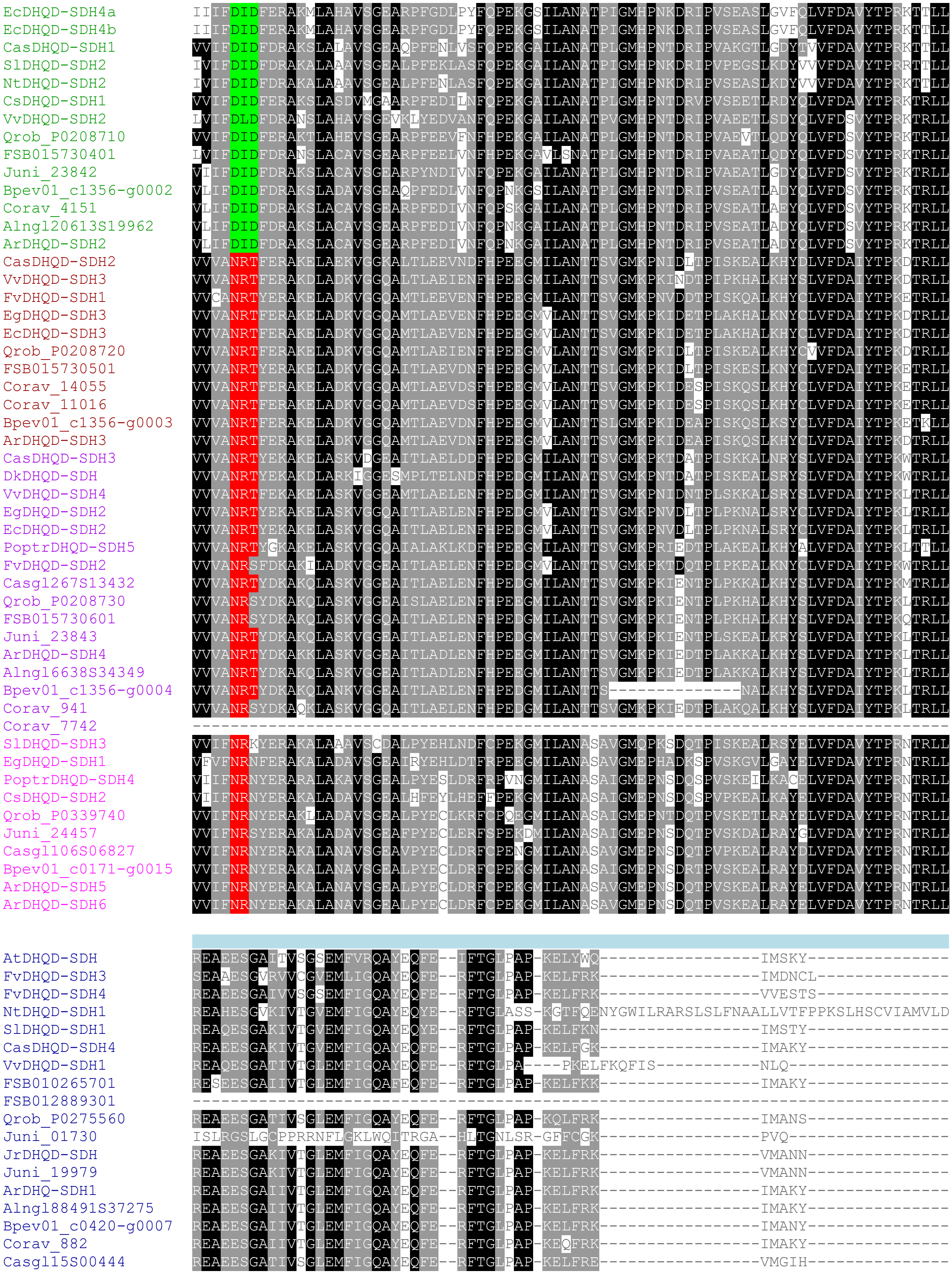
EcDHQD-SDH4a IIIFDIDFERAKMLAHAVSGEARPFGDLPYFQPEKGSILANATPIGMHPNKDRIPVSEASLGVFQLVFDAVYTPRKTTLL
EcDHQD-SDH4b IIIFDIDFERAKMLAHAVSGEARPFGDLPYFQPEKGSILANATPIGMHPNKDRIPVSEASLGVFQLVFDAVYTPRKTTLL
CasDHQD-SDH1 VVIFDIDFDRAKSLALAVSGEAQPFENLVSFQPEKGAILANATPLGMHPNTDRIPVAKGTLGDYTVVFDAVYTPRKTTLL
SlDHQD-SDH2 IVIFDIDFDRAKALAAAVSGEALPFEKLASFQPEKGAILANATPIGMHPNKDRIPVPEGSLKDYVVVFDAVYTPRRTTLL
NtDHQD-SDH2 IVIFDIDFDRAKALAAAVSGEALPFENLASFQPEKGAILANATPIGMHPNKDRIPVSEASLKDYVVVFDAVYTPRKTTLL
CsDHQD-SDH1 VVIFDIDFERAKSLASDVMGAARPFEDILNFQPEKGAILANATPLGMHPNTDRVPVSEETLRDYQLVFDAVYTPRKTRLL
VvDHQD-SDH2 IVIFDLDFDRANSLAHAVSGEVKLYEDVANFQPEKGAILANATPVGMHPNTDRIPVAEETLSDYQLVFDSVYTPRKTRLL
Qrob_P0208710 VVIFDIDFERAKTLAHEVSGEARPFEEVFNFHPEKGAILANATPLGMHPNTDRIPVAEVTLQDYQLVFDSVYTPRKTRLL
FSB015730401 IVIFDIDFDRANSLACAVSGEARPFEELVNFHPEKGAVLSNATPLGMHPNTDRIPVAEATLQDYQLVFDSVYTPRKTRLL
Juni_23842 VIIFDIDFDRAKSLACAVSGEARPYNDIVNFQPEKGAILANATPLGMHPNTDRIPVAEATLGDYQLVFDSVYTPRKTRLL
Bpev01_c1356-g0002 VLIFDIDFDRAKSLADAVSGEAQPFEDLVNFQPNKGSILANATPLGMHPNTDRIPVSEATLADYQLVFDSVYTPRKTRLL
Corav_4151 VLIFDIDFDRAKSLACAVSGEARPFEDIVNFQPSKGAILANATPLGMHPNTDRIPVSEATLAEYQLVFDSVYTPRKTRLL
Alngl20613S19962 VLIFDIDFDRAKSLACAVSGEARPFEDIVNFQPNKGAILANATPLGMHPNTDRIPVSEATLADYQLVFDSVYTPRKTRLL
ArDHQD-SDH2 VLIFDIDFDRAKSLACAVSGEARPFEDIVNFQPNKGAILANATPLGMHPNTDRIPVSEATLADYQLVFDSVYTPRKTRLL
CasDHQD-SDH2 VVVANRTFERAKELAEKVGGKALTLEEVNDFHPEEGMILANTTSVGMKPNIDLTPISKEALKHYDLVFDAIYTPKDTRLL
VvDHQD-SDH3 VVVANRTFERAKDLADKVGGQALTLAEIENFHPEEGMILANTTSVGMKPKINDTPIPKHALKHYSLVFDAIYTPKDTRLL
FvDHQD-SDH1 VVCANRTYERAKELADKVGGQAMTLEEVENFHPEEGMILANTTSVGMKPNVDDTPISKQALKHYCLVFDAIYTPKETRLL
EgDHQD-SDH3 VVVANRTFERAKELADKVGGQAMTLAEVENFHPEEGMVLANTTSVGMKPKIDETPLAKHALKNYCLVFDAIYTPKDTRLL
EcDHQD-SDH3 VVVANRTFERAKELADKVGGQAMTLAEVENFHPEEGMVLANTTSVGMKPKIDETPLAKHALKHYCLVFDAIYTPKDTRLL
Qrob_P0208720 VVVANRTFERAKELADKVGGQAMTLAEIENFHPEEGMVLANTTSVGMKPKIDLTPISKEALKHYCVVFDAIYTPKDTRLL
FSB015730501 VVVANRTYERAKELADKVGGQAITLAEVDNFHPEEGMVLANTTSLGMKPKIDLTPISKESLKNYCLVFDAIYTPKETRLL
Corav_14055 VVVANRTFERAKELADKVGGQAMTLAEVDSFHPEEGMVLANTTSVGMKPKIDESPISKQSLKHYCLVFDAIYTPKETRLL
Corav_11016 VVVANRTFERAKELADKVGGQAMTLAEVDSFHPEEGMVLANTTSVGMKPKIDESPISKQSLKHYCLVFDAIYTPKETRLL
Bpev01_c1356-g0003 VVVANRTFERAKELADKVGGQAMTLAEVDNFHPEEGMVLANTTSLGMKPKIDEAPISKQSLKSYCLVFDAIYTPKETKLL
ArDHQD-SDH3 VVVANRTFERAKELADKVGGQAMTLAEVDNFHPEEGMVLANTTSVGMKPKIDEAPISKQSLKHYCLVFDAIYTPKDTRLL
CasDHQD-SDH3 VVVANRTYEKAKELASKVDGEAITLAELDDFHPEDGMILANTTSVGMKPKTDATPISKKALNRYSLVFDAIYTPKWTRLL
DkDHQD-SDH VVVANRTYEKAKDLARKIGGESMPLTELNDFHPEDGMILANATSVGMKPNTDATPISKEALSRYSLVFDAIYTPKWTRLL
VvDHQD-SDH4 VVVANRTFEKAKELASKVGGEAMTLAELENFHPEDGMILANTTSVGMKPNIDNTPLSKKALSRYSLVFDAIYTPKLTRLL
EgDHQD-SDH2 VVVANRTYEKAKELASKVGGQAITLAELENFHPEDGMVLANTTSVGMKPNVDLTPLPKNALSRYCLVFDAIYTPKLTRLL
EcDHQD-SDH2 VVVANRTYEKAKELASKVGGQAITLAELENFHPEDGMVLANTTSVGMKPNVDLTPLPKNALSRYCLVFDAIYTPKLTRLL
PoptrDHQD-SDH5 VVVANRTYGKAKELASKVGGQAIALAKLKDFHPEEGMILANTTSVGMKPRIEDTPLAKEALKHYALVFDAIYTPKLTTLL
FvDHQD-SDH2 VVVANRSFDKAKILADKVGGEAITLAELENFHPEDGMVLANTTSVGMKPKTDQTPIPKEALKNYCLVFDAIYTPKWTRLL
Casgl267S13432 VVVANRTYDKAKQLASKVGGEAITLAELENFHPEDGMILANTTSVGMKPKIENTPLPKKALKHYSLVFDAIYTPKMTRLL
Qrob_P0208730 VVVANRSYDKAKQLASKVGGEAISLAELENFHPEEGMILANTTSVGMKPKIENTPLPKHALKHYSLVFDAIYTPKLTRLL
FSB015730601 VVVANRSYDKAKQLASKVGGQAITLAELENFHPEDGMILANTTSVGMKPKIENTPLPKHALKHYSLVFDAIYTPKQTRLL
Juni_23843 VVVANRTYDKAKQLASKVGGEAITLAELENFHPEDGMILANTTSVGMKPKIENTPLSKEALKRYSLVFDAIYTPKLTRLL
ArDHQD-SDH4 VVVANRTYDKAKKLASKVGGEAITLADLENFHPEEGMILANTTSVGMKPKIEDTPLAKKALKHYSLVFDAIYTPKLTRLL
Alngl6638S34349 VVVANRTYDKAKQLASKVGGEAITLADLENFHPEEGMILANTTSVGMKPKIEDTPLAKKALKHYSLVFDAIYTPKLTRLL
Bpev01_c1356-g0004 VVVANRTYDKAKQLANKVGGEAITLAELENFHPEDGMILANTTS-------------NALKHYSLVFDAIYTPKLTRLL
Corav_941 VVVANRSYDKAQKLASKVGGQAITLAELENFHPEDGMILANTTSVGMKPKIEDTPLAKQALKHYSLVFDAIYTPKLTRLL
Corav_7742 --
SlDHQD-SDH3 VVIFNRKYERAKALAAAVSCDALPYEHLNDFCPEKGMILANASAVGMQPKSDQTPISKEALRSYELVFDAVYTPRNTRLL
EgDHQD-SDH1 VFVFNRNFERAKALADAVSGEAIRYEHLDTFRPEEGMILANASAVGMEPHADKSPVSKGVLGAYELVFDAVYTPRNTRLL
PoptrDHQD-SDH4 VIIFNRNYERARALAKAVSGEALPYESLDRFRPVNGMILANASAIGMEPNSDQSPVSKEILKACELVFDAVYTPRNTRLL
CsDHQD-SDH2 VIIFNRNYERAKALADAVSGEALHFEYLHEFFPEKGMILANASAIGMEPNSDQSPVPKEALKAYELVFDAVYTPRNTRLL
Qrob_P0339740 VVIFNRNYERAKLLADAVSGEALPYECLKRFCPQEGMILANASAIGMEPNTDQTPVSKETLRAYELVFDAVYTPRNTRLL
Juni_24457 VVIFNRSYERAKALADAVSGEAFPYECLERFSPEKDMILANASAIGMEPNSDQTPVSKDALRAYGLVFDAVYTPRNTRLL
Casgl106S06827 VVIFNRNYERAKALANAVSGEAVPYECLDRFCPENGMILANASAVGMEPNSDQTPVPKEALRAYDLVFDAVYTPRNTRLL
Bpev01_c0171-g0015 VVIFNRNYERAKALANAVSGEALPYECLDRFCPEKGMILANASAIGMEPNSDRTPVSKEALRAYDLVFDAVYTPRNTRLL
ArDHQD-SDH5 VVIFNRNYERAKALANAVSGEALPYECLDRFCPEKGMILANASAVGMEPNSDQTPVSKEALRAYELVFDAVYTPRNTRLL
ArDHQD-SDH6 VVIFNRNYERAKALANAVSGEALPYECLDRFCPEKGMILANASAVGMEPNSDQTPVSKEALRAYELVFDAVYTPRNTRLL
AtDHQD-SDH REAEESGAITVSGSEMFVRQAYEQFE--IFTGLPAP-KELYWQ-----------------IMSKY---------------
FvDHQD-SDH3 SEAAESGVRVVCGVEMLIGQAYEQFE--RFTGLPAP-KELFRK-----------------IMDNCL--------------
FvDHQD-SDH4 REAEESGAIVVSGSEMFIGQAYEQFE--RFTGLPAP-KELFRK-----------------VVESTS--------------
NtDHQD-SDH1 REAHESGVKIVTGVEMFIGQAYEQYE--RFTGLASS-KGTFQENYGWILRARSLSLFNAALLVTFPPKSLHSCVIAMVLD
SlDHQD-SDH1 REAQESGAKIVTGVEMFIGQAYEQYE--RFTGLPAP-KELFKN-----------------IMSTY---------------
CasDHQD-SDH4 REAEESGAKIVTGVEMFIGQAYEQFE--RFTGLPAP-KELFGK-----------------IMAKY---------------
VvDHQD-SDH1 REAQESGATIVTGLEMFIGQAYEQFE--RFTGLPA----PKELFKQFIS-----------NLQ-----------------
FSB010265701 RESEESGAIIVTGLEMFIGQAFEQFE--RFTGLPAP-KELFKK-----------------IMAKY---------------
FSB012889301 --
Qrob_P0275560 REAEESGATIVSGLEMFIGQAYEQFE--RFTGLPAP-KQLFRK-----------------IMANS---------------
Juni_01730 ISLRGSLGCPPRRNFLGKLWQITRGA--HLTGNLSR-GFFCGK-----------------PVQ-----------------
JrDHQD-SDH REAEESGAKIVTGLEMFIGQAYEQFE--RFTGLPAP-KELFRK-----------------VMANN---------------
Juni_19979 REAEESGAKIVTGLEMFIGQAYEQFE--RFTGLPAP-KELFRK-----------------VMANN---------------
ArDHQ-SDH1 REAEESGAIIVTGLEMFIGQAYEQFE--RFTGLPAP-KELFRK-----------------IMAKY---------------
Alngl88491S37275 REAEESGAIIVTGLEMFIGQAYEQFE--RFTGLPAP-KELFRK-----------------IMAKY---------------
Bpev01_c0420-g0007 REAEESGAIIVTGLEMFIGQAYEQFE--RFTGLPAP-KELFRK-----------------IMANY---------------
Corav_882 REAEESGAIIVTGLEMFIGQAYEQFE--RFTGLPAP-KEQFRK-----------------IMAKY---------------
Casgl15S00444 REAEESGATIVSGLEMFIGQAYEQFE--RFTGLPAP-KELFRE-----------------VMGIH---------------

```
EgDHQD-SDH5          KEAEECGATIVSGLEMFIGQAYGQYESTKRTVQENY-VEVLKR-----------------LVDLDSIHIYIFSHL-----
EcDHQD-SDH1          KEAEECGATIVSGLEMFIGQAYGQYE--RYTGLPAP-KELFRK-----------------IMSKY---------------
CsDHQD-SDH3          REAEESGATIVSGLEMFIGQAYEQYE--RFTGLPGK-MNAPHLYKFFVL-----------LLYSFNKFHIFTYFLFSFGN
PoptrDHQD-SDH1       REAEESGAKIVTGLEMFIGQAYEQFE--RFTELPAP-KELFQK-----------------IMS-----------------
PoptrDHQD-SDH3       KDADAAGAITVSGVEMFLRQAIGQFN--LFTGREAP-KDFMREIVLAKF-------------------------------
PoptrDHQD-SDH2       EDADAAGAITVSGVEMFLKQAIGQFS--LFTGREAP-KDFMREIVLAKF-------------------------------
EgDHQD-SDH4          --------------------------------------------------------------------------------
EcDHQD-SDH4a         KEAEAAGAITVSGVEMFLRQAIGQFN--LFTGGEAP-KEFMREIVLAKF-------------------------------
EcDHQD-SDH4b         KEAEAAGAITVSGVEMFLGQAIGQFN--LFTGGEAP-KEFMREIVLAKF-------------------------------
CasDHQD-SDH1         KEAEAAGAIIVSGVEMFLRQAIEQFN--LFTGGKAP-QEFMRRTIFANF-------------------------------
SlDHQD-SDH2          EDAEAAGALIVSGVEMFLRQAIGQFN--LFTGSKAP-EEFMRDIVMSKF-------------------------------
NtDHQD-SDH2          KDAEAAGAITVSGVEMFLRQAIGQFH--LFTRTKAP-EEFMRDIVMAKF-------------------------------
CsDHQD-SDH1          KDAEAAGAIIVSGVEMFLRQAIGQFN--LFTGKEAP-KEFMREIVLAKF-------------------------------
VvDHQD-SDH2          KEAEAAGAIIVSGVEMFLRQAIGQFN--LFTGGEAP-EEFMREIILSKF-------------------------------
Qrob_P0208710        KDAEAAGAIIVSGVEMFLRQAIGQFK--LFTGKEAP-EEFMRDIILAKF-------------------------------
FSB015730401         KEAEAAGAIIVSGVEMFLRQAIGQFN--LFTGREAP-EEFMREIILAKF-------------------------------
Juni_23842           KEAEAVGAIIVSGVEMFLRQAIGQFN--LFTGREAP-EDFMREIILAKF-------------------------------
Bpev01_c1356-g0002   KEAEAAGAIIVSGVEMFLRQAIGQFN--LFTGREAP-EEFMREIIYAKF-------------------------------
Corav_4151           KDAEAAGAIIVGGVEMFLRQAIGQFN--LFTGGEAP-EELMRKIIYAKF-------------------------------
Alngl20613S19962     KESEAAGAIIVSGVEMFLRQAIGQFN--LFTGREGT-FNFLDLLLKNVNIHA----------------------------
ArDHQD-SDH2          KESEAAGAIIVSGVEMFLRQAIGQFN--LFTSREAP-EEFMQEIVYAKF-------------------------------
CasDHQD-SDH2         REARECGKIIVYGTEMLIRQGFEQYK--NFTGLQAP-EELFRELMSRHA-------------------------------
VvDHQD-SDH3          REAKESGKIIVYGTEMLIRQGFEQYK--NFTGLPAP-EELFRELMSKHA-------------------------------
FvDHQD-SDH1          REAKETGAAVVYGTEMLIRQGFEQYK--NFTGLPAP-EALFRELMEKHA-------------------------------
EgDHQD-SDH3          REARETGAVIVYGTEMLIRQGFEQYK--NFTGLPAP-EELFRTLMEKHA-------------------------------
EcDHQD-SDH3          REARETGAVIVYGTEMLIRQGFEQYK--NFTGLPAP-EELFRTLMEKHA-------------------------------
Qrob_P0208720        REARETGAVVVYGTEMLIRQGFEQYK--NFTGLPAP-EDLFRQLMEKHA-------------------------------
FSB015730501         REARETGAIIVYGTEMLIRQGFEQYK--NFTGLPAP-EELFRQLMEKHA-------------------------------
Corav_14055          REARDTGAVIVYGTEMLIRQGFEQYK--NFTGLPAP-EDLFRQLMEKHA-------------------------------
Corav_11016          REARDTGAVIVYGTEMLIRQGFEQYK--NFTGLPAP-EDLFRQLMEKHA-------------------------------
Bpev01_c1356-g0003   REARETGAVIVYGTEMLIRQGFEQYK--NFTGLPAP-EDLFRQLMEKHA-------------------------------
ArDHQD-SDH3          REARETGAVIVYGTEMLIRQGFEQYK--NFTGLPAP-EDLFRQLMEKHA-------------------------------
CasDHQD-SDH3         QEAQDSGAKVVLGTEMFINQAFVQFE--RFTGMPAP-KELIRETLAKNT-------------------------------
DkDHQD-SDH           REAKETGAKVVFGTEMFLNQAFVQFE--KFTGLPAP-KDLIRETLARNT-------------------------------
VvDHQD-SDH4          REAQESGAIIVYGTEMFINQAFVQFE--RFTGLPAP-KELIREVLVRNT-------------------------------
EgDHQD-SDH2          REAQEVGAIPVYGTEMFINQAFVQFE--RFTGYPEQLKSLIRSSLVKSS-------------------------------
EcDHQD-SDH2          REAQEVGAIPVYGTEMFINQAFVQFE--RFTGYPAP-KQLIRDTLVKNS-------------------------------
PoptrDHQD-SDH5       REAQEAGSTIVYGTEMFINQAFVQFE--RFTGLPAP-KQLIRDVLARNT-------------------------------
FvDHQD-SDH2          TEAQESGAAVVFGTEMFLNQAFVQVE--KFSGIPAN-KQLIRDTLARNT-------------------------------
Casgl267S13432       TEAEESGATIVYGTEMFINQAFVQFE--KFTGLPGK-KEQGNLNPGR---------------------------------
Qrob_P0208730        REAEESGATIVYGTEMFINQAFVQFE--KFTGLPAP-KQLIRDTLARNT-------------------------------
FSB015730601         TEAEESGATVVYGTEMFINQAFVQFE--KFTGLPAP-KQLIRDTLARNT-------------------------------
Juni_23843           REAQESGATIVYGTEMFINQAFVQFE--KFTGLPAP-KQLIRDTLARNT-------------------------------
ArDHQD-SDH4          REAQESGAITVYGTEMFINQAFVQFE--KFSGLPAP-KQLIRDTLARNT-------------------------------
Alngl6638S34349      REAQESGAITVYGTEMFINQAFVQFE--KFSGLPAP-KQLIRDTLARNT-------------------------------
Bpev01_c1356-g0004   REAQESGATIVYGTEMFINQAFVQFE--KFSGLPAP-KQLIRDTLARNT-------------------------------
Corav_941            REAQESGATIVYGTEMFINQAFVQFE--KFSGLPAP-KQLIRDTLARNT-------------------------------
Corav_7742           --------------------------------------------------------------------------------
SlDHQD-SDH3          QEATEVGATVVSGVEMFVRQALGQFK--LFTNGLAP-VDFMRRIVYEQF-------------------------------
EgDHQD-SDH1          QEAAQAGAIAVSGMEMFIRQALHQFK--LFTSGLAP-EEFMRKLVLEQF-------------------------------
PoptrDHQD-SDH4       REAKEVGAVVVSGVEMFIRQALGQFR--LFTGGLAP-EAFMRKLVLEQF-------------------------------
CsDHQD-SDH2          REAAEVGATVVSGVEMFIRQALGQFR--LFTGGLAP-EDFMRKLVLEQF-------------------------------
Qrob_P0339740        QEAAEVGAIVVSGVEMFIRQALGQFR--LFTGGLAP-EDFMRKLVLAQF-------------------------------
Juni_24457           QEAAEIGATVVSGVEMFIRQALGQFR--LFTGGLAP-EDFMRKLVLEQF-------------------------------
Casgl106S06827       QEAKEVGIAVVSGVEMFIRQALGQFR--LFTGGLAP-EDFMRKLVLEQF-------------------------------
Bpev01_c0171-g0015   QEATEVGATVVSGVEMFIRQALGQFR--LFTDGLAP-EDFMRKLVLEQF-------------------------------
ArDHQD-SDH5          QEATEVGATVVSGVEMFIRQALGQFR--LFTGGLAP-EDFMRKLVLEQF-------------------------------
ArDHQD-SDH6          QEATEVGATVVSGVEMFIRQALGQFR--LFTGGLAP-EDFMRKLVLEQF-------------------------------
```

Supplementary Figure S4. Protein sequence alignments of *bona fide* bifunctional 3-dehydroquinate dehydratases/shikimate dehydrogenases (DHQD-SDH) from *Arabidopsis thaliana* (At) (6), *Camellia sinensis* (Cas) (46), *Citrus sinensis* (Cs), *Diospyros kaki* (Dk) (47), *Eucalyptus camaldulensis* (Ec) (48), *E. grandis* (Eg), *Fragaria vesca* subsp. *vesca* (Fv), *Juglans regia* (Jr) (49), *Nicotiana tabacum* (Nt) (50), *Populus trichocarpa* (Poptr) (51)), *Solanum lycopersicum* (Sl) (52), *Vitis vinifera* (Vv) (53) and that of putative homologs (ArDHQD-SDH1 – ArDHQD-SDH6) in red alder. The *N*-terminal signal peptide of AtDHQD-SDH was not included. The DHQD domain is shown as a *red-range* bar whereas the SDH

domain is in *light blue*. The consensus motif SX[TG] is highlighted in *blue, green, purple* and *Fuchsia*. The NRT sequence motif typically associated with $NADP^+$ preference is in *red*, whereas the DID motif associated with NAD^+ preference is *green* (54). Names of Group I DHQD-SDHs are depicted in *blue*, Group II in *green*, Group III in *dark red*, Group IV in *purple* and Group V in *Fuchsia*.

AtDHQD-SDH (AAF08579), CasDHQD-SDHa (AYP64306), CasDHQD-SDHb (AYP64307), CasDHQD-SDHc (AYP64308), CasDHQD-SDHd (AYP643079), CsDHQD-SDH1 (orange1.1g010050m), CsDHQD-SDH2 (orange1.1g010101m), CsDHQD-SDH3 (orange1.1g007151m), DkDHQD-SDH (BAI40147), EcDHQD-SDH2 (BBL52471), EcDHQD-SDH3 (BBL52472), EcDHQD-SDH4a (BBL52473), EcDHQD-SDH4b (BBL52474), EgDHQD-SDH1 (Eucgr.H01214.1), EgDHQD-SDH2 (Eucgr.H04428.1), EgDHQD-SDH3 (Eucgr.H04427.1), EgDHQD-SDH4 (Eucgr.B01770.1), EgDHQD-SDH5 (Eucgr.J00263.1), FvDHQD-SDH1 (XP004302480), FvDHQD-SDH2 (XP004302479), FvDHQD-SDH3 (XP004289250), FvDHQD-SDH4 (XP004288087), JrDHQD-SDH (AAW65140), NtDHQD-SDH1 (AS90325), NtDHQD-SDH2 (AAS90324), PoptrDHQD-SDH1 (Potri.010G019000), PoptrDHQD-SDH2 (Potri.013G029900), PoptrDHQD-SDH3 (Potri.005G043400), PoptrDHQD-SDH4 (Potri.014G135500v, PoptrDHQD-SDH5 (Potri.013G029800), SlDHQD-SDH1 (AAC17991), SlDHQD-SDH2 (XP010327280), SlDHQD-SDH3 (XP004242317), VvDHQD-SDH1 (ANC67814), VvDHQD-SDH2 (ANC67815), VvDHQD-SDH3 (ANC67816), VvDHQD-SDH4 (ANC67817). Accession numbers correspond to the NCBI or the Phytozome (v13) databases. Finally, sequences from other Fagales species were retrieved (using BLASTP) from the corresponding databases as indicated in Supplementary Table S1.

```
SlSK       1 MEARVSQSLQLSSWINSDKVVRKPSGLLRFSEKWNEKPRHRVVVSCHLQPRKAAHSDRRVQL---KVSCSP-----QNVQ
ArSK       1 MEATCGAALQLSTLITGSKRNV---ALVPLNQR---VRKECIMKICDFRRKSVCTRQRRFDYGLLSHSCNHSHGLVFEVE

SlSK      73 ASVLESGCFSASIDEIETLKNKAEEVEEYLDGRCVYLVGMMGCGKTTVGRILAETLGYSFFDCDRLIEQAVGGITVAEIF
ArSK      75 IRILESGNSYASFDEDWLLKNKAQEVTSYLNGRSIFLVGMMGSGKTTVGRILSEALGYSFTDSDRYVELAMGGTSVAQIF

SlSK     153 ELRGESFFRDNETEVLHKLSLMHRLVVSTGGGAVVRPINWRHMHKGISVWLDVPLEALAKRITTEGTKSRPLLHEESGDV
ArSK     155 EQRGEGFFRDHESEALRKLSLVPRQIVSTGGGAVVRPVNWKYMRQGITVFLDVPLDALARRIAAVGTDSRPLLNFESGDA

SlSK     233 YDTTLKRLTTLMETRGENYANASARVSLENIAL-KREKDVCHITPAEITLEVLIQIENFLKTQKSVVVL---
ArSK     235 YTKAFVGLFTLTKKRSEAYADADVTISLLDLAANLRLEDVSDITPTVIAIEVLTQIEKFLGGKNGRSLRLYP
```

Supplementary Figure S5. Protein sequence alignment of *bona fide* shikimate kinase from *Solanum lycopersicum* (SlSK (7)) and that of a putative homolog (ArSK) in red alder.

```
AtEPSPS    1 MAQVSRICNGVQNPSLIS-NLSKSSQRKSPLSVSLKTQQHPRAYPISSSWGLKKSGMTLIGSELRPLKVMSSVSTAE---
ArEPSPS    1 MAQVSKICSGGAQSTQIFHNNPKPHKPKSLNSVNFR----SQFLVSSNSWTLKHKNVCVSSRRAGVVRVSASVATPEKPS

AtEPSPS   77 KASEIVLQPIREISGLIKLPGSKSLSNRILLLAALSEGTTVVDNLLNSDDINYMLDALKRLGLNVETDSENNRAVVEGCG
ArEPSPS   77 TVPEIVLQPIKEISGAITLPGSKSLSNRILLLAALSEGTTVVDNLLNSDDVHYMLGALRTLGLRVEEDKAIKRAIVEGCG

AtEPSPS  157 GIFPASIDSKSDIELYLGNAGTAMRPLTAAVTAAGGNASYVLDGVPRMRERPIGDLVVGLKQLGADVECTLGTNCPPVRV
ArEPSPS  157 GLFPVGKESRDEIQLFLGNAGTAMRPLTAAVTAAGGNSSYILDGVPRMRERPIEDLVTGLKQLGADVDCFLGTKCPPVRV

AtEPSPS  237 NANGGLPGGKVKLSGSISSQYLTALLMSAPLALGDVEIEIVDKLISVPYVEMTLKLMERFGVSVEHSDSWDRFFVKGGQK
ArEPSPS  237 IGKGGLPGGKVKLSGSISSQYLTALLLASPLALGDVEIQIIDKLISIPYVEMTLKLMERFGVTVEHTDSWDRFLVRGGQK

AtEPSPS  317 YKSPGNAYVEGDASSASYFLAGAAITGETVTVEGCGTTSLQGDVKFAEVLEKMGCKVSWTENSVTVTGPPRDAFGMRHLR
ArEPSPS  317 YKSPGNAFVEGDASSASYFLAGAAVTGGTVTVEGCGTSSLQGDVKFAEVLEKMGAKVTWTENSVTVTGPPRDSSKRKHLR

AtEPSPS  397 AIDVNMNKMPDVAMTLAVVALFADGPTTIRDVASWRVK--------------ETERMIAICTE---LRKLGATVEEGSDY
ArEPSPS  397 AIDVNMNKMPDVAMTLAVVALFADGPTSIRDGSGKLESERDRKDDCHLHRTQEGKFHVAFCTSEIPSPFLGATVEEGPDY

AtEPSPS  460 CVITPPKKVKTAEIDTYDDHRMAMAFSLAACADVPITINDPGCTRKTFPDYFQVLERITKH
ArEPSPS  477 CVITPPENLNVTAIDTYDDHRMAMAFSLAACGDVPVTINDPSCTRKTFPDYFEVLQRFTKH
```

Supplementary Figure S6. Protein sequence alignment of *bona fide* 5-enolpyruvylshikimate-3-phosphate synthase from *Arabidopsis thaliana* (AtEPSPS (8)) and that of a putative homolog (ArEPSPS) in red alder.

```
CsCS      1 -MASSLSTKPFLSGSRRRSTTDGSGWSYFQTSDLRQLSNQSVQISVRRQTAPLKLVVQASGSSFGKVFQVTTYGESHGGG
ArCS      1 MASSSLSTKPFLGSSRTDGF------SGLRHLP-SHLIPSSVHISI-RTRSPKKLQIQAAGNTFGNYFRVTTFGESHGGG

CsCS     80 VGCVIDGCPPRFPISEADIQSDLDRRRPGQSRITTPRKETDTCKIYSGVADGFTTGSPIHISVPNTDQRGNDYSEMAKAY
ArCS     73 VGCIIDGCPPRLPLSEADMQFDLDRRRPGQSRITTPRKETDTCRILSGVAEGLTTGTPILVLVPNTDQRGHDYSEMAKAY

CsCS    160 RPSHADATYDFKYGVRSVQGGGRSSARETIGRVAAGALAKKILKAYAGTEVLAYVSQAHKVVLPEGLVDHETLSLEQIES
ArCS    153 RPSHADATYDMKYGTRAIQGGGRSSARETIGRVAAGAVAKKILKSLAGIEILAYVSQVHKVVLPEGLVDHYSLALDQIES

CsCS    240 NIVRCPDSEYAEKMIAAIDAVRVKGDSVGGVVTCIMRNVPRGLGSPVFDKLEAELAKACMSLPATKGFEFGSGFSGTFLT
ArCS    233 NIVRCPNPEYADKMIEAIDAVRVKGNSIGGVVTCIARDCPRGLGSPVFDKLEAELAKAVMSLPATKGFEFGSGFAGTLLT

CsCS    320 GSEHNDEFYTDENGRIRTRTNRSGGIQGGISNGEIINMRIAFKPTSTIGKKQNTVTREREEIELIARGRHDPCVVPRAVP
ArCS    313 GIEHNDEFYTDEHGKIRTRTNRSGGIQGGISNGEIINMRVAFKPTATIAKKQLTVARDKKEMELIARGRHDPCVVPRAVP

CsCS    400 MVEAMVALVLLDQLMLQHAQGNLFSINPALQEPLSETVSSAAASLQGV--------
ArCS    393 MVEAMVALVILDQLMAQHAQCNLFPINPDLQEPLFLPRLDDGVAEHEHEHDDLVVG
```

Supplementary Figure S7. Protein sequence alignment of *bona fide* chorismate synthase from *Capnoides sempervirens* (CsCS (9)) and that of a putative homolog (ArCS) in red alder.

```
AtCM1      1 MRSSCCSSAIGGFFDHRRELSTSTPISTLLPLPSTKSSFSVRCS----LPQPSKPRSGTS-----SVHAVMTLAGSLTGK
AtCM2      1 ------------------------------------------------------------------------MARVFESD
ArCM1      1 MEAKLLGPATPAITAH-HASRFSRPISPLVLQ--SRQSFSFRS------QASSLAKRGIQ-----SVQATATSAGSLTIK
ArCM2      1 --------------------------------------MVVRL--LILFLFS----HGVV---------RNRDNSMAEAL
ArCM3      1 MEAKLLETGKPGTPAL-------------ITFKSSRPTLHFASQTTLKFLQSHSGRNGLRPLLVSSASSTSSSPFRYARK

AtCM1     72 KRVDESESLTLEGIRNSLIRQEDSIIFGLLERAKYCYNADTYDPTAFDMDGFNGSLVEYMVKGTEKLHAKVGRFKSPDEH
AtCM2      9 SGSGCSNVLSLDLIRESLIRQEDTIVFSLIERAKFPLNSPAFEESRCLDSGSFSSLTEFFVRETEIIQAKVGRYEYPEEN
ArCM1     67 KRVDESENLTLEGIRRSLIQQEDSIIFSLLERAQYCYNADTYDPNAFSMDGFHGSLVEYMVKETEKLHAQVGRFKSPDEH
ArCM2     28 SNAGSANALTLESVRQSLIRQEDTIVFGLIERARFPINSPTYNVSYANIPGFSGSLAQFLVRETEALQAKAGRFTNPEEH
ArCM3     68 KRVDESETLTLDCIRHSLIRQEDSIIFSLLERAQYCYNADTYDHDTFSMDGFHGSLVEFMVRETEKLHAQVDRYKSPDEH

AtCM1    152 PFFPDDLPEPMLPPLQYPKVLHFAADSININKKIWNMYFRDLVPRLVKKGDDGNYXSTAVCDAICLQCLSKRIHYGKFVA
AtCM2     89 PFFLENIPHSVFPTHKYPSALHPKALSVNINKQIWDIYFKELLPLFVKPGDDGNYPSTAASDLACLQALSRRIHYGKFVA
ArCM1    147 AFFPDELPEPVLPPLQYPQVLHPFADSININKKVWDMYFRDLVPRLVEEGDDGNCGSTAVCDAMCLQALSKRIHYGKYVA
ArCM2    108 PFFPKDLPPSLVPSQNHTEDLHPSAASININKSIWDVYFDKLLALFAAPGDDGNYEATAASDLVCLQALSRRIHYGKLVA
ArCM3    148 SFFPADLPEPMLPPLQYPQVLHPCADSININNKVWNMYFRDLLPRLVKTGDDGNYGSAAVCDTLCLQALSKRIHYGKFVA

AtCM1    232 EAKFQASPEAYESAIKAQDKDALMDMLTFPTVEDAIKKRVEMKTRTYGQEVKVGMEEKEEEEEEGNESHVYKISPILVGD
AtCM2    169 EVKFRDAPQDYEPAIRAQDREALMKLLTFEKVEEMVKKRVQKKAETFGQEVKFNSGYGD------ESKKKYKVDPLLASR
ArCM1    227 ETKFRASPDAYEAAIKGQDRQRLMDMLTYPKVEEAIKRRVEMKAKTYGQEVTVDLGED-------GAEPVYKIVPSLVAD
ArCM2    188 EVKFKASPSEYEPAIRAKDRDALMKLLTVESVEEEVKKRVAKKAMVFGQEVTLNKTVDD--------TGKYKVDPSVLSR
ArCM3    228 EAKFLSSPISYEAAIRVQDRAGLVELLTYETVEAAVKKRVETKAKIFGQDVRMNQEED-------EAGPAYKIKPRLVAN

AtCM1    312 LYGDWIMPLTKEVQVEYLLRRLD
AtCM2    243 IYGEWLIPLTKLVEVEYLLRRLD
ArCM1    300 LYGDWIMPLTKEVQVEYLLRRLY
ArCM2    260 LYGEWVMPLTKLVEVEYLLRRLD
ArCM3    301 LFGEWIMPLTKQVQVEYLLRRLD
```

Supplementary Figure S8. Protein sequence alignment of *bona fide* chorismate mutases from *Arabidopsis thaliana* (AtCM1 and AtCM2 (10)) and that of putative homologs (ArCM1 – ArCM3) in red alder.

```
AtPPA-AT    1 -------------------------------------------------------------MA-SQSSVAVISSAAARGE
ArPPA-AT    1 MGADGSCDSPHHPTPPTPPKLFSSHQPALARTLKSPCFQNSVAFLLLGVNPSRIQHLPVLFMANSLHSSSAIPRLSLRGQ

AtPPA-AT   19 SFPD---SKKPIGSVRFQQ-PLRLSFSYCK--SGNMSSRICAMAKPNDAETLSSSVDMSLSPRVQSLKPSKTMVITDLAA
ArPPA-AT   81 SLGLHSTSEPSFRSVSFASHPRTLPLRSLEGTRQVKSPRVSAVV-RAESRFDAMEVDLSLSPRVNSVKPSKTVAITDQAT

AtPPA-AT   93 TLVQSGVPVIRLAAGEPDFDTPKVVAEAGINAIREGFTRYTLNAGITELREAICRKLKEENGLSYAPDQILVSNGAKQSL
ArPPA-AT  160 ALVQAGVPVIRLAAGEPDFDTPAPIAEAGINAIREGYTRYTPNAGTVEIRQAICHKLKEENGISYTPDQIVVSNGAKQCI

AtPPA-AT  173 LQAVLAVCSPGDEVIIPAPYWVSYTEQARLADATPVVIPTKISNNFLLDPKDLESKLTEKSRLLILCSPSNPTGSVYPKS
ArPPA-AT  240 LQALLAVCSPGDEVIIPAPFWVSYPEMARLSDATPVILPTSISENFLLDPKVLESKITEKSRVLILCSPSNPTGSVYPKR

AtPPA-AT  253 LLEEIARIIAKHPRLLVLSDEIYEHIIYAPATHTSFASLPDMYERTLTVNGFSKAFAMTGWRLGYLAGPKHIVAACSKLQ
ArPPA-AT  320 LLEEIAQVVAKHPRLLVLSDEIYEHIIYAPATHTSFASLPGMWERTLTVNGFSKAFAMTGWRLGYIAGPKHFIAACGKIQ

AtPPA-AT  333 GQVSSGASSIAQKAGVAALGLGKAGGETVAEMVKAYRERRDFLVKSLGDIKGVKISEPQGAFYLFIDFSAYYGSEAEGFG
ArPPA-AT  400 SQSTSGASSISQKAGVAALGLGYAGGEAVSTMVKAFRERRDFLVKSFGQLEGVRISEPQGAFYLFIDFSSYYGGEAEGFG

AtPPA-AT  413 LINDSSSLALYFLDKFQVAMVPGDAFGDDSCIRISYATSLDVLQAAVEKIRKALEPLRATVSV
ArPPA-AT  480 KIENSESLCRYLLDKGQVALVPGDAFGDDTCIRISYAASLTTLKAAVERIKKALVSLRPAVPV
```

Supplementary Figure S9. Protein sequence alignment of *bona fide* prephenate aminotransferase (AtPPA-AT (11)) from *Arabidopsis thaliana* and that of a putative homolog (ArPPA-AT) in red alder.

```
AtADT5    1 ------MQTISPAFSCDLKSVIQPNLTAKKARYSHVNGKRVSVRCSYRSESFSFPNGVGSSRADWQSSCAILASKVVSAE
ArADT1    1 ------MQAIAPSAPNSLKSLTG----TIRPALSRRGPSRLVVQSVYRSDSVNFPNGVGLSRSDWQSSCAILSSKVVSQE
ArADT2    1 MALKGAAIWVCARTSCSDLGASDLGSRNSGSAVSLRFDLEKFRKWECCSLGALAQRAITPVEDEKPHVPGVESSGAVEST
ArADT3    1 ----MAAIVRSPTNPLSRHVSSKPSPSEHRSKASVTIHVPYPKRRRYFPVLASLQGDHDNNDNNDRNAQALQLQRILDDS

AtADT5   74 NSSS----------------VAVVNGHSNGSVDLSLVPSKSQHNGKPG---LIQPLTITDLSPAPSHGSTLRVAYQGVPG
ArADT1   70 QPTEKPGAAGGGGAAGGADHVAAVNGH-KASIDLDLVPISTDSNNKPPSQLAHRALTITDLSPAPMHGSELRVAYQGVPG
ArADT2   81 QGNES----------------------RGFHKDLNLLP---------------KPLSANDLSSCPSNGSKVRVAYQGLPG
ArADT3   77 QYDV-------------------------VSKDPNMLP---------------RPLSSSQFSDTISHGSRLRVAYQGVGG

AtADT5  135 AYSEAAAGKAYPNSEAIPCDQFDVAFQAVELWIADRAVLPVENSLGGSIHRNYDLLLRHRLHIVGEVQIPVHHCLLALPG
ArADT1  149 AYSEAAAGKAYPNCEAIPCDQFEVAFQAVELWIADRAVLPVENSLGGSIHRNYDLLLRHRLHIVGEVQLPVHHCLLALPG
ArADT2  124 AYSEAAALKAYPKSETVPCDQFEEAFKAVELWLVDKAVLPIENSVGGSIHRNYDLLLRHRLHIVGEVQMQVIHCLLGLPG
ArADT3  117 AYSESAAEKAYPNCEAVPCEQFDTAFEAVERWLVDRAVLPIENSLGGSIHRNYDLLLRHSLHIVGEVKFAVRHCLMANHG

AtADT5  215 VRTDCITRVISHPQALAQTEGSLNKLTPKAAIEAFHDTAAAAEYIAANNLHDTAAVASARAAELYGLQILADGIQDDAGN
ArADT1  229 VRKEYLTRVISHPQALAQCELTLTKLGLNVAREAVDDTAGAAEFVAANNLRDTAAIASARAADLYGMNILADGIQDDSSN
ArADT2  204 VRKEDLKSVLSHPMALDQCDMMLSNLG--VVRINADDTAGAAQMVASTGLRDTAAIASSRAAEIYGLDILAEKIQDDDDN
ArADT3  197 VKLEELKRVLSHPQALAQCEHTLTRLG--LVREAVDDTAGAAKHVAFHKLEDTGAVASSTAAMIYGLNILAQDIQDDCDN

AtADT5  295 VTRFLMLARDPIIPRTDRPFKTSIVFAAQEHKGTSVLFKVLSAFAFRNISLTKIESRPHQNCPVRVVGDENVGTSKHFEY
ArADT1  309 VTRFVMLAREPIIPRTDRPFKTSIVFA--HDKGTSVLFKVLSAFAFRNISLTKIESRPHRNRPIRLVDDANVGTAKHFEY
ArADT2  282 ITRFLILAREPIIPGTDRPYKTSIVFT--LEEGPGILFKALSVFALRGINLSKIESRPQKQRPLRVVDDSNDGSAKYFDY
ArADT3  275 VTRFLILAREPIIPGIDRPFKTSIVFS--LEEGPGVLFKALAVFALRQINLTKIESRPLRKQLLGASDDKNNGVPKYFDY

AtADT5  375 TFYVDFEASMAEARAQNALAEVQEYTSFLRVLGSYPMDMTPWSTLPSEDV
ArADT1  387 MFYVDFEASMAEVRAQNALAEVQEFTSFLRVLGSYPMDMTPWCPSGGD--
ArADT2  362 LFFIDFEASMAEPRAQYALGHLQEFSRFLRVLGSYPIDTVL---------
ArADT3  353 LFYVDFEASMADQNAQNALRHLKEFATFLRVLGSYPVDTSMI--------
```

Supplementary Figure S10. Protein sequence alignment of *bona fide* arogenate dehydratase from *Arabidopsis thaliana* (AtADT5 (12)) and that of putative homologs (ArADT1 – ArADT3) in red alder.

```
UGT84A13     1 MGSEA-LVHVFLVSFPGQGHVNPLLRLGKRLAAKGLLVTFSTPESIGKQMRKASNITD-EPAPVGEGFIRFEFFEDGWDE
UGT84A22     1 MGSES-LVHVFLVSFPGQGHVNPLLRLGKRLASRGLLVTFSAPESIGKDMRKASNCTD-EPTPVGDGFIRFEFFEDGWDE
UGT84A23     1 MGSESSLVHVFLVSFPGQGHVNPLLRLGKRLASKGLLVTFTTPESIGKQMRKASNISD-QPAPVGDGFIRFEFFEDGWDE
UGT84A24     1 MGSES-LVHVFLVSFPGQGHVNPLLRLGKRLASKGLLVTFTTPESIGKQMRKASNIGE-EPSPIGDGFIRFEFFEDGWDE
ArUGT1       1 MGSEA-LVHVLLISFPGQGHVNPLLRLGKRLASKGLLVTFSTPESIGKQMRKASNITD-EPAPVGEGFIRFEFFEDGWDE
ArUGT2       1 MGSEA-LVHVLLISFPGQGHVNPLLRLGKRLASKGLLVTFSTPESIGKQMRKASNITD-EPAPVGEGFIRFEFFEDGWDE
ArUGT3       1 MDSGA-PTHVFLVSFPAQGHINPLLRLGKRLAAKGLLVTFSTTESIGKDMRKANNTTDDQAIPVGEGFIRFEFFKDGWEM
ArUGT4       1 ---MG-SPHVLLVSFPGQGHINPLLRLGKRLAAKGLLVTFSTTESIGKDMRKANNITDDQATPVGQGFIRFEFFQDGWEE
ArUGT5       1 MATEA-PTHVLLVSFPAQGNINPLLRLGKRLAAKGMLVTFSTTETIGKDMRKANDITD-QATPVGDGFIRFDFFDDGWEE

UGT84A13    79 DEPRRQDLDQYLPQLELIGKDIIPKMIRKNAEMGRPVSCLINNPFIPWVSDVAESLGLPSAMLWVQSCACFCAYYHYYHG
UGT84A22    79 NEPRRQDLDQYLPQLELVGKDLLPKMLQKHADQDRPVSCLINNPFIPWVSDLAETLGLPSAMLWVQSCACFSAYYHYYHG
UGT84A23    80 DEPRRQDLDQYLPQLEKVGKVLIPQMIQKNAEQGRPVSCLINNPFIPWVSDVAETLGLPSAMLWVQSCACFLAYYHYYHG
UGT84A24    79 DEPRRQDLDQYLPQLEKVGKEVIPRMIKKNEEQNRPVSCLINNPFIPWVSDVAESLGLPSAMLWVQSCACFAAYYHYYHG
ArUGT1      79 NETRRQDLDQYLPQLELVGKEVFPEMIRRNAEQGRPISCLINNPFIPWVSDVAESLGLPSAMLWVQSCACFSSYYHYCHG
ArUGT2      79 NETRRQDLDQYLPQLELVGKEVFPEMIRRNAEQGRPISCLINNPFIPWVSDVAESLGLPSAMLWVQSCACFSSYYHYCHG
ArUGT3      80 DDPRRKDLEGYLRQLELAGKDALHQIIKKHAHKDHPVSCLINNAFISWVCDVAIELGIPNAVLWVQSCAAFSAYYHYHHN
ArUGT4      77 DDPRRKDLEEFLRHLEPAGKDALHQIIKKHAGKDHPVSCLINNSFIPWVCDVATELGIPNAVLWVQSCAAFSAYYHYHHN
ArUGT5      79 DDPRRKDLDEYLPQLELAGKDALHQIIKKHAHEDRPVSCLINNPFDPWVCDVATELGIPNAILWVQSCAVFSAYYHYYHN

UGT84A13   159 LVPFPSEAEPFIDIQLPCMPLLKYDETPSFLYPTTPYPFLRRAILGQYGNLDKPFCILMDTFQELEHEVIEFMSKICPIK
UGT84A22   159 IVPFPSEDNMEIDVQLPCMPLLKYDEVPSFLYPNTPYPFLRRAILGQYKNLSKPFCILMDTFQELEHEVIEYMSKISPIK
UGT84A23   160 LVPFPSENAMEIDVQLPSMPLLKHDEVPSFLYPTTPYPFLRRAILGQYKNLEKPFCILMDTFQELEHEIIEYTSKICPIK
UGT84A24   159 LVPFPSESAMEIDVQLPCMPLLKHDEVPSFLYPTTPYPFLRRAIMGQYKNLDKPFCVLMDTFQELEHEIIEYMSKICPIK
ArUGT1     159 LVPFPSEAEPFIDVQLPCMPLLKYDEVPSFLYPTTPYPFLRRAILGQYRNLDKPFCILMDTFQELEHDVIEYASKLCPIK
ArUGT2     159 LVPFPSEAEPFIDVQLPCMPLLKYDEVPSFLYPTTPYPFLRRAILGQYRNLDKPFCILMDTFQELEHDVIEYASKLCPIK
ArUGT3     160 LVPFPSESQPMIDVQLPSMPLLRYDEVPDFLHPSSPFWFLGRIVLGQFKSISKPFCILMDSFQELEHEIIDYMAKLCVIK
ArUGT4     157 LVPFPSETEPKLDVQLPSMPLLRYDEVPDFLHPSSPYHFFGRLILGQFKNLSKPFCILMDTFQELEHEIIDYMAKLCMIK
ArUGT5     159 LASFPSETEPKIDVQLPCMPLLKYDEVPDFLHPSSPFQVLGRLILGQFKNLSKPFCILMDTFQQLEPEIINYMSKLCMIK

UGT84A13   239 TVGPLFKNPKAPNS-VRGDFMKADDCLEWLDSKPPQSVVYISFGSVVYLTQKQVDEIAFGLLQSGVSFLWVMKPPHKDAG
UGT84A22   239 PVGPLFKNPKAPNSNVRGDFMKADNCMEWLDSKPPGSVVYISFGSVVYLKQEHVDEIAHGILSSGVSFLWVMKPPHKDAG
UGT84A23   240 TVGPLFKNPKAPNTTVKGDFMKADDCIGWLDSKPASSVVYVSFGSVVYLKQDQWDEIAYGLLNSGVNFLWVMKPPHKDSG
UGT84A24   239 TVGPLFKNPKAPNANVRGDFMKADDCISWLDSKPPASVVYVSFGSVVYLKQDQWDEIAFGLLNSGLNFLWVMKPPHKDSG
ArUGT1     239 TVGPLFKNPKAQNTTVRGDFMKADDCMEWLDSKPPQSVVYISFGSVVYLTQPQVDEIAHGLLNSGVSFLWVMKPPHKDSG
ArUGT2     239 TVGPLFKNPRHRTQPCAATS------------------------------------------------------------
ArUGT3     240 PVGPLFINPKAPNTSVRGDILKADDCIEWLNSKPLASVAYISFGSVAYVEQEQVDEIAYGLLNSGISFLWVIKPPGKDTD
ArUGT4     237 PVGPLFINPKAPNTSIRGDVLKADDCIEWLNSKPPASVAYISFGTVAHVKQEQVDEIAYGLLNSGISFLWVMKPPHKDTD
ArUGT5     239 PVGPLFINPKAPTTSIRGDFLKADDCIEWLNSKPPASVVYISFGSSANIGQAQADELAYGLFNSGTSFLWVMKPPQKDAP

UGT84A13   319 LELLVLPDGFLEKAGDNGRVVQWSPQEQVLAHPSVACFVTHCGWNSTMESLTSGMPVVAFPQWGDQVTDAVYLVDVFKTG
UGT84A22   319 LELLVLPEGFLEKAGDKGRVVQWSPQEQVLVHPAVACFVTHCGWNSTMESLASGMPVVAFPQWGDQVTDAKYLVDVFKVG
UGT84A23   320 YTVLTLPEGFLEKAGDRGKVVQWSPQEQVLAHPATACFVTHCGWNSSMEALTSGMPVVAFPQWGDQVTDAKYLVDEFKVG
UGT84A24   319 YQLLTLPEGFLEKAGDKGKVVQWSPQEQVLAHPSVACFVTHCGWNSSMEALSSGMPVVAFPQWGDQVTDAKYLVDVFKVG
ArUGT1     319 YELLVLPDGFLEKAGDKGRVVHWSPQEQVLAHPSVACFVTHCGWNSTMESLTSGMPVVAFPQWGDQVTDAVYLVDVFKTG
ArUGT2         --------------------------------------------------------------------------------
ArUGT3     320 FKLHVLPDGFLEKVGNRGKLIQWSPQEEVLAHPSVACFVTHCGWNSSMEALTLGVPVVTFPQWGDQVTNAKFLVDVFGVG
ArUGT4     317 FKLHVLPDGILEKVGDRGKVIQWSPQEEVLAHPSVACFVTHCGWNSSVEALTLGVPVLTFPQWGDQVTNSKFLVDVFGVG
ArUGT5     319 FKLHVLPDGFMEKVGNKGKVIQWSPQEQVLAHPSVACFITHCGWNSSMEALTLGVPVVAFPHWGDQVTNAKFLVDVFGVG

UGT84A13   399 VRMCRGEAENRVITRDEVEKCLLEATVGPKAVEMKQNASKWKAAAEAAFSEGGSSDRNIQAFVDEVRARSVAITGKSTAN
UGT84A22   399 IRMCRGEAEDRVIPREEVEMCLREATSGPKAAEMKANALKWKETAEAAVAEGGSSDRNMQAFVDEVRRRSLGITSKSTTP
UGT84A23   400 VRMCRGEAEDKLITRDVVEQCLREATQGPKAAEMKKNALKWKAAAEASFVEGGSSDRNLQAFVDEVKRRSIEITASKPAV
UGT84A24   399 VRMCRGEAENKLIMRDVVEKCLLEATVGPKAAEVKENALKWKAAAEAAVAEGGSSDRNIQAFVDEVKRRSIAIQSNKSEP
ArUGT1     399 LRMCRGEAENRVITRDEIEKCLVEATVGPKAVEMKQNALKWKAAAEDAVAEGGSSDRNIQAFVDEVRKRSIALTSKSAAS
ArUGT2         --------------------------------------------------------------------------------
ArUGT3     400 VRLSRGLAENRLILRDEVEKCLLEATVGPKAVEVKQHALKWKTAAGEAVAEGGSSDQNIQSFVDEIRKRCVAATSA----
ArUGT4     397 VRLCRGQAENRLILRDEVEKCLLEATLSPKAVELKQHALKWKTAAGEAVAEGGSSDQNIQSFVDEIRKRCVAVTSTSITQ
ArUGT5     399 LRLSRGLAENRLILRDEVEKCLLDAMVGPKAVEMKHNALKWKRAAKEVVAEGGSSDQNVKALVDEIRKRCAAVTSKSTTQ
```

Supplementary Figure S11. Protein sequence alignment of *bona fide β*-glucogallin forming UDP glucosyltransferases from oak [*Quercus robur*, UGT84A13 (13)], tea [*Camellia sinensis*, UGT84A22 (55)], pomegranate [*Punica granatum*, UGT84A23 and UGT84A24 (56)] and that of putative homologs (ArUGT1 to ArUGT5) in red alder.

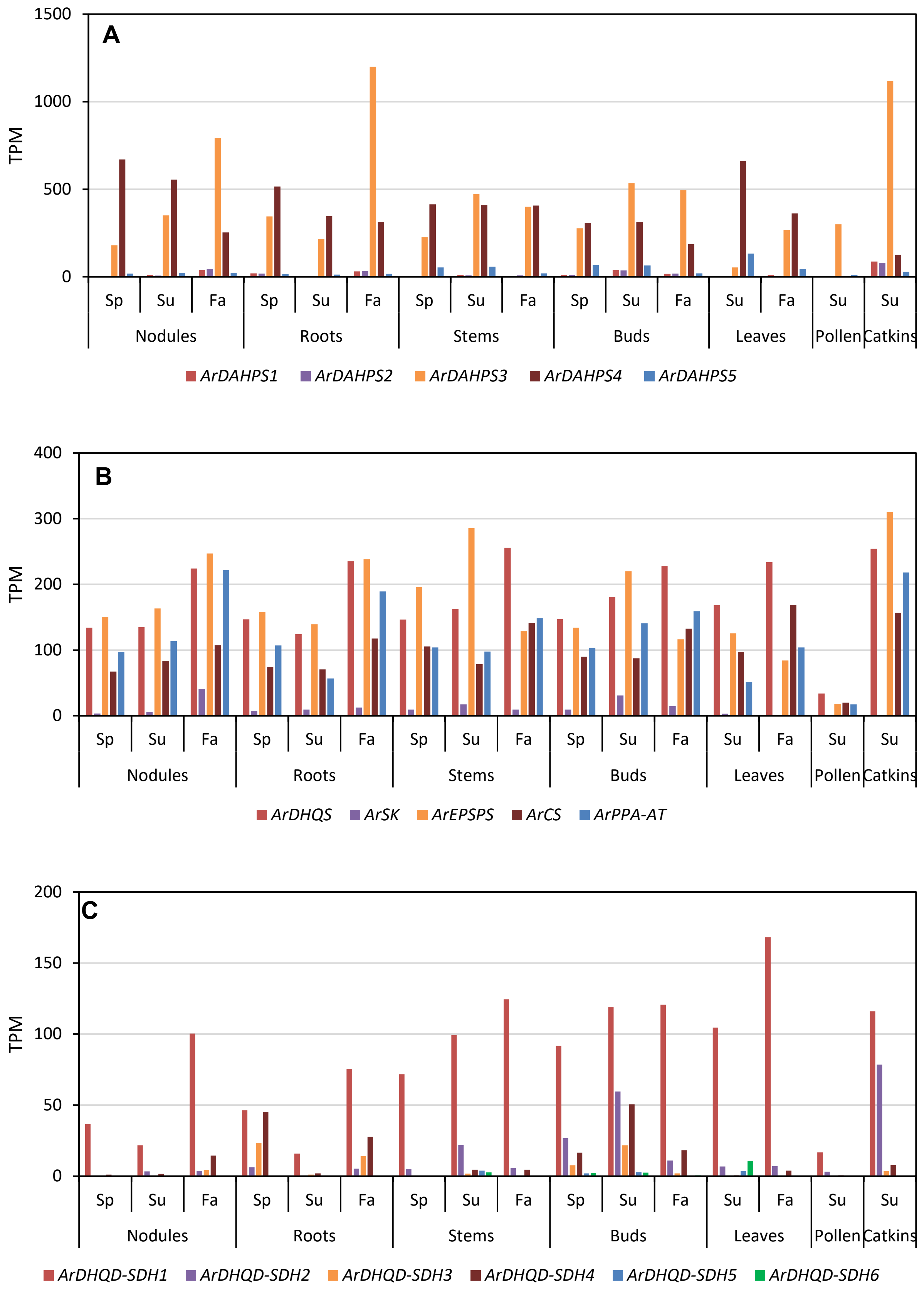
A
B
C
TPM
Sp Su Fa Sp Su Fa Sp Su Fa Sp Su Fa Su Fa Su Su
Nodules Roots Stems Buds Leaves Pollen Catkins
ArDAHPS1 ArDAHPS2 ArDAHPS3 ArDAHPS4 ArDAHPS5
ArDHQS ArSK ArEPSPS ArCS ArPPA-AT
ArDHQD-SDH1 ArDHQD-SDH2 ArDHQD-SDH3 ArDHQD-SDH4 ArDHQD-SDH5 ArDHQD-SDH6

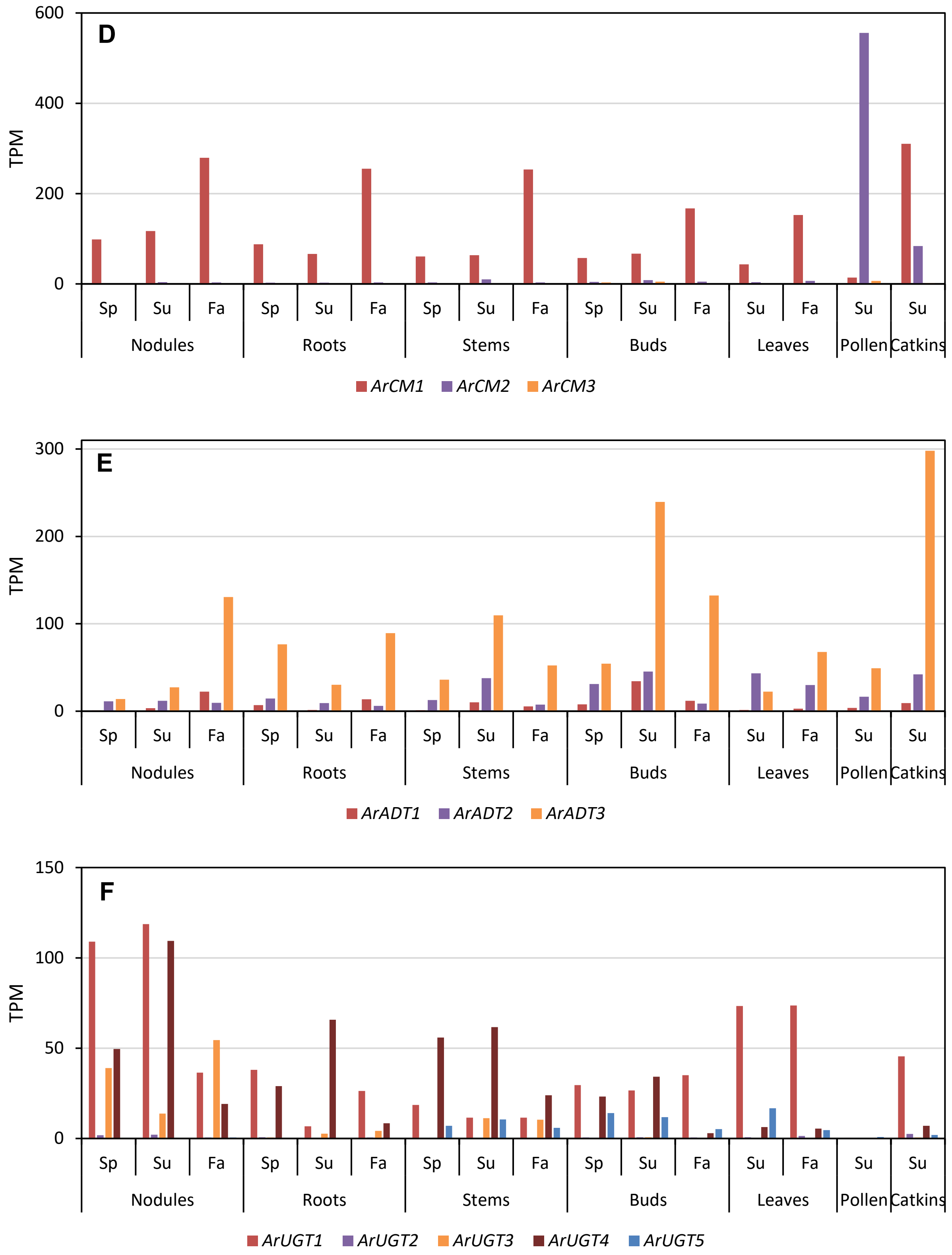

Supplementary Figure S12. Expression levels of red alder homologs of *bona fide* shikimate, chorismate and phenylalanine pathway genes, as well as β-glucogallin forming genes. **(A)** 3-

Deoxy-D-arabinoheptulosonate 7-phosphate synthase (*ArDAHPS*). **(B)** Dehydroquinate synthase (*ArDHQS*), shikimate kinase (*ArSK*), 5-enolpyruvylshikimate-3-phosphate synthase (*ArEPSPS*), chorismate synthase (*ArCS*), and prephenate aminotransferase (*ArPPA-AT*). **(C)** Bifunctional 3-dehydroquinate dehydratase/shikimate dehydrogenase (*ArDHQD-SDH*). **(D)** Chorismate mutase (*ArCM*). **(E)** Arogenate dehydratase (*ArADT*). **(F)** β-glucogallin forming UDP glucosyltransferase (*ArUGT*). Abbreviations: TPM, transcripts per million; Sp, spring; Su, summer; Fa, fall.

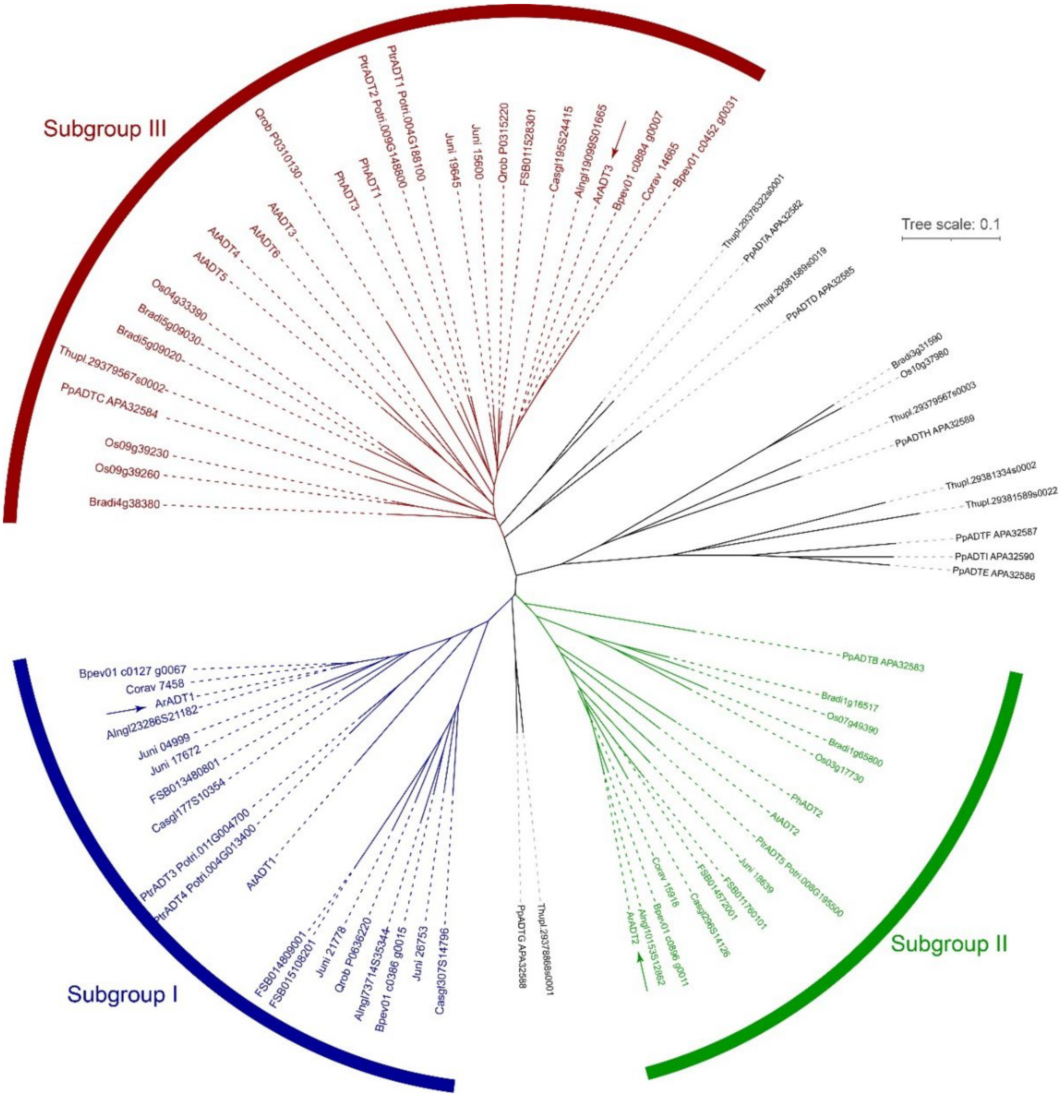

Supplementary Figure S13. Unrooted phylogenetic tree of arogenate dehydratases (ADTs) from *Alnus glutinosa* (Alngl)*,* *Alnus rubra* (Ar), *Arabidopsis thaliana* (At), *Betula pendula* (Bpev), *Brachypodium distachyon* (Bradi), *Casuarina glauca* (Casgl), *Corylus avellana* (Corav), *Fagus sylvatica* (FSB), *Juglans nigra* (Juni), *Oryza sativa* (Os) *Petunia hybrida* (Ph), *Pinus pinaster* (Pp), *Populus trichocarpa* (Ptr), *Quercus robur* (Qrob), and *Thuja plicata* (Thupl). A multiple-sequence alignment was built using Clustal Omega (57), and the unrooted phylogenetic tree was rendered with iTOL (58).

AtADT1 (At1g11790), AtADT2 (At3g07630), AtADT3 (At2g27820), AtADT4 (At3g44720), AtADT5 (At5g22630), AtADT6 (At1g08250), PhADT1 (ACY79502), PhADT2 (ACY79503), PhADT3 (ACY79504), PpADTA (APA32582), PpADTB (APA32583), PpADTC (APA32584), PpADTD (APA32585), PpADTE (APA32586), PpADTF (APA32587), PpADTG (APA32588), PpADTH (APA32589), PpADTI (APA32590), PtrADT1 (Potri.004G188100), PtrADT2 (Potri.009G148800), PtrADT3 (Potri.011G004700), PtrADT4 (Potri.004G013400), PtrADT5 (Potri.008G195500). Sequences were obtained from NCBI and from the Phytozome (v13) databases, whereas sequences from other Fagales species were retrieved (using BLASTP) from the corresponding databases as indicated in Supplementary Table S1.

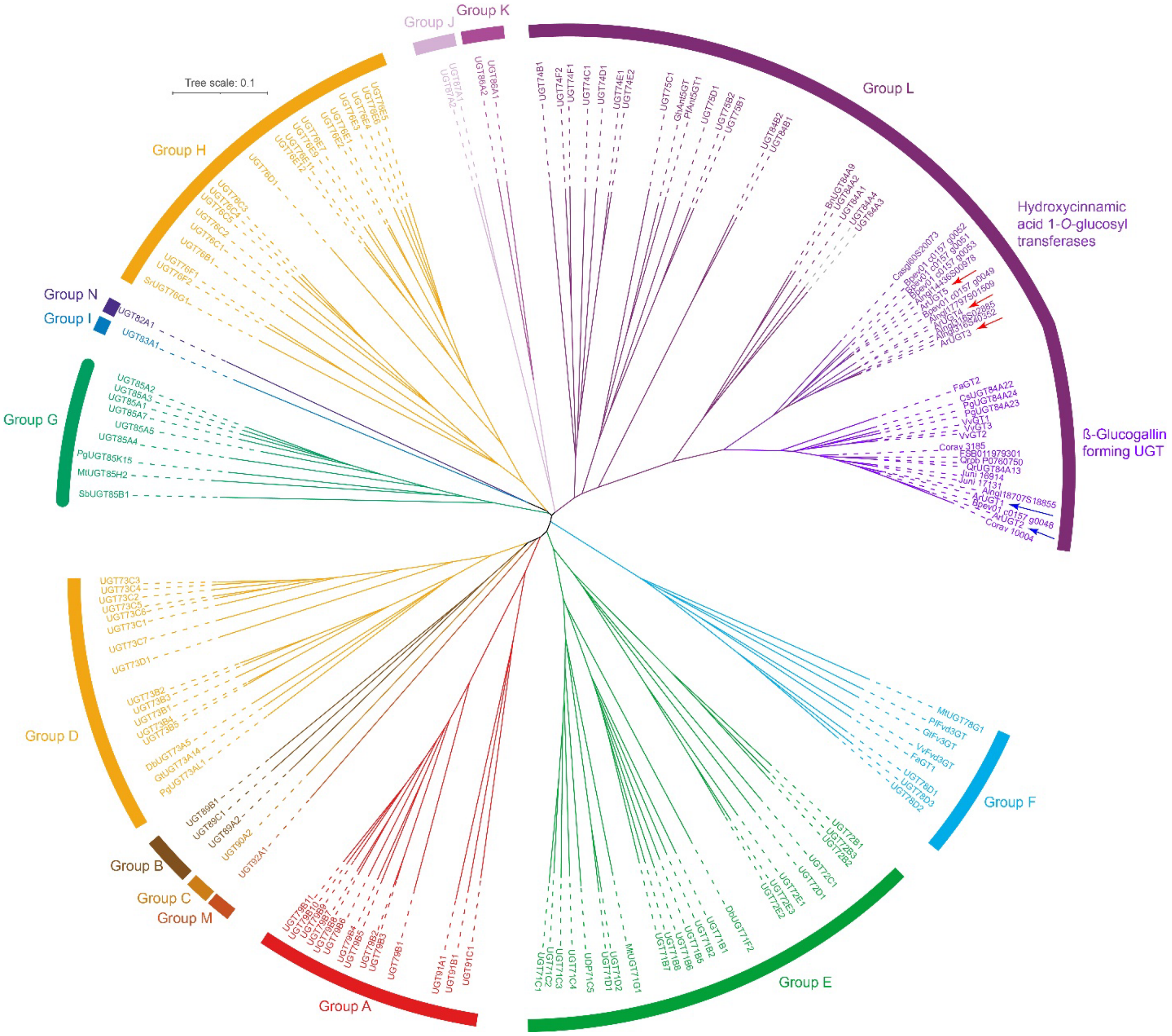

Supplementary Figure S14. Unrooted phylogenetic tree of UDP-glucosyltransferases (UGTs). Analysis used the *Arabidopsis* superfamily UGTs (59) and other UGTs of established functions. A multiple-sequence alignment was built using Clustal Omega (57), and the unrooted phylogenetic tree was rendered with iTOL (58). Red alder UGTs, ArUGT1 and 2, cluster with *β*-glucogallin forming UGTs in subgroup L and are indicated with blue arrows. ArUGT3–ArUGT5 are shown with red arrows.

Arabidopsis thaliana: UGT71B1 (AT3G23330), UGT71B2 (AT3G21760), UGT71B5 (AT4G15280), UGT71B6 (AT3G21780), UGT71B7 (AT3G21790), UGT71B8 (AT3G23390), UGT71C1 (AT2G29750), UGT71C2 (AT2G25780), UGT71C3 (AT1G07260), UGT71C4 (AT1G06880), UGT71C5 (AT1G07240), UGT71D1 (AT2G25790), UGT71D2 (AT2G29710), UGT72B1 (AT4G01070), UGT72B2 (AT1G01390), UGT72B3 (AT1G01420), UGT72C1 (AT4G36770), UGT72D1 (AT2G18570), UGT72E1 (AT3G50740), UGT72E2 (AT5G66690), UGT72E3 (AT5G26310), UGT73B1 (AT4G39040), UGT73B2 (AT4G39030), UGT73B3 (AT4G34131), UGT73B4 (AT2G15490), UGT73B5 (AT2G10670), UGT73C1 (AT2G33480), UGT73C2 (AT2G36760), UGT73C3 (AT2G36780), UGT73C4 (AT2G36770), UGT73C5 (AT2G36800), UGT73C6 (AT2G36790), UGT73C7 (AT3G53160), UGT73D1 (AT3G53150),

UGT74B1 (AT1G25270), UGT74C1 (AT2G31790), UGT74D1 (AT2G31750), UGT74E1 (AT1G05675), UGT74E2 (AT1G05160), UGT74F1 (AT2G43840), UGT74F2 (AT2G43820), UGT75B1 (AT1G05560), UGT75B2 (AT1G05530), UGT75C1 (AT4G14090), UGT75D1 (AT4G15550), UGT76B1 (AT3G11340), UGT76C1 (AT5G05870), UGT76C2 (AT5G05860), UGT76C3 (AT5G05900), UGT76C4 (AT5G05880), UGT76C5 (AT5G05890), UGT76D1 (AT2G26480), UGT76E1 (AT5G59580), UGT76E2 (AT5G59590), UGT76E3 (AT3G46700), UGT76E4 (AT3G46690), UGT76E5 (AT3G46720), UGT76E6 (AT3G46680), UGT76E7 (AT5G38040), UGT76E9 (AT5G38010), UGT76E11 (AT3G46670), UGT76E12 (AT3G46660), UGT76F1 (AT3G55700), UGT76F2 (AT3G55710), UGT78D1 (AT1G30530), UGT78D2 (AT5G16690), UGT78D3 (AT5G17030), UGT79B1 (AT5G53060), UGT79B2 (AT4G27560), UGT79B3 (AT4G27570), UGT79B4 (AT3G29630), UGT79B5 (AT1G50580), UGT79B6 (AT5G54010), UGT79B7 (AT4G09500), UGT79B8 (AT2G22930), UGT79B9 (AT5G53990), UGT79B10 (AT1G64910), UGT79B11 (AT1G64920), UGT82A1 (AT3G22250), UGT83A1 (AT3G02100), UGT84A1 (AT4G15480), UGT84A2 (AT3G21560), UGT84A3 (AT4G15490), UGT84A4 (AT4G15500), UGT84B1 (AT2G23260), UGT84B2 (AT2G18900), UGT85A1 (AT1G22400), UGT85A2 (AT1G22360), UGT85A3 (AT1G22380), UGT85A4 (AT1G78270), UGT85A5 (AT1G22370), UGT85A7 (AT1G22340), UGT86A1 (AT2G36970), UGT86A2 (AT2G28080), UGT87A1 (AT2G30150), UGT87A2 (AT2G26230), UGT89A2 (AT5G03490), UGT89B1 (AT1G73880), UGT89C1 (AT1G06000), UGT90A2 (AT1G10400), UGT91A1 (AT2G22590), UGT91B1 (AT5G65550), UGT91C1 (AT5G49690) and UGT92A1 (AT5G12890). *Brassica napus*: BnUGT84A9 (AAF98390). *Camellia sinensis*: CsUGT84A22 (ALO19890). *Cleretum bellidiforme*: DbUGT71F2 (AAL57240) and DbUGT73A5 (CAB56231). *Fragaria × ananassa*: FaGT1 (AAU09442) and FaGT2 (AAU09443). *Gentiana triflora*: GtUGT73A14 (BAC54092) and GtFv3GT (Q96493). *Glandularia hybrida*: GhAnt5GT (BAA36423). *Medicago truncatula*: MtUGT71G1 (AAW56092), MtUGT78G1 (ABI94025) and MtUGT85H2 (ABI94024). *Perilla frutescens*: PfAnt5GT1 (BAA36421) and PfFvd3GT (BAA19659). *Punica granatum*: PgUGT84A24 (ANN02877), PgUGT85K15 (ANN02874), PgUGT73AL1 (ANN02876) and PgUGT84A23 (ANN02875). *Quercus robur*: QrUGT84A13 (XP_050262807). *Sorghum bicolor*: SbUGT85B1 (AAF17077). *Stevia rebaudiana*, SrUGT76G1 (AAR06912). *Vitis vinifera*, VvGT1 (AEW31187), VvGT2 (AEW31188), VvGT3 (AEW31189) and VvFvd3GT (BAB41026). Accession numbers correspond to the NCBI database. Finally, sequences from other Fagales species were retrieved (using BLASTP) from the corresponding databases as indicated in Supplementary Table S1.

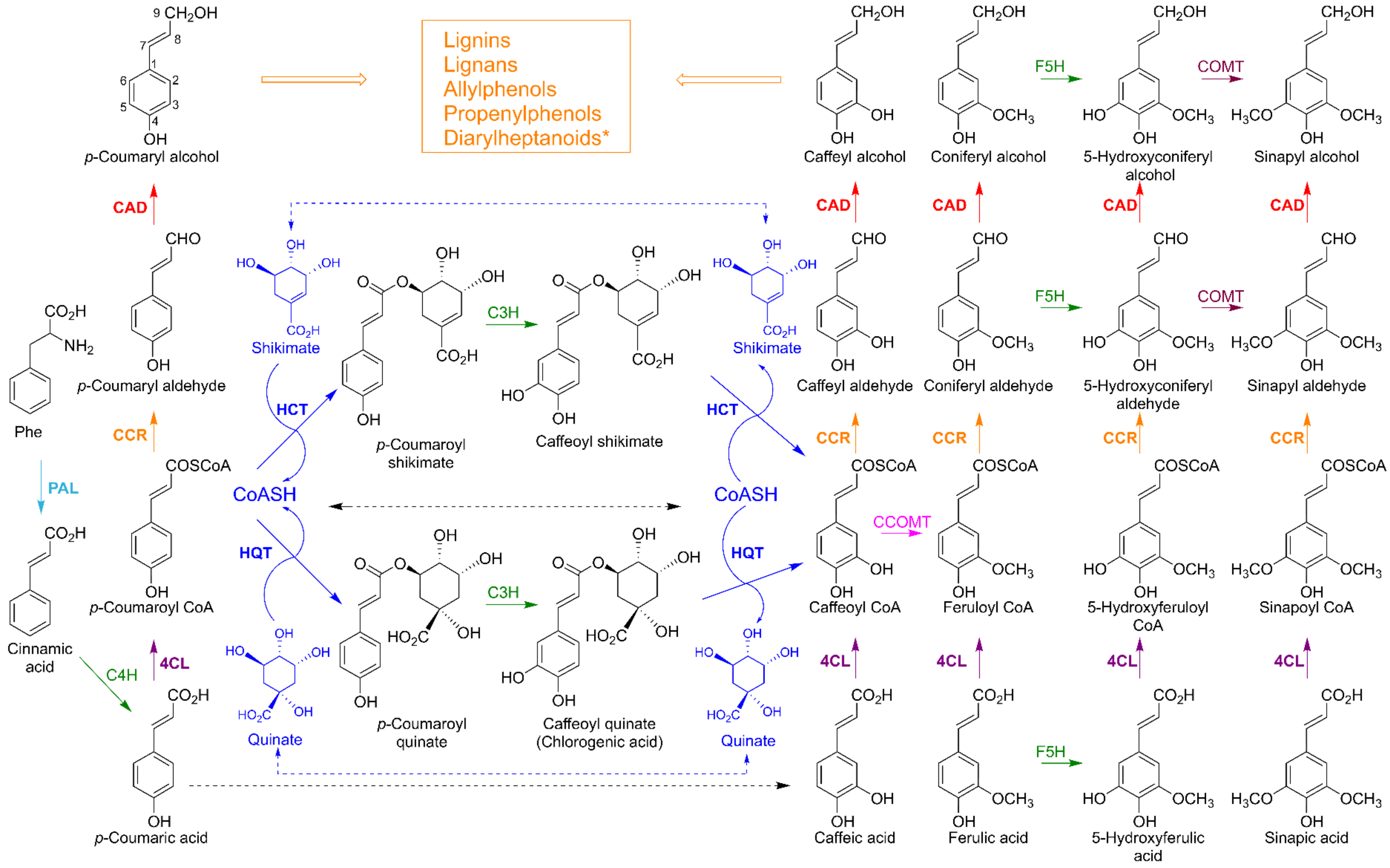

Supplementary Figure S15. Simplified angiosperm phenylpropanoid pathway to diarylheptanoids, lignins, lignans, *etc.*
4CL: 4-Coumarate CoA ligase; C4H: Cinnamate 4-hydroxylase; CAD: Cinnamyl alcohol dehydrogenase; CCOMT: Caffeoyl CoA *O*-methyltransferase; CCR: Cinnamoyl CoA reductase; COMT: Caffeic acid *O*-methyltransferase; F5H: Ferulate 5-hydroxylase; HCT: Hydroxycinnamoyl CoA:shikimate hydroxycinnamoyl transferase; HQT: Hydroxycinnamoyl CoA: quinate hydroxycinnamoyl transferase; PAL: Phenylalanine ammonia lyase; C3H: *p*-Coumarate 3-hydroxylase. NOTE: PAL, C4H, and 4CL enzymes are also common to the first three biochemical steps leading to the flavonoid metabolic class.
*Diarylheptanoids are thought to be derived from CoA esters (e.g. *p*-coumaroyl or caffeoyl CoAs).

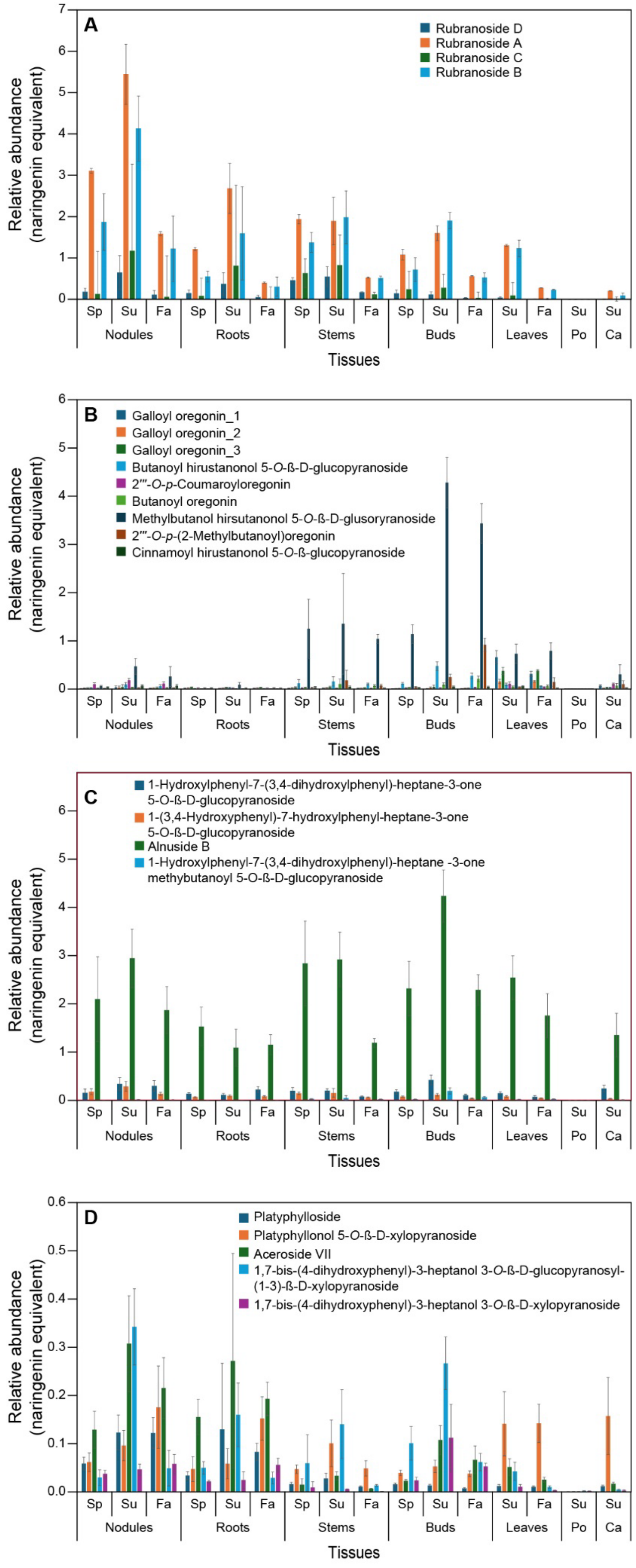

Supplementary Figure S16. Relative abundance of diarylheptanoids in naringenin equivalents.
(A) Rubranosides A to D.
(B) Hirsutanonol derived metabolites.
(C) Alnuside B and related diarylheptanoids.
(D) Platyphyloside and related diarylheptanoids.
Abbreviations: Ca, catkins; Fa, fall; Po, pollen; Sp, spring; Su, summer.

```
AtPAL1    1 MEINGAHKSNGGGVDAMLCGGDIKTKNMVINAEDPLNWGAAAEQMKGSHLDEVKRMVAEFRKPVVNLGGETLTIGQVAAI
ArPAL1    1 ----------------METTPKNGETLCVSHDP--LNWGVAAESLKGSHLDEVKRMVAEYRKPVVRLGGETLTISQVAAI
ArPAL3    1 ----------------METTPNNGETLCVSHDPDSLNWGVAAESLKSSHLDEVKRMVAEYRKPVVRLGGETLTISQVAAI
ArPAL2    1 ----------------MAACNGNGTA-TNFCTGDPLNWGAAAEALNGSHLDEVKRMVEEFRKPVVRLGGETLTISQVAAI
ArPAL4    1 ----------------MKICNNV------SESGDPLNWGAAAEALNGSHLDEVKRMVEEFRKPVVRLGGETLTISQVAAI

AtPAL1   81 STIGNSVKVELSETARAGVNASSDWVMESMNKGTDSYGVTTGFGATSHRRTKNGVALQKELIRFLNAGIFGSTKETSHTL
ArPAL1   63 ATCDSGVKVELSESARAGVKASSDWVMDSMASGTDSYGVTTGFGATSHRRTKQGAALQKELIRFLNAGIFGNGTESCHTL
ArPAL3   65 ATCDSGVKVELSESARAGVKASSDWVMDSMVSGTDTYGITTGFGAASHRRTKQGAALQKELIRFLNAGIFGNGTESCHTL
ArPAL2   64 ATHDAGVKVELSEAARPGVKASSDWVMDSMNKGTDSYGVTTGFGATSHRRTKQGGALQKELIRFLNSGIFGNGTESCHTL
ArPAL4   59 ATHDAGVKVELSEAARPGVKASSDWVMDSMNKGTDSYGVTTGFGANSHRRTKQGGALQKELIRFLNSGIFGNGTESCHTL

AtPAL1  161 PHSATRAAMLVRINTLLQGFSGIRFEILEAITSFLNNNITPSLPLRGTITASGDLVPLSYIAGLLTGRPNSKATGPNGEA
ArPAL1  143 PHSATRAAMLVRINTLLQGYSGIRFEIMEAITKFLNHNITPCLPLRGTITASGDLVPLSYIAGLLTGRPNSKAVGPSGES
ArPAL3  145 PYSATRAAMLVRINTLLQGYSGIRFEIMEAITKFLNHNITPCLPLRGTITASGDIVPLSYIAGLLTGRPNSKAVGPSGES
ArPAL2  144 PQTATRAAMLVRINTLLQGYSGIRFEVMEAITKLLNHNVTPCLPLRGTITASGDLVPLSYIAGLLTGRPNSKAIGPNGES
ArPAL4  139 PQTATRAAMLVRINTLLQGYSGIRFEVMEAITKLLNHNITPCLPLRGTITASGDLVPLSYIAGLLTGRRNSKAIGPNGES

AtPAL1  241 LTAEEAFKLAGISSGFFDLQPKEGLALVNGTAVGSGMASMVLFETNVLSVLAEILSAVFAEVMSGKPEFTDHLTHRLKHH
ArPAL1  223 LDAVEAFRLAGIDSGFFELQPKEGLALVNGTAVGSGMASMVLFEANILSVLSEILSAIFAEVMQGKPEFTDHLTHKLKHH
ArPAL3  225 LDAVEAFRLAGIDSEFFELQPKEGLALVNGTAVGSGMASMVLFEANILSVLSEILSAIFAEVMQGKPEFTDHLTHKLKHH
ArPAL2  224 LDATKAFELAGINGGFFELQPKEGLALVNGTAVGSGLASMVLFEANIQAVLSEIISAIFAEVMQGKPEFTDHLTHKLKHH
ArPAL4  219 LDATKAFELAGINGGFFELQPKEGLALVNGTAVGSGLASMVLFEANIQAVLSEIISAIFAEVMQGKPEFTDHLTHKLKHH

AtPAL1  321 PGQIEAAAIMEHILDGSSYMKLAQKLHEMDPLQKPKQDRYALRTSPQWLGPQIEVIRYATKSIEREINSVNDNPLIDVSR
ArPAL1  303 PGQIEAAAIMEHILDGSAYVKEAKKLHEMDPLQKPKQDRYALRTSPQWLGPQIEVIRYSTKSIEREINSVNDNPLIDVSR
ArPAL3  305 PGQIEAAAIMEHILDGSAFVKEAKKLHEMDPLQKPKQDRYALRTSPQWLGPQIEVIRYSTKSIEREINSVNDNPLIDVSR
ArPAL2  304 PGQIEAAAIMEHILDGSAYVKAAQKLHETDPLQKPKQDRYALRTSPQWLGPQIEVIRTSTKMIEREINSVNDNPLIDVSR
ArPAL4  299 PGQIEAAAIMEHILDGSAYVKAAQKLHETDPLQKPKHDRYALRTSPQWLGPQIEVIRTATKMIEREINSVNGNPLIDVSR

AtPAL1  401 NKAIHGGNFQGTPIGVSMDNTRLAIAAIGKLMFAQFSELVNDFYNNGLPSNLTASRNPSLDYGFKGAEIAMASYCSELQY
ArPAL1  383 NKALHGGNFQGTPIGVSMDNTRLAIASIGKLMFAQFSELVNDFYNNGLTSNLSASRNPSLDYGFKGAEIAMASYCSELQF
ArPAL3  385 NKALHGGNFQGTPIGVSMDNTRLAIASIGKLMFAQFSELVNDFYNNGLTSNLSASRNPSLDYGFKGAEIAMASYCSELQF
ArPAL2  384 NKALHGGNFQGTPIGVSMDNTRLAIASIGKLMFAQFSELVNDFYNNGLPSNLSGGRNPSLDYGFKGAEIAMASYCSELQF
ArPAL4  379 NKALYGGNFQGTPIGVSMDNTRLAIASIGKLIFAQFSELVNDFCNNGLPSNLSASRNPSLDYGFKGAEVAMASYCSELQF

AtPAL1  481 LANPVTSHVQSAEQHNQDVNSLGLISSRKTSEAVDILKLMSTTFLVAICQAVDLRHLEENLRQTVKNTVSQVAKKVLTTG
ArPAL1  463 MANPVTSHVQSAEQHNQDVNSLGLISSRKTGEAVEILKLMASTFLVALCQAIDLRHLEENLKSIVKNTVSQVAKRTLTTG
ArPAL3  465 MANPVTSHVQSAEQHNQDVNSLGLISSSKTGEAVEILKLMASTFLVALCQAIDLRHLEENLKSIVKNTLSQVAKRTLTTG
ArPAL2  464 LANPVTNHVQSAEQHNQDVNSLGLISSRKTAEAVDILKLMSSTFLVALCQAIDLRHLEENLKSTVKNTISQVAKRVLTMG
ArPAL4  459 LANPVTNHVQSAEQHNQDVNSLGLISSRKTAEAVDILKLMSSTFLVALCQAIDLRHLEENLKNTVKNTVSQVAKRVLTTG

AtPAL1  561 VNGELHPSRFCEKDLLKVVDREQVYTYADDPCSATYPLIQKLRQVIVDHALINGESEKNAVTSIFHKIGAFEEELKAVLP
ArPAL1  543 VNGELHPSRFCEKDLLKVVDREHLFAYIDDPCSATYPLMQKLRQVLVDHALSNGDSEKNANTSIFQKIGAFEEELKTLLP
ArPAL3  545 VNGELHPSRFCEKDLLKVVDREHLFAYIDDPCSATFPLMQKLRQVLVDHALSNGDSEKNANTSILQKIGAFEEELKTILP
ArPAL2  544 SNGELHPSRFCEKDLLKVVDREYVFAYIDDPCSATYPLMQKLRQVLVEHALNNGEKERNLSTSIFQKIGSFEEELKVLLP
ArPAL4  539 SNGEPHPSSFCEEDLLKVVDRENVFAYIDDPCNTTYPLMQKLRQVLLEHALNNGEKERNLSTSIFQRIGPFEEELKVLLP

AtPAL1  641 KEVEAARAAYDNGTSAIPNRIKECRSYPLYRFVREELGTELLTGEKVTSPGEEFDKVFTAICEGKIIDPMMECLNEWNGA
ArPAL1  623 KEVESARVAFESGNPAVLNRIKECRSYPLYRFVREELGTGLLTGEKDRSPGQDFDKVFTAMCQGKIIDPLLDCLRDWNGA
ArPAL3  625 KEVESARVAFESGNPAILNRIKECRSYPLYRFVREELGTGLLTGEKDRSPGQDFDKVFTAMCQGKIIDPLLDCLRDWNGA
ArPAL2  624 KEVESARTELENEKAAIPNLIKNCRSYPLYKFVREELGTGLLTGEKARSPGEEFDKVFSAMCAGKLIDPMLDCLKEWNGA
ArPAL4  619 KEVESARTELENGKAAIPNPIKNCRSYPLYKFVREELGTDLLTGEKDRSPDEEFDKVFSAMCAGKLNDPMLDCLKEWNGA

AtPAL1  721 PIPIC
ArPAL1  703 PLPIC
ArPAL3  705 PLPIC
ArPAL2  704 PLPIN
ArPAL4  699 PLPIN
```

Supplementary Figure S17. Protein sequence alignment of *bona fide* phenylalanine ammonia lyase from *Arabidopsis thaliana* (AtPAL1 (17)) and that of putative homologs (ArPAL1 – ArPAL4) in red alder.

```
HtC4H     1 ---------------------MDLLLIEKTLVALFAAIIGAILI----SKLRGKKFKLPPGPIPVPIFGNWLQVGDDLNH
ArC4H1    1 ---------------------MDLLLLEKTLLALFLAVIVAITI----SKLRGKRFKLPPGPIPVPVFGNWLQVGDDLNH
ArC4H2    1 MAHLVATPIFYTLITVLFLISATNFFFSPTLLSAALPLIPLIAYYFGSTSGRRGSGALPPGPLSFPIFGNWLQVGNDLNH
ArC4H3    1 MAHLVATPIFYTLITVLFLISATNFFFSPTLLSAALPLIPLIAYYFGSTSGRRGSGALPPGPLSFPIFGNWLQVGNDLNH

HtC4H    56 RNLTDLAKRFGEILLLRMGQRNLVVVSSPELAKEVLHTQGVEFGSRTRNVVFDIFTGKGQDMVFTVYGEHWRKMRRIMTV
ArC4H1   56 RNLTDLAKKFGDIFLLRMGQRNLVVVSSPELAKEVLHTQGVEFGSRTRNVVFDIFTGKGQDMVFTVYGEHWRKMRRIMTV
ArC4H2   81 GVLATMAQKYGPVFLLKLGSKNLAVVSSPELASQVLHTQGVEFGSRPRNVVFDIFTGNGQDMVFTVYGDHWRKMRRIMTL
ArC4H3   81 GVLATMAQKYGPVLLLKLGSKNLAVVSSPELASQVLHTQGVEFGSRPRNVVFDIFTGNGQDMVFTVYGDHWRKMRRIMTL

HtC4H   136 PFFTNKVVQQYRYGWEAEAAAVVDDVKKNPAAATEGIVIRRRLQLMMYNNMFRIMFDRRFESEDDPLFLKLKALNGERSR
ArC4H1  136 PFFTNKVVQQYRHGWEAEAASVVEDVKKNPEAATTGTVIRRRLQMMMYNNMYRIMFDRRFDSEDDPLFMKLKALNGERSR
ArC4H2  161 PFFTNKVVHSYSGMWEEEMDLVVHDLKRDERVRTKGLVIRKRLQLMLYNIMYRMMFDAKFESQEDPLFIEATKFNAERSR
ArC4H3  161 PFFTNKVVHSYSGMWEEEMDLVVHDLKRDERVRTKGLVIRKRLQLMLYNIMYRMMFDAKFESQEDPLFIEATKFNAERSR

HtC4H   216 LAQSFEYNYGDFIPILRPFLRNYLKLCKEVKDKRIQLFKDYFVDERKKIGSTKKMDNNQLKCAIDHILEAKEKGEINEDN
ArC4H1  216 LAQSFDYNYGDFIPILRPFLRSYLKICKEVKERRLQLFKDYFVDERKKLASTKPTDNEGLKCAIDHILDAQQKGEINEDN
ArC4H2  241 LAQSFEYNYGDFIPLLRPFLRGYLNKCRDLQSRRLAFFNNFYVEKRRKIMAANG-EKHKINCAIDHIIDAEMKGEISEAN
ArC4H3  241 LAQSFEYNYGDFIPLLRPFLRGYLNKCRDLQSRRLAFFNNFYVEKRRKIMAANG-EKHEINCAIDHIIDAEMKGEISEAN

HtC4H   296 VLYIVENINVAAIETTLWSIEWGIAELVNHPEIQAKLRHELDTKLGPGVQITEPDVQNLPYLQAVVKETLRLRMAIPLLV
ArC4H1  296 VLYIVENINVAAIETTLWSVEWGLAELVNHPKIQKKVRDEIDTVLGLGVQVTEPDIQKLPYLQAVVKETLRLRMAIPLLV
ArC4H2  320 VLYIVENINVAAIETTLWSMEWAIAELVNHPTVQRKIRDEISAVL-KGQPVTESNLHELPYLQATVKETLRLHSPIPLLV
ArC4H3  320 VLYIVENINVAAIETTLWSMEWAIAELVNHPTVQRKIRDEISAVL-KGQPVTESNLHELPYLQATVKETLRLHSPIPLLV

HtC4H   376 PHMNLHDAKLGGFDIPAESKILVNAWWLANNPDQWKKPEEFRPERFLEEEAKVEANGN---DFRYLPFGVGRRSCPGIIL
ArC4H1  376 PHMNLHDAKLGGFDIPAESKILVNAWWLANNPANWKNPEEFRPERFLEEESKVEANGN---DFRYLPFGVGRRSCPGIIL
ArC4H2  399 PHMNLEEAKLGGYTIPKESKVVVNAWWLSNNPEWWKNPEEFRPERFLEEEGSTDAVAGGKVDFRYLPFGMGRRSCPGIIL
ArC4H3  399 PHMNLEEAKLGGYTIPKESKVVVNAWWLSNNPEWWKNPEEFRPERFLEEEGSTDAVAGGKVDFRYLPFGMGRRSCPGIIL

HtC4H   453 ALPILGITIGRLVQNFELLPPPGQSKIDTDEKGGQFSLHILKHSTIVAKPRSF
ArC4H1  453 ALPILGITLGRLVQNFELLPPPGQSKIDTAEKGGQFSLHILKHSNIVAKPRSF
ArC4H2  479 ALPILGLVIAKLVCNFEMKAPVGMEKIDVSGKGGQFSLHIASHSTVAFDPITA
ArC4H3  479 ALPILGLVIAKLVCNFEMNAPVGMEKIDVSGKGGQFSLHIASHSTVAFDPITA
```

Supplementary Figure S18. Protein sequence alignment of *bona fide* cinnamate 4-hydroxylase from *Helianthus tuberosus* (HtC4H (18)) and that of putative homologs (ArC4H1 – ArC4H3) in red alder.

```
NtHCT     1 ------------MKIEVKESTMVKPAAETPQQRLWNSNVDLVVPNFHTPSVYFYRPTGSPNFFDGKVLKEALSKALVPFY
ArHCT1    1 ------------MIINVKESTMVRPAGETPQRALWNSNVDLVVPRFHTPSVYFYRPTGAPNFFDANVLKEALSRALVHFY
ArHCT2    1 ------------MIINVKESTMVRPGGETPKRGLWNSSLDLVVTRFHTPSVYFYRPTGAPNFFDASVLKEALSKVLVPFY
ArHCT3    1 MQVGKSEKRRAKMIISVKESTMVRPGGETPKHRLWNSSLDLVVTRFHTPSVYFYRPTGAPNFFDASVLKEALSKVLVPFY

NtHCT    69 PMAGRLCRDEDGRIEIDCKGQGVLFVEAESDGVVDDFGDFAPTLELRQLIPAVDYSQGIQSYALLVLQITHFKCGGVSLG
ArHCT1   69 PMAARLKRDEDGRIEIDCNAEGVLFVEAETTSVIDDFGDFAPTLELRQLIPAVDYSKGIESYPLLVLQVTYFKCGGVSLG
ArHCT2   69 PMAGRLKLDEEGRIEIDCNAEGVLFVEAETGSVLDDFGNFTPTMELRQLIPAVDDSKEINSFPLVVMQVTYFKCDGVSVG
ArHCT3   81 PMAGRLKLDEEGRIEIDCNAEGVLFVEAETGSVLDDFGNFTPTMELRQLIPAVDDSKGINSFPLVVMQVTYFKCGGVSVG

NtHCT   149 VGMQHHAADGASGLHFINTWSDMARGLDLTIPPFID-RTLLRARDPPQPQFPHVEYQPPPTLKVTPENTPISEAVPETSV
ArHCT1  149 VGMQHHVADGFSGLHFVNAWSDMARGLDLTIPPFIDDRTLLRARDPPKPAFQHIEYQPAPAMKTPVQS--AKSGPDSTVV
ArHCT2  149 IGTQHLLADGFSGLHFVNTWSDMARGLDLTIPPFIN-RTLLRARIPPQPAFQHVEYQPSPPMKTPRQS--I----ASTTV
ArHCT3  161 IGTQHLLADGFSGLHFVNTWSDMARGLDLTIPPFIN-RTLLRARIPPQPAFQHVEYQPSPPMKTPRQS--I----ASTMV

NtHCT   228 SIFKLTRDQINTLKAKSKEDGNTVNYSSYEMLAGHVWRSTCMARGLAHDQETKLYIATDGRSRLRPSLPPGYFGNVIFTT
ArHCT1  227 SMFRITREQLNALKAKSKDDGNTINYSSYEMLSGHVWRCTCVARALPGDQETKLYIATDGRSRLRPPLPHGYFGNVIFTA
ArHCT2  222 SMFKITRDQLNTLKAKSKDGINTVNYTSYEMLS-----------------ETKLYVAVDGRSRLHPPLPPGYFGNVIFIT
ArHCT3  234 SMFKITRDQLNTLKAKSKDGINTVNYTSYEMLSGHLWKCVSKARALPDDQETKLYVAVDGRSRLHPPLPPGYFGNVILIT

NtHCT   308 TPIAVAGDIQSKPIWYAASKLHDALARMDNDYLRSALDYLELQPDLKALVRGAHTFKCPNLGITSWSRLPIHDADFGWGR
ArHCT1  307 TPIALAGDLQSKPTWYAASRIHNALKRMDNEYLRSALDYLELQPDLSALVRGAHTFRCPNLGITSWIRLPIHDADFGWGR
ArHCT2  285 TPIALAGDLQSKPTSYAASMIHNGLARMDDEYLRSALDYLEVQPHISALVRGAHTYRCPNLGITSWSRLPIHDADFGWGR
ArHCT3  314 TPIALAGDLQSKPTSYAASMIHNGLVRMDDEYLRSALDYLEVQPDISALVRGAHTYRCPNLGITSWSRLPIHDADFGWGR

NtHCT   388 PIFMGPGGIAYEGLSFILPSPTNDGSQSVAISLQAEHMKLFEKFLYDF
ArHCT1  387 PIFMGPGGIPYEGLSFMIPSPTNDGSLSVAIALQSEHMKVFESLLYEI
ArHCT2  365 PIFMGPGGIPYEGLSYILPSPTNDGSLSVAIALQSEHMKVFENLLYDF
ArHCT3  394 PIFMGPGGIPYEGLSYILPSPTNDGSLSVAIALQSEHMKVFENLLYDF
```

Supplementary Figure S19. Protein sequence alignment of *bona fide* hydroxycinnamoyl CoA:shikimate hydroxycinnamoyl transferase from *Nicotiana tabacum* (NtHCT (19)) and that of putative homologs (ArHCT1 – ArHCT3) in red alder.

```
AtC3H     1 MSWFLIAVATIAAVVSYKLIQRLRYKFPPGPSPKPIVGNLYDIKPVRFRCYYEWAQSYGPIISVWIGSILNVVVSSAELA
ArC3H1    1 MSLPLIPISLIVLLLAYKLYQWLRFKLPPGPRPWPIVGNLYDIKPVRFRCFADWAQAYGPIISVWFGSTLNVIVSNSELA
ArC3H2    1 MALPLIPVSLIILLLAFKLYQRLRFKLPPGPRPWPIVGNLYDVKPVRFRCFAEWAQAYGPVISVWFGSMLNVIVSNSELA
ArC3H3    1 MALPLIPVSLIILLLAYKLYQRLRFKLPPGPRPWPFVGNVYNVKPVRFRCFAEWAQAYGPVISVWFGSVLNVIVSNSELA
ArC3H4    1 MALPLIPVSLIILLLAYKLYQRLRFKLPPGPRPWPFVGNVYNVKPVRFRCFAEWAQAYGPVISVWFGSVLNVIVSNSELA

AtC3H    81 KEVLKEHDQKLADRHRNRSTEAFSRNGQDLIWADYGPHYVKVRKVCTLELFTPKRLESLRPIREDEVTAMVESVFRDCNL
ArC3H1   81 KEVLKEHDMQLADRHRSRSAAKFSRDGKDLIWADYGPHYVKVRKVCTLELFSPKRLEALRPIREDEVTAMVESIFKDCTN
ArC3H2   81 KEVLKENDQQLADRYRNRSSARFSRDGKDLIWADYGPHYVKVRKVCTLELFSPKRLEALRPIREDEVRTMVESIFNDCAH
ArC3H3   81 KEVLKEHDQQLADRERNRSATMFSKDGKDLIWADYGPHYVKVRKVCTLELFSAKRLEALRPIREDEVRTMVKSIFNDCTH
ArC3H4   81 KEVLKEHDQQLADRERNRSATMFSKDGKDLIWADYGPHYVKVRKVCTLELFSAKRLEALRPIREDEVRTMVKSIFNDCTH

AtC3H   161 PENRAKGLQL-RKYLGAVAFNNITRLAFGKRFMNAEGVVDEQGLEFKAIVSNGLKLGASLSIAEHIPWLRWMFPADEKAF
ArC3H1  161 PENNGKSLLV-KKYLGAVAFNNITRLAFGKRFVNSEGVMDEQGLEFKAIVANGLKLGASLAMAEHIPWLRWMFPLEEEAF
ArC3H2  161 PYNCGKRLLV-KEYLGAVAFNNITRLTFGKRFMNSEGVMDELGLEFKGIVANGVKIGGSLPIADHIPWLRWMFPRHEEAF
ArC3H3  161 PDNCGKRLLVIKEYLGAVAFNNITRLTFGKRFMNSEGVMDEQGLEFKGIVANGVKLGGLLPIADHIPWVRWMFPQQEEAF
ArC3H4  161 PDNCGKRLLVIKEYLGAVAFNNITRLTFGKRFMNSEGVMDEQGLEFKGIVANGVKLGGLLPIADHIPWVRWMFPQQEEAF

AtC3H   240 AEHGARRDRLTRAIMEEHTLARQKSSGAKQHFVDALLTLKDQYDLSEDTIIGLLWDMITAGMDTTAITAEWAMAEMIKNP
ArC3H1  240 AKHGARRDRLTRLIMEEHTQAREKSGGAKQHFVDALLTLKDEYDLSEDTIIGLLWDMITAGMDTTAISVEWAMAELIKNP
ArC3H2  240 AKHGERRDRLTKTIMEEHTQARNKSGSAKQHFVDALFTLQDKYDLSDDTVIGLLWDMITAGTDTTAISVEWAMAELIKNP
ArC3H3  241 FKHWEMRDRLTKTIMEEHSQARNKSGEAKQHFADALFTLQEKYDLCDDTVTGLLWDMMGAGTDTTAISVEWAMAELIKNP
ArC3H4  241 FKHWEMRDRLTKTIMEEHSQARNKSGEAKQHFADALFTLQEKYDLCDDTVTGLLWDMMGAGTDTTAISVEWAMAELIKNP

AtC3H   320 RVQQKVQEEFDRVVGLDRILTEADFSRLPYLQCVVKESFRLHPPTPLMLPHRSNADVKIGGYDIPKGSNVHVNVWAVARD
ArC3H1  320 RVQHKAQEELDRVIGLERVITEADFTTLPFLQCVAKESLRLHPPTPLMLPHRANANVKIGGYDIPKGSNVHVNVWAVARD
ArC3H2  320 RVQHKVQEELDSVIGFKRFMTEADFSSLPYLQCVVKEGLRLHPPTPLMLPHRANANVKIGGYDIPKGTSVHVNVWAIARD
ArC3H3  321 RVQHKAQEEIDRVIGFERFMTEADFSSLPYLQCVVKEALRLHPPTPLMLPHRANANVKIGGYDIPKGTSVHVNVWAIARD
ArC3H4  321 RVQHKAQEEIDRVIGFERFMTEADFSSLPYLQCVVKEALRLHPPTPLMLPHRANANVKIGGYDIPKGTSVHVNVWAIARD

AtC3H   400 PAVWKNPFEFRPERFLEEDVDMKGHDFRLLPFGAGRRVCPGAQLGINLVTSMMSHLLHHFVWTPPQGTKPEEIDMSENPG
ArC3H1  400 PAVWKNPMQFRPERFLEVDVDMKGHDFRLLPFGAGRRVCPGAQLGINLVTSMIGHLLHHFVWTPPEGVKPEEIDMSENPG
ArC3H2  400 PAVWKNPLEFRPERFLEEDVDMKGHDFRLLPFGAGRRMCPGAQLAINLVTSMLGHLLHHFVWAPPEGVKLEEIDMSENPG
ArC3H3  401 PVVWKNPLEFRPERFLEEDVDMKGHDFRLLPFGAGRRMCPGVQLAINLVTSMLGHLLHHFVWAPPEGVKLEEIDMSENPG
ArC3H4  401 PVVWKNPLEFRPERFLEEDVDMKGHDFRLLPFGAGRRMCPGVQLAINLVTSMLGHLLHHFVWAPPEGVKLEEIDMSENPG

AtC3H   480 LVTYMRTPVQAVATPRLPSDLYKRVPYDM
ArC3H1  480 LVTYMRTPLQAVANPRLPSHLYKRVAVDM
ArC3H2  480 LVAYMRTPLQAMANPRLPSHLYKSVEVDM
ArC3H3  481 LVTYMRTPLQAVANPRLPSHLYKSVEVDM
ArC3H4  481 LVTYMRTPLQAVANPRLPSHLYKSVEVDM
```

Supplementary Figure S20. Protein sequence alignment of *bona fide p*-coumarate 3-hydroxylase from *Arabidopsis thaliana* (AtC3H (20)) and that of putative homologs (ArC3H1 – ArC3H4) in red alder.

```
At4CL1    1 --------MAPQEQAVSQVMEKQSNNNNSDVIFRSKLPDIYIPNHLSLHDYIFQNISEFATKPCLINGPTGHVYTYSDVH
Ar4CL1    1 ---------------------MAIQAEQQEFIFRSKLPDIYIPKHLPIHSYCFENISKFGSRPCLINGSTGEVHTYYDVE
Ar4CL2    1 ---------------------MAIQAEQQEFIFRSKLPDIYIPKHLPIHSYCFENISKFGSRPCLINGSTGEVHTYYDVE
Ar4CL3    1 ---------------------MEPQKDLQEFIFRSKLPDIYIPNHLPLHTYCFENLSQFKDRPCLINGANGVTYTYSEVE
Ar4CL4    1 MISVASNSTEPQNQEFSPKIPP-TPPSQAACVFKSKLPEIPISNHLPLHTYCFEHLAEFADRPCLIVGSTGKTFSFAETH

At4CL1   73 VISRQIAANFHKLGVNQNDVVMLLLPNCPEFVLSFLAASFRGATATAANPFFTPAEIAKQAKASNTKLIITEARYVDKIK
Ar4CL1   60 LTARKVASGLSKLGIRKGDVIMLLLPNSPEFAFVFLGASYLGAMTTAANPYFTAAEVSKQGKASNAKLVVTQSCYYDKVK
Ar4CL2   60 LTARKVASGLSKLGIRKGDVIMLLLPNSPEFAFVFLGASYLGAMTTAANPYFTATEVSKQGKASNAKLVVTQSCYYDKVK
Ar4CL3   60 LTARKVASGLDKVGIKQGEVIMLLLQNCPEFAFAFLGASYIGAVSTTANPFFTPAEVAKQAKASNAKLIITQALYVDKVK
Ar4CL4   80 LVSRKIAAGLSNLGIKKGDVVMVLLQNCAEFIFSFLGASMLGAVATTANPFYTSAEVFKQLNSSRAKLIITQSQNVDKLR

At4CL1  153 PLQNDDGV------VIVCIDDNESVPIPEGCLRFTELTQSTTEASEVIDSVEISPDDVVALPYSSGTTGLPKGVMLTHKG
Ar4CL1  140 DYAHENGV------KIICIDDSP----PKDCLHFSELTQADE---NDIPEVDISPDDVVALPYSSGTTGLPKGVMLTHKG
Ar4CL2  140 DYAHENGV------KIICIDDSP----PKDCLHFSELTQADE---NDIPEVDISPDDVVALPYSSGTTGLPKGVMLTHKG
Ar4CL3  140 DFAKENGV------KVMCIDS-Q----LDDFLHFSQLTQADE---NDIPAVKINPDDVVALPYSSGTTGLPKGVMLTHGG
Ar4CL4  160 EPEGENFPKLGEDFTVITVDD-----PPEKCLHFSVISEANE---DEFPTVSIEPDDPVALPFSSGTTGLPKGVVLTHKS

At4CL1  227 LVTSVAQQVDGENPNLYFHSDDVILCVLPMFHIYALNSIMLCGLRVGAAILIMPKFEINLLLELIQRCKVTVAPMVPPIV
Ar4CL1  207 LVTSVAQQVDGENPNLYFHSEDVILCVLPLFHIYSLNSVFLCGLRAGAAILIVQKFEIASLLELIQNYKVSVMPIVPPIV
Ar4CL2  207 LVTSVAQQVDGENPNLYFHSEDVILCVLPLFHIYSLNSVFLCGLRAGAAILIVQKFEIASLLELIQNYKVSVMPIVPPIV
Ar4CL3  206 LVTSVAQQVDGENPNLYFHSGDVILCVLPLFHIYSLNSVFLCGLRVGAAILIMQKFEIVKLLELVQQYKVTIAPFVPPIV
Ar4CL4  232 LTTSVAQQVDGENPNLYLNPEDVVLCVLPLFHIYSLNSVLLCSLRAGAAVLLMQKFEIGALLELIQRHRVSVAAVVPPLV

At4CL1  307 LAIAKSSETEKYDLSSIRVVKSGAAPLGKELEDAVNAKFPNAKLGQGYGMTEAGPVLAMSLGFAKEPFPVKSGACGTVVR
Ar4CL1  287 LSITKFPDLHKYDLSSIKMLKSGGAPLGKEIEDTVKAKFPKALFGQGYGMTEAGPVLAMCLAFAKEPMEVKSGACGTVVR
Ar4CL2  287 LSITKFPDLHKYDLSSIKMLKSGGAPLGKEIEDTVKAKFPKALFGQGYGMTEAGPVLAMCLAFAKEPMEVKSGACGTVVR
Ar4CL3  286 LAIAKSPDAQWYDVSSIRMVMSGAAPMGKELEDDVRAKLPNAKLGQGYGMTEAGSVLTMCLAFAKEPFEIKSGACGTVVR
Ar4CL4  312 LALAKNPVVANFDLSSIRVVLSGAAPLGKELEEALRNRVPQAILGQGYGMTEAGPVLSMCLGFAKQPFPTKSGSCGTVVR

At4CL1  387 NAEMKIVDPDTGDSLSRNQPGEICIRGHQIMKGYLNNPAATAETIDKDGWLHTGDIGLIDDDDELFIVDRLKELIKYKGF
Ar4CL1  367 NAEMKIVDPDTGASLPRNQSGEICIRGDQIMKGYVNDPEATARTIDKEGWLHTGDIGFIDDDDELFIVDRLKELIKYKGF
Ar4CL2  367 NAEMKIVDPDTGASLPRNQSGEICIRGDQIMKGYVNDPEATARTIDKEGWLHTGDIGFIDDDDELFIVDRLKELIKYKGF
Ar4CL3  366 NAEMKIVDPETGASLPRNQAGEIYIRGSQIMKGYLNDPEATERTIDKDGWLQTGDIGYIDGDDELFIVDRIKELIKYKGF
Ar4CL4  392 NAELKVIDPETGCSLGRNQPGEICIRGSQIMKGYLNDAEATATTIDVEGWLHTGDIGYVDDDDEIFIVDRV---------

At4CL1  467 QVAPAELEALLIGHPDITDVAVVAMKEEAAGEVPVAFVVKSKDSELSEDDVKQFVSKQVKSCVLQENQQSVLH-------
Ar4CL1  447 QVAPAELEALLLTHPNISDAAVVPMKDDLAGEVPVAFVVRSNGSQITEDEIKQFVSKQVVFYKRI---SRVFFIDVVPKS
Ar4CL2  447 QVAPAELEALLLTHPNISDAAVVPMKDDLAGEVPVAFVVRSNGSQITEDEIKQFVSKQVVFYKRI---SRVFFIDVVPKS
Ar4CL3  446 QVAPAELEAMLIAHPNIADAAVVPMKDEAAGEVPVAFVVKSNGSKISEDDIKQYISRQVVFYKRV---KRVFFTDAIPKA
Ar4CL4  463 --PPAELEALLVSHPSIADAAVVPQKDDVAGEVPVAFVVRSSDFELTEEAVKEFIAKQVVFYKRL---YKVYFVHAIPKS

At4CL1      ---------------------
Ar4CL1  524 PSGKILRKELRAKLAAGFSN-
Ar4CL2  524 PSGKILRKELRAKLAAGFSN-
Ar4CL3  523 PSGKILRKDLRAILAAGGLPD
Ar4CL4  538 PSGKILRKDLRAKLAAASPVP
```

Supplementary Figure S21. Protein sequence alignment of *bona fide* 4-coumarate CoA ligase from *Arabidopsis thaliana* (At4CL1 (21)) and that of putative homologs (Ar4CL1 – Ar4CL4) in red alder.

```
AtF5H       1 MESSISQTLSKLSDPTTSLVIVVSLFIFISFITRRRRPPYPPGPRGWPIIGNMLMMDQLTHRGLANLAKKYGGLCHLRMG
ArF5H       1 -MDSLLQALQPLH--MAAFFIIPLLFLLGIAFRIRQRLPYPPGPRGLPIIGNMGMMDQLTHRGLAKLAKQYGGILHLRIG

AtF5H      81 FLHMYAVSSPEVARQVLQVQDSVFSNRPATIAISYLTYDRADMAFAHYGPFWRQMRKVCVMKVFSRKRAESWASVRDEVD
ArF5H      78 YLHMVAVSSPDVARQVLQVQDNIFSNRPATIAISYLTYDRADMAFAHYGPFWRQMRKLCVMKLFSRKRAESWESVRDEVD

AtF5H     161 KMVRSVSCNVGKPINVGEQIFALTRNITYRAAFGSACEKGQDEFIRILQEFSKLFGAFNVADFIPYFGWIDPQGINKRLV
ArF5H     158 LIVREVAANIGKPVNIGELVFSLTKNIIYRAAFGTSSHDGQDEFIGILQEFSKLFGAFNIADFIPWLSWVDPQGLYTRLP

AtF5H     241 KARNDLDGFIDDIIDEHMKKKENQNAVDDGDVVDTDMVDDLLAFYSEEAKLVSETADLQNSIKLTRDNIKAIIMDVMFGG
ArF5H     238 R-----------------------------------------------------------------------LASHWTG

AtF5H     321 TETVASAIEWALTELLRSPEDLKRVQQELAEVVGLDRRVEESDIEKLTYLKCTLKETLRMHPPIPLLLHETAEDTSIDGF
ArF5H     246 SSTRSSTITWRRGRKIMAP-------------------------------------------------------------

AtF5H     401 FIPKKSRVMINAFAIGRDPTSWTDPDTFRPSRFLEPGVPDFKGSNFEFIPFGSGRRSCPGMQLGLYALDLAVAHILHCFT
ArF5H     265 ----------------------------------------------------------------LLKVRLIWWMIC--

AtF5H     481 WKLPDGMKPSELDMNDVFGLTAPKATRLFAVPTTRLICAL
ArF5H   ----------------------------------------
```

Supplementary Figure S22. Protein sequence alignment of *bona fide* ferulate 5-hydroxylase from *Arabidopsis thaliana* (AtF5H (22)) and that of a putative homolog (ArF5H) in red alder.

```
AtCOMT1    1 MGSTAETQLTP-------VQVTDDEAALFAMQLASASVLPMALKSALELDLLEIMAKNG--SPMSPTEIASKLPTKNPEA
ArCOMT1    1 MSSASETQMTP-------TQVSDEEANLFAMQLASASVLPMILKSAIELDLLEIMATAGPGAYLSPSEIASQLPTTNPDA
ArCOMT2    1 MSSSEQTNPNPITQQQELDQGEEEEVGKLAVRLANGVVLPMVLKSALELNLIEIISDAGTGAFLSPSEIAGRLPTKNPDA

AtCOMT1   72 PVMLDRILRLLTSYSVLTCSNRKLSGDGVERIYGLGPVCKYLTKNEDGVSIAALCLMNQDKVLMESWYHLKDAILDGGIP
ArCOMT1   74 PVMLDRILRLLASYSVLTYSLRTLPDGRVERLYGLGPVCKFLTKNEDGVSIAALNLMNQDKVLMESWYYLKDAVLEGGIP
ArCOMT2   81 PVLLDRMLRLLASYSILKCSLVTREDGEVERLYGVGPICKFLVKNPHGGSVAPLFLLHHDKVFMESWYHLNDVILEGGIP

AtCOMT1  152 FNKAYGMSAFEYHGTDPRFNKVFNNGMSNHSTITMKKILETYKGFEGLTSLVDVGGGIGATLKMIVSKYPNLKGINFDLP
ArCOMT1  154 FNKAHGMTSFEYHGKDLRFNKVFNKGMSDHSTITMKKILETYKGFEGLTSVVDVGGGTGAVLSMIVSKYPSIRGINFDLP
ArCOMT2  161 FNRAYGMTAFEYPGTDQRFNRVFNKAMSNHTTLIMKKLLDVYKGFEGLKVLVDVGGGIGVTLNIITSKYPQIEGINFDLP

AtCOMT1  232 HVIEDAPSHPGIEHVGGDMFVSVPKGDAIFMKWICHDWSDEHCVKFLKNCYESLPEDGKVILAECILPETPDSSLSTKQV
ArCOMT1  234 HVIEDAPSYPGVDHVGGDMFVSVPKGDAIFMKWICHDWSDEHCLKFLKNCYDALPNNGKVIVAECILPVAPDTSLATKGV
ArCOMT2  241 HVLADAPSYPGVEHIGGDMFDSVPEGDAIFMKWILHDWSDEHCLKLLKNCWKALPNSGKVIIVESIRPVAPESSVSANIV

AtCOMT1  312 VHVDCIMLAHNPGGKERTEKEFEALAKASGFKGIKVVCDAFGVNLIELLKKL
ArCOMT1  314 IHIDVIMLAHNPGGKERTEKEFEALAKGAGFQGFQVMCSAFNTYIMEFIKKL
ArCOMT2  321 FEQDLLMLAQNPGGKERTQKEFEALALQSGFSGCEVICCAYNSWVMEFHK—
```

Supplementary Figure S23. Protein sequence alignment of *bona fide* caffeic acid *O*-methyltransferase from *Arabidopsis thaliana* (AtCOMT1 (23, 24)) and that of putative homologs (ArCOMT1 and ArCOMT2) in red alder.

```
AtCCOMT1     1 MATTTTEATKTSSTNGEDQKQSQNLRHQEVGHKSLLQSDDLYQYILETSVYPREPESMKELREVTAKHPWNIMTTSADEG
ArCCOMT1     1 -----------MAFAYELADLEQAGSHQGFGHKSLLQSDALYQYILDTSVYPREHEAMKELREMTAKHPWNTMTTSADEN
ArCCMOT2     1 -----------MAFAYGLADLEQAGSHQGFGHKSLLQSDALYQYILDTSVYPREHEAMKELREMTAKHPWNSMTTSADEN
ArCCOMT3     1 --------------------MEQNLKFIPSRSKSLLQSDDLYQYILETSVYPREPELLKELRHITAGHPRARMATAPDAA

AtCCOMT1    81 QFLNMLIKLVNAKNTMEIGVYTGYSLLATALALPEDGKILAMDVNRENYELGLPIIEKAGVAHKIDFREGPALPVLDEIV
ArCCOMT1    70 QFLSMLLKLINAKNTMEIGVYTGYSLLATALALPDDGKILAMDVNREHYELGLPIIKKAGVAHKIDFREGPALPVLDKLV
ArCCMOT2    70 QFLSMLLKLINAKNTMEIGVYTGYSLLATALALPDDGKILAMDVNREHYELGLPIIKKAGVAHKIDFREGPALPVLDKLV
ArCCOMT3    61 QLIAMLLKLVNPKKTIEVGVFTGYSLLLTALTIPEDGKITAIDINQETFEIGLPIIKKAGVADKINFIESEALPVLDQLL

AtCCOMT1   161 ADEKNHGTYDFIFVDADKDNYINYHKRLIDLVKIGGVIGYDNTLWNGSVVAPPDAPMRKYVRYYRDFVLELNKALAADPR
ArCCOMT1   150 EDEKNHGTFDFVFVDADKDNYLNYHERLIKLVKVGGLIGYDNTLWNGSVAAPADAPLPEYLTYYRQFVVELNNALAVDPK
ArCCMOT2   150 EDEKNHGTFDFVFVDADKDNYLNYHERLIKLVKVGGLIGYDKHPLERLCGGTC---------------------------
ArCCOMT3   141 QTHENEGSFEFAFIDADKVNYWSYHERLMKLLKVGGVVVYDNTLWGGTVAMP-DELVPEFLKQGRQFTIEFNKLLVADPR

AtCCOMT1   241 IEICMLPVGDGITICRRIS
ArCCOMT1   230 IEICMLSVGDGITLCRRIK
ArCCMOT2       -------------------
ArCCOMT3   220 VQISHASIGDGITICKRLY
```

Supplementary Figure S24. Protein sequence alignment of *bona fide* caffeoyl CoA *O*-methyltransferase from *Arabidopsis thaliana* (AtCCOMT1 (25)) and that of putative homologs (ArCCOMT1 – ArCCOMT3) in red alder.

```
AtCCR1       1 MPVDVASPAG-KTVCVTGAGGYIASWIVKILLERGYTVKGTVRNPDDPKN-THLRELEGGKERLILCKADLQDYEALKAA
ArCCR1       1 MPVDCSSASGLSTVCVTGAGGFIASWIVKLLLEKGYTVKGTLRNPDDPKN-AHLKELEGAQERLTLWKTDLLDYESLKVA
ArCCR2       1 ------MSGAEKVVCVTGASGYIASWLVKLLLQRGYTVKASVRDPNDPKKMEHLLVFDGAKERLQLFKADLLEEGAFDSV

AtCCR1      79 IDGCDGVFHTASPVT----DDPEQMVEPAVNGAKFVINAAA-EAKVKRVVITSSIGAVYM-DPNRDPEAVVDESCWSDLD
ArCCR1      80 IDGCDGVIHTASPVT----DDPELMVEPAVDGTKNVIIAAA-EAKLRRVVFTSSIGAVYM-DPNRGPDVVVDESCWSDLE
ArCCR2      75 VHGCEGVFHTASPCYHNVTDPQAEFIDPALKGTLNVLRSCAKVPTVKRVVITSSMQAVAFNGKSLAPDVIIDETWFSDPA

AtCCR1     153 FCKNTKNWYCYGKMVAEQAAWETAKEKGVDLVVLNPVLVLGPPLQPTINASLYHVLKYLTGSAKTYANLTQAYVDVRDVA
ArCCR1     154 FCKNTKNWYCYGKAVAEQAAWEVAKEKGVDLVVVNPVLVLGPLLQPNVNASVVHVLKYLTGSAKTYANSVQAYVHVRDVA
ArCCR2     155 VCEKSKSWYRLSKTLAEEAAWKFAKENGIDMVVINPGLVIGPLLQPTLNSSAETVLKLVNGA-ERFPNVTYRLVDVRDVA

AtCCR1     233 LAHVLVYEAPSASGRYLLAESARHRGEVVEILAKLFPEYPLPTKCKDEKNPRAKPYKFTNQKIKDLGLEFTSTKQSLYDT
ArCCR1     234 LAHILVLETPSASGRYLCAESVLHRGVVVQILAKLFPEYPIPTKCSDEKNPRAKPYKFSNQKLKELGLEFTPVKQCLYET
ArCCR2     234 NAHILAFEKPSASGRYCLVGRVIHCSEVIKMLRELFPDLNLPEKCADDK-PFLSTYKVSKERAESLGLNFTPVEVSLKDT

AtCCR1     313 VKSLQEKGHLAPPPPPPSASQESVENGIKIGS
ArCCR1     314 VKSLQEKGVLPVPTQQQE----SI----RIQS
ArCCR2     313 VESLKEKNFLCA--------------------
```

Supplementary Figure S25. Protein sequence alignment of *bona fide* cinnamoyl CoA reductase from *Arabidopsis thaliana* (AtCCR1 (26)) and that of putative homologs (ArCCR1 and ArCCR2) in red alder.

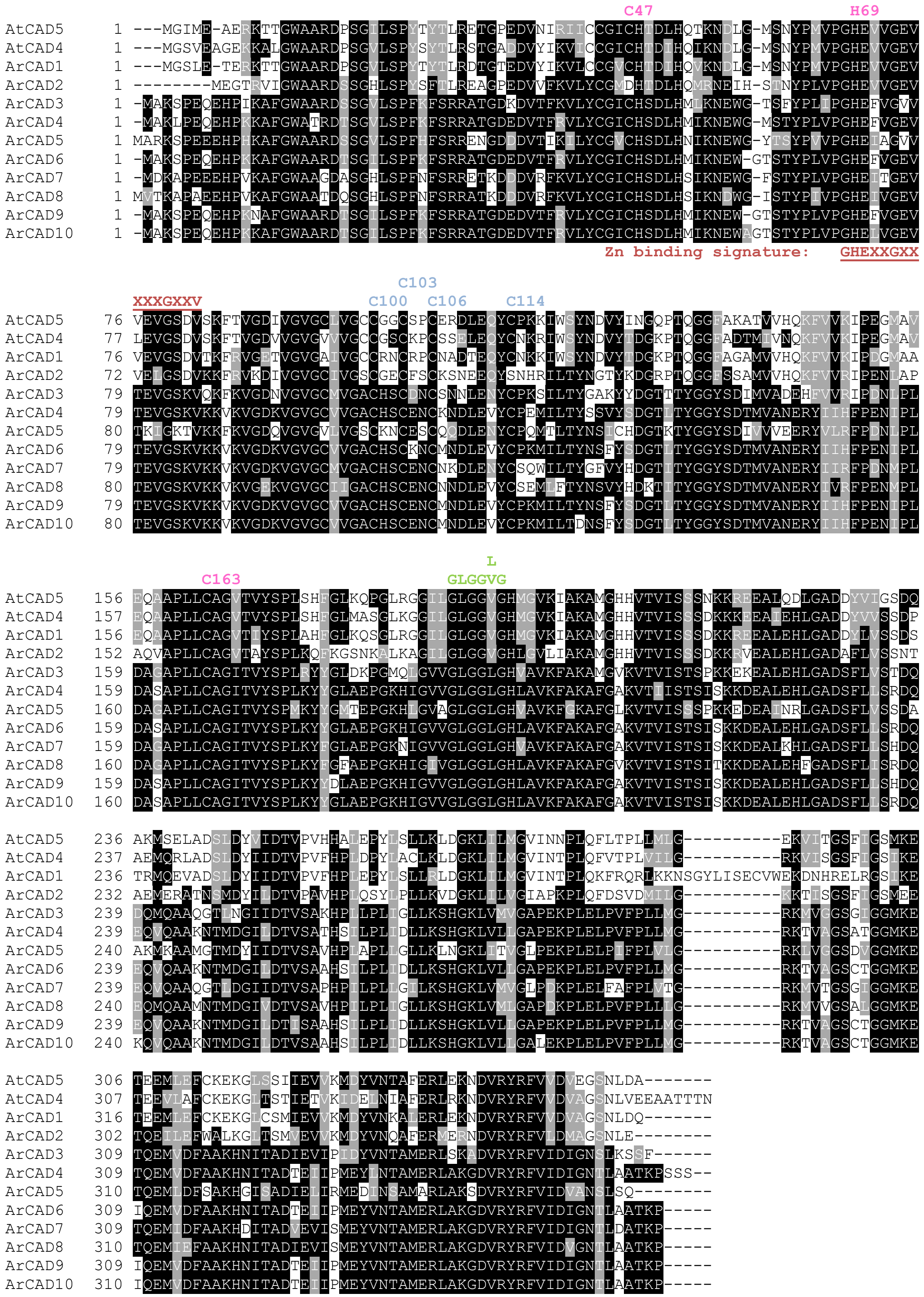

Supplementary Figure S26. Protein sequence alignment of *bona fide* cinnamyl alcohol

dehydrogenases from *Arabidopsis thaliana* (AtCAD4 and AtCAD5 (27)) and that of putative homologues (ArCAD1 – ArCAD10) in red alder. Highly conserved Zn1 catalytic center (C47, H69, and C163), the Zn-binding signature GHEXXGXXXXXGXXV, the Zn2 structural motif (C100, C103, C106, and C114), and the NADPH-binding domain [**GLGGV(L)G**] motif are highlighted.

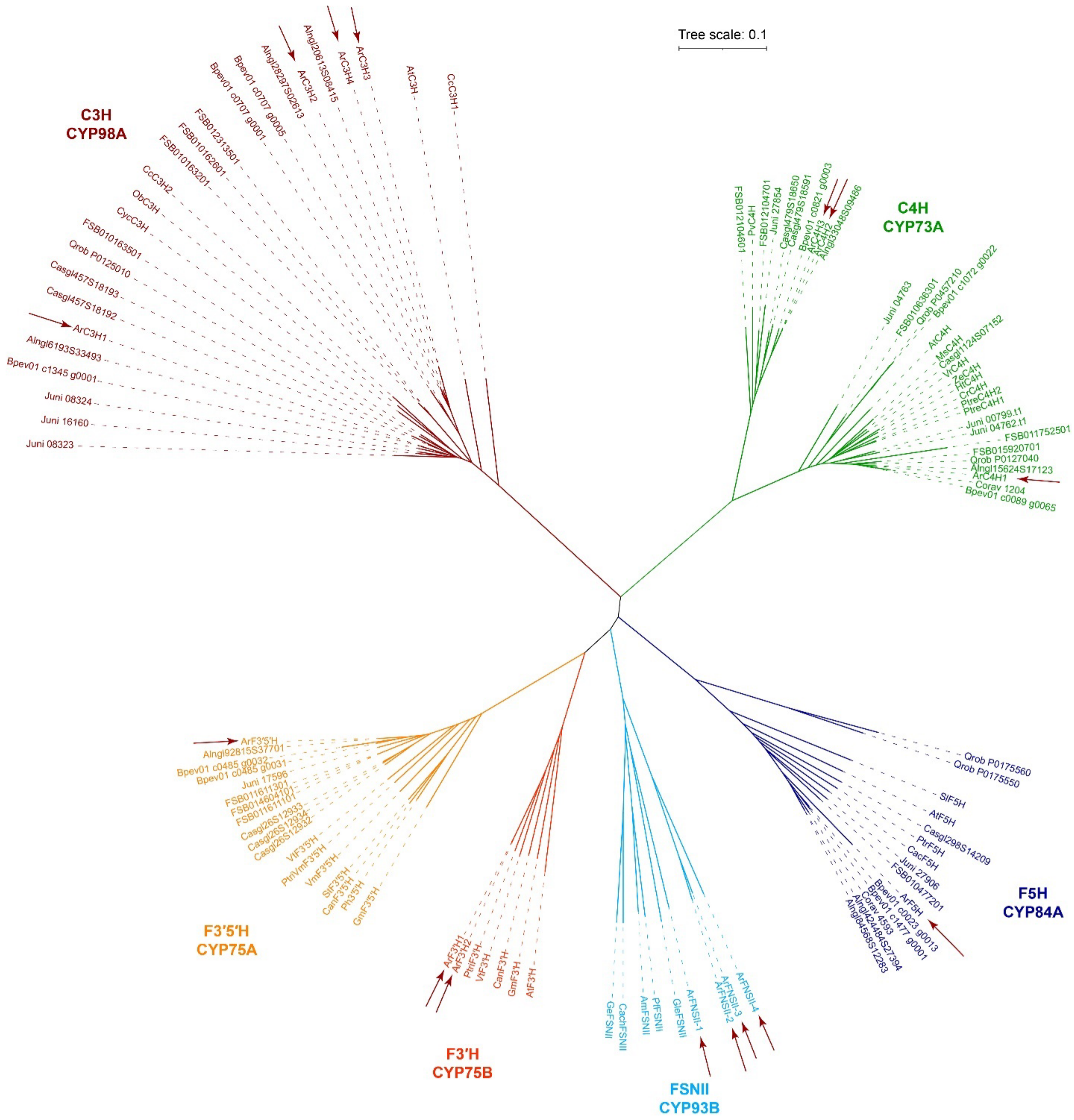

Supplementary Figure S27. Unrooted phylogenetic tree of hydroxylases involved in phenylpropanoid (C4H, C3H and F5H) and phenylpropanoid-acetate (F3′H, F3′5′H and FSNII) pathways from *Alnus glutinosa* (Alngl)*, Alnus rubra* (Ar), *Antirrhinum majus* (Am), *Arabidopsis thaliana* (At), *Betula pendula* (Bpev), *Callistephus chinensis* (Cach), *Camptotheca acuminata* (Cac), *Capsicum annuum* (Can), *Casuarina glauca* (Casgl), *Catharanthus roseus* (Cr), *Coffea canephora* (Cc), *Corylus avellana* (Corav), *Cynara cardunculus* (Cyc), *Fagus sylvatica* (FSB), *Gerbera* hybrid cultivar (Ge), *Glycine max* (Gm), *Glycyrrhiza echinata* (Gle), *Helianthus tuberosus* (Ht), *Juglans nigra* (Juni), *Medicago sativa* (Ms), *Ocimum basilicum* (Ob), *Perilla frutescens* (Pf), *Petunia* × *hybrida* (Ph), *Phaseolus vulgaris* (Pv), *Populus tremuloides* (Ptre), *Populus trichocarpa* (Ptri), *Quercus robur* (Qrob), *Solanum lycopersicum* × *S. peruvianum* (Sl), *Solanum tuberosum* (St), *Vigna radiata* (Vr), *Vinca major* (Vm), *Vitis vinifera* (Vt) and *Zinnia elegans* (Ze). Sequences were obtained from NCBI and a multiple-

sequence alignment was built using Clustal Omega (57), with the unrooted phylogenetic tree rendered with iTOL (58). *Dark red* arrows show red alder hydroxylases.

AmFSNII (CYP93B3; BAA84071), AtC3H (CYP98A3; At2g40890), AtC4H (CYP73A5, At2g30490), AtF3′H (CYP75B1, At5g07990), AtF5H (CYP84A1), CacF5H (AAT39511), CachFSNII (CYP93B5; AAF04115), CanF3′,5′H (QGZ19237), CanF3′H (XP_016563358), CcC3H1 (CYP98A35; ABB83676), CcC3H2 (CYP98A36; ABB83677), CrC4H (CAA83552), CycC3H1 (CYP98A49; ACO25188), GeFSNII (CYP93B2; AAD39549), GleFSNII (CYP93B1; P93149), GmF3′,5′H (AAM51564), GmF3′H (BAB83261), HtC4H (CAA78982), MsC4H (L11046), ObC3H (CYP98A13; AAL99200), PfFSNII (CYP93B6; BAB59004), PhF3′,5′H (CAA80266), PtreC4H1 (ABF69099), PtreC4H2 (ABF69101), PtrF5H (CAB65335), PtriF3′H (XP_002319761), PtriVmF3′,5′H (XP_002314004), PvC4H (CAA70595), SlF5H (AAD37433), StF3′,5′H (NP_001274807), VmF3′,5′H (BAC97831), VrC4H (P37115), VtF3′,5′H (BAE47007), VtF3′H (BAE47004), ZeC4H (Q43240). Sequences from other Fagales species were retrieved (using BLASTP) from the corresponding databases as indicated in Supplementary Table S1.

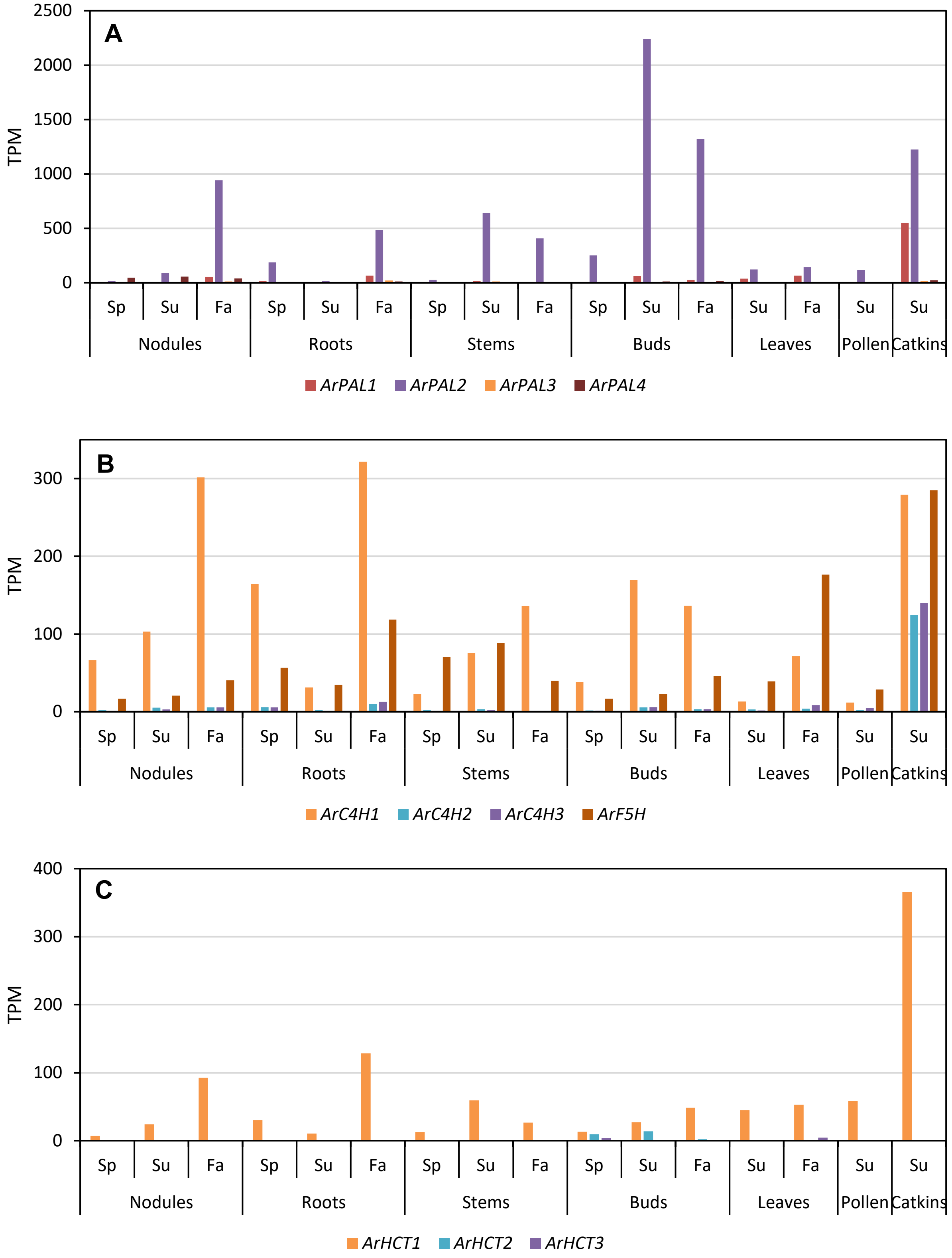
A
2500
2000
1500
1000
500
0
TPM
Sp Su Fa Sp Su Fa Sp Su Fa Sp Su Fa Su Fa Su Su
Nodules Roots Stems Buds Leaves Pollen Catkins
ArPAL1 ArPAL2 ArPAL3 ArPAL4
B
300
200
100
0
TPM
Sp Su Fa Sp Su Fa Sp Su Fa Sp Su Fa Su Fa Su Su
Nodules Roots Stems Buds Leaves Pollen Catkins
ArC4H1 ArC4H2 ArC4H3 ArF5H
C
400
300
200
100
0
TPM
Sp Su Fa Sp Su Fa Sp Su Fa Sp Su Fa Su Fa Su Su
Nodules Roots Stems Buds Leaves Pollen Catkins
ArHCT1 ArHCT2 ArHCT3

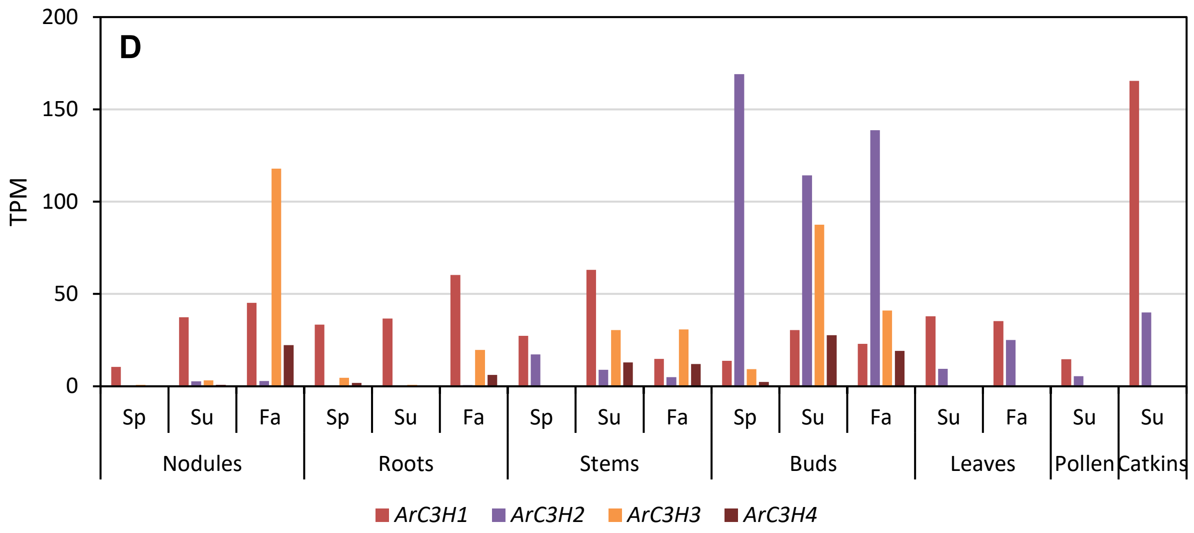
D
200
150
100
50
0
TPM
Sp Su Fa Sp Su Fa Sp Su Fa Sp Su Fa Su Fa Su Su
Nodules Roots Stems Buds Leaves Pollen Catkins
ArC3H1 ArC3H2 ArC3H3 ArC3H4

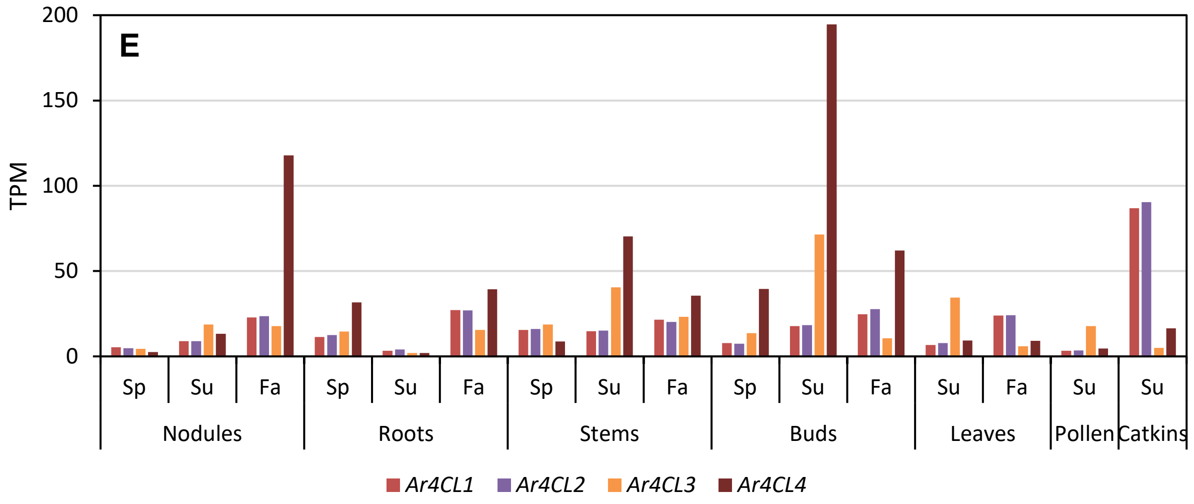
E
200
150
100
50
0
TPM
Sp Su Fa Sp Su Fa Sp Su Fa Sp Su Fa Su Fa Su Su
Nodules Roots Stems Buds Leaves Pollen Catkins
Ar4CL1 Ar4CL2 Ar4CL3 Ar4CL4

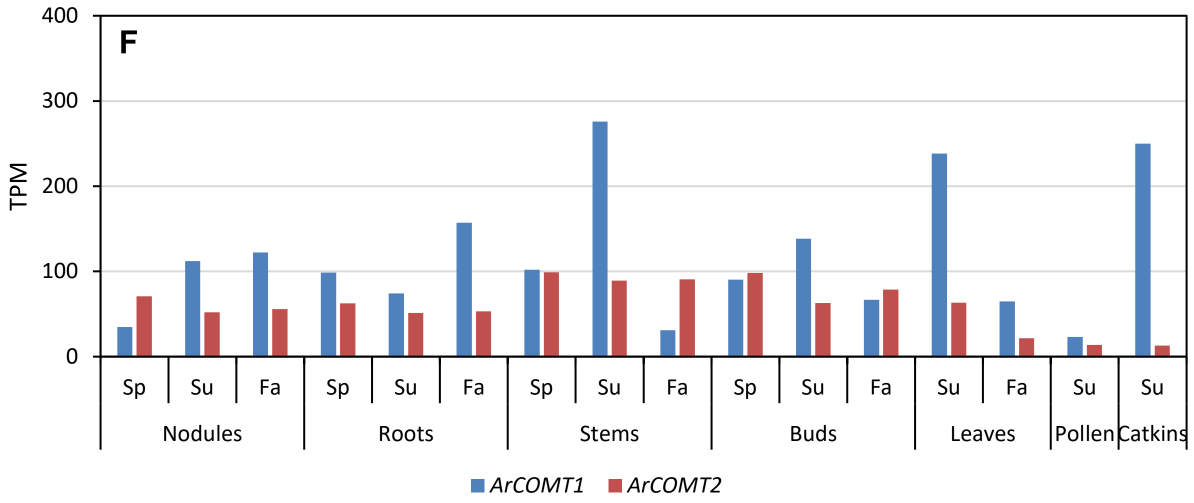
F
400
300
200
100
0
TPM
Sp Su Fa Sp Su Fa Sp Su Fa Sp Su Fa Su Fa Su Su
Nodules Roots Stems Buds Leaves Pollen Catkins
ArCOMT1 ArCOMT2

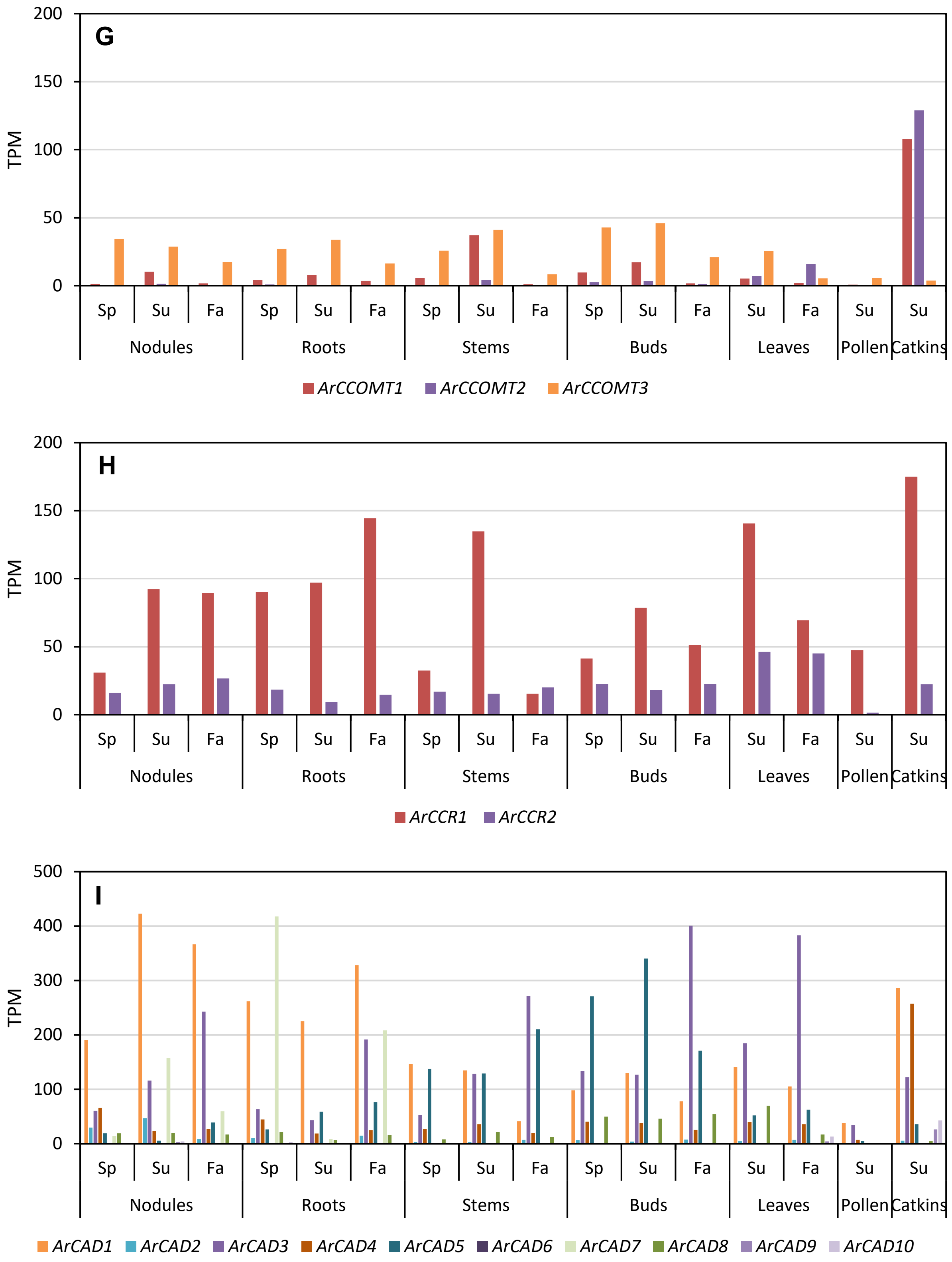

Supplementary Figure S28. Expression levels of red alder homologs of *bona fide* phenylpropanoid pathway genes. **(A)** Phenylalanine ammonia lyase (*ArPAL*). **(B)** Cinnamate 4-hydroxylase (*ArC4H*) and ferulate 5-hydroxylase (*ArF5H*). **(C)** Hydroxycinnamoyl CoA: shikimate hydroxycinnamoyl

transferase (*ArHCT*). **(D)** *p*-Coumarate 3-hydroxylase (*ArC3H*). **(E)** 4-Coumarate CoA ligase (*Ar4CL*). **(F)** Caffeic acid *O*-methyltransferase (*ArCOMT*). **(G)** Caffeoyl CoA *O*-methyltransferase (*ArCCOMT*). **(H)** Cinnamoyl CoA reductase (*ArCCR*). **(I)** Cinnamyl alcohol dehydrogenase (*ArCAD*). Abbreviations: TPM, transcripts per million; Sp, spring; Su, summer; Fa, fall.

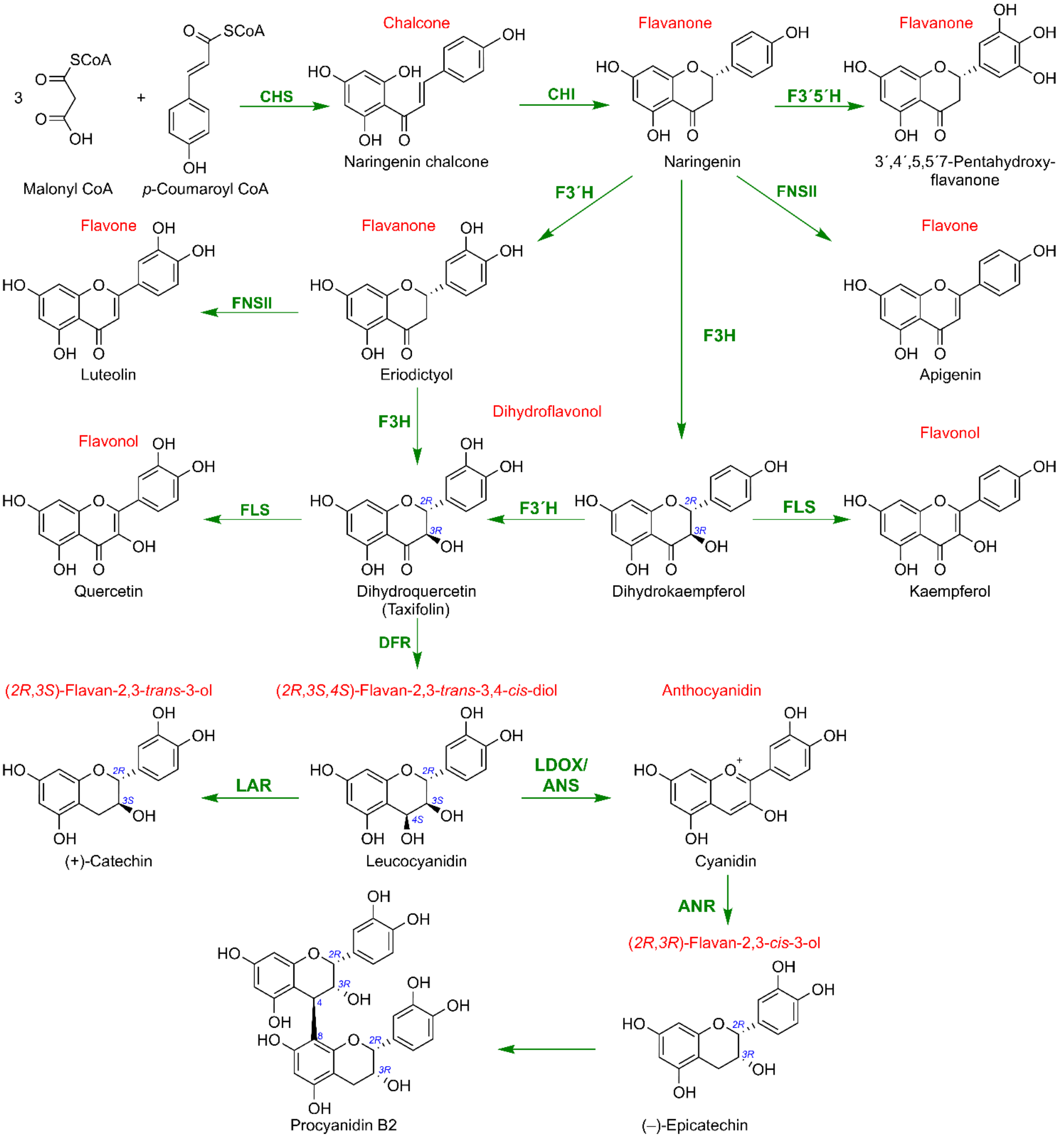

Supplementary Figure S29. Simplified flavonoid biochemical pathway. ANR: Anthocyanidin reductase; CHI: Chalcone isomerase; CHS: Chalcone synthase; DFR: Dihydroflavonol 4-reductase; F3′,5′H: Flavonoid 3′,5′-hydroxylase; F3′H: Flavonoid 3′-hydroxylase; F3H: Flavanone 3-hydroxylase; FLS: Flavonol synthase; FNSII: Flavone synthase II; LAR: Leucoanthocyanidin reductase; LDOX/ANS: Leucoanthocyanidin dioxygenase/anthocyanidin synthase.

```
AmCHS     1 MVTVEEVRRAQRAEGPATVLAIGTATPANCVDQSTYPDYYFRITNSEHMTELKEKFKRMCDKSNIKRRYMHLTEEILKEN
ArCHS1    1 MVTVDEVRKAQRAQGPATVMAIGTATPPNCVEQSTYPDYYFRITKSEHKTELKEKFKRMCEKSMIKKRYMYLTEEILKEH
ArCHS2    1 MASVDEIFKAQRAQGPATVLAIGTANPSHCIYQDDFPDYYFRVTKSEHMTDLKRKFKRICEKSMIKKRYTHLTEDILKEN
ArCHS3    1 MASVEEIRKAQRAQGPATILAIGTATPSNCVSQADYPDFYFRITKSDHMTELKEKFKRMCDKSMIKKRYMYLNEEILKEN
ArCHS4    1 --------------------------------------------------------------------------------

AmCHS    81 PAMCEYMAPSLDARQDIVVVEVPRLGKEAAQKAIKEWGQPKSKITHLVFCTTSG-VDMPGADYQLTKLLGLRPSVKRFMM
ArCHS1   81 PNLCAYMAPSLDARQDMVVVEIPKLGKEAATKAIKEWGQPKSKITHLVFCTTSG-VDMPGADYQLTKLLGLRPSVKRLMM
ArCHS2   81 PSMCTFMEPSLDARQEIGVVELPKLAREAASKAIKEWGQPKSKITHLIFSTTLGTSDMPGADYQLTKLLGLKSSVKRIMM
ArCHS3   81 PNMCAYMAPSLDARQDIVVVEVPKLGKEAATKAIKEWGQPKSKITHLVFCTTSG-VDMPGADYQLTKLLGLRPSVKRLMM
ArCHS4    1 ---------------------------------------------------------MPGADYQLTKLLGLKSSVKRIMM

AmCHS   160 YQQGCFAGGTVLRMAKDLAENNAGARVLVVCSEITAVTFRGPADTHLDSLVGQALFGDGAAAVIVGSDPVVGVERPLFQI
ArCHS1  160 YQQGCFAGGTVLRLAKDLAENNKGARVLVVCSEITAVTFRGPSDSHLDSLVGQALFGDGAAALIVGADPVPEVEKPLFEL
ArCHS2  161 YQHGCFGGGTVLRVAKDFAENHKGARVLAVCAEITASNFRGPSESHFDALVGQSLFGDGAAALIVGADPDTSVERPLFQI
ArCHS3  160 YQQGCFAGGTVLRLAKDLAENNKGARVLVVCSEITAVTFRGPTDTHLDSLVGQALFGDGAAALIVGADPDTPVERPLFEL
ArCHS4   24 YQHGCFGGGTVLRVAKDFAENHKGARVLAVCAEINASCFRGPYASHLDALVGQSLFGDGAAAVLVGADPDTGVERPLFQI

AmCHS   240 VTAAQTLLPDSHGAIDGHLREVGLTFHLLKDVPGLISKNIEKSLKEAFDPLGISDWNSVFWIAHPGGPAILDQVEEKLGL
ArCHS1  240 VSAAQTILPDSDGAIDGHLREVGLTFHLLKDVPGLISKNIEKSLVEAFQPLGITDWNSLFWVAHPGGPAILDQVESKLGL
ArCHS2  241 VSATQTLLPDSDGAVQGHMLERGLTFHLLKDLPKIASTNIESSLVESFGPIGISDWNSLFWIVHPGGPAILDQVEARLGL
ArCHS3  240 ISAAQTILPDSDGAIDGHLREVGLTFHLLKDVPGIISKNIEKSLAEAFAPLGISDWNSLFWIAHPGGPAILDQVESKLGL
ArCHS4  104 VSATQTLLLDSDGSVQGHMLERGRTFHLLKDLPKIASTNIERSLVESFSPIGISDWNSLFWIVHPGGPAIL--VEARLGL

AmCHS   320 KPEKLRSTRQVLSEYGNMSSACVLFILDEMRKSSAKEGMSTTGEGLDWGVLFGFGPGLTVETVVLHSVPLN---
ArCHS1  320 KAEKLAATRHVLSEYGNMSSACVLFILDEMRKKSAENGLKTTGEGLEWGVLFGFGPGLTVETVVLHSLST----
ArCHS2  321 EEEKLKASRHVLSEYGNMSTACVLFILDEIRKRSVEEGKATTGGGLDWGVLFGLGPGLTVETVVLHSVPLETLH
ArCHS3  320 KEEKLRATRHILSEYGNMSSACVLFILDEMRKNSAEKGKVTTGEGLEWGVLFGFGPGLTVETVVLHSIPVPV--
ArCHS4  182 EEEKLRASRHVLSEFGNMSTACVLFIFG----------------------------------------------
```

Supplementary Figure S30. Protein sequence alignment of *bona fide* chalcone synthase from *Antirrhinum majus* (AmCHS (28)) and that of putative homologs (ArCHS1 – ArCHS4) in red alder.

```
PhCHI     1 MSPSVSVTKVQVENYVFPPTVKPPASTKTLFLGGAGHRGLDVEGKFVKFTVIGVYLEESAVQFLAPKWKGKSAEELIHSV
ArCHI     1 MVSPLSLSGVQVENVEFPPTVKPPGSTKTLFLGGAGERGLEIQGKFVKFTAIGVYLEDTAVPSLAVKWKGKSAEELTESV

PhCHI    81 DFFRDIVTGPFEKFTRVRFILPLTGKQFSEKVAENCVAHWKATGTYSDAGSRAIEKFLNVVKSETFLPGASILFTQSPLG
ArCHI    81 EFFRDIVTGPFEKFIRVTMILPLTGQQYSEKVSENCVAAWKSVGVYTDAEGKAIEKFLEVFKDEKFPPGSSILFTQSPNG

PhCHI   161 SLTISFTKDDSISEAGNAVIENKQFSEAVLETIIGEHGVSPAAKCSIAARMSELFKNSLF-------
ArCHI   161 SLAIGFSKDGSIPEIGNAVIENKLLSGAVLESIIGKHGVSPAAKQSLAIRLSELLAEKGKPETEKVT
```

Supplementary Figure S31. Protein sequence alignment of *bona fide* chalcone isomerase from *Petunia × hybrida* (PhCHI (29)) and that of a putative homolog (ArCHI) in red alder.

```
AtF3H     1 MAPGTLTELAGESKLNSKFVRDEDERPKVAYNVFSDEIPVISLAGIDDVDGKRGEICRQIVEACENWGIFQVVDHGVDTN
ArF3H     1 MAPTTLTALAGEKTLQTSFVRDEDERPKVAYNQFSSEIPVISLAGIDEVDGRRAEICKKIVDACEDWGIFQILDHGVDAN

AtF3H    81 LVADMTRLARDFFALPPEDKLRFDMSGGKKGGFIVSSHLQGEAVQDWREIVTYFSYPVRNRDYSRWPNKPEGWVKVTEEY
ArF3H    81 LISDMTRLAREFFALPPDEKLRFDMSGGKKGGFIVSSHLQGEAVQDWREIVTYFSYPIRSRDYSRWPDKPEAWRAVTEQY

AtF3H   161 SERLMSLACKLLEVLSEAMGLEKESLTNACVDMDQKIVVNYYPKCPQPDLTLGLKRHTDPGTITLLLQDQVGGLQATRDN
ArF3H   161 SDRLMELACKLLEVLSEAMGLEKEALTKACVDMDQKVVVNYYPKCPQPDLTLGLKRHTDPGTITLLLQDQVGGLQATRDG

AtF3H   241 GKTWITVQPVEGAFVVNLGDHGHFLSNGRFKNADHQAVVNSNSSRLSIATFQNPAPDATVYPLKVREGEKAILEEPITFA
ArF3H   241 GKTWITVQPVEGAFVVNLGDHGHFLSNGRFKNADHQAVVNSNYSRLSIATFQNPAPEAKVYPLKIREGEKAVLEAPITFA

AtF3H   321 EMYKRKMGRDLELARLKKLAKEERDH--KEVAKPVDQIFA
ArF3H   321 EMYKRKMSKDIELARLKKLAKEQDLEKDKMDAKPIEDIFA
```

Supplementary Figure S32. Protein sequence alignment of *bona fide* flavanone 3-hydroxylase from *Arabidopsis thaliana* (AtF3H (30)) and that of a putative homolog (ArF3H) in red alder.

```
AtF3'H   MATLFLTILLATVLFLILRIFSHRRNRSHNNRLPPGPNPWPIIGNLPHMGTKPHRTLSAMVTTYGPILHLRLGFVDVVVA
ArF3'H1  -MSPFSLILGTVALAIFLYYLLNLRTHRHRLPLPPGPKPWPIIGNLPHMGTVPHHSLAAMARTYGPLMHLRLGFVDVVVA
ArF3'H2  -MSPFSLILGTVALAIFLYYLLNLRTHRHRLPLPPGPKPWPIIGNLPHMGTVPHHSLAAMARTYGPLMHLRLGFVDVVVA

AtF3'H   ASKSVAEQFLKIHDANFASRPPNSGAKHMAYNYQDLVFAPYGHRWRLLRKISSVHLFSAKALEDFKHVRQEEVGTLTREL
ArF3'H1  ASASVAAQFLKHHDSNFSSRPPNSGAKYIAYNYQDLVFAPYGDRWRMLRKISSVHLFSGKALDEFRHVRQEEVAVLNRAL
ArF3'H2  ASASVAAQFLKHHDSNFSSRPPNSGAKYIAYNYQDLVFAPYGERWRMLRKISSVHLFSGKALDEFRHVRQEEVAVLNRAL

AtF3'H   VRVGTKPVNLGQLVNMCVVNALGREMIGRRLFGADA---DHKADEFRSMVTEMMALAGVFNIGDFVPSLDWLDLQGVAGK
ArF3'H1  ANAGQKPVNLGQLLNVCTVNALGRVMLGRRVFGDGSGGGDAKADEFKSMVVEVMVLAGVFNIGDFIPALEWLDLQGVAAK
ArF3'H2  ANAGQKPVNLGQLLNVCTVNALGRVMLGRRVFGDGSGGGDAKADEFKSMVVEVMVLAGVFNIGDFIPALEWLDLQGVAAK

AtF3'H   MKRLHKRFDAFLSSILKEHEMNGQDQKHTDMLSTLISLKGTDLDGDGGSLTDTEIKALLLNMFTAGTDTSASTVDWAIAE
ArF3'H1  MKKLHNRFDAFLTGIVEEHKKNGGG-KHMDLLSTLISLK-ENADGEGGKLTDTEIKALLLNMFTAGTDTTSSTVEWAIAE
ArF3'H2  MKKLHNRFDAFLTGIVEEHKKNGGG-KHTDLLSTLISLK-ENADGEGGKLTDTEIKALLLNMFTAGTDTTSSTVEWAIAE

AtF3'H   LIRHPDIMVKAQEELDIVVGRDRPVNESDIAQLPYLQAVIKENFRLHPPTPLSLPHIASESCEINGYHIPKGSTLLTNIW
ArF3'H1  LIRHPKILAQVRQELDSVVGQERLVSELDVAQLTYLQAVVKETFRLHPSTPLSLPRMADDDCEIAGYHIPKGSTLLVNVW
ArF3'H2  LIRHPKILAQVRQELDSVVGQERLVSELDVAQLTYLQAVVKETFRLHPSTPLSLPRMAADDCEIDGYRIPKGSTLLVNVW

AtF3'H   AIARDPDQWSDPLAFKPERFLPGGEKSGVDVKGSDFELIPFGAGRRICAGLSLGLRTIQFLTATLVQGFDWELAGGVTPE
ArF3'H1  AIARDPDQWSNPLEFRPERFLPGSEKAGVDVKGNDFEVIPFGAGRRICAGMSLGLRMVHLLTASLVHAFDWDLANGLMPE
ArF3'H2  AIARDPDQWSNPLEFRPERFLPGSEKASVDVKGNDFEVIPFGAGRRICAGMSLGLRMVHLLTASLVHAFDWDLANGLMPE

AtF3'H   KLNMEESYGLTLQRAVPLVVHPKPRLAPNVYGLGSG
ArF3'H1  KLNMDEAYGLTLQRAEPLMVHPRPRLSPHVYRASS-
ArF3'H2  KLNMDEAYGLTLQRAEPLMVHPRPRLSPHVYRASS-
```

Supplementary Figure S33. Protein sequence alignment of *bona fide* flavonoid 3′-hydroxylase from *Arabidopsis thaliana* (AtF3′H (31)) and that of putative homologs (ArF3′H1 and ArF3′H2) in red alder.

```
PhF3'5'H    1 -----MMLLTELGAATSIFLIAHIIISTLISKTTGRHLPPGPRGWPVIGALPLLGAMPHVSLAKMAKKYGAIMYLKVGTC
ArF3'5'H    1 MAALDIFLFRELATAIILFFITLFSVRSLL-GKPSRKLPPGPKGWPIIGALPLLGTMPHVTLANMAKKYGPVMYLKMGTC

PhF3'5'H   76 GMAVASTPDAAKAFLKTLDINFSNRPPNAGATHLAYNAQDMVFAHYGPRWKLLRKLSNLHMLGGKALENWANVRANELGH
ArF3'5'H   80 NMVVASTPDAARAFLKTLDINFSNRPPNAGATHLAYDAQDMVFADYGQRWKLLRKLSNLHMLGGKALDDWAHVRGNELGH

PhF3'5'H  156 MLKSMSDMSREGQRVVVAEMLTFAMANMIGQVMLSKRVFVDKGVEVNEFKDMVVELMTIAGYFNIGDFIPCLAWMDLQGI
ArF3'5'H  160 MLRAMNESSKQGEAVVVPEMLTYAMANMIGQVILSRRVFVTKGEESNEFKDMVVELMTSAGFFNIGDFIPSIAWMDLQGI

PhF3'5'H  236 EKRMKRLHKKFDALLTKMFDEHKATTYERKGKPDFLDVVMENGDNSEGERLSTTNIKALLLNLFTAGTDTSSSAIEWALA
ArF3'5'H  240 EGGMKRLHKRFDVLLTKMIEEHTASAHQRKGKPDFLDVVMANRENSAGEKLTLTNIKALLLNLFTAGTDTSSSIIEWALA

PhF3'5'H 3 16 EMMKNPAILKKAQAEMDQVIGRNRRLLESDIPNLPYLRAICKETFRKHPSTPLNLPRISNEPCIVDGYYIPKNTRLSVNI
ArF3'5'H  320 EMLNNPTILKRAHEEMDKVIGRNRRLEETDIQKLPYLQAICKETMRKHPSTPLNLPRVSTVACEVDGYYIPKNTRLSVNI

PhF3'5'H  396 WAIGRDPQVWENPLEFNPERFLSGRNSKIDPRGNDFELIPFGAGRRICAGTRMGIVMVEYILGTLVHSFDWKLPSEVIEL
ArF3'5'H  400 WAIGRDPDVWENPLDFTPERFLSGRNAKIDPRGNDFELIPFGAGRRICAGTRMGIVLVEFILGTLVHSFDWKLPNG-VEL

PhF3'5'H  476 NMEEAFGLALQKAVPLEAMVTPRLQLDVYVP
ArF3'5'H  479 NMDESFGLALQKAVPLAAIVSPRLSLSAYAS
```

Supplementary Figure S34. Protein sequence alignment of *bona fide* flavonoid 3′,5′-hydroxylase from *Petunia × hybrida* (PhF3′,5′H (32)) and that of a putative homolog (ArF3′,5′H) in red alder.

```
PhFLS       1 --MKTAQGV----SATLTMEVARVQAIASLSKCMDTIPSEYIRSENEQPAATTLHGVVLQVPVIDLRDPDENKMVKLIAD
ArFLS1      1 MGVERVQGIASLDGHKKLMGVERVQGIASLS--KDEIPAEFVRSVNEQPGITTVHGEVLEVPTIDLTDPDEEKVLRAIAD
ArFLS2      1 ------------------MAVESVQALASLSLTTNTIPAEFIRPEKEQPALTTFHGPAPEIPTIDLSDPDQENLVRSIAD

PhFLS      75 ASKEWGIFQLINHGIPDEAIADLQKVGKEFFEHVPQEEKELIAKTPGSNDIEGYGTSLQKEVEGKKGWVDHLFHKIWPPS
ArFLS1     79 ASRNWGMFQIVNHGIPVEAITKLQASGKEFFE-LPQEEKEVYAKPPGPHSMEGYGTKLQKEAEGKKAWVDHLFHKIWPPS
ArFLS2     63 ASKEWGIFQVVNHGIPTDVVSNLQAVGKEFFQ-LSPEEKEAYAKPPGAQSMEGYGNKLQKDLNGKKSWVDHLFHRIWPPS

PhFLS     155 AVNYRYWPKNPPSYREANEEYGKRMREVVDRIFKSLSLGLGLEGHEMIEAAGGDEIVYLLKINYYPPCPRPDLALGVVAH
ArFLS1    158 AVNYRFWPKNPPSYREANEEYAKQLHGVVEKLFKSLSLGLGLEGHELKEAASGDDMVYLLKINYYPPCPRPDLALGVPAH
ArFLS2    142 LINYHFWPKNPPSYREANEVYAKYVREVADKLLTRLSLGLGLQGDALKEAGGGEEMVFLLKINYYPPCPRPDLTLGVVAH

PhFLS     235 TDMSYITILVPNEVQGLQVFKDGHWYDVKYIPNALIVHIGDQVEILSNGKYKSVYHRTTVNKDKTRMSWPVFLEPPSEHE
ArFLS1    238 TDMSVITILVPNHVQGLQASRDGHWYDVKYIPNALVIHVGDQLEILSNGKYKAVLHRTTVNKEQTRMSWPVFLEPPGEFE
ArFLS2    222 TDFSILTLLVPNEVPGLQVLKDDRWIDAKYIPNALIVHIGDQIEILSNGKYKSVLHRSTVDKEKARMSWPVFVEPPAEFV

PhFLS     315 VGPIPKLLSEANPPKFKTKKYKDYVYCKLNKLPQ
ArFLS1    318 VGPHPKLVDHENPPKYKTKKYSDYVYCKLNKIPQ
ArFLS2    302 VGPMPQLVNEENPKKYKARKFKDYAYCKLNKLPQ
```

Supplementary Figure S35. Protein sequence alignment of *bona fide* flavonol synthase from *Petunia × hybrida* (PhFLS (33)) and that of putative homologs (ArFLS1 and ArFLS2) in red alder.

```
AmDFR     1 MSPTSLNTSSETAPPSSTTVCVTGAAGFIGSWLVMRLLERGYTVRATVRDPGNMKKVKHLIELPKADTNLTLWKADMTVE
ArDFR1    1 ------------MGAESETVCVTGAAGFIGSWLVMRLLERGYTVRATVRDPENMKKVKHLVELPKAKTHLTLWKADLADE
ArDFR2    1 ------------MGSESESVCVTGASGFIGSWLVMRLLERNYSVRATVRDPGKYKVEGDQPNVIGKKRQ-----------

AmDFR    81 GSFDEAIQGCEGVFHLATSMEFDSVDPENEVIKPTIDGMLNIIKSCVQAKTVKKFIFTTSGGTVNVEEHQKPVYDETDSS
ArDFR1   69 GSFDEAIKGCSGVFHVATPMDFESQDPENEVIKPTVNGVLSIMKACVEAKTVRRLVFTSSAGTLDVSGHQKPVNDESCWS
ArDFR2   58 GNRE-----REDKIIQLKLFSLCGTAKMNEVIKPTINGLLGIMNACVKAKTVRKLVFTSSAGSVNVEEHQKPVYDESCWS

AmDFR   161 DMDFINSKKMTGWMYFVSKILAEKAGMEAAKENNIDFISIIPPLVVGPFIMPTFPPSLITALSPITGNEAHYSIIKQCQY
ArDFR1  149 DVDFCRTKKMTGWMYFVSKTLAEQAAWKFAKENNLDFISIIPPLVVGPFLMPSMPPSLITALSPITGNEAHYSIIKQGQF
ArDFR2  133 DVEFCRAKKMTGWMYFVSKTLAEQAAWKFAKENGLDFITIIPPLVVGPFIMPSMPPSLITALSLITGNEAHYSIIKQGQF

AmDFR   241 VHLDDLCEGHIFLFEYPKAEGRYICSSHDATIYDIAKLITENWPEYHIPDEFEGIDKDIPVVSFSSKKMIGMGFIFKYTL
ArDFR1  229 VHLDDLCMAHIFLFENPKAEGRYICSACDATILDVAKLLREKYPEYNVPTKFKDVDENLEVVSFTSKKVKDLGFQFKYSL
ArDFR2  213 VHLDDLCIAHIFLYEHPKAEGRYICSADDATIHDIAKLLREKYPEYNIPTKLKGIEEKLDIVSFSSKKVKELGFQFKYSL

AmDFR   321 EDMVRGAIDTCREKGMLPYSTKNNKGDEKEPILNSLENNYNIQDKELFPISEEKHINGQENALLSNTQDKELLPTSEEKR
ArDFR1  309 EDMFVEAVETCRAKGLIPLAAENSVSGTS---------------------------------------------------
ArDFR2  293 EDMFVGAVETCRAKGLLPPAAGN---------------------------------------------------------

AmDFR   401 VNGLESALLSKIQDKEVLPTSGVKHAKGQENALLPDIANDHTDGRI
ArDFR1      ----------------------------------------------
ArDFR2      ----------------------------------------------
```

Supplementary Figure S36. Protein sequence alignment of *bona fide* dihydroflavonol 4-reductase from *Antirrhinum majus* (AmDFR (34)) and that of putative homologs (ArDFR1 and ArDFR2) in red alder.

```
DuLAR      1 MTVSGAIPSMTKNRTLVVGGTGFIGQFITKASLGFGYPTFLLVRPGPVS--PSKAVIIKTFQDKGAKVIYGVINDKECME
ArLAR1     1 MTVTT-SVSTAKGRVLIAGATGFIGQFVAEASLDAGRPTYILVRPGPVGGGPSKASTIRAFQDKGATILHGLINDKEFME
ArLAR2     1 MCGSGPNVSEAGGRAMIIGSTGFIGQFVAEASLDSGRPTYLLVQSSQAS--PSKASTIKSLQDKGAIIIYGSIEDRDLLE

DuLAR     79 KILKEYEIDVVISLVGGARLLDQLTLLEAIKSVKTIKRFLPSEFGHDVDRTDPVEPGLTMYKEKRLVRRAVEEYGIPFTN
ArLAR1    80 KILKEHEIDIVISAVGGGNILDQLTLIEAIKSVGTIKRFLPSEFGHEVDRADPVEPGLDMYQAKRKVRRLIEDYGVPHTY
ArLAR2    79 KILKEHKIEVVISAIGGDRISDQLILVDAIKAVGTVKRFLPSEFGHDIDRADPVEPALTFYNSKRIVRRYIEAAGIAYTY

DuLAR    159 ICCNSIASWPYYDNCHPSQVPPPMDQFQIYGDGNTKAYFIDGNDIGKFTMKTIDDIRTLNKNVHFRPSSNCYSINELASL
ArLAR1   160 ICCNSIASWPYYDNTHPSEVVPPLDQFQIYGDGNVKAYFVAGADIGKLTIKTIDDIRAVNKCVHFRPAGNLYTINELASL
ArLAR2   159 ICCNSIAAWPYHDNTHPADVLPPLDRFYIYGDGTVKAYFVAGADIGKFTMKSIDDDRTLNKCVHFRPSSNLLNVNELASL

DuLAR    239 WEKKIGRTLPRFTVTADKLLAHAAENIIPESIVSSFTHDIFINGCQVNFSIDEHSDVEIDTLYPDEKFRSLDDCYEDFVP
ArLAR1   240 WEKKIGRTLPRVTVSENDLLAAAAENRIPQSIVASFTHDIFIKGCQVNFTIDGPQDVEVGALYPDETFRTLDECFNDFLV
ArLAR2   239 WEKKIKRELPRVSISEDDLLAAAKDMRIPESIVAALTHDIFIKGCQINYAMDKPNDVDVCSLYPDTPFRTIDDCFNDLIK

DuLAR    319 MVHDKIHAGKSGE----IKIKDGKP--LVQTGTIEEINKDIKTLVETQPNEEIKKDMKALVEAVPISAMG
ArLAR1   320 NIDSKSKSTDEIT----TASPVAEP--MAVTAAYA-----------------------------------
ArLAR2   319 NSVGAPKVVEEPISNDAIIAPNSKPEALVITA--------------------------------------
```

Supplementary Figure S37. Protein sequence alignment of *bona fide* leucoanthocyanidin reductase from *Desmodium uncinatum* (DuLAR (35)) and that of putative homologs (ArLAR1 and ArLAR2) in red alder.

```
AtLDOX     1 -----MVAVERVESLAKSGIISIPKEYIRPKEELESINDVFLEEKKEDGPQVPTIDLKNIESDDEKIRENCIEELKKASL
ArLDOX     1 MVTSNSSMAPRVESLSSSGIQAIPKEYVRPQEELNSIGNIFEEERKEEGPQVPTIDLKNIESDDEAVREKCREELKRAAV

AtLDOX    76 DWGVMHLINHGIPADLMERVKKAGEEFFSLSVEEKEKYANDQATGKIQGYGSKLANNASGQLEWEDYFFHLAYPEEKRDL
ArLDOX    81 EWGVMHIVNHGIPDELLERVKEAGKVFFDLPVEEKEKYANDQALGKIQGYGSKLANNASGQLEWEDYFFHLAYPEDKRDM

AtLDOX   156 SIWPKTPSDYIEATSEYAKCLRLLATKVFKALSVGLGLEPDRLEKEVGGLEELLLQMKINYYPKCPQPELALGVEAHTDV
ArLDOX   161 SIWPKTPTDYIAATSEYARQLRKLASKVLSVLSLGLGLEEGRLEKEVGGLEELLLQMKINYYPICPQPELALGVEAHTDI

AtLDOX   236 SALTFILHNMVPGLQLFYEGKWVTAKCVPDSIVMHIGDTLEILSNGKYKSILHRGLVNKEKVRISWAVFCEPPKDKIVLK
ArLDOX   241 SALTFILHNMVPGLQLFYQGKWVTAKCVPNSIIMHIGDTLEILSNRKYKSILHRGLVNKEKVRISWAVFCEPPKEKIILK

AtLDOX   316 PLPEMVSVESPAKFPPRTFAQHIEHKLFGKEQEELVSEKND
ArLDOX   321 PLPEVVSEEEPAIFPPRTFAQHIQHKLFRKTQDALDAK---
```

Supplementary Figure S38. Protein sequence alignment of *bona fide* leucoanthocyanidin dioxygenase [also called anthocyanidin synthase (ANS)] from *Arabidopsis thaliana* (AtLDOX (36)) and that of a putative homolog (ArLDOX) in red alder.

```
MtANR     1 MASIKQIEIEKKKACVIGGTGFVASLLIKQLLEKGYAVNTTVRDLDSANKTSHLIALQSLGELNLFKAELTIEEDFDAPI
ArANR1    1 ---MATQHIGKKSVCVVGGTGFVASLLVKLLLEKGYAVNTTVRDPDNQKKISHLIALQDLGDLKIFGADLTDEGSFDAPI
ArANR2    1 ---MATQHIGKKSACVVGGTGFVASLLVKLLLEKGYAVNTTVRDPDNQKKISHLIALQDLGDLKIFGADLTDEGSFDAPI

MtANR    81 SGCELVFQLATPVNFASQDPENDMIKPAIKGVLNVLKACVRAKEVKRVILTSSAAAVTINELEGTGHVMDETNWSDVEFL
ArANR1   78 AGCDFVFHLATPVNFASEDPENDMIKPAIQGVHNVLKASARAKTVKRVVLTSSAAAVTINKLNGTGLIIDESHWTDVEFL
ArANR2   78 AGCDFVFHLATPVNFASEDPENDMIKPAIQGVHNVLKASARAKTVKRVVLTSSAAAVTINKLNGTGLIIDESHWTDVEFL

MtANR   161 NTAKPPTWGYPVSKVLAEKAAWKFAEENNIDLITVIPTLTIGPSLTQDIPSSVAMGMSLLTGNDFLINALKGMQFLSGSI
ArANR1  158 STEKPPTWGYPASKTLAEKAAWKFAEENNIDLITVIPTLMAGASLTPDVPSSVGLAMSLITGNEFLINGIKGMQMLSGSI
ArANR2  158 STEKPPTWGYPASKTLAEKAAWKFAEENNIDLITVIPTLMAGASLTPDVPSSVGLAMSLITGNEFLINGIKGMQMLSGSI

MtANR   241 SITHVEDICRAHIFVAEKESTSGRYICCAHNTSVPELAKFLSKRYPQYKVPTEFDDFPSKAKLIISSGKLIKEGFSFKHS
ArANR1  238 SITHVEDVCRAQILLAEKESTSGRYICCGVNTSVPELAKFLNKRYPQYKVPTE---------------------------
ArANR2  238 SITHVEDVCRAQILLAEKESASGRYICCGVNTSVPELAKFLNKRYPQYKVPTDFGDFPSKAKLALSSDKLIKEGFSFKYG

MtANR   321 IAETFDQTVEYLKTQGIK--
ArANR1      --------------------
ArANR2  318 IEAIYDQTVEYFKAKGLLQN
```

Supplementary Figure S39. Protein sequence alignment of *bona fide* anthocyanidin reductase from *Medicago truncatula* (MtANR (37)) and that of putative homologs (ArANR1 and ArANR2) in red alder.

```
GhFSNII       1 -MNTLQLIFLLFFFP-TLLFLYCLPYKRNQNHRRLPPSPPSFPIIGHLHHLGPLIHQSFHALSTRYGSLIHLRLGSVPCV
ArFNSII-1     1 --MAIELLLV---LT-LLVGLLVSTILFSAFHSKLPPSPIALPIIGHFHLLGPIIHQSLHKLSLRYGPLFSLRLGSVPCI
ArFNSII-2     1 MPHDLQGYIILFLIWLVSIILVRAIFHRIKTKVRLPPSPMALPIIGHLHHLTSLPHQALHKLSNRHGPLIHIFLGSVPCV
ArFNSII-3     1 ---------------------------------------MALPIIGHLHHLAPLPHQALHKLSNRHGPLIHIFLGSVPCV
ArFNSII-4     1 -MPDFQYYLLLLLVWLATI-LVRNFFLTKTKKARLPPSPRALPIIGHLHLLSRIPHQALHKLSNQHGPLIYLLFGSKPCV

GhFSNII      79 VVSTPDLAKDFLKTNELAFSSRKHSLAIDHITYGV-AFAFAPYGTYWKFIKKLFTVELLGTQNLSHFLPIRTHEIRELLR
ArFNSII-1    75 VASTPEYAKELLKTNELSFTSRKHTIAIDRLTYNS-SFAFAPSGPYWIFMKKLSANELLGSRTVNAFISLREKEYRRLLR
ArFNSII-2    81 VASSPEMAKEFLKTHEASFSNRPHIAAFDYLTYGSANFSFAPYGPYWKFMKKLCMSELLSGRKLDQLLPVRREEINRFVQ
ArFNSII-3    42 VASSPEMAKEFLKTHETSFSNRPHTAAVDYLTYGYADFSFAPYGPYWKFMKKLCMSELLGGRTLDQLLLVRREEINKFMQ
ArFNSII-4    79 IVSSPEMARECLKTQETRFLNRPKMTNIDYLTYGSADFVLAPYGQYWKFMKKLCMTELLGGRTLEQNISVRGEEIKQFLQ

GhFSNII     158 TLMVKSRAKERVNLTEELLKLTNNVISQMMMSIRCSGTNSEADEAKNLVREVTKIFGQFNVSDFIWFCKNIDLQGFKKRY
ArFNSII-1   154 VLADKSEAGEAVNVTEELLKLTNNIISQMMLGRAE--------EARAV-----------------------IREVGFGKRI
ArFNSII-2   161 FILQKAEAGEAVDVGGQLMRVTNNVISRMVMSVRCSENEHEADEVAKVVKATAELSGKFNLSDFIWFCKNLDLQGFGKKL
ArFNSII-3   122 FILQKAEAGEAVDVGGQLMRVTNNVISRMAMSVRCSKNEHEADEMAK---------------------------LGFGKRV
ArFNSII-4   159 LLLDKANANEAVDVGAELIRLTNNIISRMALRTRCSEKEDEAEEVGKLVKEMCELAGKANVSEMIWFCKNLDLQGFGKRL

GhFSNII     238 EGTHRRYDALLERIIMGREENRR-------RGKIKDGEGKDFLDMLLDVLEDGKAEIKITRDHIKALILDFLTAGTDTTA
ArFNSII-1   204 EDIHRRFDALVEKIITERKDLWKKKENGERERDGREHQVRDFMDILLDFSEDENSEIKLTRAHIKALIVDFFAAGTDTTA
ArFNSII-2   241 KEVRGNFDTMMERIIKEHEEARKKEKAKDMDG---GYAVKDLLHILLDIYEDKSSEMRLTRENIKAFILDIFGAGTDTSA
ArFNSII-3   176 KEVRGNFDSMMERIIKEHEEARKKE--KDMDG---GYAVKDLLHILLDIYEDKSSKMRLTRENIKAFILDIFAAGTDTSA
ArFNSII-4   239 KDVRGRYDAMMERIMKEHEEARKKKK-EMGDG---GDGIKDVLDILLDIYEDESSEIRMTRENIKGFIMNMFGAGTDTSA

GhFSNII     311 IAIEWALVELINNPNALEKARQEIDQVIGDERLVQESDTPNLPYIQAIIKEALRLHPPIPMLIRKSTENVIVQGYDIPAG
ArFNSII-1   284 IAIEWALAELINHPMVLEKARKEIDNVVKNSRLVQESDVPNLPYIYAIIKETLRLHPPLPMVARKCIEDRRIGSYVIPAN
ArFNSII-2   318 ITTEWALAELINHPIVMNKARQEIDYVVGKSRLVEESDITNLPYLQAIVKETLRLHPTGPVIVRESSEHCTVGGYEIPAK
ArFNSII-3   251 ITTEWALAELINHPSVMNRARQEIDSEVGKSRLVEESDITNLPYLQAIVKETLRLHPTGPIIVRESSEHCTVGGYEIPAK
ArFNSII-4   315 VTTEWAISELINHPHVMAKARKEIDIVIGKKRVVEESDIANLPYIQAIVKETLRLHPTGPLVVRECTEDCMVAGYEIPAK

GhFSNII     391 TLLFVNIWSIGRNPQCWETPLEFKPHRFLDGG----DLKSSLDIKGHNFQLLPFGTGRRGCPGVNLAMRELSVVIANLIQ
ArFNSII-1   364 SLLFVNVWAIGRDSNYWKNPLDFEPERFLPKE----GM-GMIDVRGQQFQFLPFGSGRRSCPGINLAMQELPALLAAMIQ
ArFNSII-2   398 TRLFVNVWAIGRDPKYWENPLEFRPERFNISESEGSTGKSQLDVRGQHFHLLPFGSGRRGCPGASLALQVVPTTLATMIQ
ArFNSII-3   331 TRLFVNVWAIGRDPKHWENPLEFRPERFIISESEE-SGKSQLDVRGQHFHLLPFGSGRRGCPGTSLALQVVQTTLATMIQ
ArFNSII-4   395 TRLFVNVWAIGRDPNHWENPLEFWPERFLDKE---GDGKSQLDVRGQHFHLLPFGSGRRSCPGATLAMQVVQTTLASMVQ

GhFSNII     467 CFDWDVVGER---------LLNTDERAGLTAPRAVDFVCVPLERGNTLKILGSN----------
ArFNSII-1   439 CFDFKLVGQQGGIMDGENVVVNMDEGPGLTAPRAHDLVCVPMARLSSLDILHP-----------
ArFNSII-2   478 CFEWKVVGED-G-------AVNMEEGPGLTLPRAHPLICVPAARLHPFPSIS------------
ArFNSII-3   410 CFEWKVVGGD-G-------AVDMEEGPGLTLPRAHPLICVPRLGSIHSPQPHNHKDCSLLCYCN
ArFNSII-4   472 CFEWKVGDGDQY-------SVDMEEGPGISLPRAHPLVCIPVARLTPFPSIN------------
```

Supplementary Figure S40. Protein sequence alignment of *bona fide* synthase II from *Gerbera* hybrid cultivar (GhFSNII (38)) and that of putative homologs (ArFNSII-1 to ArFNSII-4) in red alder.

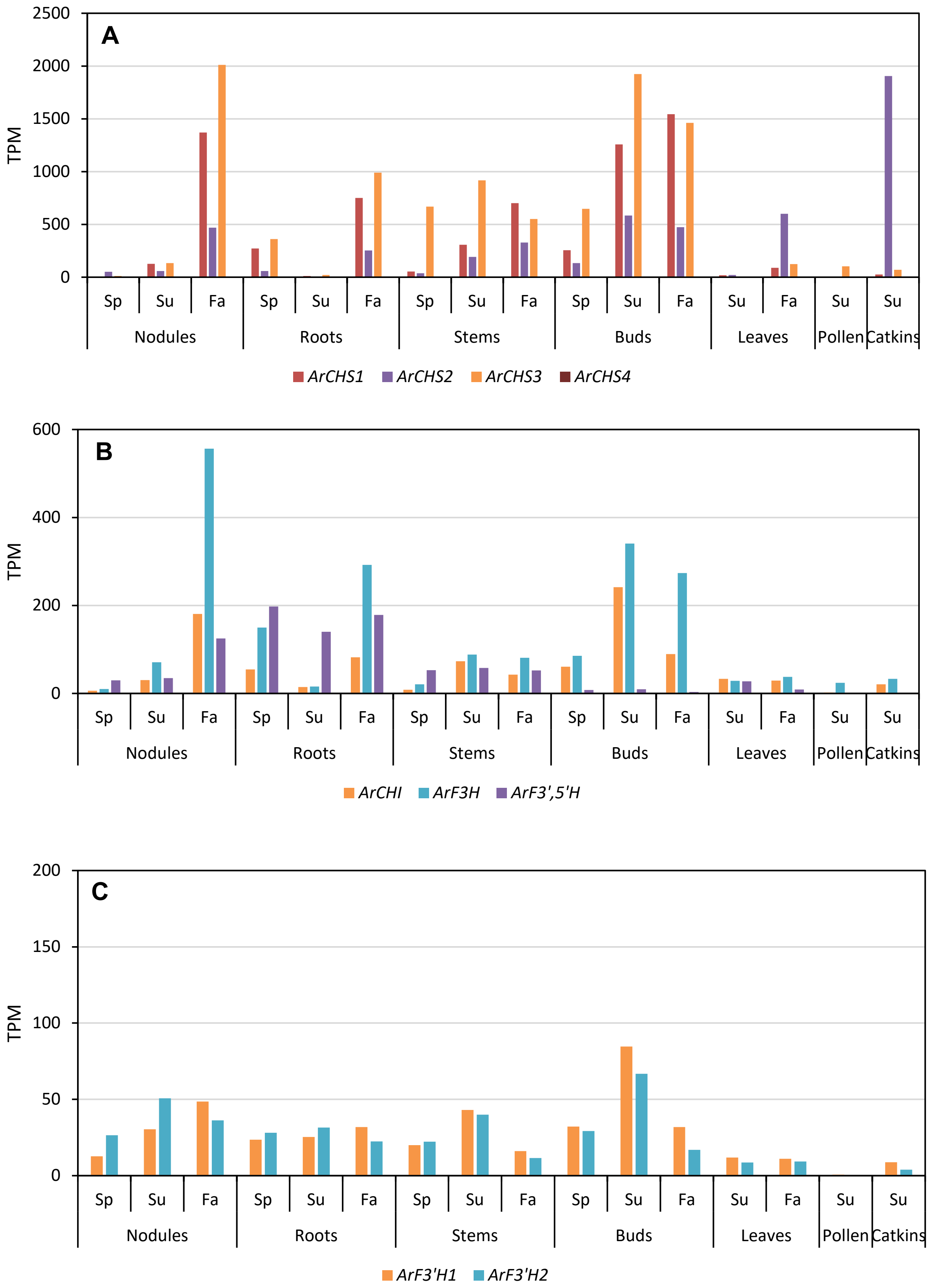
A
TPM
2500
2000
1500
1000
500
0
Sp Su Fa Sp Su Fa Sp Su Fa Sp Su Fa Su Fa Su Su
Nodules Roots Stems Buds Leaves Pollen Catkins
ArCHS1 ArCHS2 ArCHS3 ArCHS4
B
TPM
600
400
200
0
Sp Su Fa Sp Su Fa Sp Su Fa Sp Su Fa Su Fa Su Su
Nodules Roots Stems Buds Leaves Pollen Catkins
ArCHI ArF3H ArF3',5'H
C
TPM
200
150
100
50
0
Sp Su Fa Sp Su Fa Sp Su Fa Sp Su Fa Su Fa Su Su
Nodules Roots Stems Buds Leaves Pollen Catkins
ArF3'H1 ArF3'H2

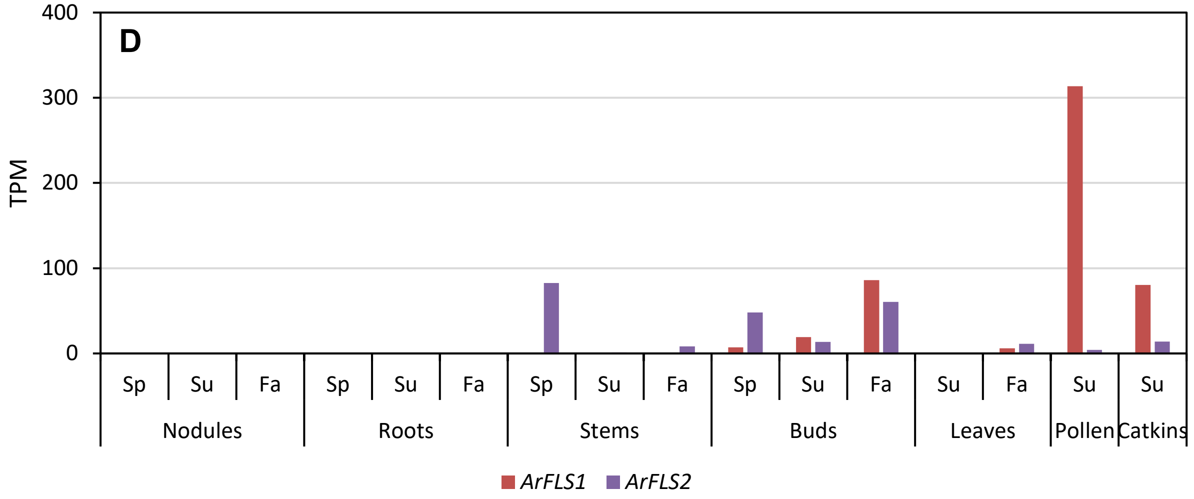
D
TPM
400
300
200
100
0
Sp Su Fa Sp Su Fa Sp Su Fa Sp Su Fa Su Fa Su Su
Nodules Roots Stems Buds Leaves Pollen Catkins
ArFLS1 ArFLS2

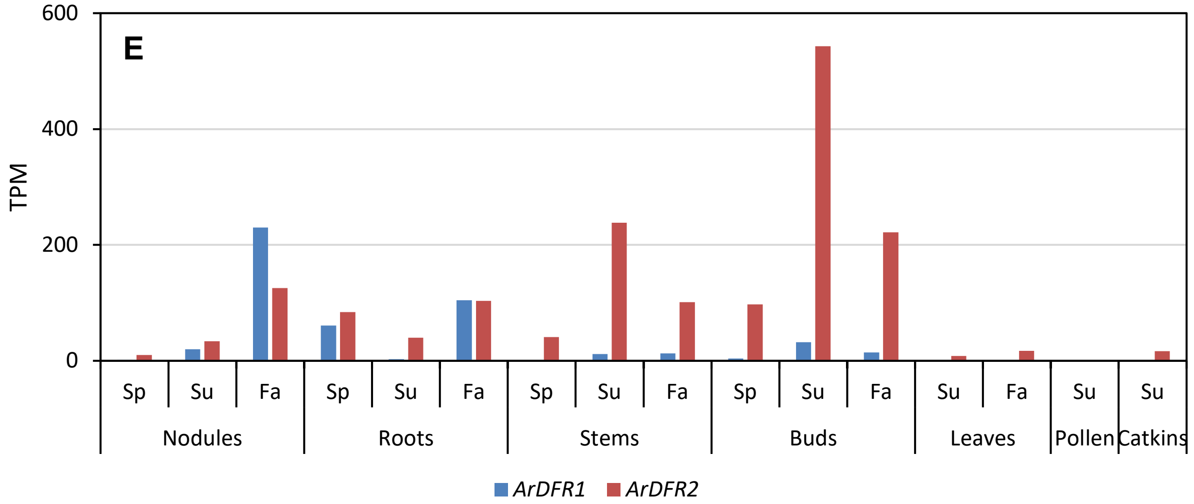
E
TPM
600
400
200
0
Sp Su Fa Sp Su Fa Sp Su Fa Sp Su Fa Su Fa Su Su
Nodules Roots Stems Buds Leaves Pollen Catkins
ArDFR1 ArDFR2

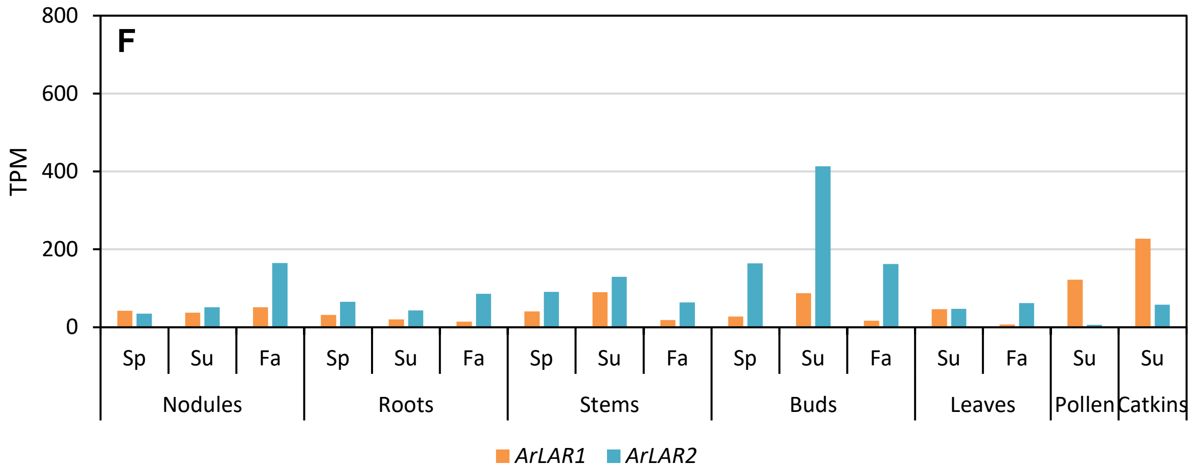
F
TPM
800
600
400
200
0
Sp Su Fa Sp Su Fa Sp Su Fa Sp Su Fa Su Fa Su Su
Nodules Roots Stems Buds Leaves Pollen Catkins
ArLAR1 ArLAR2

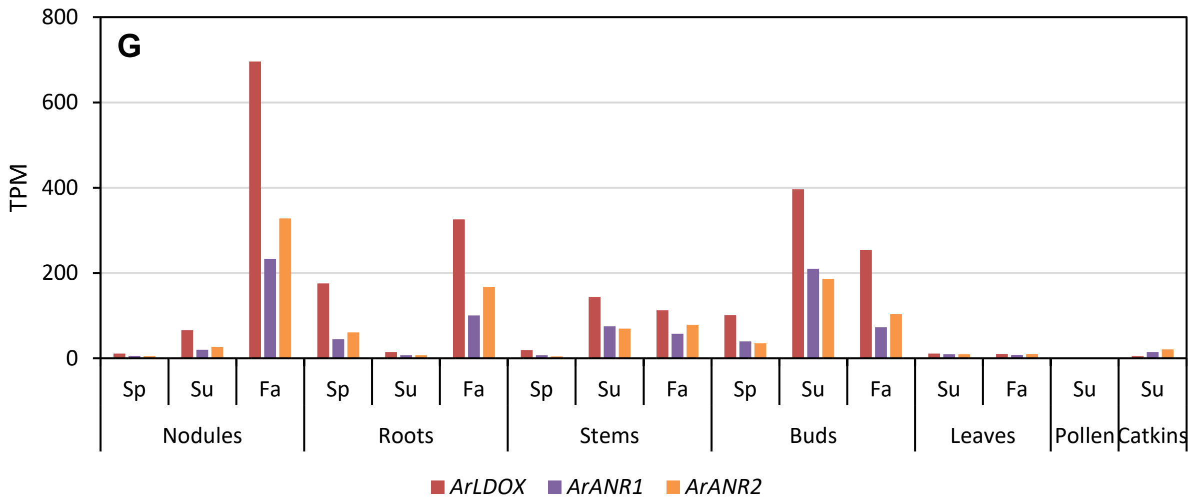

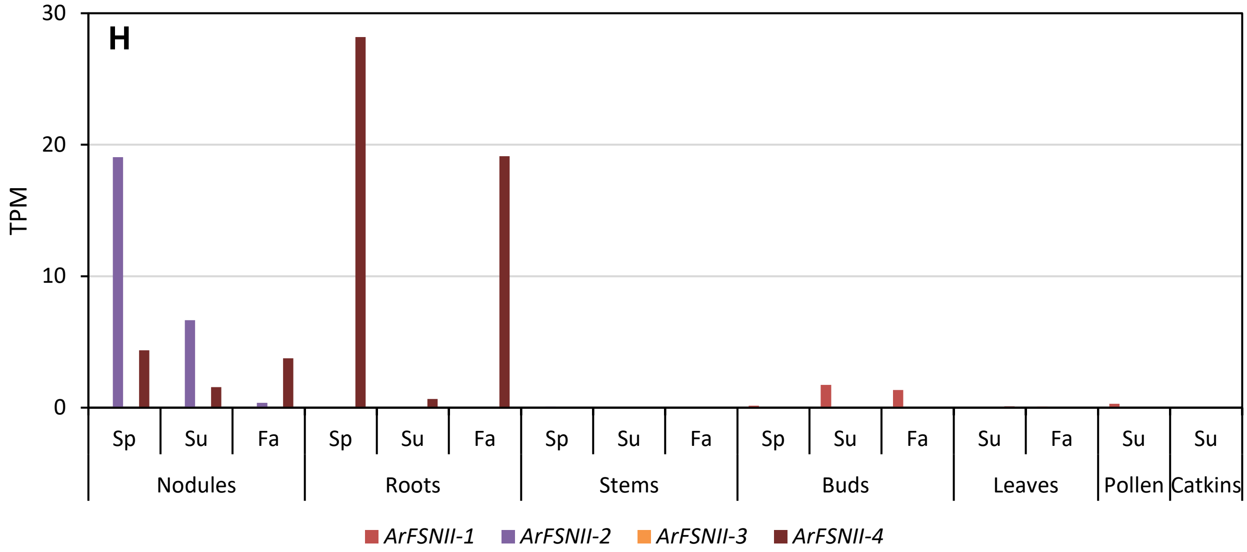

Supplementary Figure S41. Expression levels of red alder homologs of *bona fide* flavonoid pathway genes. **(A)** Chalcone synthase (*ArCHS*). **(B)** Chalcone isomerase (*ArCHI*), flavanone 3-hydroxylase (*ArF3H*), flavonoid 3′,5′-hydroxylase (*ArF3′,5′H*). **(C)** Flavonoid 3′-hydroxylase (*ArF3′H*). **(D)** Flavonol synthase (*ArFLS*). **(E)** Dihydroflavonol 4-reductase (*ArDFR*). **(F)** Leucoanthocyanidin reductase (*ArLAR*). **(G)** Leucoanthocyanidin dioxygenase (*ArLDOX*) and anthocyanidin reductase (ANR). **(H)** Flavone synthase II (*ArFNSII*). Abbreviations: TPM, transcripts per million; Sp, spring; Su, summer; Fa, fall.

```
DRR206      1 --MG---SKLL--VLFVFVML-FAL-----SSAIPNKRKPYKPCKNLVFYFHDILYNGKNAANATSAIVAAPEGVSLTKL
ArDIR1      1 --MG---GRYYYVVVFCFLTL-LAV-----SSAF-QGKKQYKPCKRLSLYFHDIIYNGENAANATSAIVAAPEGANLTIL
ArDIR2      1 --ME---SNIQILAFFLFFLL-LGSMAAPAQKAKDQKAKDRRPCRRLVFYFHDIVYNGKNLKNATSAIVGAPAWANTTTM
AtDIR6      1 --MAFLVEKQLFKALFSFFLLVLLFSDTVLSF--RKTIDQKKPCKHFSFYFHDILYDGDNVANATSAAIVSPPG------
ArDIR3      1 MAMGELVQ----KSCFLLFLMILIC-QSVLASQKYYSLRHRKPCKRLELYYHDILFNGTDVANATSAKATNQTA------

DRR206     68 APQSHFGNIIVFDDPITLSHSLSSKQVGRAQGFYIYDTKNTYTSWLSFTFVLNSTHHQGTITFAGADPIVAKTRDISVTG
ArDIR1     69 AGQFHFGNIAVFDDPITLDNNLHSKPVGRAQGLYIYDTKNTFTAWLGFTFVLNSTDHQGTINFNGADPILAKTRDISIVG
ArDIR2     75 AGQNHFGDLVVFDDPITLDNNLHSTPVGRAQGFYVYDKKDIFTAWLGFSFVFNSTQHKGSINFAGADPLMNKTRDISVVG
AtDIR6     71 LGNFKFGKFVIFDGPITMDKNYLSKPVARAQGFYFYDMKMDFNSWFSYTLVFNSTEHKGTLNIMGADLMMEPTRDLSVVG
ArDIR3     70 LGNFHFGMLVVFDDPITEDSHLRSPPIARAQGFYFYNKKDDYNAWFSFTLVFNSSQHKGTINIMGADLMAEETRDLSVVG

DRR206    148 GTGDFFMHRGIATITTDAFEGEAYFRLGVYIKFFECW
ArDIR1    149 GTGDFFMHRGIATIMTDAFEGEVYFRLHVDIKFYECW
ArDIR2    155 GTGDFFMARGIATLMTDAFEGEVYFRLRVDIQLYECW
AtDIR6    151 GTGDFFMARGIATFVTDLFQGAKYFRVKMDIKLYECY
ArDIR3    150 GTGDFFMTRGIATIRTDTFQGSYYFRLKMDIKLYECY
```

Supplementary Figure S42. Protein sequence alignment of *bona fide* dirigent proteins from the Dir-a sub-family, DRR206 from *Pisum sativum* (39) and AtDIR6 from *A. thaliana* (60) and that of putative homologs (ArDIR1 – ArDIR3) in red alder.

```
PsPTS1     1 ------------------MAK-SSS-----FFITFF-----ISLISFATATKYYQSLSPTMLGF--QEEKFTHLHFYFHD
ArDIR4     1 ------------------MAK-SLSKLTPILFIFL--FSTYVT----AETHNFSRNLSPSSLGL--KREKLSHLHFYFHD
ArDIR5     1 ------------------MAK-TLPKVTPTVLILFTIFSFSAF----TTAGSFSRALSPAKLGL--KREKLSHLHFYFHD
ArDIR6     1 ------------------MAK-TLPKVTPTFLILFTILSFATF----ATAGGFSRTLSPAKLGL--KREKLSHLHFYFHD
ArDIR7     1 ------------------MAK-TLPKVIPTFLILFSIFSFSAF----ATAGGFSRTLSPVKFGL--KREKLSHLHFYFHD
ArDIR8     1 ------------------MAK-ILPILAFQLLLFFFISSFSTVSVAGDQDEGFSRSLDRKLLGL--KKEKLSHFRFYWHD
ArDIR9     1 -----MQALYSIIVP---MGR-ILPILASQLLLFFFISSYSTVSVTGE-DEGFSRSLDRKLLGL--KKEKLSHFRFYWHD
ArDIR10    1 ------------------MAR-ILPILASQLLLFFFISSFSTVSVAGE-DGGFSRSLDRKLLGL--KKEKLSHFRFYWHD
ArDIR11    1 ------------------MAR-ILPILASQLLLFFFISSFSTVSVAGE-DGGFSRSLDRKLLGL--KKEKLSHFRFYWHD
ArDIR12    1 ------------------MAR-ILPA----FIVFFLLHYFSATLV-YGEDKGFGTPLDPKLHKV--KKEKLSHFNFFFHD
ArDIR13    1 ------------------MAR-ILPIFPSKFIIFSLLSFLAIILVTG-ENHGFVRNLDRRLIGQIKKKEKLSHIHFYWHD
ArDIR15    1 ------------------MAR-ILPA----FIVFFLLCSFAATSVSAREEQGFGRPLDRKKHKL--KKEKLSHFNFFFHD
ArDIR21    1 MYSNPMQALYSIIVP---MAK-ILPILAFQLLLFFFISSFSTVSVAGDQDEGFSRSLDRKLLGL--KKEKLSHFRFYWHD

PsPTS1    50 IVTGPKPSMVFVAEPNGKVENALPFGTVVAMDDPLTAGPERDSKLVGKAQGIYTSISQEEMGLMMVMTMAFSDGEFNGST
ArDIR4    54 IVSGPNPTAVRVAEAATTNTSATGFGAVVMMDDPLTLEPESSSKLVGRAQGIYALASQHEAGLLMVLNFAFIEGKYSGST
ArDIR5    56 IVSGRNPTAVRVAEAAMTNMSLTGFGAVFMMDDPLTVRPELSSKLVGRAQGIYASASQTEVGFLMVLNFAFMEGKYNGSS
ArDIR6    56 IVSGPNQTAVRVAEAPMTNTSLTGFGAVVMIDDPLTARPELTSKHVGRAQGIYASASQTEVGFLMVLNFAFMEGKYNGSS
ArDIR7    56 IVSGRNPTAVRVAEAPMTNTSLTGFGAVVMMDDPLTTRPELTSKHVGRAQGIYASASQTELGLLMVLNFAFMEGKYNGSS
ArDIR8    60 IVSGRNPSAVLVVP-PASNT-STGFGLVSMIDDPLTLGPKLSSKLVGRAQGFYASASQQELGLLMVMNFAFIEGKYNGST
ArDIR9    69 IVSGRNPSAVMVVP-PASNT-STGFGLVSMIDDPLTLGPKLSSKMVGRAQGFYASASQQELGLLMVMNFAFIEGKYNGST
ArDIR10   59 TISGRNPSAVMVVP-PVSNT-STGFGLVNMIDNPLTLGPNLSSKLVGRAQGFYASASQQELGLLMVMNFAFTEGKYNGST
ArDIR11   59 TISGRNPSAVMVVP-PVSNT-STGFGLVNMIDNPLTLGPNLSSKLVGRAQGFYASASQQELGLLMVMNFAFIEGKYNGST
ArDIR12   55 ILSGRNPTAVQVVP-PPSNTSKTGFGSIAMFDDPLTLGPELSSKTIGKAQGLYGSASQEEFALLMVMNFAFTEGKYNGST
ArDIR13   61 TITGSNPSVAQIVP-PVSNASATSFGAINIIDDPLTLGPKLSSKLIGRAQGFYALTSQEEVGLLMAMNFAFIEGKYNGSV
ArDIR15   56 IQSGRNPTAVQIVP-PPSNTSKTFFGFVNMFDNPLTLGPELSSKTVGKAQGFYGSASQEEFALLMVMNFAFIEGKYNGST
ArDIR21   75 IVSGRNPSAVLVVP-PASNT-STGFGLVSMIDDPLTLGPKLSSKMVGRAQGFYASASQQELGLLMVMNFAFIEGKYNGST

PsPTS1   130 LSILGRNMIMSETIREMAIVGGTGAFRFVRGYAQAKFFSVDFTTGDATVEYDIFVFHY
ArDIR4   134 LSVLGRNSV-FSAVREMPIVGGSGIFRFARGYAQARTHTFDLKTGDAVVEYNVYAFHY
ArDIR5   136 LSVLGRNVI-LSAVRELPIVGGSGLFRFARGYAQARTHTFDMRTKDAVVEYNVYAFHY
ArDIR6   136 LSVLGRNTV-LSAVRELPIVGGSGLFRFARGYAHARTHTFDIGSGDAVVEYNVYVFHY
ArDIR7   136 LSVLGRNTV-LSAVRELPIVGGSGLFRFARGYAHARTHTLDIETGDAVVEYNVYVFHY
ArDIR8   138 ISVLGRNAV-FSAVREMPVIGGSGLFRFARGYVQASTHSFDIKTGDATVEYNVYVLHY
ArDIR9   147 ISVLGRNAV-FSAVREMPVIGGSGLFRFARGYVQASTHSLDIKTGDATVEYNVYVLHY
ArDIR10  137 ISVLGRNAV-FSALREMPVIGGSGLFRFARGYVQASTHSFDIKTKDAIVEYNVYVLHY
ArDIR11  137 ISVLGRNAV-FSALREMPVIGGSGLFRFARGYVQASTHSFDIKTKDAIVEYNVYVLHY
ArDIR12  134 LSIFGRNPGATSKVREFPVIGGSGLFRFARGYALATTHELNMTTGDAIIEYNLYVLHY
ArDIR13  140 ITVLGRNPV-FSKVREMPVIGGSGLFRFARGYVQLSTNKFNSSTGDAIVEYNVYVLHY
ArDIR15  135 LSILGRNPGATSRVREFPVIGGSGLFRFARGYALATTHELNMKTGDAIIEYNVYVFHY
ArDIR21  153 ISVLGRNAV-FSAVREMPVIGGSGLFRFARGYVQASTHSLDIKTGDATVEYNVYVLHY
```

Supplementary Figure S43. Protein sequence alignment of *bona fide* dirigent proteins from the Dir-b sub-family, PsPTS1 from *Pisum sativum* (40) and that of putative homologs (ArDIR4 – 13, 15 and 21) in red alder.

```
GhDIR4     1 --------------------------MRGTSVLSW-----ILILCVCQA-------------AVRSQYYSDTLPYQPRPV
ArDIR14    1 ---MHSERRAR-----EFSKELSIMKLMRVNFVS----FCALLLCMVSIALL-------------RQYCTMPLLPYHMLE
ArDIR16    1 ------------------------M--HMAKVAP-----VLLLFSIAMAIQLEKQSTVAEPNEEVDEWL--Q-NLQTSKQ
ArDIR17    1 ----------------------------MACFVTFITLYSFLIFS-AS------LNTISG--AFVEEFS-EA-MAIKREE
ArDIR20    1 ------------------------M--QMAKLIGV----VLMLFSLVAAIPWAQS-------SEEESWATSQ-EFQKKET
ArDIR22    1 ---MHQPGYSTNRAESTLLETIPAE--LMDSFVTF------LFFSASL------INTIDG--AFIEKFS-EP-RAVKREQ
ArDIR25    1 ----------------------------MASFLSLFY-FCLILF-STS------STTVFG--VFSEESL-IT-MYAKRME
ArDIR27    1 MFLMHSERRAR-----EFSKELSIMKLMRVNFVS----FCALLLCMVTIALL-------------RQYCTMPLLPYHMLE

GhDIR4    37 LVTNLHFYMHEFTGTT---AVVLTQAN-ITSNNSSVPFATLVAVNDPLRTGPEPDSELIGNVQGISLLAGSNA-SSTQYI
ArDIR14   56 KETVLHFFLHDTPGGSSPSAVRVVQANGTVHNTSLTPFGTVWVVDDPLTEGPDPKSTILGNSRGVYVSAGQDELMLVAYL
ArDIR16   47 TISRLNFYFHDTLSGKQPSAVQVAQASNT--DKSPTLFGAVYIFDDPLTEGPEPTSRLVGRAQGLYGSACQQELSLLVAM
ArDIR17   42 KTTHLHFFFHDTISGKHPSAVRIAGPT----ESTFVNFGNTMMIDDALTEGPEPTSKLVGRAQGFYSMAAQDDLALLMVM
ArDIR20   43 VITNLNFYFHDIVGGKNPTAVRVAQAANT--NKSPTSFGVVMMADDPLTKTPDPNSLLVGRAQGLYGSAGQQELALIMAL
ArDIR22   60 --SHLHFFFHDILGGEHPTAVFIAGPP----KSTILDFGNTMMIDNALTEGPDPTSKLVGRAQGLYSKADQNDVALLMVV
ArDIR25   41 KTTRLHFYFHDVLDGTNPTAIFIVTPQ----NLSVGGFGATYMIDDLLTEGLEPTSKPVGRAQGMYAVASQHDLGLLMVI
ArDIR27   62 KETVLHFFLHDTPGGSSPSAVFVVQANGTAHNTSLTPFGTVWVVDDPLTEGPDPKSTILGNSRGVYVSAGQDELMLVAYL

GhDIR4   113 EFGFNTGKFNGSSLSVFSRGD-----PGLAVVGGRGQFAMATGTALFNPILINA--TNVIMEFNFTVIHY----------
ArDIR14  137 DFGFTSGEFNGSSLSVLSRNTVTQIEREFAVVGGRGKFRMARGFANLKCIFFNESNGDSIVEYTMVVRHY----------
ArDIR16  126 TVVFTAGKFNASSLTILGRNAALQEVREMPIIGGSGVFRLARGFAIAKTHSLNFTSGDAIVEYNVVAVHY----------
ArDIR17  119 NFAFVEDKYNGSSISMLGRNPVLNDVREMPIVGGSGLFRNSRGYALAHTNWMDPNTGDAIVEYDVYVSNFQVDAG-LATA
ArDIR20  122 NFGFVDGIYNGSSISLLGKNPASHPVREMAIVGGTGIFRLARGYAIAHTHWFNATSGDAIVRYNVTVVH-----------
ArDIR22  135 NFVFTEDKYKGSSISMIGRNPVANDVREMPIVGGSGLFRNVRGYALAHTNSMD-NAGDAIVEYNVYVSNVEVVDASLATQ
ArDIR25  118 NFAFLEGIYNGSALSILGRNPVFHAVREMPIVGGSGVFRLARGYALAKTVRFNKKSGDAVVEYNVSVLHF----------
ArDIR27  143 DFGFTSGEFNGSSLSVLSRNTVTQIEREFAVVGGRGKFRMARGFANLKCIFFNESNGDSIVEYTMVVRHY----------
```

Supplementary Figure S44. Protein sequence alignment of *bona fide* dirigent proteins from the Dir-d sub-family, GhDIR4 from *Gossypium hirsutum* (41) and that of putative homologs (ArDIR14, 16, 17, 20, 22, 25 and 27) in red alder.

```
AtDIR10    1 --------MAGQKILSLLVIALVVTFAAAARLLDEENAFSATTTTLGSGSGSTGIGFGAGTGSSGSGSTGFGFGAGSGSS
ArDIR18    1 --------------------------------------------------------------------------------
ArDIR24    1 --------------------------------------------------------------------------------
ArDIR19    1 --------------------------------------------------------------------------------
ArDIR23    1 MAKLAITANTLKGFFCLVLLAITIGCANSARILDEVDPQPPILPYPPQSTNPTATPV-----------------------
ArDIR26    1 --------------------------------------------------------------------------------

AtDIR10   73 GSGSTGSGLGAGTGSIPSSGSGPGLLPTASSVPGSLAGGGSGSLPTTGSATGAGAGTGSAL-----------GGGPGAGS
ArDIR18    1 -------------------------MHHRKT------------FPISSMAA----------------------SEPSNIF
ArDIR24    1 --------------------------------------------------------------------------------
ArDIR19    1 ------------------------------------------------------------------------------ME
ArDIR23   58 -PDVTPQVVGPTTA----------ILP-------------SGQIPASTVADDVDVDVDVAPVASPDVEPDVDSPQPEPVE
ArDIR26    1 --------------------------------------------------------------------------------

AtDIR10  142 ALGGGAGAGPALGGGAGAGPALGGGAGAGSALGGGGAGAGPALGGGG--AGAGPALGGGVA---GSGSALGGGASAGPDN
ArDIR18   22 PLK-------NLL------RLLL------LALTITCTASARILDEETTVPTTEPPVSS------------VPPVVTAGNR
ArDIR24    1 -----------MSKGL---------SSASFFLTML----------------------IAVVHVGMIMAAVDPVAATGKEP
ArDIR19    3 THK-------AIA------NLLF------LTLTILSATSARILDGQPAVPVVPPEATNGV---------DTPVTNADPSH
ArDIR23  114 PIVDSPQPEPEEP------D---------TVPTPVTTGVTPVVGP--TIP----APTGPVAPVATTAGATVAKPGGPQAS
ArDIR26    1 -----------MA-----------KLPSISMLTIACALLFMFIIHQQ-------------ASSARTIGN-PTPTPRKHYH

AtDIR10  217 TLVFFMHDILGG-SNPTARAVTGVVANPALSGQLPFAKPNGANLPVSNGVPSN---NNNNGIVNNNNVPFLVGLGGTTA-
ArDIR18   71 PMSFFMHDILGG-SNPSAKAVSGVVSNPAVNGQVPFAKPNGAVLPVNNGVPQSN-NNNNNGVINNNNIPFLTGLSGNIP-
ArDIR24   39 ILELYMHDILGG-NSPTARPITGLLGN-IYNGQVPFARPIGFVPPQG-GVAIPNANGAIPTVNGVTGIPLGTGLTGAGN-
ArDIR19   55 TLTFFLHDILGG-SNPTAEAVTGIVNNPAINGQLPFAKPNGAVLPVSNGNNGLT-DN-----NNNNNIPFLTGLSGATA-
ArDIR23  173 GLTFFMHDILGG-IHPSARVVTGIIANTQING-LPFTTPNNDIFPVSGGVPLSN--ANLNGILNNNNLPFLAGLNGVGPQ
ArDIR26   45 KITFLMRDVLINVTQPSSKPATAK----VTSTQLPFPKPLGLFPPNG-GIPVLL--GSNPTVPG-----TAAGLSTQTL-

AtDIR10  292 --NILQNNNNGNNI--LNGFPVASGGQLPSGSALQMLMFGTMTVIDDELTEGHEL--GSGLLGKAQGYYVASAIDGTSQT
ArDIR18  148 --NVIQNNGNNG------NN-GINGAQLPAGMTLQKLMFGTLSVFDNELTEGHEL--GSGLVGKAQGFYVASSEDGTSQT
ArDIR24  115 ----------A---------NNQNQPQIQLGPDGLGLGFGTITVIDDVLTSGPEL--GSQTIGKAQGVYVASSADGITQM
ArDIR19  127 --AVMQNNGGFG------NVPVVNGGQLPSGNTLQKLMFGTMTVFDDELTEGHEL--RSGLVGKAQGFYVGSSEDGTGQT
ArDIR23  249 ASTVIQNSGNNGIVNGGNNLPFVTAGQLPSGATLQKLMFGSLTVIDDELTEGHVL--GSAVVGKAQGFYLASSLDGTSQT
ArDIR26  112 -------------------DLSAIGLSFPASATLQELEFGTVTVIDQELFKGSSKNNMSPMLGKAQGIYVASSEDGSSHM

AtDIR10  366 MAFTAMFESGG----YEDSISFFGVLRTAVSESHIGVMGGTGKYVNARGFAILKTFTGSSGTQQNQPHQFTDGLETVVEC
ArDIR18  217 MAFTTMFQSGG----YADSLSFFGVHRTAASESQLAVMGGTGKYVDAKGYATVKTFPATN-----QQ--NNGGVETLLQF
ArDIR24  174 MAFTAVIEGGE----YGDSLNFYGIYKIGSTLSYLSVTGGTGKFKNASGFAEVRSLIP-------PGQHVLDGAETLLRI
ArDIR19  197 MAFTVMFESGR----YADSLSFFGIHRTAVSESQLAIMGGTGKYVNAKGFATVKTYAETN-----NQQHETDGLETLLEF
ArDIR23  327 MAVTVLLHGAGQHEVVEDTISFFGVHRTASLESQIAVVGGTGKYENAKGYATVETLHS-------EDQHTTDGVDTILQF
ArDIR26  173 MAMTAYFAGSD---GFKDGLRFFGVHQTDVPESHIAVVGGIGKYQGANGYAKVKAVDDHVGSHTD---EEGKGTNKLLLF

AtDIR10  442 TVYLSY
ArDIR18  286 TVYLTY
ArDIR24  243 TVHLTY
ArDIR19  268 TVYVTY
ArDIR23  400 SVYLSE
ArDIR26  247 HVYLS-
```

Supplementary Figure S45. Protein sequence alignment of *bona fide* dirigent proteins from Dir-e sub-family, AtDIR10 from *A. thaliana* (42) and that of putative homologs (ArDIR18, 19, 23, 24 and 26) in red alder.

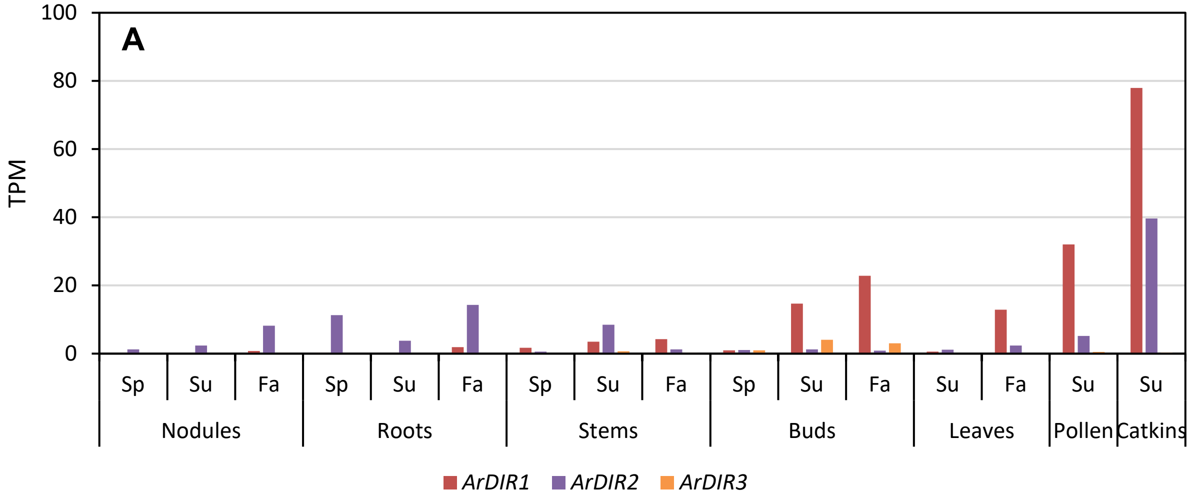

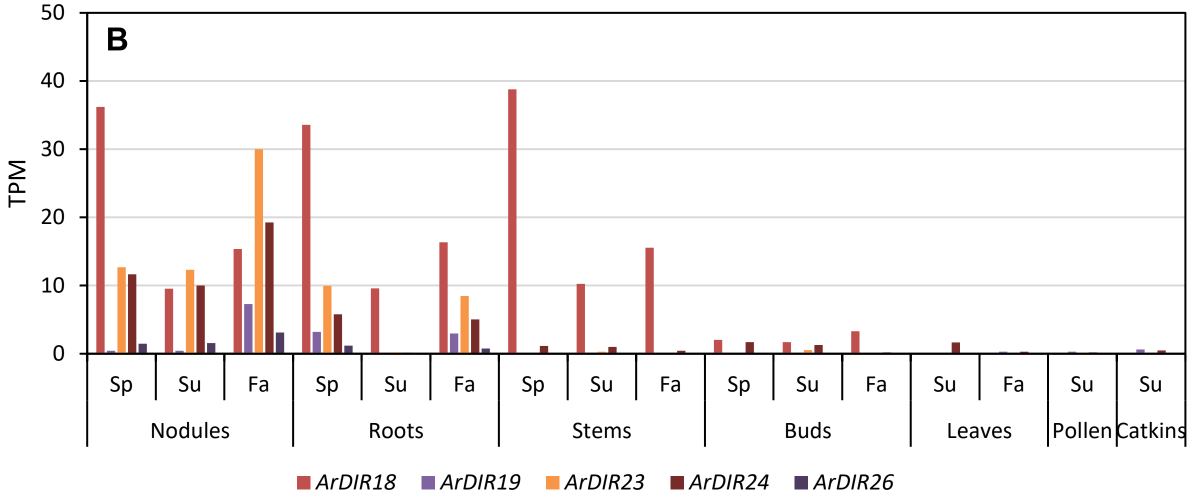

Supplementary Figure S46. Expression levels of red alder homologs of *bona fide* dirigent protein genes in the DIR-a **(A)** and DIR-e **(B)** sub-families. Abbreviations: TPM, transcripts per million; Sp, spring; Su, summer; Fa, fall.

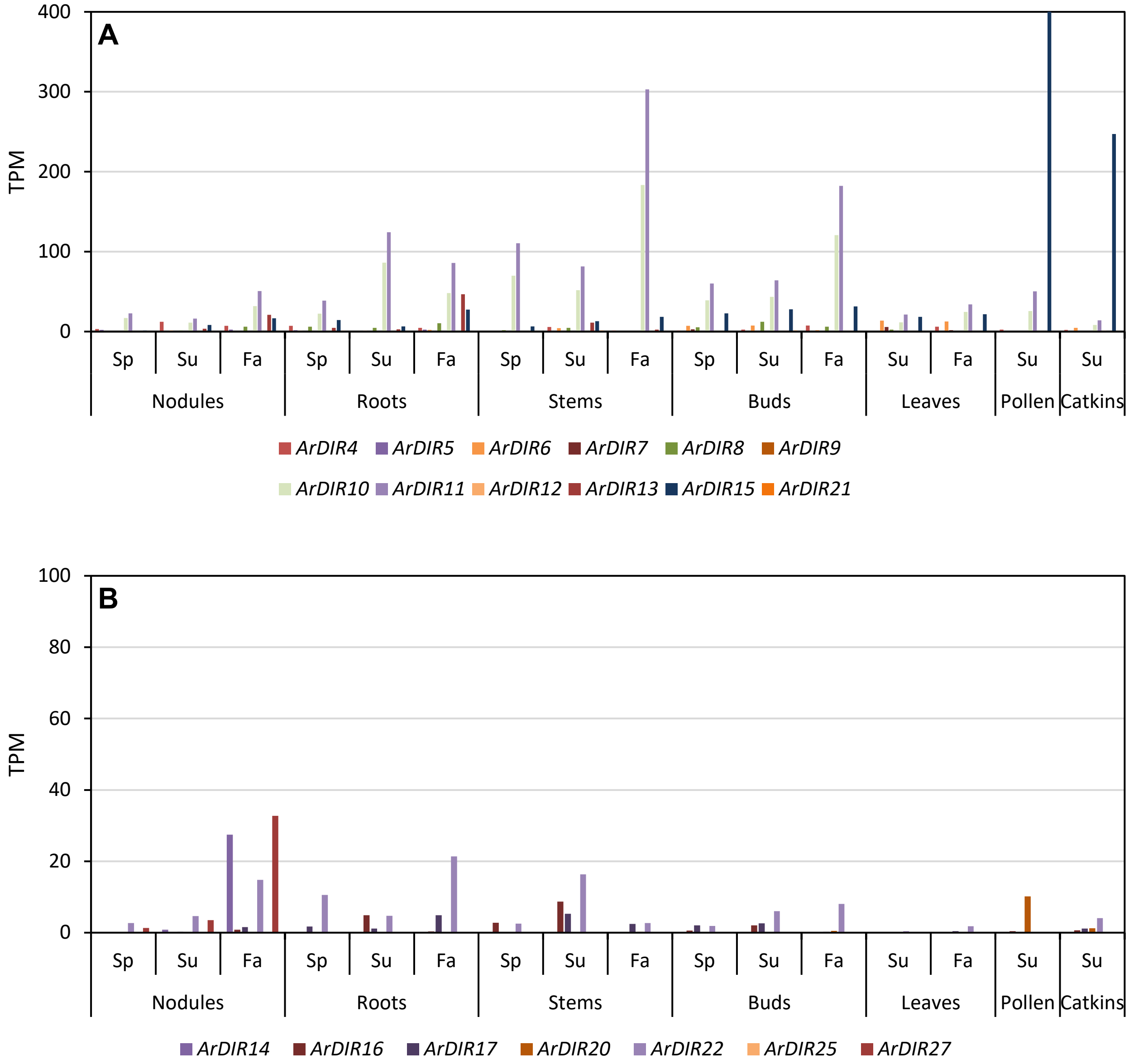

Supplementary Figure S47. Expression levels of red alder homologs of *bona fide* dirigent protein genes in the DIR-b **(A)** and Dir-d **(B)** sub-families. Abbreviations: TPM, transcripts per million; Sp, spring; Su, summer; Fa, fall.

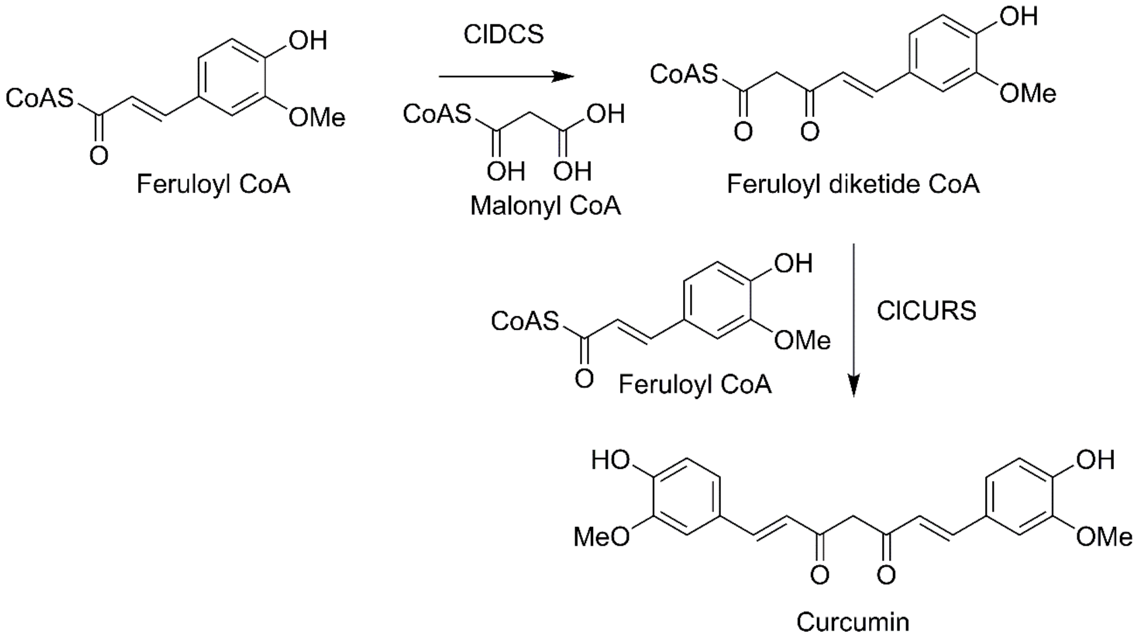

Supplementary Figure S48. Pathway to curcumin in *Curcuma longa* (Cl). ClDCS, diketide-CoA synthase; ClCURS, curcumin synthase (61).

```
FiPLR1    1 --MGKSKVLIIGGTGYLGRRLVKASLAQGHETYILHRPEI-GVDIDKVEMLISFKMQGAHLVSGSFKDFNSLVEAVKLVD
ArPLR1    1 --MEKSRVLIVGGTGYLGKRLVKASLDQGHETYVLQRPEI-GVDIEKVQMLLSFKKQGAHLVSGSFTDHQSLVNAVKLVD
ArAPS1    1 -MAHKSKILFIGGTGYIGKFIVEASAKSGHPTFALVREST-VSDPVKGKLVEHFKSLGVTLLHGDLYDHASLVKAIKHVD
ArAPS2    1 -MAHKSKILFIGGTGYIGKFIVEASAKSGHPTFALVREST-VSDPVKGKLVEHFKSLGVTLLHGDLYDHASLVKAIKHVD
ArAPS3    1 -MAQKSRILIIGGTGYIGKFIVAASAKAGHPTFALIREST-RSDPVKGKLVENFKNLGVTLLHGDLHDHGSLVKAIKQVD
ArAPS4    1 -MAQKSKILIIGSTGFIGKFIVEASAKSGHPTFALVREST-VSDPIKGKLVQHFKSLGVTLLHGDLYDHASLVKAIKQVD
ArAPS5    1 -MAQKSKILIIGSTGFIGKFIVEASAKSGHPTFALVREST-VSDPIKGKLVQHFKSLGVTLLHGDLYDHASLVKAIKQVD
ArPPS1    1 MANEKSKILIFGGTGYIGNYLARASCLLGHPTYVYARPLTPQTTPSKLELHKQLHSMGATFVLGELEEHEKIVSAVREVD
ArPPS2    1 MENEKSKILIFGGTGYIGNYLAKASCLLGHPTYVYARPLTPQTTPSKLELHKELHSMGATFVLGELEEHEKIVSALREVD

FiPLR1   78 VVISAISGVHIRSHQILLQLKLVEAIKEAGNVKRFLPSEFGMDPAKFMDTAMEPGKVTLDEKMVVRKAIEKAGIPFTYVS
ArPLR1   78 VVICAISGVHIRTHQILLQLKLVDAIKEAGNVKRFLPSEFGTDPAR-MPNALEPGRVTFDDKMVVRKAIQDAKIPFTYVS
ArAPS1   79 VVISTVGHLQLAD-----QVKIIAAIKEAGNVKRFFPSEFGNDVDR--VHAVEPAKTAFATKVEIRRKIEAEGIPYTYVS
ArAPS2   79 VVISTVGHLQLAD-----QVKIIAAIKEAGNVKRFFPSEFGNDVDR--VHAVEPAKTAFATKVEIRRKIEAEGIPYTYVS
ArAPS3   79 VVISTVGRLQFGD-----QVKIIAAIKEAGNVKRFFPSEFGNDVDR--VHAIGPAKTAFATKAKIRRTIEAEGIPYTYVT
ArAPS4   79 VVISAVGHPQVED-----QDKIIAAIKEAGNVKKFFPSEFTNDVDR--VHAVGPAQAAFDTRVEIRRKIETAGIPYTYVA
ArAPS5   79 VVISAVGHPQVED-----QDKIIAAIKEAGNVKKFIPSEFTNDVDR--VHAVGPAKAAFDTRVEIRRKIETAGIPYTYVA
ArPPS1   81 IVISALAYPQVLD-----QLKIIDAIKVAGNIKRFLPSDFGVEEDR--VTPLPPFEAFLDKKRKIRRAAEAAGIPYTFVS
ArPPS2   81 IVISALAYPQVLE-----QLKIIDAIKVAGNIKRFLPSDFGVEEDR--ITPLPPFEAFLDKKRKIRRVVEAAGIPYTFVS

FiPLR1  158 ANCFAGYFLGGLCQFGKI--------LPSRDFVIIHGDGNKKAIYNNEDDIATYAIKTINDPRTLNKTIYISPPKNILSQ
ArPLR1  157 ANCFAGYFLGGLCQPGYI--------LPSRDSVVLFGDGNTKAIYVDEDDIAMYTIKTIDDPRTLNKTVYIRPPKNILSQ
ArAPS1  152 SNYFAGYFLPTLAQPGLT--------SPPRDKITIFGDGHVKAIFNKEDDIGTYTIRTVDDPRTLNKIVYLRPPKNIYSF
ArAPS2  152 SNYFAGYFLPTLAQPGLT--------SPPRDKITIFGDGHVKAIFNKEDDIGTYTIRTVDDPRTLNKIVYLRPPKNIYSF
ArAPS3  152 SNLFAGYFLPTLAQPGAR--------APSRDKVIIFGDGNAKAIFNKEEDIGSYTIRAVDDPRTLNKILYLRTPKNIYSL
ArAPS4  152 SNWFAGYFLPTLAQPGFTAPPIEKVTTPPREKVTIFGDGNAKAIFNAEDDIGTYTIRAVDDPRTLNKIVYLRPPKNIYSF
ArAPS5  152 SNWFAGYFLPTLAQPGFTAPPIEKVTTPPREKVTIFGDGNAKAIFNAEDDIGTYTIRAVDDPRTLNKIVYLRPPKNIYSF
ArPPS1  154 ANCCASYFLSHLLHPHEK-----------GDDIAVYGSGEAKAVLNYEEDIATYTIKVANDPRTCNRIVIYRPPKNILSQ
ArPPS2  154 ANCFGSYFLNYLLHPHEK-----------GDDIAVYGSGEAKVVLNYEEDIATYTIKVANDPRTCNRVVIYRPLKNILSQ

FiPLR1  230 REVVQTWEKLIGKELQKITLSKEDFLASVKELEYAQQVGLSHYHDVNYQGCLTSFEIG--DEEEASKLYPEVKYTSVEEY
ArPLR1  229 REVVEIWEKLIGQELHKSSISKEDFLASIKGLEYAEQVGLIHYYHVCYEGCLTNFEIGK-DAGEASELYPEIDYTSVHDY
ArAPS1  224 NELVALWEKKIGKTLEKTYVSEEKILKDIQEAPIPINVILAINHSVFVKGDHTNFEIEASFGVEASELYPDVKYTTVEEY
ArAPS2  224 NELVALWEKKIGKTLEKTYVSEEKILKDIQEAPIPINVILAINHSVFVKGDHTNFEIEASFGVEASELYPDVKYTTVEEY
ArAPS4  224 NELVALWEKKIGKTLEKIHVSEENILKDIKESEFPVNAILSIIHSVFVKGDETSFEIEASFGVEASELYPDFNYTTVDEY
ArAPS5  232 NELVALWEKKIGKTLKKIYVPEEKLLKDIQESPIPTNLILAIYHSVLVKGDHTNFEIEASFGVEASELYPDVRYTTVEEY
ArPLR6  232 NELVALWEKKIGKTLKKIYVPEEKLLKDIQESPIPTNLILAIYHSVLVKGDHTNFEIEASFGVEASELYPDVRYTTVEEY
ArPPS1  224 LELISLWEKKTGRSFNKVYVSEEEIVKLSETLPTAEAIPISILHSIFIKG-QMVFELRE-DDLEASRLYPELEYTTVDKL
ArPPS2  224 LELISLWEKTTGRSFNRIHVPEEEIVKLSETLPPPKDIPMSILHSIFIKG-QTVFELRE-DDMEASRLYPDFKYTTIDQL

FiPLR1  308 LKRYV--------------------
ArPLR1  308 MKRYL--------------------
ArAPS1  304 LQQFV--------------------
ArAPS2  304 LQQFV--------------------
ArAPS3  304 LDPFVGDFMEKGLSVPALFGYCNEP
ArAPS4  312 LQQFV--------------------
ArAPS5  312 LQQFV--------------------
ArPPS1  302 LDIFLVNPPKPTMGSFE--------
ArPPS2  302 LDIFLVNPPKFAAAAFE--------
```

Supplementary Figure S49. Protein sequence alignment of *bona fide* pinoresinol-lariciresinol reductase from *Forsythia intermedia* (FiPLR1 (43)) and that a putative homolog (ArPLR1), as well as that of allylphenol and propenylphenol synthases (ArAPS and ArPPS) in red alder.

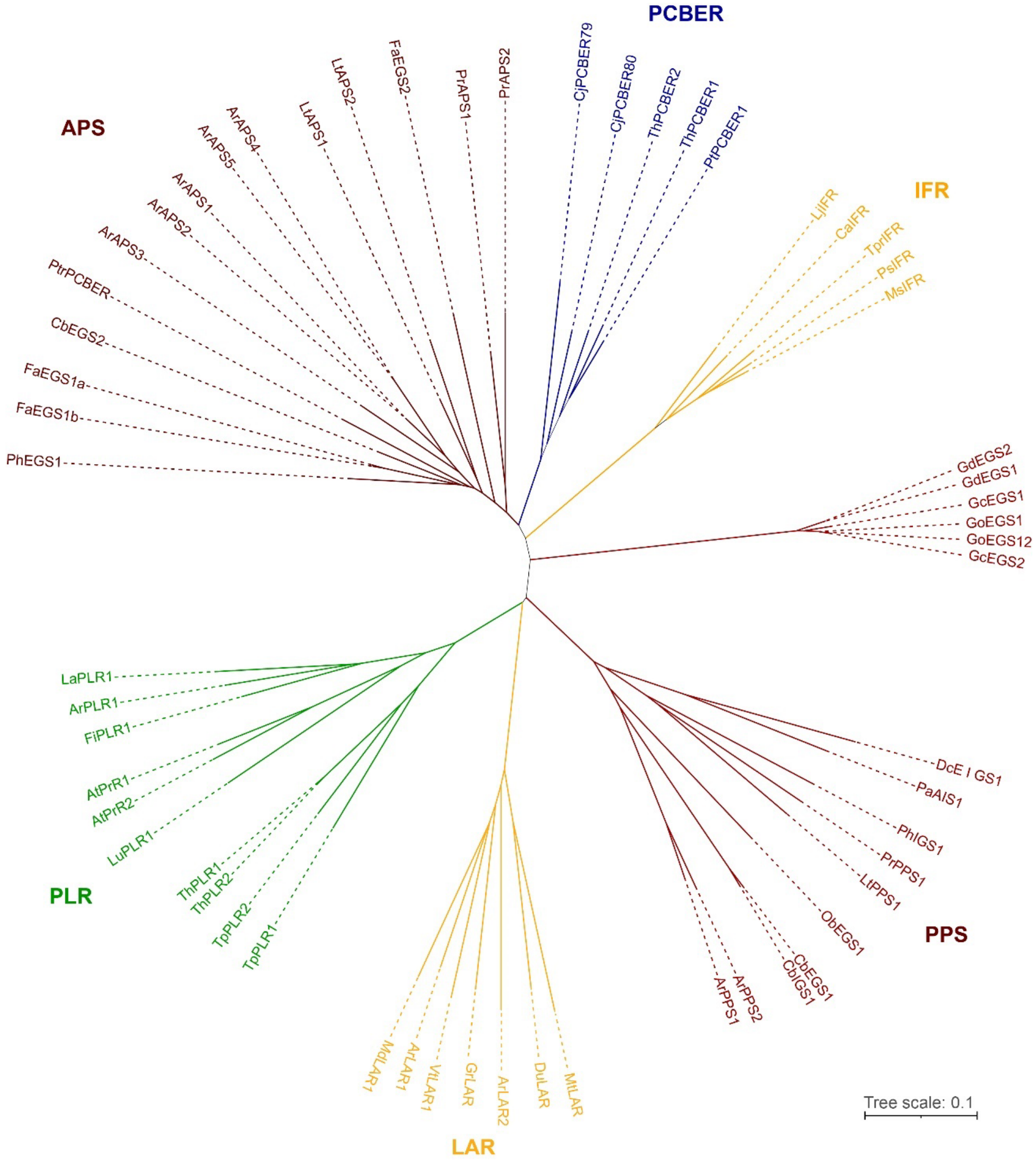

Supplementary Figure S50. Unrooted phylogenetic tree of known pinoresinol-lariciresinol reductases (PLRs), phenylcoumaran benzylic ether reductases (PCBERs), allylphenol and propenylphenol synthases (APS, PPS), and isoflavone reductases (IFRs), as well as leucoanthocyanidin reductases (LARs) from *Alnus rubra* (Ar), *Arabidopsis thaliana* (At), *Cicer arietinum* (Ca), *Clarkia breweri* (Cb), *Cryptomeria japonica* (Cj), *Daucus carota* (Dc), *Desmodium uncinatum* (Du), *Forsythia intermedia* (Fi), *Fragaria × ananassa* (Fa), *Gossypium raimondii* (Gr), *Gymnadenia conopsea* (Gc), *G. densiflora* (Gd), *G. odoratissima* (Go), *Larrea tridentata* (Lt), *Linum album* (La), *Linum corymbulosum* (Lc), *Linum usitatissimum* (Lu), *Lotus japonicus* (Lj), *Malus domestica* (Md), *Medicago sativa* (Ms), *Medicago truncatula* (Mt), *Ocimum basilicum* (Ob), *Petunia hybrida* (Ph), *Pimpinella anisum* (Pa), *Pinus taeda* (Pt), *Piper regnellii* (Pr), *Pisum sativum* (Ps), *Populus trichocarpa* (Ptri), *Thuja plicata* (Tp), *Trifolium pratense* (Tpr), *Tsuga heterophylla* (Th), and *Vitis vinifera* (Vt). Sequences were

obtained from NCBI and a multiple sequence alignment was built with Clustal Omega (57). The tree was rendered with iTOL (58).

PLRs: AtPrR1 (Q9FVQ6), AtPrR2 (Q9SVP6), FiPLR (AAC49608), LaPLR1 (Q4R0I0), LuPLR1 (CAH60858), TpPLR2 (Q9LD13), TpPLR1 (Q9LD14), ThPL1R (AAF64184), and ThPLR2 (AAF64185).

PCBERs: CjPCBER79, CjPCBER80, LcPCBER1 (ACA60729), LcPCBER2 (ACA60730), PtPCBER (AAC32591), PtrPCBER (CAA06706), ThPCBER1 (AAF64176), and ThPCBER2 (AAF64177).

APSs and PPSs: CbIGS1 (ABR24112), CbEGS1 (ABR24113), CbEGS2 (ABR24114), *DcE(I)GS1* (XP_017241251), FaEGS1a (AGV02006), FaEGS1b (AGV02007), FaEGS2 (AGV02008), GcEGS1 (AKB11747), GcEGS2 (AKB11748); GdEGS1 (AKB11749), GdEGS2 (AKB11750), GoEGS1 (AKB11751), GoEGS2 (AKB11752), LtAPS1 (AHA90804), LtAPS2 (AHA90805), LtPPS1 (AHA90806), ObEGS1 (ABD17321), PhIGS1 (ABD17322), PhEGS1 (ABR24115), PaAIS1 (ACL13526), PrAPS1 (AHA90807), PrAPS2 (AHA90808), and PrPPS1 (AHA90809).

IFRs: CaIFR (Q00016), LjIFR (QBF58800), MsIFR (AAC48976), PsIFR (P52576), and TprIFR (QBF58799).

LARs: DuLAR (Q84V83), GrLAR (CAI56324), MdLAR1 (AAZ79364), MtLAR (CAI56327), and VtLAR1 (CAI26309).

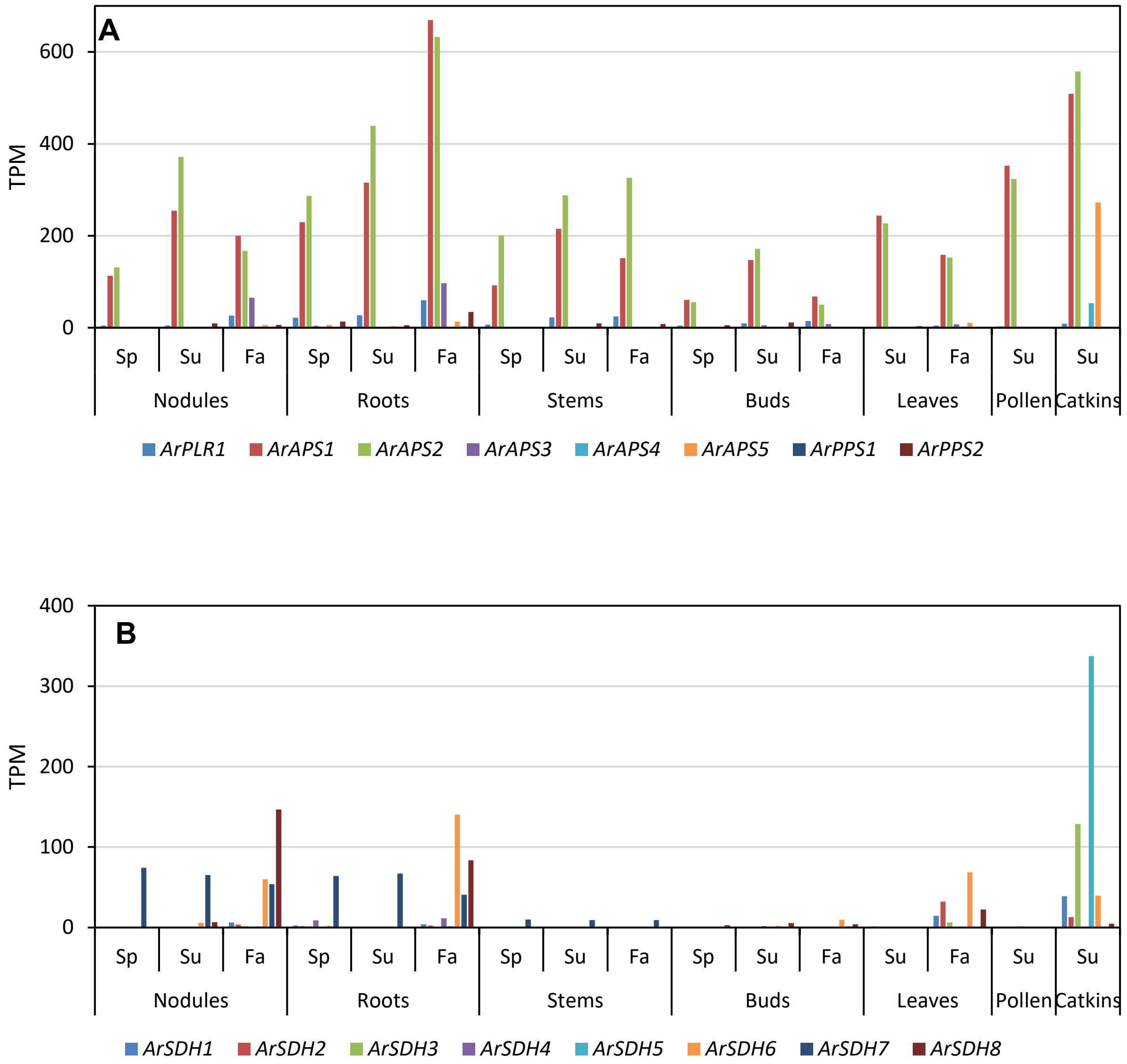

Supplementary Figure S51. Expression levels of red alder homologs of **(A)** *bona fide* pinoresinol lariciresinol reductase (*ArPLR*), allylphenol synthase (*ArAPS*) and propenylphenol synthase (*ArPPS*) genes, and **(B)** secoisolariciresinol dehydrogenase (*ArSDH*) genes. Abbreviations: TPM, transcripts per million; Sp, spring; Su, summer; Fa, fall.

```
PpSDH     1 -------------------------------MGSTSTPDSSTNRLQDKVAIITGGAGGIGETTAKLFVRYGAKVVIADIA
ArSDH1    1 HSLFYYKRPSKPADLHIEKHSHILTNNKLKMEGASLPPAPIFKRLEGKVALITGGASGIGETTARLFVQHGAKVIIADVQ
ArSDH2    1 ------------------------------MEGALLPPAPIFKRLEGKVALITGGASGIGETTARLFVQHGAKVIIADVQ
ArSDH3    1 HSLFYYKRPSKPADLHIEKHSQILTNNKLKMEGASLPPAPIFKRLEGKVALITGGASGIGETTARLFVQHGAKVIIADVQ
ArSDH4    1 ------------------------------MEGASL-PAPIAKRLEGKVALITGGASGMGEATARLFVQHGAKVIIADVQ
ArSDH5    1 ------------------------------MKGASL-LAPIAKRLEGKVALITGGASGIGETTARLFVQHGAKVIIADVQ
ArSDH6    1 -------------------------------MGSFSLLSAAARRLEGKVALITGGASGIGESTVRLFSKHGAKVVIADIQ
ArSDH7    1 -------------------------------MATSSLLSAVTRRLEGKVALITGGASGIGECTAKVFAEHGAKVVIADIQ
ArSDH8    1 -------------------------------MGSVSLVSAAARRLEGKVALITGGASGIGESTARLFSKHGAKVVIADIQ

PpSDH    50 DDHGQKVCNNIGSP-DVISFVHCDVTKDEDVRNLVDTTIAKHGKLDIMFGNVGVLSTTPYSILEAGNEDFKRVMDINVYG
ArSDH1   81 DDIGHSVCRDIGTA-DDISYVHCDVTNESDVQNAVKTAVSKHGKLDILFSNAGITNA-DDGILEFDSEKYNRVFNVNVLG
ArSDH2   51 DDIGHSVCRDIGTA-DDISYVHCDVTNESDVQNAVKTAVSKHGKLDILFSNAGITNA-DDGILEFDSEKYNRVFNVNVLG
ArSDH3   81 DDIGHSVCRDIGTA-DDISYVHCDVTNESDVQNAVKTAVSKHGKLDILFSNAGITNA-DDGILEFDREKYNRVFNVNVLG
ArSDH4   50 DELGYSVCRDIGTNTDDISYVHCDVTNESDVQNAVETAVSKHGKLDILFSNAGITNA-DNSILDFDLEKYNKVFNVNVLG
ArSDH5   50 DDLGHSICSDISTGDDMISYVHCDVTNESDVQNAVNIVVSKHAKLDILFSNAGITNA-DEGILDFHREKFSRVFNVNVLG
ArSDH6   50 DDLGHSVCKDLNSQ--STSFVHCDVTKEKDVENAVNLAVSKFGKLDIMFNNAGIAGVAKTDIREITKAEFEQVIGINLVG
ArSDH7   50 DELGHAVNEALGPS--NSLYVHCDVTDEAHIKNAVEKAVDTYGKLDIMFNNAGIADDNKARIIDNEKSDFERVLSVNVTG
ArSDH8   50 DDLGHSVCKDLNSQ--STSFVHCDVTKEKDVENAVNLAVSKFGKLDIMFNNAGIAGVAKTNILEITKAEFEQVVGINLMG

PpSDH   129 AFLVAKHAARVMIPAKKGSIVFTASISSFTAGEGVSHVYTATKHAVLGLTTSLCTELGEYGIRVNCVSPYIVASPLLTDV
ArSDH1  159 GFLAAKHAAKVMIPAKKGSILFTSSTASVIHGE-TPHIYAASKHALVGLTKNLCVELGEYGIRVNCVSPSGVATPLLRNV
ArSDH2  129 GFLAAKHAAKVMIPAKKGSILFTSSTASVIHGE-TPHIYAASKHALVGLTKNLCVELGEYGIRVNCVSPSGVATPLLRNV
ArSDH3  159 GFLAAKHAAKVMIPAKKGSILFTSSTASVIHGE-TPHIYAASKHALVGLTKNLCVELGEYGIRVNCVSPSGVATPLLRNV
ArSDH4  129 GFLAAKHAAKVMIPAKKGSILFTSSTASVIHGE-TPHIYTASKHAVVGLTKNLCVELGEYGIRVNCLSPSGVATPLLRNV
ArSDH5  129 GFLAAKYGAKVMIPAKKGSILFTSSIASVIHGT-TPHIYTASKHALVGLSKNLCAELGQYGIRVNCISPAGVATPLLRNV
ArSDH6  128 VFLGTKHAARVMIPAHHGSIITTASVCSTVGGT-ASHGYTSSKHGVVGLMRNTAVELGQFGIRVNCVSPYLVLTPLAKDF
ArSDH7  128 VFLGIKHAAQAMIPARSGSIISTASISSNIGGA-ASHAYACSKHAVLGLTKNAAVELGQFGIRVNCLSPYALATPLARKF
ArSDH8  128 VFLGTKHAARVMIPACHGSIITTASAASTVGGS-ASHGYTSSKHGVVGLMRNTAVELGQFGIRVNCVSPYVVPTPLAKDF

PpSDH   209 FGVDSS-RVEELAHQAANLKGTLLRAEDVADAVAYLAGDESKYVSGLNLVIDGGYTRTNPAFPTALKHGLA-
ArSDH1  238 SGIQDKKTLEDMICSAANLKGVVLETDDVAEAALFLVSDESKYVSGLNLVLDGGHSTTNKAIRDGKEKHRS-
ArSDH2  208 SGIQDKKTLEDMICSAANLKGVVLGTDDVAEAALFLVSDESKYVSGLNLVLDGGHSTTNKAIRDGKEKHRS-
ArSDH3  238 SGIQDKKTLEDMICSSANLKGVVLGTDDVAEAALFLVSDESKYVSGLNLVLDGGHSTTNKAIRDGKETHRS-
ArSDH4  208 CGIQDKKTLEDMICSAANLKGVVFRTEDVAEAALFLASDESKYVSGLNLILDGGRSTTNEAIKNAKEKYGSN
ArSDH5  208 SGIQDKKTLEDMICSSANLKGVVLGTDDVAEAALFLASDESKYVSGLNLVLDGGHSTTNKAIRDAKENHRS-
ArSDH6  207 LKLDDD-GVHRA---YSNLEGKGLEAEDVAEAVLYLGSDESKYVSGHNLLVDGGFTIENSGFSMYA------
ArSDH7  207 VGLDDE-GLENVMNSLANLKGVTLKAEDVANAALYLASNESRYVSGHNLFIDGGFSIHNSAFQIFQYPDS--
ArSDH8  207 YKLDND-GIHRA---YSNLKGKVLEAEDVAEAVLYLGSDESKYVSGHNLLVDGGFTIENSGFSMYA------
```

Supplementary Figure S52. Protein sequence alignment of *bona fide* secoisolariciresinol dehydrogenase from *Podophyllum peltatum* (PpSDH1 (45)) and that of putative homologs (ArSDH1 – ArSDH8) in red alder.

Supplementary Material References

1. Sati, S. C., Sati, N., and Sati, O. P. (2011) Bioactive constituents and medicinal importance of genus *Alnus*. *Pharmacogn. Rev.* **5**, 174–183
2. Jackrel, S. L. (2016) Drivers and consequences of intraspecific trait variation: Ecology of a forest-stream community. *Ph.D. Thesis, The University of Chicago*
3. Jackrel, S. L., Morton, T. C., and Wootton, J. T. (2016) Intraspecific leaf chemistry drives locally accelerated ecosystem function in aquatic and terrestrial communities. *Ecology* **97**, 2125–2135
4. Keith, B., Dong, X., Ausubel, F. M., and Fink, G. R. (1991) Differential induction of 3-deoxy-D-arabino-heptulosonate 7-phosphate synthase genes in *Arabidopsis thaliana* by wounding and pathogenic attack. *Proc. Natl. Acad. Sci. USA* **88**, 8821–8825
5. Bischoff, M., Roesler, J., Raesecke, H.-R., Goerlach, J., Amrhein, N., and Schmid, J. (1996) Cloning of a cDNA encoding a 3-dehydroquinate synthase from a higher plant, and analysis of the organ-specific and elicitor-induced expression of the corresponding gene. *Plant Mol. Biol.* **31**, 69–76
6. Singh, S. A., and Christendat, D. (2006) Structure of *Arabidopsis* dehydroquinate dehydratase-shikimate dehydrogenase and implications for metabolic channeling in the shikimate pathway. *Biochemistry* **45**, 7787–7796
7. Fucile, G., Garcia, C., Carlsson, J., Sunnerhagen, M., and Christendat, D. (2011) Structural and biochemical investigation of two *Arabidopsis* shikimate kinases: The heat-inducible isoform is thermostable. *Protein Sci.* **20**, 1125–1136
8. Klee, H. J., Muskopf, Y. M., and Gasser, C. S. (1987) Cloning of an *Arabidopsis thaliana* gene encoding 5-enolpyruvylshikimate-3-phosphate synthase: Sequence analysis and manipulation to obtain glyphosate-tolerant plants. *Mol. Gen. Genet.* **210**, 437–442
9. Schaller, A., Schmid, J., Leibinger, U., and Amrhein, N. (1991) Molecular cloning and analysis of a cDNA coding for chorismate synthase from the higher plant *Corydalis sempervirens* Pers. *J. Biol. Chem.* **266**, 21434–21438
10. Eberhard, J., Ehrler, T. T., Epple, P., Felix, G., Raesecke, H.-R., Amrhein, N., and Schmid, J. (1996) Cytosolic and plastidic chorismate mutase isoenzymes from *Arabidopsis thaliana*: Molecular characterization and enzymic properties. *Plant J.* **10**, 815–821
11. Maeda, H., Yoo, H., and Dudareva, N. (2011) Prephenate aminotransferase directs plant phenylalanine biosynthesis via arogenate. *Nat. Chem. Biol.* **7**, 19–21
12. Cho, M.-H., Corea, O. R. A., Yang, H., Bedgar, D. L., Laskar, D. D., Anterola, A. M., Moog-Anterola, F. A., Hood, R. L., Kohalmi, S. E., Bernards, M. A., Kang, C., Davin, L. B., and Lewis, N. G. (2007) Phenylalanine biosynthesis in *Arabidopsis thaliana*: Identification and characterization of arogenate dehydratases. *J. Biol. Chem.* **282**, 30827–30835
13. Mittasch, J., Böttcher, C., Frolova, N., Bönn, M., and Milkowski, C. (2014) Identification of UGT84A13 as a candidate enzyme for the first committed step of gallotannin biosynthesis in pedunculate oak (*Quercus robur*). *Phytochemistry* **99**, 44–51
14. Madeira, F., Park, Y. m., Lee, J., Buso, N., Gur, T., Madhusoodanan, N., Basutkar, P., Tivey, A. R. N., Potter, S. C., Finn, R. D., and Lopez, R. (2019) The EMBL-EBI search and sequence analysis tools APIs in 2019. *Nucleic Acids Res.* **47**, W636–W641
15. Sperschneider, J., Catanzariti, A. M., DeBoer, K., Petre, B., Gardiner, D. M., Singh, K. B., Dodds, P. N., and Taylor, J. M. (2017) LOCALIZER: Subcellular localization prediction of both plant and effector proteins in the plant cell. *Sci Rep* **7**, 44598

16. Thumuluri, V., Almagro Armenteros, J. J., Johansen, Alexander R., Nielsen, H., and Winther, O. (2022) DeepLoc 2.0: Multi-label subcellular localization prediction using protein language models. *Nucleic Acids Res.* **50**, W228–W234
17. Cochrane, F. C., Davin, L. B., and Lewis, N. G. (2004) The *Arabidopsis* phenylalanine ammonia-lyase gene family. Kinetic characterization of the four PAL isoforms. *Phytochemistry* **65**, 1557–1564
18. Teutsch, H. G., Hasenfratz, M. P., Lesot, A., Stoltz, C., Garnier, J. M., Jeltsch, J. M., Durst, F., and Werck-Reichhart, D. (1993) Isolation and sequence of a cDNA encoding the Jerusalem artichoke cinnamate 4-hydroxylase, a major plant cytochrome P450 involved in the general phenylpropanoid pathway. *Proc. Natl. Acad. Sci. USA* **90**, 4102–4106
19. Hoffmann, L., Maury, S., Martz, F., Geoffroy, P., and Legrand, M. (2003) Purification, cloning, and properties of an acyltransferase controlling shikimate and quinate ester intermediates in phenylpropanoid metabolism. *J. Biol. Chem.* **278**, 95–103
20. Schoch, G., Goepfert, S., Morant, M., Hehn, A., Meyer, D., Ullmann, P., and Werck-Reichhart, D. (2001) CYP98A3 from *Arabidopsis thaliana* is a 3′-hydroxylase of phenolic esters, a missing link in the phenylpropanoid pathway. *J. Biol. Chem.* **276**, 36566–36574
21. Costa, M. A., Bedgar, D. L., Moinuddin, S. G. A., Kim, K.-W., Cardenas, C. L., Cochrane, F. C., Shockey, J. M., Helms, G. L., Amakura, Y., Takahashi, H., Milhollan, J. K., Davin, L. B., Browse, J., and Lewis, N. G. (2005) Characterization *in vitro* and *in vivo* of the putative multigene 4-coumarate CoA ligase network in *Arabidopsis*: Syringyl lignin and sinapate/sinapyl alcohol derivative formation. *Phytochemistry* **66**, 2072–2091
22. Meyer, K., Cusumano, J. C., Somerville, C., and Chapple, C. C. S. (1996) Ferulate-5-hydroxylase from *Arabidopsis thaliana* defines a new family of cytochrome P450-dependent monooxygenases. *Proc. Natl. Acad. Sci. USA* **93**, 6869–6874
23. Goujon, T., Sibout, R., Pollet, B., Maba, B., Nussaume, L., Bechtold, N., Lu, F., Ralph, J., Mila, I., Barriere, Y., Lapierre, C., and Jouanin, L. (2003) A new *Arabidopsis thaliana* mutant deficient in the expression of *O*-methyltransferase impacts lignins and sinapoyl esters. *Plant Mol. Biol.* **51**, 973–989
24. Moinuddin, S. G. A., Jourdes, M., Laskar, D. D., Ki, C., Cardenas, C. L., Zhang, D., Davin, L. B., and Lewis, N. G. (2010) Insights into lignin primary structure and deconstruction from *Arabidopsis thaliana* COMT (caffeic acid *O*-methyl transferase) mutant *Atomt1*. *Org. Biomol. Chem.* **8**, 3928–3946
25. Schmitt, D., Pakusch, A.-E., and Matern, U. (1991) Molecular cloning, induction, and taxonomic distribution of caffeoyl-CoA 3-*O*-methyltransferase, an enzyme involved in disease resistance. *J. Biol. Chem.* **266**, 17416–17423
26. Patten, A. M., Cardenas, C. L., Cochrane, F. C., Laskar, D. D., Bedgar, D. L., Davin, L. B., and Lewis, N. G. (2005) Reassessment of effects on lignification and vascular development in the *irx4 Arabidopsis* mutant. *Phytochemistry* **66**, 2092–2107
27. Kim, S.-J., Kim, M.-R., Bedgar, D. L., Moinuddin, S. G. A., Cardenas, C. L., Davin, L. B., Kang, C.-H., and Lewis, N. G. (2004) Functional reclassification of the putative cinnamyl alcohol dehydrogenase multigene family in *Arabidopsis*. *Proc. Natl. Acad. Sci. USA* **101**, 1455–1460
28. Sommer, H., and Saedler, H. (1986) Structure of the chalcone synthase gene of *Antirrhinum majus*. *Mol. Gen. Genet.* **202**, 429–434
29. van Tunen, A. J., Hartman, S. A., Mur, L. A., and Mol, J. N. (1989) Regulation of chalcone flavanone isomerase (CHI) gene expression in *Petunia hybrida*: The use of alternative promoters in corolla, anthers and pollen. *Plant Mol. Biol.* **12**, 539–551

30. Pelletier, M. K., and Shirley, B. W. (1995) A genomic clone encoding flavanone 3-hydroxylase (Accession No. U33932) from *Arabidopsis thaliana*. *Plant Physiol.* **109**, 1125
31. Schoenbohm, C., Martens, S., Eder, C., Forkmann, G., and Weisshaar, B. (2000) Identification of the *Arabidopsis thaliana* flavonoid 3′-hydroxylase gene and functional expression of the encoded P450 enzyme. *Biol. Chem.* **381**, 749–753
32. Holton, T. A., Brugliera, F., Lester, D. R., Tanaka, Y., Hyland, C. D., Menting, J. G. T., Lu, C.-Y., Farcy, E., Stevenson, T. W., and Cornish, E. C. (1993) Cloning and expression of cytochrome P450 genes controlling flower colour. *Nature* **366**, 276–279
33. Holton, T. A., Brugliera, F., and Tanaka, Y. (1993) Cloning and expression of flavonol synthase from *Petunia hybrida*. *Plant J.* **4**, 1003–1010
34. Beld, M., Martin, C., Huits, H., Stuitje, A. R., and Gerats, A. G. (1989) Flavonoid synthesis in *Petunia hybrida*: Partial characterization of dihydroflavonol-4-reductase genes. *Plant Mol. Biol.* **13**, 491–502
35. Tanner, G. J., Francki, K. T., Abrahams, S., Watson, J. M., Larkin, P. J., and Ashton, A. R. (2003) Proanthocyanidin biosynthesis in plants: Purification of legume leucoanthocyanidin reductase and molecular cloning of its cDNA. *J. Biol. Chem.* **278**, 31647–31656
36. Pelletier, M. K., Burbulis, I. E., and Winkel-Shirley, B. (1999) Disruption of specific flavonoid genes enhances the accumulation of flavonoid enzymes and end-products in *Arabidopsis* seedlings. *Plant Mol. Biol.* **40**, 45–54
37. Xie, D. Y., Sharma, S. B., Paiva, N. L., Ferreira, D., and Dixon, R. A. (2003) Role of anthocyanidin reductase, encoded by *BANYULS* in plant flavonoid biosynthesis. *Science* **299**, 396–399
38. Martens, S., and Forkmann, G. (1999) Cloning and expression of flavone synthase II from *Gerbera* hybrids. *Plant J.* **20**, 611–618
39. Seneviratne, H. K., Dalisay, D. S., Kim, K. W., Moinuddin, S. G. A., Yang, H., Hartshorn, C. M., Davin, L. B., and Lewis, N. G. (2015) Non-host disease resistance response in pea (*Pisum sativum*) pods: Biochemical function of DRR206 and phytoalexin pathway localization. *Phytochemistry* **113**, 140–148
40. Meng, Q., Moinuddin, S. G. A., Kim, S.-J., Bedgar, D. L., Costa, M. A., Thomas, D. G., Young, R. P., Smith, C. A., Cort, J. R., Davin, L. B., and Lewis, N. G. (2020) Pterocarpan synthase (PTS) structures suggest a common quinone methide-stabilizing function in dirigent proteins and proteins with dirigent-like domains. *J. Biol. Chem.* **295**, 11584–11601
41. Effenberger, I., Zhang, B., Li, L., Wang, Q., Liu, Y., Klaiber, I., Pfannstiel, J., Wang, Q., and Schaller, A. (2015) Dirigent proteins from cotton (*Gossypium* sp.) for the atropselective synthesis of gossypol. *Angew. Chem. Int. Ed. Engl.* **54**, 14660–14663
42. Hosmani, P. S., Kamiya, T., Danku, J., Naseer, S., Geldner, N., Guerinot, M. L., and Salt, D. E. (2013) Dirigent domain-containing protein is part of the machinery required for formation of the lignin-based Casparian strip in the root. *Proc. Natl. Acad. Sci. USA* **110**, 14498–14503
43. Dinkova-Kostova, A. T., Gang, D. R., Davin, L. B., Bedgar, D. L., Chu, A., and Lewis, N. G. (1996) (+)-Pinoresinol/(+)-lariciresinol reductase from *Forsythia intermedia*: Protein purification, cDNA cloning, heterologous expression and comparison to isoflavone reductase. *J. Biol. Chem.* **271**, 29473–29482
44. Kim, S.-J., Vassão, D. G., Moinuddin, S. G. A., Bedgar, D. L., Davin, L. B., and Lewis, N. G. (2014) Allyl/propenyl phenol synthases from the creosote bush and engineering production of specialty/commodity chemicals, eugenol/isoeugenol, in *Escherichia coli*. *Arch. Biochem. Biophys.* **541**, 37–46

45. Xia, Z.-Q., Costa, M. A., Pélissier, H. C., Davin, L. B., and Lewis, N. G. (2001) Secoisolariciresinol dehydrogenase purification, cloning and functional expression: Implications for human health protection. *J. Biol. Chem.* **276**, 12614–12623
46. Huang, K., Li, M., Liu, Y., Zhu, M., Zhao, G., Zhou, Y., Zhang, L., Wu, Y., Dai, X., Xia, T., and Gao, L. (2019) Functional analysis of 3-dehydroquinate dehydratase/shikimate dehydrogenases involved in shikimate pathway in *Camellia sinensis*. *Front. Plant Sci.* **10**, 1268
47. Akagi, T., Ikegami, A., Suzuki, Y., Yoshida, J., Yamada, M., Sato, A., and Yonemori, K. (2009) Expression balances of structural genes in shikimate and flavonoid biosynthesis cause a difference in proanthocyanidin accumulation in persimmon (*Diospyros kaki* Thunb.) fruit. *Planta* **230**, 899–915
48. Tahara, K., Nishiguchi, M., Funke, E., Miyazawa, S. I., Miyama, T., and Milkowski, C. (2021) ehydroquinate dehydratase/shikimate dehydrogenases involved in gallate biosynthesis of the aluminum-tolerant tree species *Eucalyptus camaldulensis*. *Planta* **253**, 3
49. Muir, R. M., Ibanez, A. M., Uratsu, S. L., Ingham, E. S., Leslie, C. A., McGranahan, G. H., Batra, N., Goyal, S., Joseph, J., Jemmis, E. D., and Dandekar, A. M. (2011) Mechanism of gallic acid biosynthesis in bacteria (*Escherichia coli*) and walnut (*Juglans regia*). *Plant Mol. Biol.* **75**, 555–565
50. Ding, L., Hofius, D., Hajirezaei, M. R., Fernie, A. R., Börnke, F., and Sonnewald, U. (2007) Functional analysis of the essential bifunctional tobacco enzyme 3-dehydroquinate dehydratase/shikimate dehydrogenase in transgenic tobacco plants. *J. Exp. Bot.* **58**, 2053–2067
51. Guo, J., Carrington, Y., Alber, A., and Ehlting, J. (2014) Molecular characterization of quinate and shikimate metabolism in *Populus trichocarpa*. *J. Biol. Chem.* **289**, 23846–23858
52. Bischoff, M., Schaller, A., Bieri, F., Kessler, F., Amrhein, N., and Schmid, J. (2001) Molecular characterization of tomato 3-dehydroquinate dehydratase-shikimate:NADP oxidoreductase. *Plant Physiol.* **125**, 1891–1900
53. Bontpart, T., Marlin, T., Vialet, S., Guiraud, J.-L., Pinasseau, L., Meudec, E., Sommerer, N., Cheynier, V., and Terrier, N. (2016) Two shikimate dehydrogenases, *VvSDH3* and *VvSDH4*, are involved in gallic acid biosynthesis in grapevine. *J. Exp. Bot.* **67**, 3537–3550
54. Gritsunov, A., Peek, J., Diaz Caballero, J., Guttman, D., and Christendat, D. (2018) Structural and biochemical approaches uncover multiple evolutionary trajectories of plant quinate dehydrogenases. *Plant J.* **95**, 812–822
55. Cui, L., Yao, S., Dai, X., Yin, Q., Liu, Y., Jiang, X., Wu, Y., Qian, Y., Pang, Y., Gao, L., and Xia, T. (2016) Identification of UDP-glycosyltransferases involved in the biosynthesis of astringent taste compounds in tea (*Camellia sinensis*). *J. Exp. Bot.* **67**, 2285–2297
56. Ono, N. N., Qin, X., Wilson, A. E., Li, G., and Tian, L. (2016) Two UGT84 family glycosyltransferases catalyze a critical reaction of hydrolyzable tannin biosynthesis in pomegranate (*Punica granatum*). *PLOS ONE* **11**, e0156319
57. Madeira, F., Madhusoodanan, N., Lee, J., Eusebi, A., Niewielska, A., Tivey, A. R. N., Lopez, R., and Butcher, S. (2024) The EMBL-EBI Job Dispatcher sequence analysis tools framework in 2024. *Nucleic Acids Res.* **52**, W521–W525
58. Letunic, I., and Bork, P. (2021) Interactive Tree Of Life (iTOL) v5: an online tool for phylogenetic tree display and annotation. *Nucleic Acids Res.* **49**, W293–W296
59. Ross, J., Li, Y., Lim, E., and Bowles, D. J. (2001) Higher plant glycosyltransferases. *Genome Biol* **2**, REVIEWS3004
60. Kim, K.-W., Moinuddin, S. G. A., Atwell, K. M., Costa, M. A., Davin, L. B., and Lewis, N. G. (2012) Opposite stereoselectivities of dirigent proteins in *Arabidopsis* and *Schizandra* species. *J. Biol. Chem.* **287**, 33957–33972

61. Katsuyama, Y., Kita, T., Funa, N., and Horinouchi, S. (2009) Curcuminoid biosynthesis by two type III polyketide synthases in the herb *Curcuma longa*. *J. Biol. Chem.* **284**, 11160–11170
